# VGGish-based detection of biological sound components and their spatio-temporal variations in a subtropical forest in eastern China

**DOI:** 10.7717/peerj.16462

**Published:** 2023-11-15

**Authors:** Mei Wang, Jinjuan Mei, Kevin FA Darras, Fanglin Liu

**Affiliations:** 1 Division of Life Sciences and Medicine, University of Science and Technology of China, Hefei, China; 2 Sustainable Agricultural Systems & Engineering Lab, School of Engineering, Westlake University, Hangzhou, China; 3 Hefei Institutes of Physical Science, Chinese Academy of Sciences, Hefei, China

**Keywords:** Soundscape, VGGish, Clustering, Birds, Insects, Biophony, Ecoacoustics

## Abstract

Passive acoustic monitoring technology is widely used to monitor the diversity of vocal animals, but the question of how to quickly extract effective sound patterns remains a challenge due to the difficulty of distinguishing biological sounds within multiple sound sources in a soundscape. In this study, we address the potential application of the VGGish model, pre-trained on Google’s AudioSet dataset, for the extraction of acoustic features, together with an unsupervised clustering method based on the Gaussian mixture model, to identify various sound sources from a soundscape of a subtropical forest in China. The results show that different biotic and abiotic components can be distinguished from various confounding sound sources. Birds and insects were the two primary biophony sound sources, and their sounds displayed distinct temporal patterns across both diurnal and monthly time frames and distinct spatial patterns in the landscape. Using the clustering and modeling method of the general sound feature set, we quickly depicted the soundscape in a subtropical forest ecosystem, which could be used to track dynamic changes in the acoustic environment and provide help for biodiversity and ecological environment monitoring.

## Introduction

Biodiversity is declining globally (Balvanera et al., 2006) due to human activities and global environmental change (Cardinale et al., 2012). Monitoring and tracking biodiversity change is an essential task of global governance (Pollock et al., 2002; Johnson et al., 2017). Sound is a significant element of animal behavior and is used for communication (Hart et al., 2015). Animals’ vocal activity plays roles in territory defense, mate attraction, orientation, prey localization, predator escape, *etc*. (Buxton et al., 2018). Passive acoustic monitoring technology (Van Parijs et al., 2009) can help collect data on large temporal and spatial scales, providing a promising solution for the biodiversity assessment of vocalizing animals at a large scale, such as birds, bats, marine mammals, and insects (Dumyahn & Pijanowski, 2011b; Kasten et al., 2012; Duarte et al., 2021), whose vocalizations often act as indicators for biodiversity assessments (Gregory et al., 2005). Passive acoustic monitoring can reduce human labor in field investigations and the observers’ potential impact on animal activity (Darras et al., 2019; Sugai & Llusia, 2019; Ross et al., 2021). In addition to biological sounds, it also records the geophysical environment and human-made sounds in the landscape, which are essential components of the soundscape (Pijanowski et al., 2011). Therefore, the information in audio recordings can also help understand and predict the profound impacts of human activities and environmental change on biodiversity (Burivalova et al., 2018).

Extensive acoustic recordings have been collected from a multitude of habitats around the world, but methods for translating these data into a rapid monitoring process have not been keeping pace (Bradfer-Lawrence et al., 2019; Duarte et al., 2021). In most cases, experts are required to analyze the spectrogram or playback the audio when inferring a target species’ presence, abundance, decline, or spatiotemporal patterns (Figueira et al., 2015; Mei et al., 2022). Unfortunately, it is time-consuming to manually process a large number of recordings (Wimmer et al., 2013). Researchers have developed automatic recognizers such as Kaleidoscope Pro (Merchant et al., 2015; Abrahams & Geary, 2020), WEKA (Frank, Hall & Witten, 2016), Song Scope (Pérez-Granados et al., 2019), and monitoR (Katz, Hafner & Donovan, 2016). However, building an automatic recognizer takes time and skill, and it can be prone to a high error rate (false negatives and false positives), especially in noisy field recordings (Terry, Peake & McGregor, 2005; Priyadarshani, Marsland & Castro, 2018; Dufourq et al., 2021).

Acoustic indices provide alternative solutions for the automatic analysis of a large number of recordings (Bradfer-Lawrence et al., 2019). Rather than focusing on the detection of individual species, acoustic indices measure variations in acoustic activity, predominantly statistical summaries of the amplitude variation in time domains, or the magnitude differences between the frequency bands of a spectrogram, such as temporal entropy index, spectral entropy index (Sueur et al., 2008), the acoustic diversity index (Villanueva-Rivera et al., 2011), normalized difference soundscape index (Kasten et al., 2012), and acoustic complexity index (Pieretti, Farina & Morri, 2011). These indices can evaluate variation in animal activities (Sueur et al., 2008; Pieretti, Farina & Morri, 2011), as well as supporting habitats and biodiversity assessments (Gage et al., 2017; Borker et al., 2019; Yip et al., 2021), or estimating species richness (Buxton et al., 2018) without information about the species that are present. With the development of unsupervised clustering technology, various acoustic indices combined with k-means, hierarchical, or Gaussian mixture model clustering algorithms can be used to obtain different soundscape categories from diverse sound sources (Lin, Tsao & Akamatsu, 2018; Phillips, Towsey & Roe, 2018; Kannan, 2020). The diel variation and seasonal patterns can be further analyzed according to the soundscape categories (Flowers et al., 2021). In addition, the relationship between components of acoustically rich soundscapes can help to reflect the complex social and ecological interactions in animal communities (Farine, Whitehead & Altizer, 2015; Wang et al., 2019), which can be aided by social network analysis, a data analytics method that uses networks and graph theory to understand social structures (Butts, 2008). Day-to-day changes in soundscape categories in an environment or different sites can be distinguished *via* social network analysis when considering each category individually (Wang et al., 2019). However, the choice of the acoustic index and its performance is limited by the survey scale and the ecosystem type (Mammides et al., 2017); for example, inland birds’ vocal activities and the seabird recovery following invasive predator removal require different acoustic indices (Gage et al., 2017; Borker et al., 2019). In addition, index values may be biased by the presence of abiotic and anthropogenic sounds (Lin, Fang & Tsao, 2017a). Therefore, further research on general acoustic features that describe the soundscape is needed.

Deep learning technology has been applied to audio tasks (such as speech and music), providing alternative solutions for big data analysis in ecoacoustics research (Hershey et al., 2017). The critical innovation of acoustic deep learning in audio recognition is based on convolutional neural networks (CNNs), that eliminate the manual design step and keep the input in a much higher dimensional format, thus allowing much richer information to be presented (Hershey et al., 2017). Models based on CNN architecture include ResNet (He et al., 2016), VGG (Simonyan & Zisserman, 2015), VGGish (Hershey et al., 2017), DenseNet (Huang et al., 2017), AlexNet (Krizhevsky, Sutskever & Hinton, 2012), Inception (Szegedy et al., 2014), LeNet (Lecun et al., 1998), MobileNet (Sandler et al., 2018), EfficientNet (Tan & Le, 2019), Xception (Chollet, 2017), CityNet (Fairbrass et al., 2018), BirdNet (Kahl et al., 2021), *etc*. For example, the acoustic analysis system CityNet uses CNNs for measuring audible biotic and anthropogenic acoustic activity in audio recordings from urban environments (Fairbrass et al., 2018). BirdNET, the model architecture derived from the ResNets and using extensive training data, can identify different bird species by sound (Kahl et al., 2021). The acoustic features generated by the VGGish model can serve as ecological indicators to replace acoustic indices (Sethi et al., 2020). VGGish is a configuration based on the VGG image classification model (Simonyan & Zisserman, 2015) and is pre-trained by Google’s AudioSet (Gemmeke et al., 2017; Hershey et al., 2017). AudioSet contains over two million labeled audio samples drawn from various sources appearing on YouTube so that the resulting VGGish acoustic features can perform general-purpose audio classification (Hershey et al., 2017). VGGish generates 128-dimensional feature embedding, which can efficiently capture audio characteristics and be used as the input of downstream models (Sethi et al., 2020). The VGGish feature embedding has been used to identify anomalous events based on Gaussian mixture model clustering; it was shown to contain ecological information that describes temporal and spatial trends in different habitats and is more general and has higher resolution compared with various acoustic indices (Sethi et al., 2020).

In this study, we explore a data-driven solution for overcoming the limitation of acoustic indices and distinguishing biological sounds within diverse sound sources in a soundscape. We address the hypothesis that the general, high-resolution VGGish feature embedding is able to identify biological sound components of a soundscape and detect their temporal-spatial variations. Using the VGGish model, 128-dimensional acoustic features were obtained from recordings, and an unsupervised clustering model was used to distinguish different sound components. We conducted this by investigating the soundscape of a high-elevation subtropical forest in eastern China, where the seasonal variation in bioacoustic activities was important (Lin et al., 2017b). In particular, we investigated whether there was a spatiotemporal difference in acoustic spaces between birds and insects because they vigorously compete for acoustic space (Hart et al., 2015).

## Materials and Methods

### Study area

The Yaoluoping National Nature Reserve area (YNNR) (Fig. S1), located in the hinterland of the Ta-pieh Mountains, spans the belt between the north subtropical and temperate zones. YNNR covers a 123 km^2^ area, including the core (21.2 km^2^), buffer (28.4 km^2^), and experimental (73.4 km^2^) areas. The reserve area is situated at the junction of the north subtropical alpine forest ecosystem and is dominated by the subtropical evergreen broad-leaved forest and warm temperate deciduous broad-leaved forest (Xie & Wu, 1995). The zoogeographic region divisions of YNNR include the southern limit, which belongs to the Huanghuai Plain sub-region of the ancient northern region, and the northern limit, which belongs to the hilly plain sub-region of the eastern Dongyang region. Due to its unique geographical location, more than one hundred species of birds (Li et al., 2017) and hundreds of insect species (Shu et al., 2008; Jingmin et al., 2013) live in YNNR, constituting a unique soundscape for ecoacoustic research.

### Acoustic data acquisition

We selected six representative sites in YNNR (Fig. S1) to sample the protected area. Their elevations spanned a gradient: 1,341 m for site 1, 1,211 m for site 2, 1,180 m for site 3, 1,090 m for site 4, 805 m for site 5, and 689 m for site 6. Of these sites, sites 2, 3, and 4 were located in the buffer, and the others were in the experimental area. The experimental zone allows scientific research, teaching practice, and a specific range of human production activities, while the buffer zone only allows scientific research and observation (Song et al., 2021). The vegetation around all the sites is deciduous broad-leaved forest and evergreen mixed forest, and there are coniferous forests near sites 2 and 3.

Access to the YNNR is governed by Forest Law and Regulations of the People’s Republic of China on Nature Reserves. The sound sampling was conducted under the permission of the YNNR forest managers. On public land, audio data can be captured according to the Department of Ecology and Environment of Anhui Province that allows non-destructive scientific research observations. No materials were collected from the field, and the deployment of automated sound recorders was restricted to six sites which were rarely visited by humans. Furthermore, these sites were approximately 100 to 400 m away from the public tracks. Besides, we displayed posters on the public tracks near recording sites to instruct visitors to remain silent because of ongoing audio recording for research purposes. Despite having limited resources, we randomly checked 10% of the recorded audio data manually, as we did not have the capacity to screen the massive amount of sound recordings for human activity. Logging and hunting are strictly prohibited in this area according to the Regulations of the People’s Republic of China on Nature Reserves. We did not find any evidence of illegal human activity such as chainsaw or gunshot sounds, or private human conversations in this subset and double-checked public uploaded recordings to ensure no ethical issues. Recordings with human voices only included loudspeaker-broadcast recorded advertisements (common in many natural areas in China to inform visitors of applicable laws). Results of the acoustic survey were not shared with other institutions or individuals.

At each site, an automated sound recorder with two SM4 stub microphones (SM4+,Wildlife Acoustics, Maynard, MA, USA) was fixed on a tree trunk about 1.5 meters above the ground. All the acoustic recorders were scheduled to record the first 5 min of every half hour from 0:00 to 23:59 h. The recordings were saved in the WAV stereo format on secure digital (SD) cards at a sampling rate of 24,000 Hz and 16 bit-depth on each channel. We obtained a maximum sound frequency of 12,000 Hz, which included most birds and some insects that we were interested in. For all sites, the recordings lasted six months, from April 5, 2019, to October 6, 2019, producing a total of 4,240.5 h of audio recordings. Some data were lost due to battery replacement and equipment damage.

### VGGish feature embeddings and clustering

**Figure 1:**
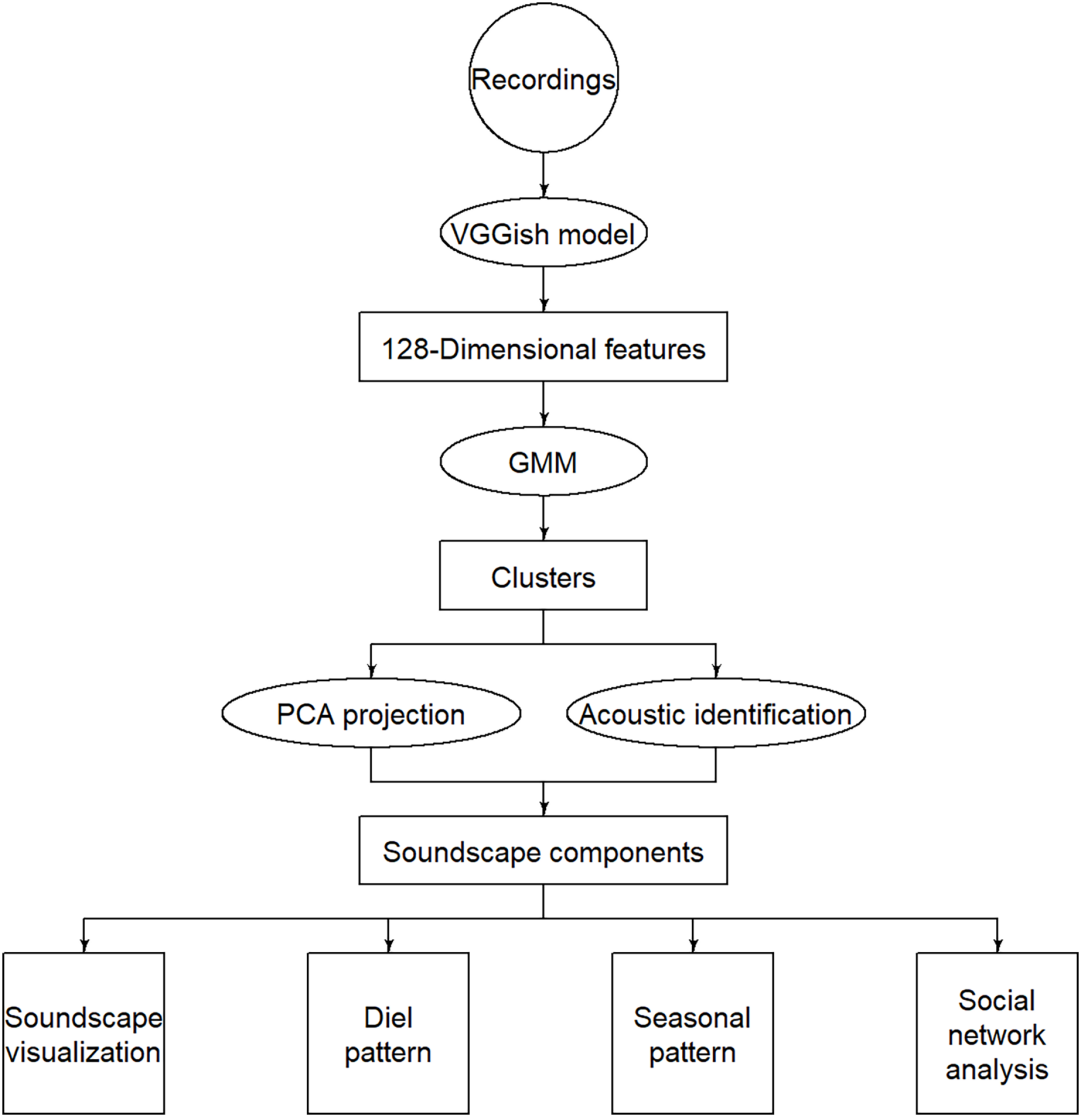
Flow chart of the methodology. The circles represent inputs, ellipses represent operations, rectangles represent intermediate outputs, and squares represent final outputs.

The VGGish model is pre-trained by Google’s AudioSet project using a preliminary version of the YouTube-8M dataset (Hershey et al., 2017). This website https://doi.org/10.5281/zenodo.3907296 (Sethi, 2020) provides the pre-trained VGGish model and code; when inputting audio data, it can compute the 128-dimensional acoustic feature embedding for every 0.96 s of audio. As shown in the flow chart (Fig. 1), we input each 5-min audio into the VGGish model, and the output was 128 features at every 0.96 s window, so the output data were 312 × 0.96 s × 128 for every 5-min recording. Then, we averaged the acoustic feature vectors over consecutive 1-min periods (62 × 0.96 s, *i.e*., 59.52 s) to account for the high stochasticity of short audio samples.

For all extracted VGGish features, an unsupervised learning technology was used to recognize different sound sources. Euclidean distance-based k-means and hierarchical clustering are often used for audio classification, and the clustering performance is subject to the selection of acoustic features (Phillips, Towsey & Roe, 2018). A Gaussian mixture model is a probabilistic model that assumes the data are generated from a mixture of a finite number of Gaussian distributions. It can be regarded as an optimization of the k-means model and is expected to reconstruct individual sound sources. Therefore, the feature embedding was fed to a Gaussian mixture model for clustering in order to separate the categories from various confounding sound sources without prior information about the acoustic community. In order to determine the optimal number of clusters, the Gaussian classes can use the Bayesian information criterion (BIC) as a discriminant (Fraley & Raftery, 1998; Clavel, Ehrette & Richard, 2005). BIC balances error minimization (more clusters reduce error) with model complexity (more clusters increase complexity) (Phillips & Towsey, 2017), which is an effective method to measure the clustering quality (Xu & Wunsch, 2005). We calculated the BIC for 5 to 200 clusters in step 5 to find the optimal cluster number where the BIC reached a relative minimum (Fig. S2). The clustering results are difficult to interpret in high-dimensional feature spaces, so we used principal component analysis (PCA) to reduce dimensions for visualization. The principal components are linear combinations of the original variables, which reduces variables while minimizing information loss (Jolliffe & Cadima, 2016). The centers and covariances of each Gaussian mixture model component were projected from 128 dimensions into two principal components (PCA1 and PCA2), so the distribution of each sound component can be shown on a 2-dimensional plane.

### Sound components identification

Once the clusters were calculated, we checked ten recordings (each of 1-min duration) closest to the Gaussian mixture model center to determine their sound component type (Flowers et al., 2021). The clusters were identified by listening to the audio recordings and visually inspecting their spectra using Raven Pro 1.5 software (Bioacoustics Research Program, 2014), which was used to play the recordings and annotate the spectrogram to help identify the sound components for each cluster. Here, we could only distinguish the biological sounds of different vocal communities and abiotic sounds qualitatively instead of identifying all species exhaustively.

According to the investigation of known vocal organisms in the area, the primary vocal organisms are insects and birds (Shu et al., 2008; Jingmin et al., 2013; Li et al., 2017). Combined with the scheme used to determine the acoustic content of each cluster in other research (Phillips, Towsey & Roe, 2018), we summarized the 95 sound clusters into seven main sound component types (hereafter “components”: ‘mainly bird’, ‘mainly insect’, ‘mainly rain’, ‘no obvious biophony’, ‘bird and insect’, ‘bird and rain’, and ‘biophony and anthropophony’). The representative audio samples of each cluster can be checked at https://ecosound-web.de/ecosound_web/collection/show/45 (Darras et al., 2023).

### Spatio-temporal variations analysis

The function *image* of the R programming language (R Core Team, 2019) was used to visualize the distribution of each of the seven sound components. Soundscape visualization can intuitively display the dynamic changes of sound components by hour, month, and site. The proportion of sound components at different times of the day can show their daily patterns. We counted the daily number of sound components for each month and displayed the condensed information with boxplots. Soundscape component proportions were also calculated for the different sites to understand their spatial variation. In addition to analyzing the seven main sound component types, we used social network analysis to grasp the relationship between the 95 clusters similarly to previous analyses of natural habitats (Wang et al., 2019): Nodes (*i.e*., clusters) can represent a variety of actors, and edges (*i.e*., cluster connections) can represent a variety of relationships. Based on the Spearman correlation of 95 clusters, social network analysis was carried out using the *igraph* package (R Core Team, 2019).

## Results

### (Main) sound component (type)s of the soundscape

**Figure 2:**
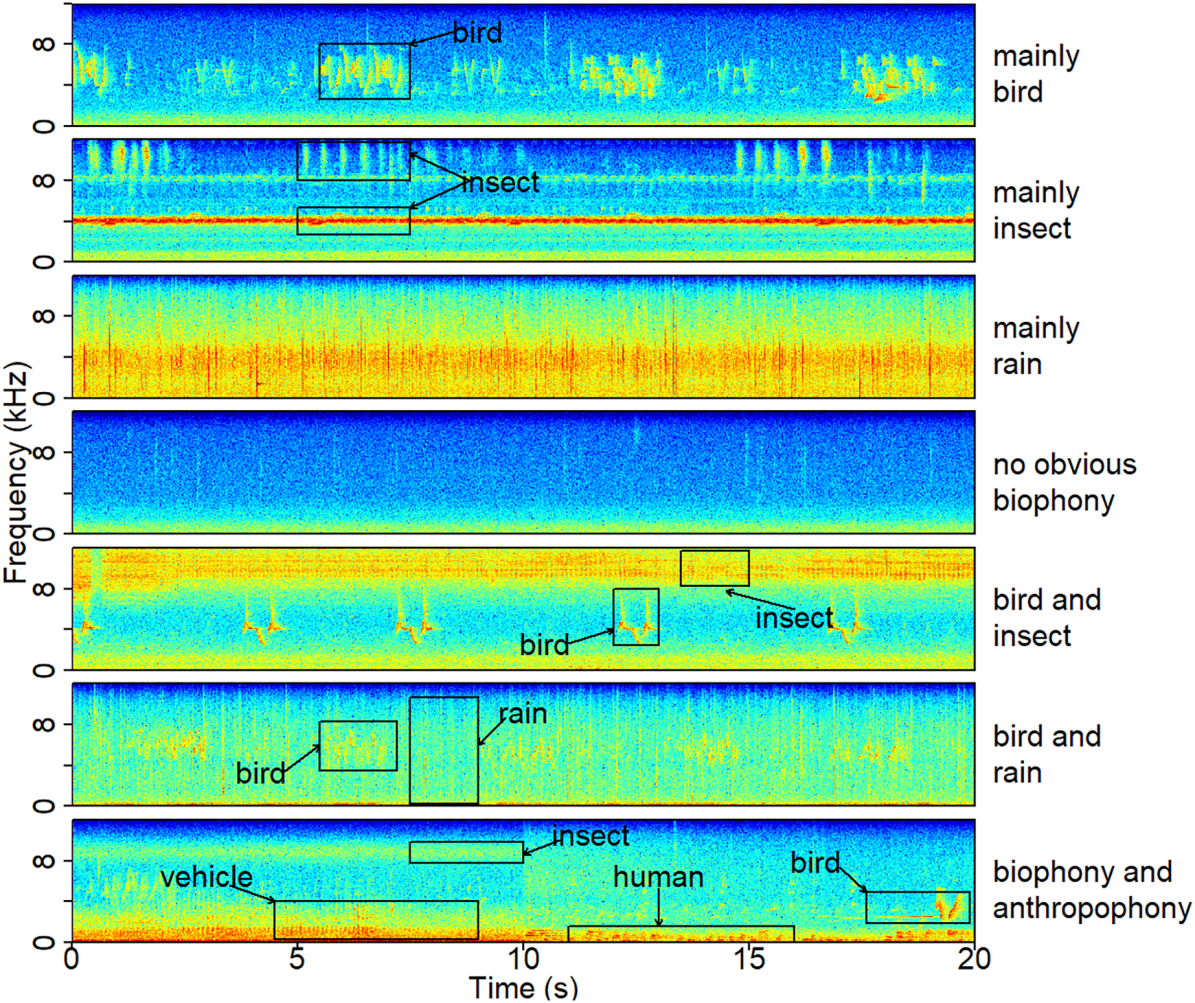
A typical spectrogram of each sound component. The spectrograms were computed using a Hann window, FFT = 512, window overlap of 50%, and frame size of 100%. The ‘no obvious biophony’ component has no clear biological sound or rain sound. The ‘biophony and anthropophony’ component contains bird, insect, vehicle, and human sounds. Representative recordings can be found under https://ecosound-web.de/ecosound_web/collection/show/45.

**Figure 3:**
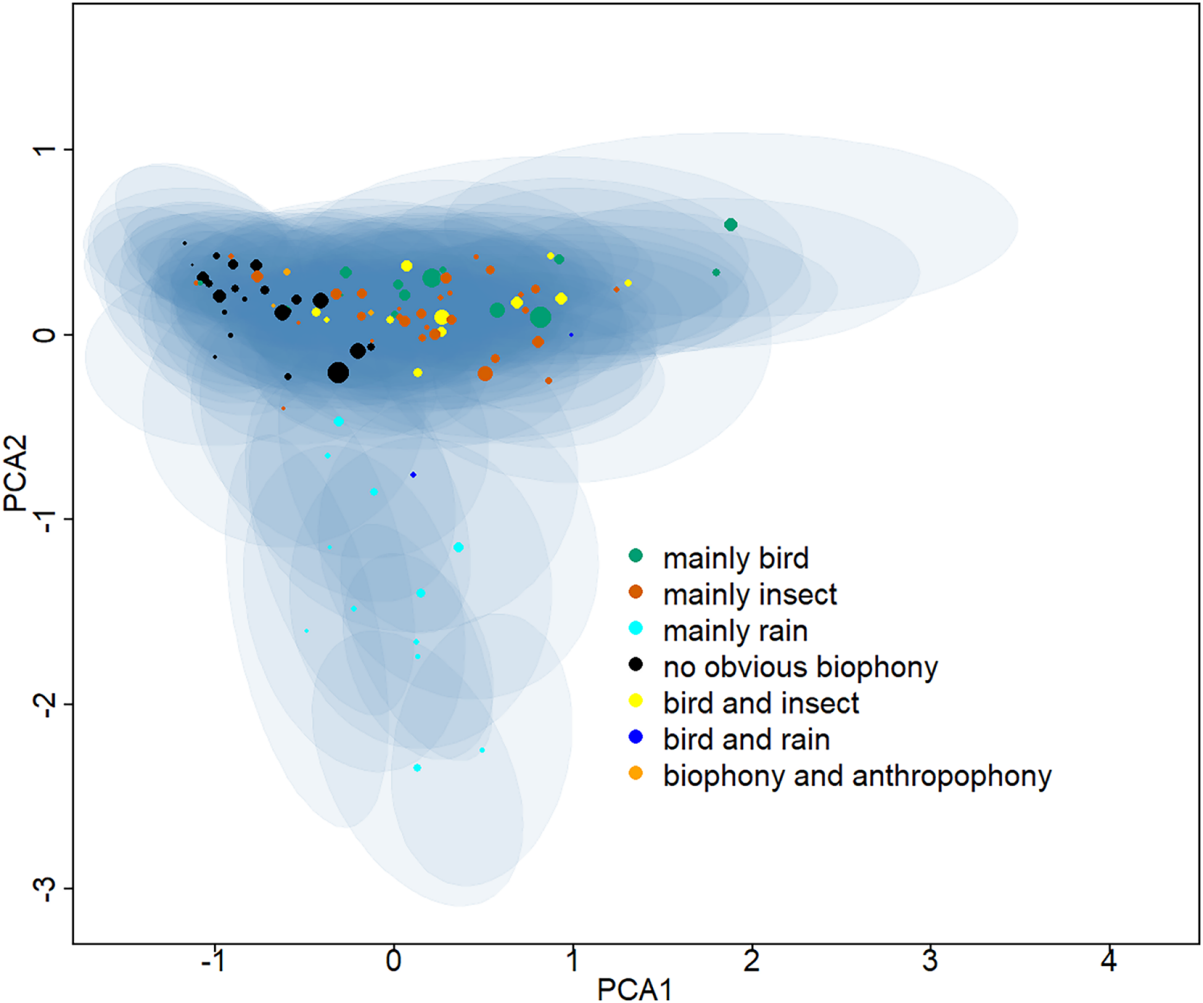
Two-dimensional projection of the Gaussian mixture model clustering results. The Gaussian mixture model center and covariance were projected from 128 dimensions to two dimensions using principal component analysis. The centers of different clusters belong to seven soundscape components and are displayed in different colors, and shaded areas correspond to two standard deviations from each Gaussian mixture model center. The larger the point, the greater the weight of its corresponding Gaussian mixture model.

Ninety-five clusters were summarized into seven sound components (Table S1), whose typical spectrograms are shown in Fig. 2. Many bird songs could be seen in the ‘mainly bird’ sound component, and 14 of the 95 clusters were classified as ‘mainly bird’. The ‘mainly insect’ component is represented by continuous chirping, and 30 clusters were classified as this sound component. There are 12 clusters identified as ‘mainly rain’, 22 clusters identified as ‘no obvious biophony’, and 12 categories belonged to ‘bird and insect’. In addition, two clusters contained the sound of both rain and birds and were identified as a ‘bird and rain’. Among the remaining three clusters, not only the sound of birds and insects but also obvious artificial sounds, such as car engines, speech, and shouting, can be heard, which were consequently classified as a ‘biophony and anthropophony’. Representative spectrograms of 95 clusters can be seen in the (Information S3). The projection of the Gaussian mixture model clustering results shows the differences between clusters in two dimensions (Fig. 3). In the dimension of PCA2, the ‘mainly rain’ and other components can be distinguished. The ‘no obvious biophony’ is on the left of PCA1, and other components are on the right of PCA1.

### Spatio-temporal pattern

**Figure 4:**
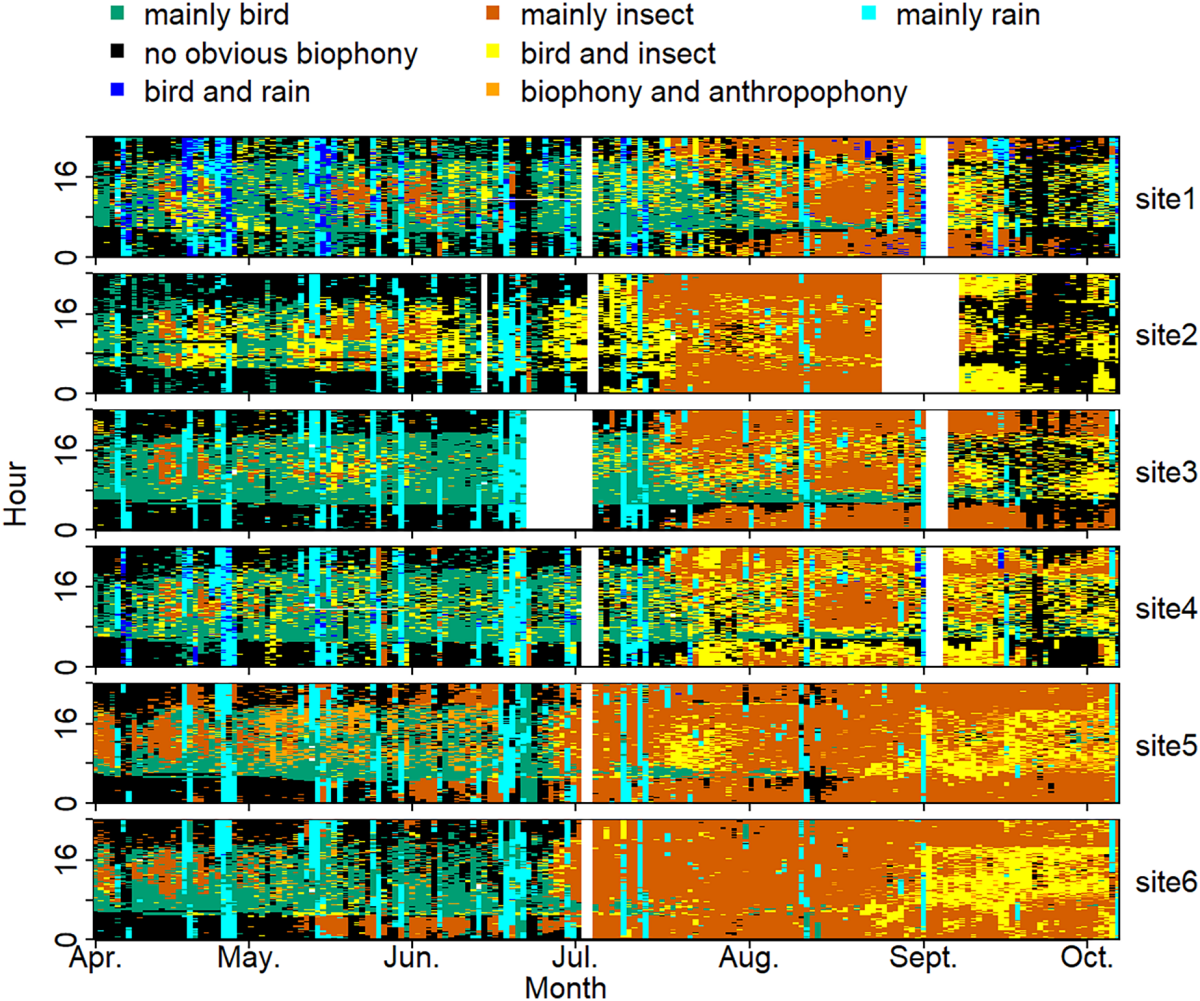
Spatio-temporal soundscape patterns in six sampling sites with different components represented by different colors. The X-axis represents different months, and the Y-axis represents the daytime (0–24 h). The elevation gradually decreases from site 1 to site 6 (from 1,341 to 689 m). The blanks represent missing data due to audio data loss.

**Figure 5:**
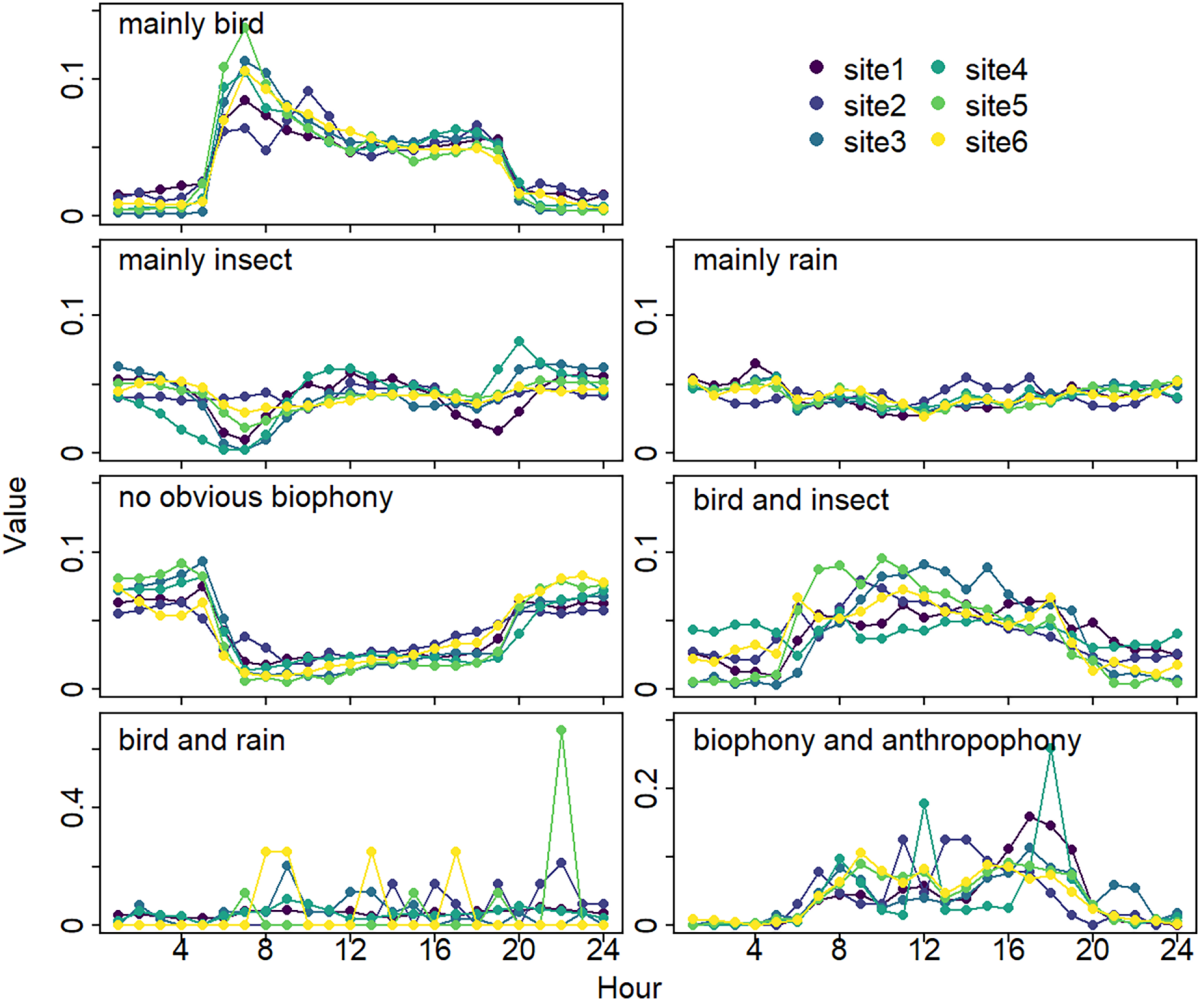
Diurnal changes of sound components. The X-axis represents hours, and the Y-axis represents the proportion of the number of sound components in different hours. Different colors correspond to different sites.

**Figure 6:**
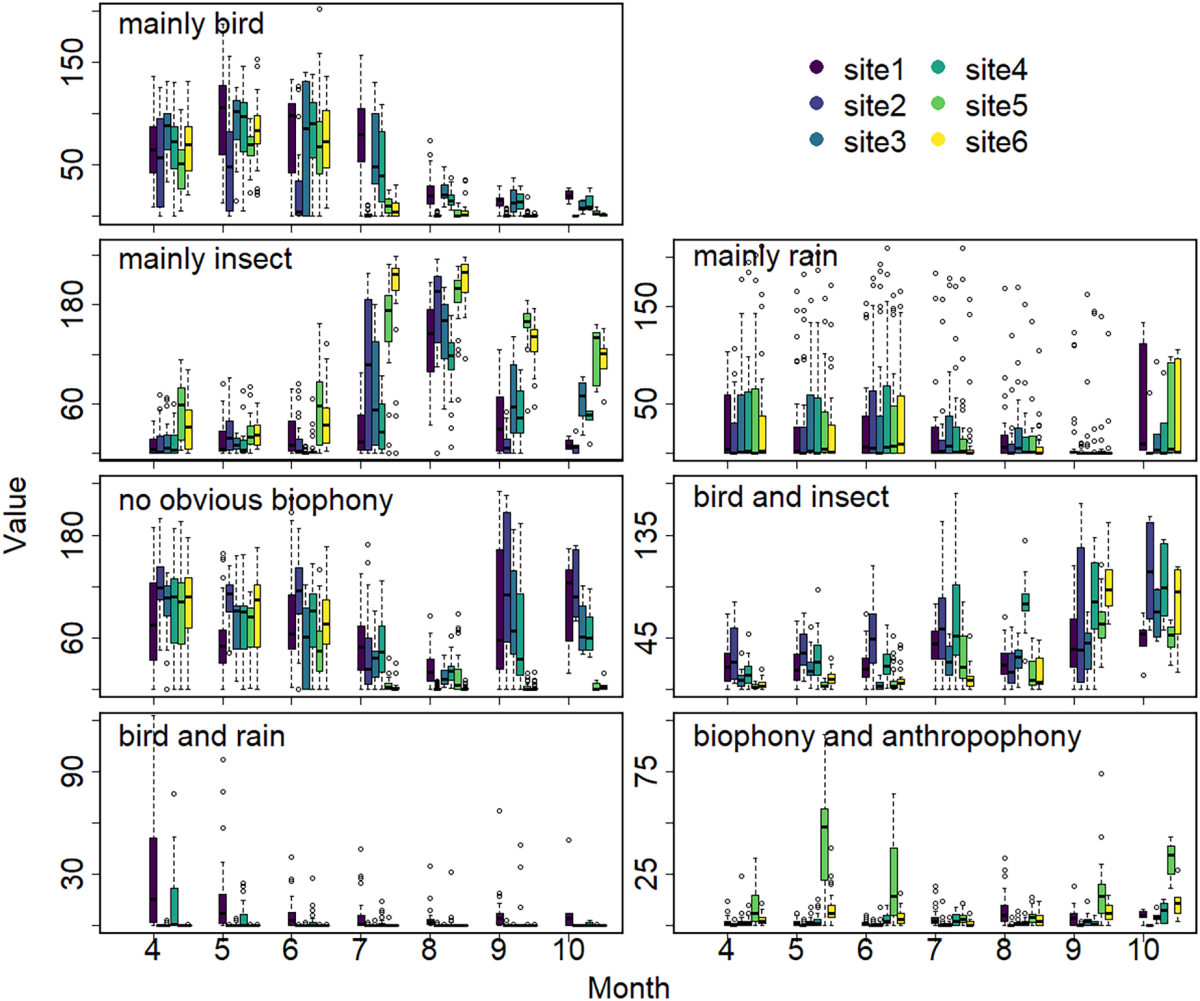
Seasonal changes of sound components. The X-axis exhibits months, and the Y-axis exhibits the number of sound components (the boxplot summarizes total days of the respective months). Different colors correspond to different sites.

The sound components form a diverse soundscape (Fig. 4). The visualization diagram simultaneously displays the changes in different sound categories that occurred within 24 h, on different days, and at different sites. The diel pattern of each sound component is different (Fig. 5). The ‘mainly bird’ component appeared most in the daytime, with a first peak at dawn and a second peak at dusk. The ‘mainly insect’ component appeared during both the day and night. Comparing ‘mainly bird’ and ‘mainly insect’ components, when the sound of the birds reached a peak, insects had a trough. The component ‘no obvious biophony’ mainly occurred at night. There is no obvious diurnal time trend for the ‘mainly rain’ sound component. The ‘biophony and anthropophony’ mainly occurred during the day. The monthly variation shows the seasonal pattern (Fig. 6). The ‘mainly bird’ component appeared from April to June, and the ‘mainly insect’ category appeared from August to October. The ‘no obvious biophony’ is the smallest in August, while the insect component appears most frequently in that month.

**Figure 7:**
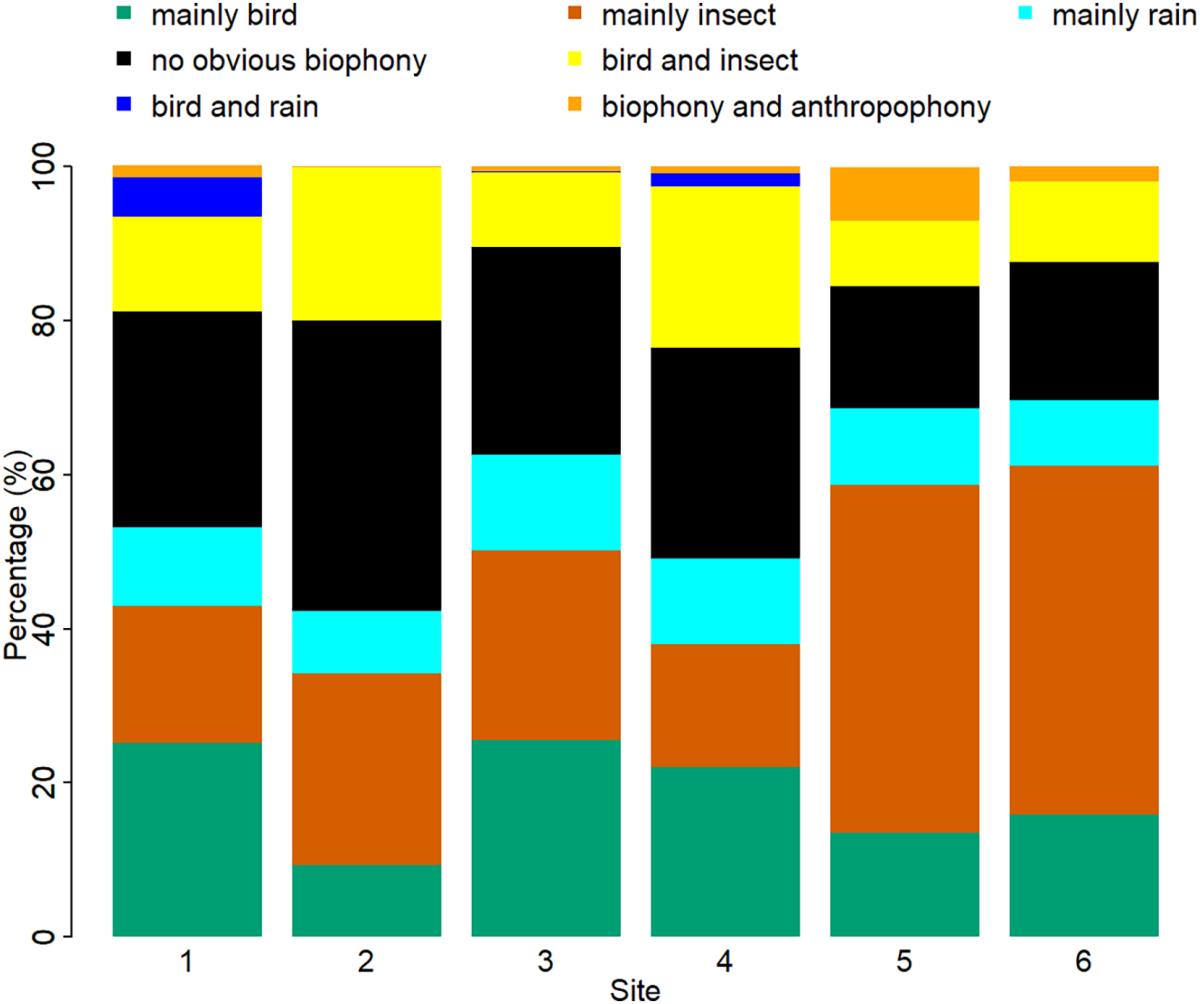
Percentage of different soundscape components in different sites. The X-axis represents six sites, and the Y-axis represents the percentage of each soundscape component. The elevation gradually decreases from site 1 to site 6 (from 1,341 to 689 m).

The sound components for each location are unique, and the biological sound varies along the altitude direction (Fig. 7, Table S2). The proportion of the ‘mainly bird’ component at sites 1-6 was 25.10%, 9.38%, 25.62%, 22.02%, 13.53%, and 15.93%, respectively. The proportion of the ‘mainly insect’ component at sites 1-6 was 17.80%, 24.82%, 24.59%, 15.96%, 45.13%, and 45.20%, respectively. The ‘mainly rain’ component occupied about 10% of all the sites. Compared with other sites, the ‘no obvious biophony’ component accounted for the largest proportion in site 2. The ‘biophony and anthropophony’ component occupied a higher proportion in site 5 than in other sites.

### Network analysis

**Figure 8:**
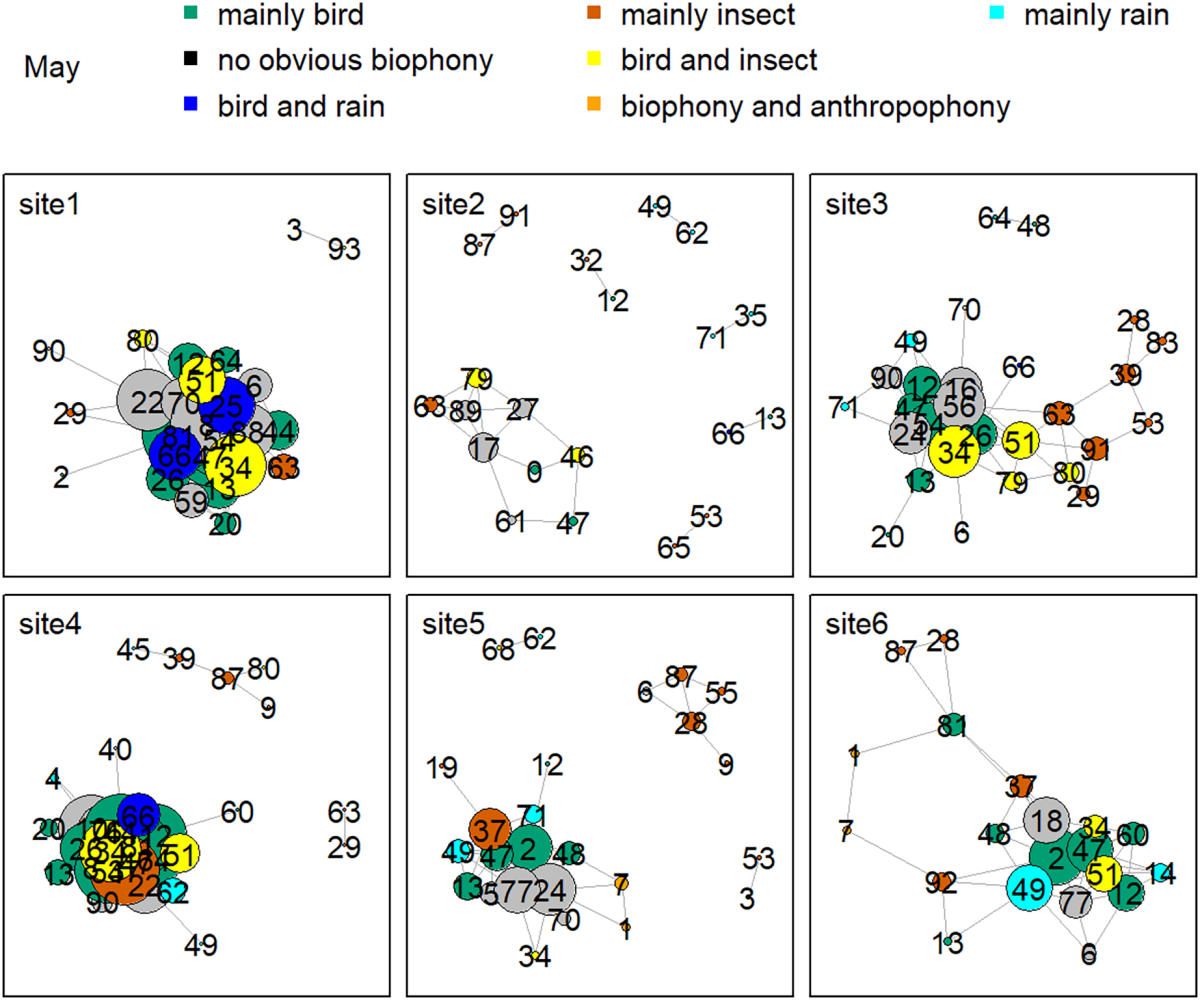
Network map of different clusters at six sites in May. The nodes represent each cluster, the edges represent cluster association, and a large dot size represents a large degree. The different clusters belong to seven soundscape components displayed in different colors.

**Figure 9:**
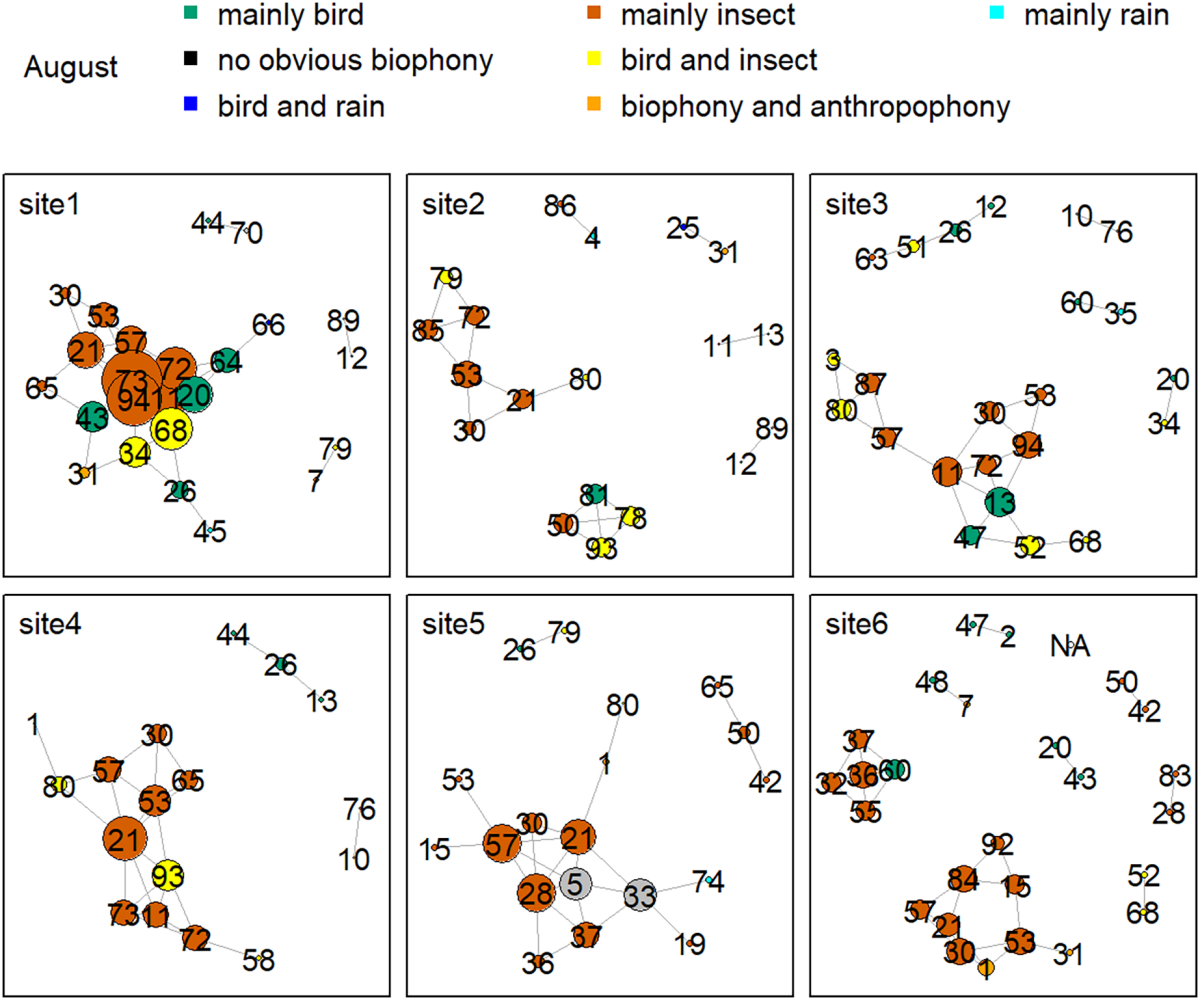
Network map of different clusters at six sites in August. The nodes represent each cluster, the edges represent cluster association, and a large dot size represents a large degree. The different clusters belong to seven soundscape components displayed in different colors.

Figure 8 shows the network relationship of different locations in May. The clusters for each location constitute a unique social network relationship. In site 1, clusters 12, 13, 20, 26, 44, 47, 51, 61, and 81, which belong to the ‘mainly bird’ component, had more links, while cluster 0 and 47 in site 2 had fewer connections. Figure 9 is the network relationship diagram for the different locations in August. Unlike May, the important nodes this month mostly belong to the ‘mainly insect’ component. See Figs. S3–S7 for the network diagram of other months. Using the social network analysis map, we can quickly find the acoustic cluster differences in multiple months and sites.

## Discussion

Our study provides a quantitative and visual description of the biological sound sources of a forest soundscape and their spatiotemporal variations using 128-dimensional VGGish feature embedding and unsupervised clustering. Birds and insects were the two primary biophony sound sources, and their sounds displayed distinct temporal patterns across 24 h as well as months and distinct spatial patterns in the landscape. Soundscape conservation has become increasingly focused on protected areas of the earth (Irvine et al., 2009; Dumyahn & Pijanowski, 2011a; Monacchi, 2013). Our work illustrates the application of VGGish feature embedding to identify biological sound sources and provides a valuable baseline for soundscape conservation in this region. Because the VGGish model is independent of ecosystem-specific data or human expertise, it may be explored as a general, data-driven solution for acoustic-based biodiversity monitoring and soundscape conservation.

An acoustic cluster may contain multiple types of sound because sometimes sounds from different sources occur at the same time. Although it is not easy to use a single element, such as biological or geophysical sound, to represent a specific cluster, clustering can still broadly represent different sound types. Anthropophony, geophony, and biophony present different intensity values or variability in intensity modulation (Farina et al., 2016). Most birds vocalize every few seconds during their activity time, whereas the sounds of insects may last for several minutes. Existing research shows that various acoustic indices and k-means clustering can be used to discriminate soundscape categories (Phillips, Towsey & Roe, 2018) and study the differences between urban and rural soundscapes (Flowers et al., 2021). Additionally, different acoustic index combinations have been used to detect rainfall (Ferroudj et al., 2014). Here, our results show that diverse soundscapes can be automatically divided into distinct components representing different biophony sound types, mixtures of sound types, as well as anthropophony and geophony sound types. We used general acoustic features to distinguish biological and non-biological sounds from the soundscape without selecting a specific acoustic index. This means that VGGish feature embeddings, when combined with the unsupervised clustering method, made good use of the characteristics of different sound types to be used for effective classification.

The soundscape visualization enables long-time audio recordings to be depicted on a graph, which is more convenient for the reserve manager to monitor the activity of vocal organisms. There are hundreds of birds in YNNR, and the sound clusters dominated by birds display a peak in vocal activity in the morning and a secondary lower activity peak toward sunset. Many birds increase vocal production in the morning to guard their territories or attract mates (Puswal et al., 2022). Studies on the activities of several birds in the region have also given the same results, which shows that the time pattern we discovered based on category analysis is consistent with the statistics of specific species (Mei et al., 2022; Puswal et al., 2022). Our research also shows that the sound clusters dominated by birds increased gradually from late April and reached a maximum level during May and June before starting to decrease after July. The seasonal distribution of birds in this area is related to the pattern of several known passerine birds (Puswal, Jinjun & Liu, 2021). Our cluster reflects the contribution of various birds rather than a specific bird. In addition to resident birds, migratory birds spend the summer here, increasing bird activities in these months since the YNNR is a transitional zone between the north and the south (Li et al., 2017). Insects are also major contributors to biological sounds in YNNR. Compared with birds, the sound clusters dominated by insects increase at night, which is consistent with the activity of insects such as cicadas (Sousa-Lima et al., 2018).

Birds’ signals will interfere with cicada sounds if they have the same frequency (Sousa-Lima et al., 2018; Schilke et al., 2020). For example, birds shut down their vocalizations at the onset of cicada signals that utilize the same frequency range or start vocalizing at non-overlapping frequencies (Hart et al., 2015). Birds also delay their songs when their frequency bands are shared by nocturnal insects to avoid acoustic masking (Hart et al., 2015). Our daily activity results produced the same findings: when birds’ sounds increased during the dawn chorus, the insects’ sounds decreased in the mornings. In addition, our study showed that birds vocalized at sites with high elevations and mainly in May and June, whereas insect sounds were found at sites with low elevations, and their signals mainly occurred in August. The temporally and spatially differentiated patterns between bird and insect sounds prevented masking each other. Our findings significantly improve our understanding of how the temporal and spatial dynamics shape biophony patterns in a forest. In addition to the differences between bird and insect communities, social network analysis shows that there are also differences within the community. For example, more bird clusters have connections in site 1, while fewer clusters are in site 2 in May. Species communities may vary across sites: for example, the *Cuculus saturatus* calls were only found at some sites in YNNR (Mei et al., 2022). The distinct clusters across sites suggest differences in the internal soundscape structure.

Although our six sampling sites belong to the same ecosystem type, the proportion of sound components and the dynamics of soundscapes are different. As shown in Fig. 7, sites 1, 3, and 4 are similar, while sites 5 and 6 are alike. These differences among sites are mainly due to geographical factors since they share similar weather conditions. It appears that higher altitude sites are more suitable for bird sounds, whereas lower altitudes are more suitable for insect sounds. These soundscape relationships help our understanding of why seasonal variations in bioacoustic activities are most evident in high-elevation forests (Lin, Fang & Tsao, 2017a). However, site 2 has fewer birds even though it is at a higher altitude; this may be because site 2 is closer to a freshwater stream, whose ambient noise may interfere with the communication of vocal organisms. The difference in vegetation type also affects the existence of organisms; different species inhabit different altitudes and have specific preferences for vegetation (de Andrade et al., 2014; Shao, Zhang & Yang, 2021), which can also affect the sound detection spaces of the recorders (Darras et al., 2016), that are also variable with time (Haupert, Sèbe & Sueur, 2022). At the same time, sites 1, 5, and 6, belonging to the experimental zone, had more artificial sound components than other sites, reflecting indeed that the experimental area has more human interference than the buffer area. The results of this article are consistent with the fact that the experimental area allows more human activities, which once again proves the reliability of the VGGish model and clustering results, as well as the potential application value of ecological monitoring in a nature reserve. We hope to use this method to track and compare soundscapes for multiple years in YNNR and to monitor habitat degradation, habitat restoration, species abundance changes, and climate change effects.

Some mixed sounds were not wholly distinguished, such as the mixture of bird and insect sounds, the mixture of bird and rain, and the mixture of biophony and anthropophony. One possible reason for this is that different vocal groups are active simultaneously, and the other may be that the separation method needs to be improved. The mixed cluster suggests that we need to pay more attention to this in future research, such as studying the interactions and relationships between biological groups. In addition, it is also necessary to optimize cluster parameters, perform cross-validation, or adapt clustering algorithms without selecting the number of clusters in advance, *etc*., to improve the results. At the same time, the soundscape patterns may be influenced by the subjective assignment of clusters to soundscape components. Finally, since we currently cannot determine the exact proportion of different vocalization groups in mixed soundscape components, we need to optimize our method to solve this problem and enhance the automation of the whole workflow in the future.

## Conclusions

In this study, we extracted biological components from the soundscape using VGGish feature embeddings and unsupervised clustering, and we illustrated basic patterns of the bioacoustics community in a subtropical forest. The general acoustic features are powerful in their ability to identify broad soniferous animals/biophony, geophony and anthropophony from the soundscape, thereby helping to determine their spatial and temporal trends. Acoustic-based biodiversity assessments using this data-driven solution at a fine spatial scale may help in detecting acoustic hotspots for soundscape conservation.

## Supplemental Information

10.7717/peerj.16462/supp-1 Supplemental Information 1 Supplemental table and figures.

10.7717/peerj.16462/supp-2 Supplemental Information 2 Raw data and code.

10.7717/peerj.16462/supp-3 Supplemental Information 3 Representative spectrograms from cluster 0 to cluster 18. The spectrograms were computed using a Hann window, FFT = 512, window overlap of 50%, and frame size of 100%. The X-axis represents time, the Y-axis represents frequency. There are 10 audio segments for each cluster.

10.7717/peerj.16462/supp-4 Supplemental Information 4 Representative spectrograms from cluster 19 to cluster 37. The spectrograms were computed using a Hann window, FFT = 512, window overlap of 50%, and frame size of 100%. The X-axis represents time, the Y-axis represents frequency. There are 10 audio segments for each cluster.

10.7717/peerj.16462/supp-5 Supplemental Information 5 Representative spectrograms from cluster 38 to cluster 56. The spectrograms were computed using a Hann window, FFT = 512, window overlap of 50%, and frame size of 100%. The X-axis represents time, the Y-axis represents frequency. There are 10 audio segments for each cluster.

10.7717/peerj.16462/supp-6 Supplemental Information 6 Representative spectrograms from cluster 57 to cluster 75. The spectrograms were computed using a Hann window, FFT = 512, window overlap of 50%, and frame size of 100%. The X-axis represents time, the Y-axis represents frequency. There are 10 audio segments for each cluster.

10.7717/peerj.16462/supp-7 Supplemental Information 7 Representative spectrograms from cluster 76 to cluster 94. The spectrograms were computed using a Hann window, FFT = 512, window overlap of 50%, and frame size of 100%. The X-axis represents time, the Y-axis represents frequency. There are 10 audio segments for each cluster.

## Additional Information and Declarations

### Field Study Permissions

The following information was supplied relating to field study approvals (*i.e*., approving body and any reference numbers): The sound sampling was conducted under the permission of the forest managers of the Yaoluoping National Nature Reserve.

### Data Availability

The following information was supplied regarding data availability: The data and code are available in the Supplemental File. The Supplement_information_S3 is the spectrogram (in five parts: Supplement_information_S3_spec95_01,02,03,04,05). The audio sample is available at Fanglin Collection: https://ecosound-web.de/ecosound_web/collection/show/45. The audio can also be found by going to Projects ->Open collections ->Yaoluoping National Nature Reserve. The audio sample is also available at FigShare: Wang, Mei (2023). audio. figshare. Dataset. https://doi.org/10.6084/m9.figshare.22584775.v1.
