# Supplementary material for: VGGish-based detection of biological sound components and their spatio-temporal variations in a subtropical forest in eastern China
Source: PeerJ. 2023 Nov 15;11:e16462. doi: 10.7717/peerj.16462 (PMC10656901; doi:10.7717/peerj.16462)
Supplement: Supplemental Information 1 [file peerj-11-16462-s001.docx]

**VGGish-based detection of biological sound components and their spatio-temporal variations in a subtropical forest in eastern China：**

**Supplementary Information Appendix**

Table S1. The 95 acoustic clusters grouped into classes according to their dominant acoustic sources

| Class Label | Clusters assigned to class label |
| --- | --- |
| mainly bird | 0, 2, 12, 13, 20, 26, 43, 44, 47, 48, 54, 60, 64, 81 |
| mainly insect | 9, 11, 15, 19, 21, 28, 29, 30, 32, 36, 37, 39, 41, 42, 50, 53, 55, 57, 63, 65, 72, 73, 83, 84, 85, 86, 87, 91, 92, 94 |
| mainly rain | 4, 14, 23, 35, 45, 49, 62, 67, 69, 71, 74, 82 |
| no obvious biophony | 5, 6, 8, 10, 16, 17, 18, 22, 24, 27, 33, 40, 56, 59, 61, 70, 75, 76, 77, 88, 89, 90 |
| bird and insect | 3, 34, 38, 46, 51, 52, 58, 68, 78, 79, 80, 93 |
| bird and rain | 25, 66 |
| biophony and anthropophony | 1, 7, 31 |

Table S2. Percentage of different acoustic sources in each site

| Class Label | site1 | site2 | site3 | site4 | site5 | site6 |
| --- | --- | --- | --- | --- | --- | --- |
| mainly bird | 25.10% | 9.38% | 25.62% | 22.02% | 13.53% | 15.93% |
| mainly insect | 17.80% | 24.82% | 24.59% | 15.96% | 45.13% | 45.20% |
| mainly rain | 10.25% | 8.14% | 12.38% | 11.11% | 9.96% | 8.62% |
| no obvious biophony | 28.03% | 37.66% | 26.90% | 27.36% | 15.82% | 17.87% |
| bird and insect | 12.18% | 19.82% | 9.73% | 20.88% | 8.53% | 10.38% |
| bird and rain | 5.16% | 0.03% | 0.11% | 1.74% | 0.02% | 0.01% |
| biophony and anthropophony | 1.49% | 0.16% | 0.67% | 0.93% | 7.02% | 1.99% |


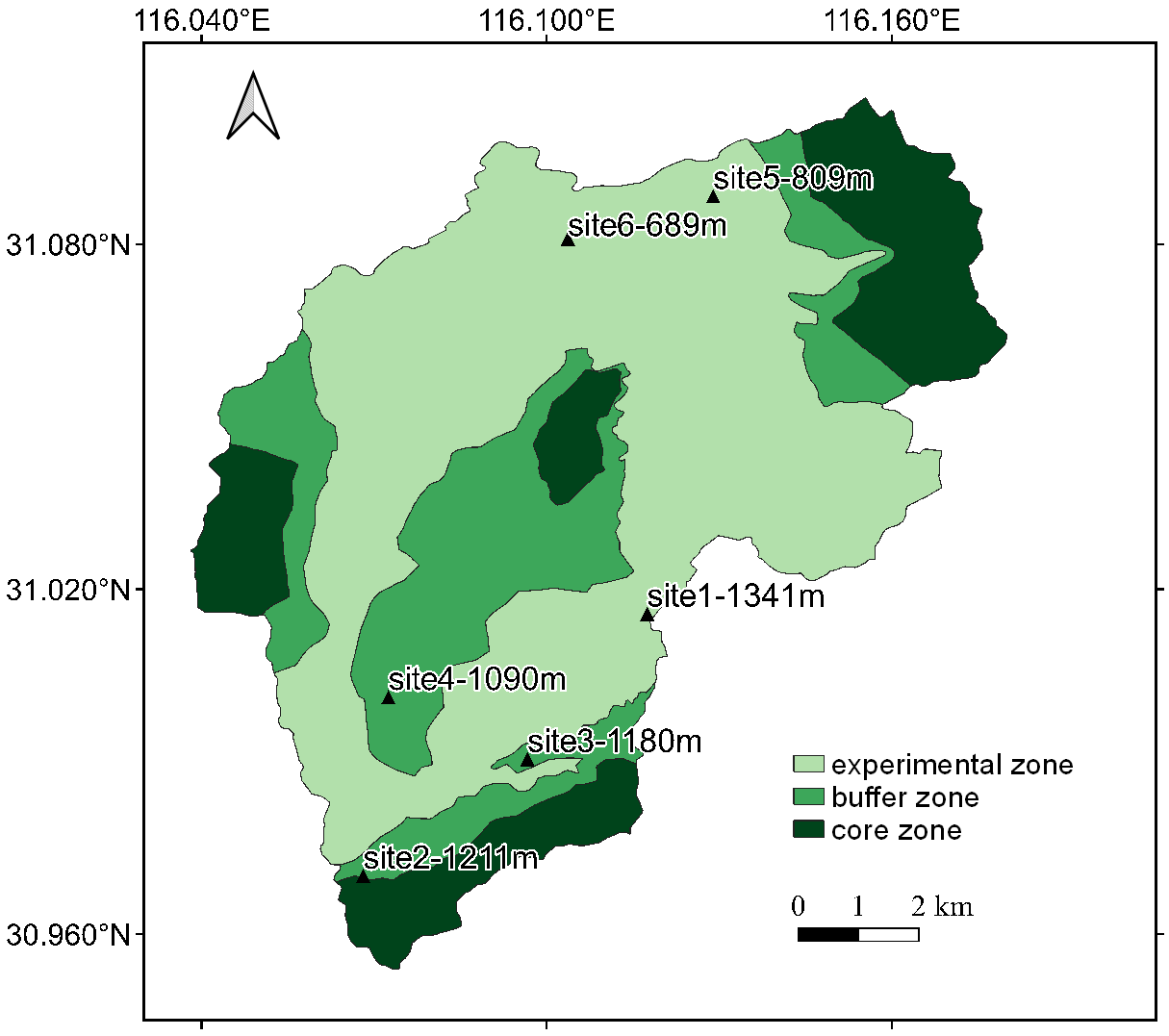


Figure S1. The map of the Yaoluoping National Nature Reserve (116°02′ E -116°11′ E, 30°57′ N -31°06′ N) and the distribution of the soundscape sample sites


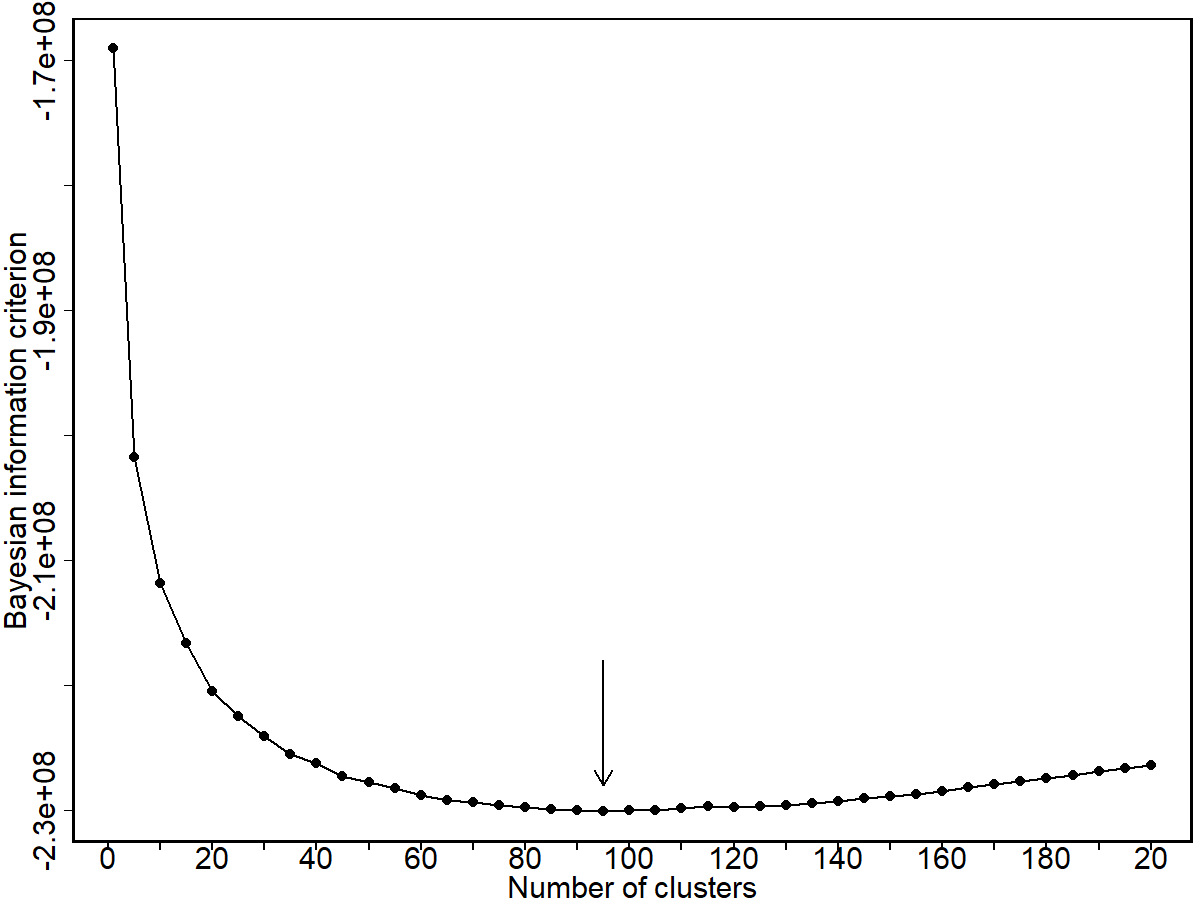


Figure S2. Bayesian information criterion for different clusters. There is a minimum value at 95.


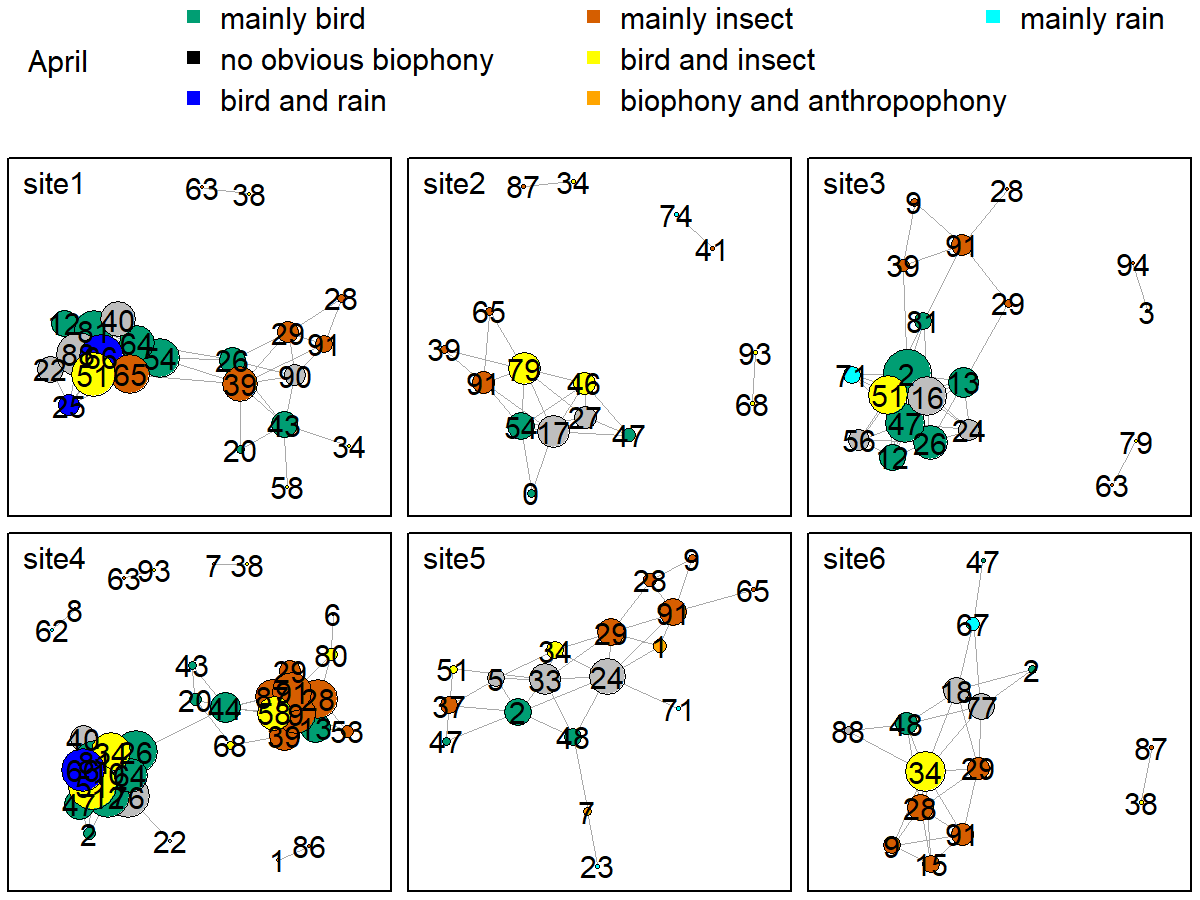


Figure S3. Network map of different clusters at six sites in April. The nodes represent each cluster, the edges represent cluster association, and a large dot size represents a large degree. The different clusters belong to seven soundscape components displayed in different colors.


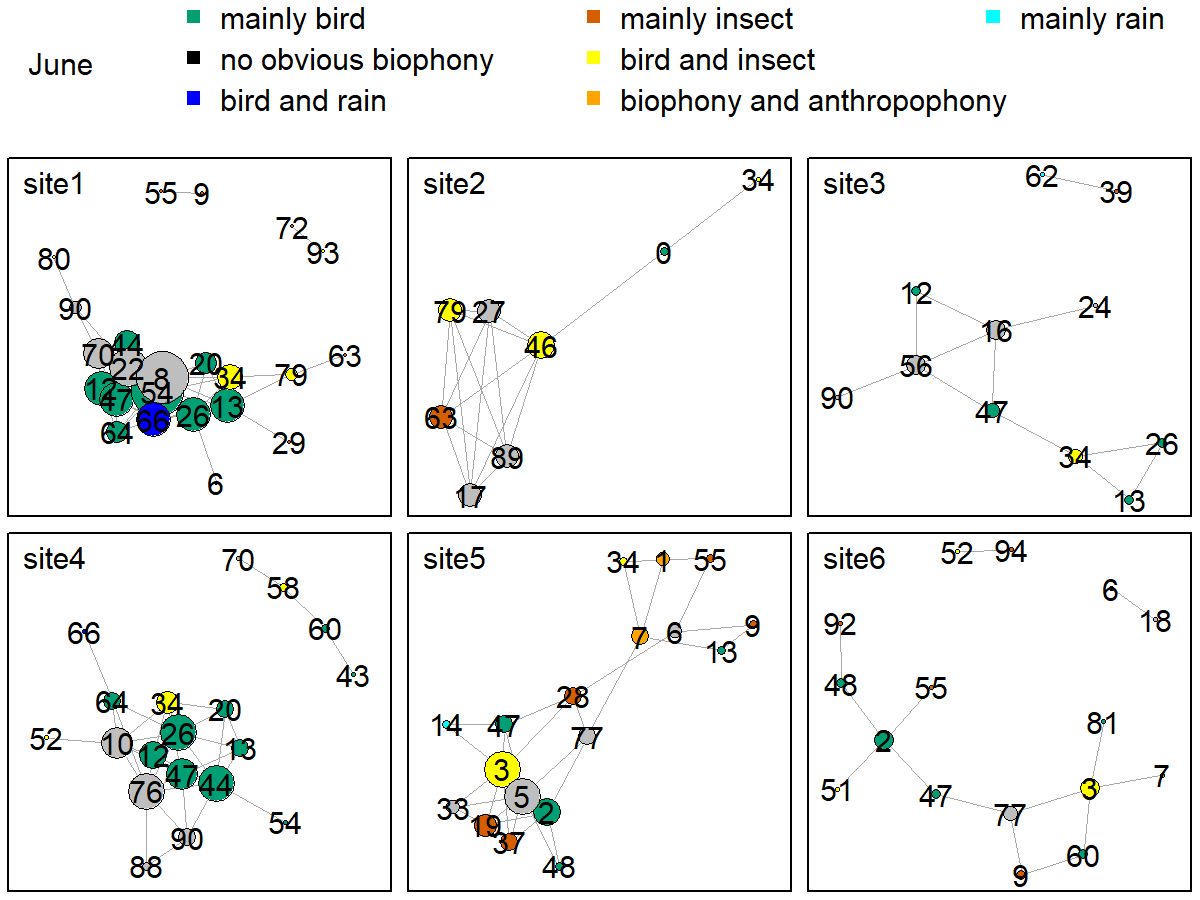


Figure S4. Network map of different clusters at six sites in June. The nodes represent each cluster, the edges represent cluster association, and a large dot size represents a large degree. The different clusters belong to seven soundscape components displayed in different colors.


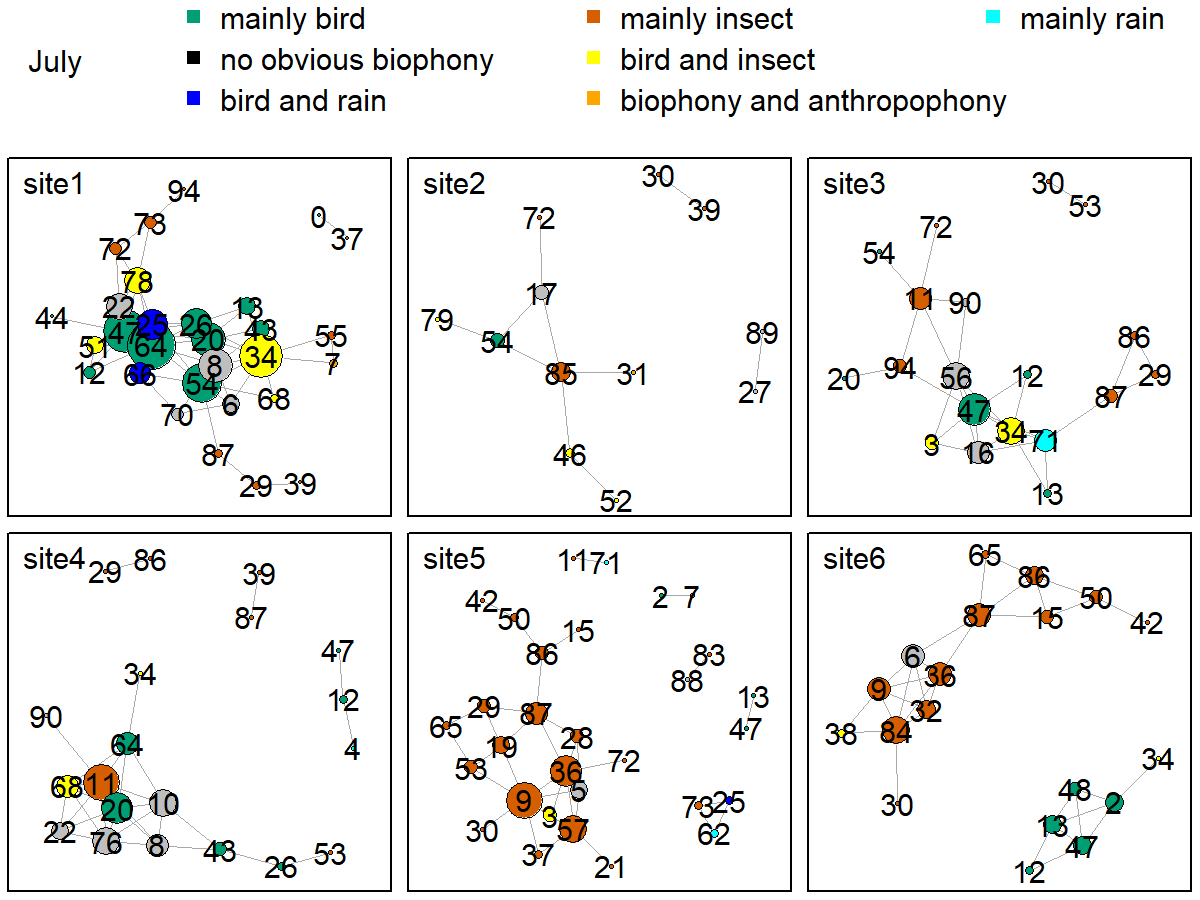


Figure S5. Network map of different clusters at six sites in July. The nodes represent each cluster, the edges represent cluster association, and a large dot size represents a large degree. The different clusters belong to seven soundscape components displayed in different colors.


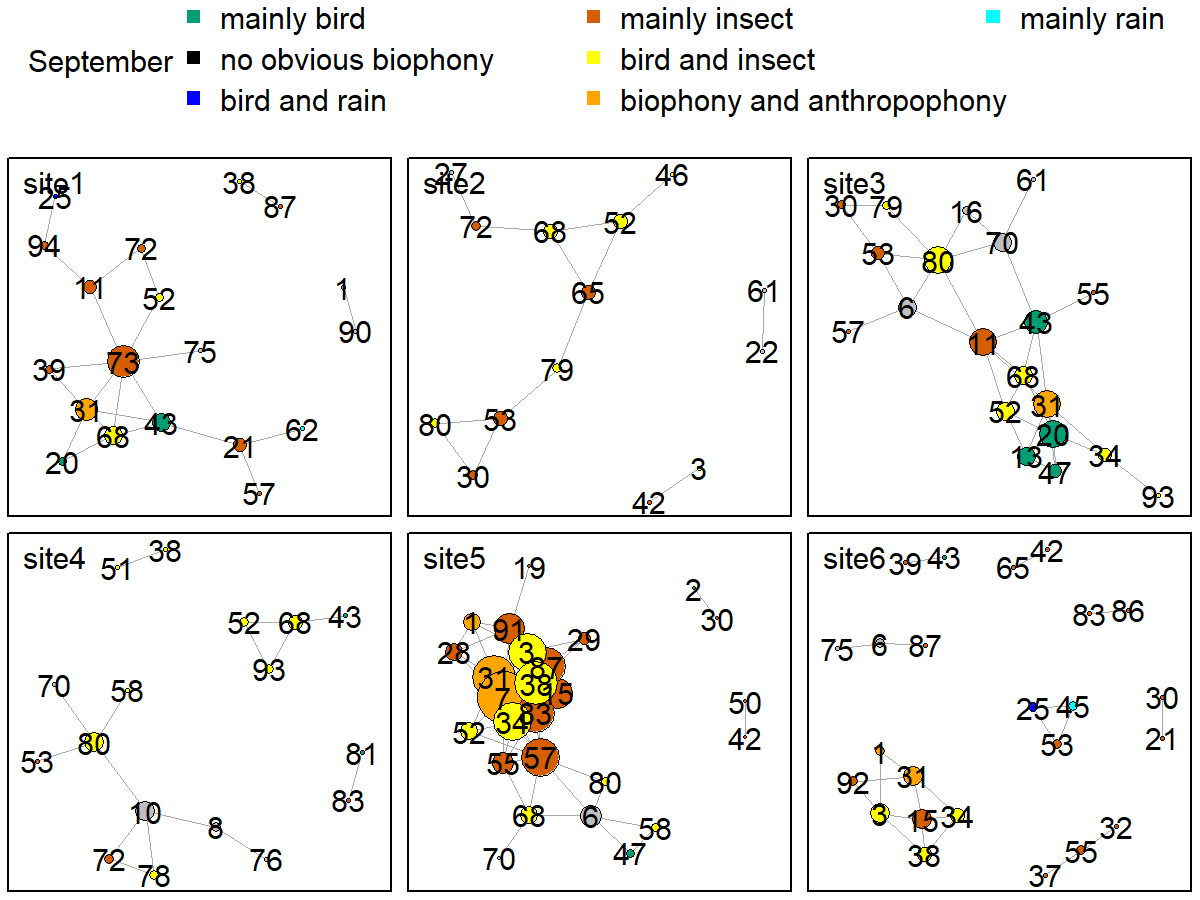


Figure S6. Network map of different clusters at six sites in September. The nodes represent each cluster, the edges represent cluster association, and a large dot size represents a large degree. The different clusters belong to seven soundscape components displayed in different colors.


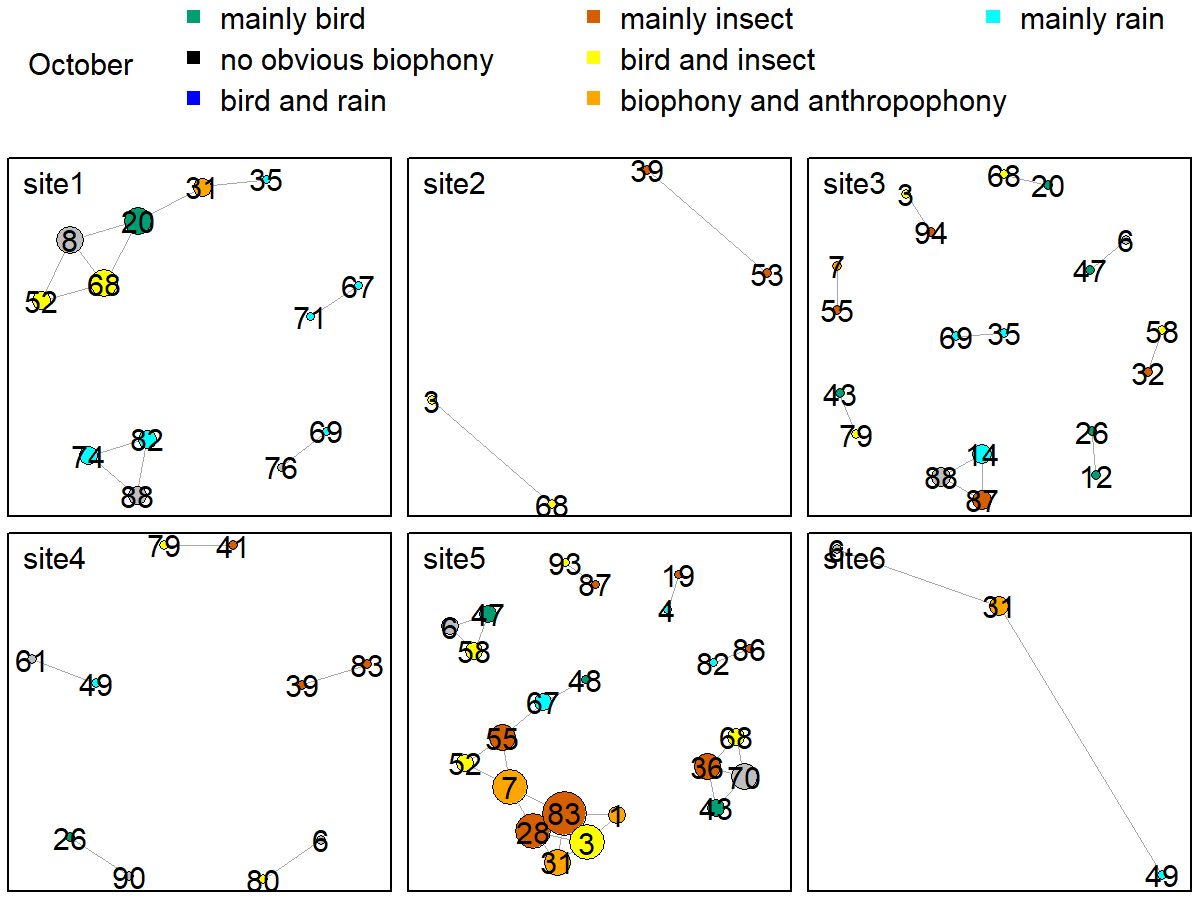


Figure S7. Network map of different clusters at six sites in October. The nodes represent each cluster, the edges represent cluster association, and a large dot size represents a large degree. The different clusters belong to seven soundscape components displayed in different colors.
