# Supplementary figures and images for: VGGish-based detection of biological sound components and their spatio-temporal variations in a subtropical forest in eastern China (part 1 of 2)
Source: PeerJ. 2023 Nov 15;11:e16462. doi: 10.7717/peerj.16462 (PMC10656901; doi:10.7717/peerj.16462)

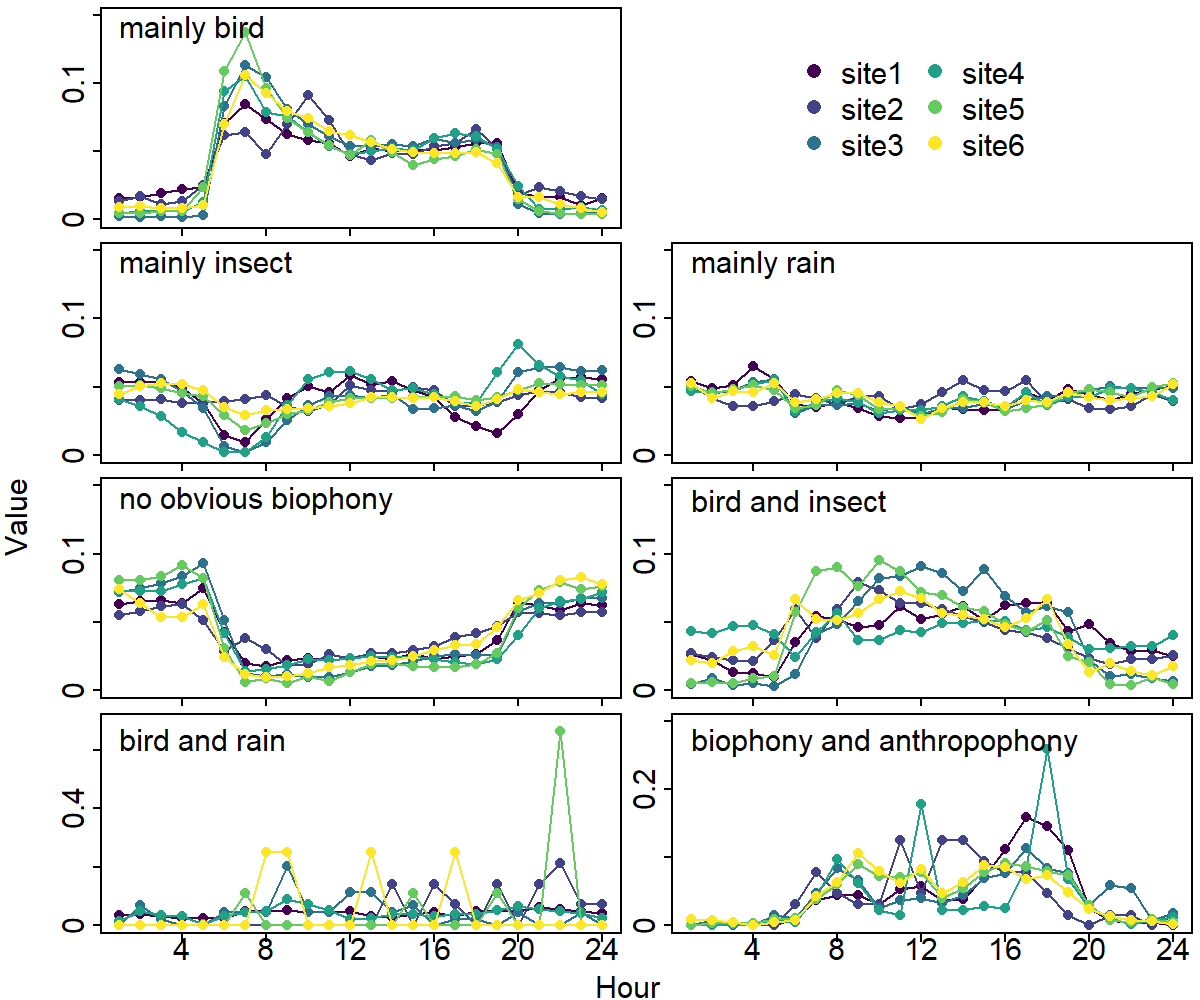

Supplement: Supplemental Information 2 [file peerj-11-16462-s002.zip › Supplemental_Information_S2_data/drawpicture/output/hour.png]

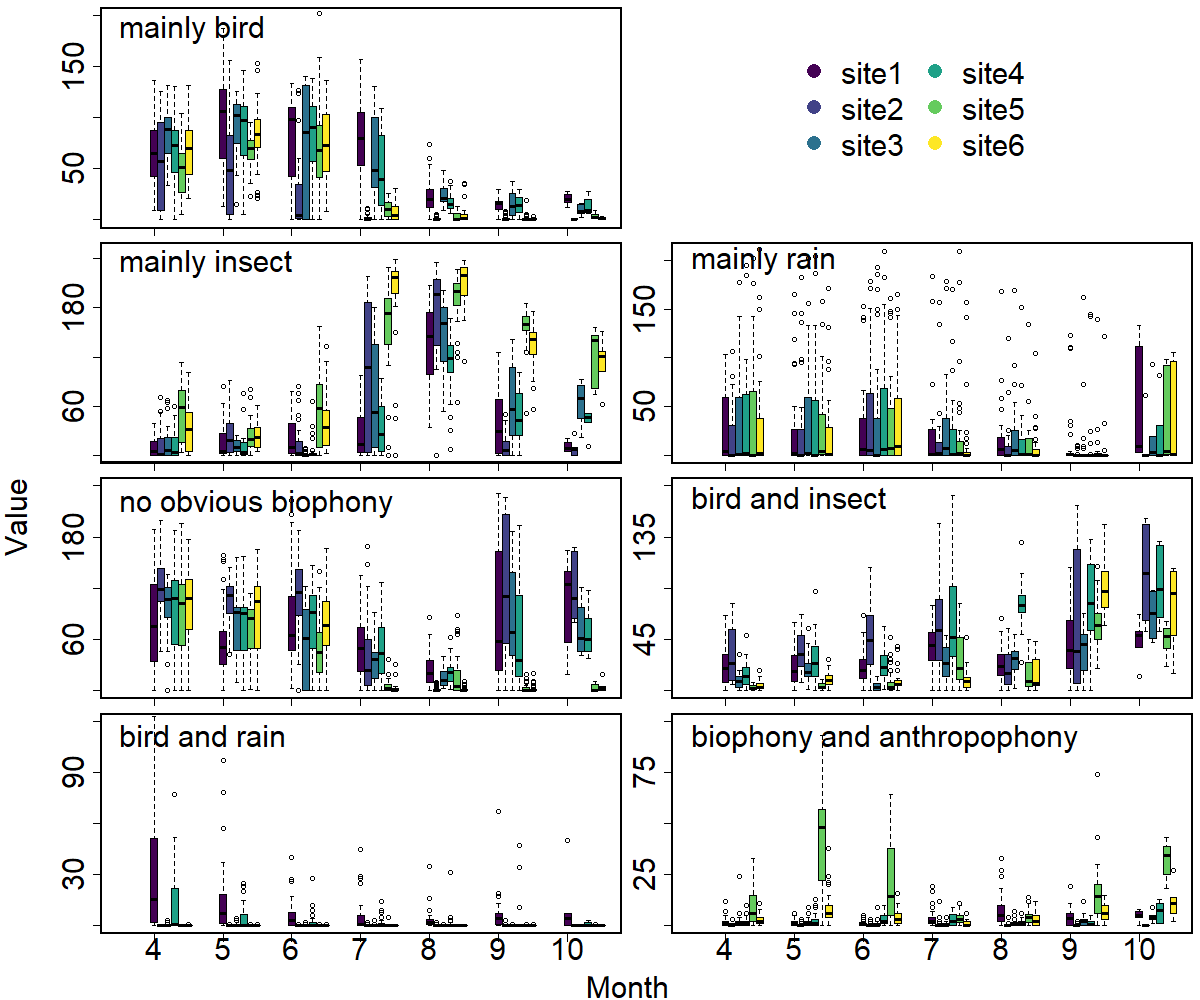

Supplement: Supplemental Information 2 [file peerj-11-16462-s002.zip › Supplemental_Information_S2_data/drawpicture/output/month.png]

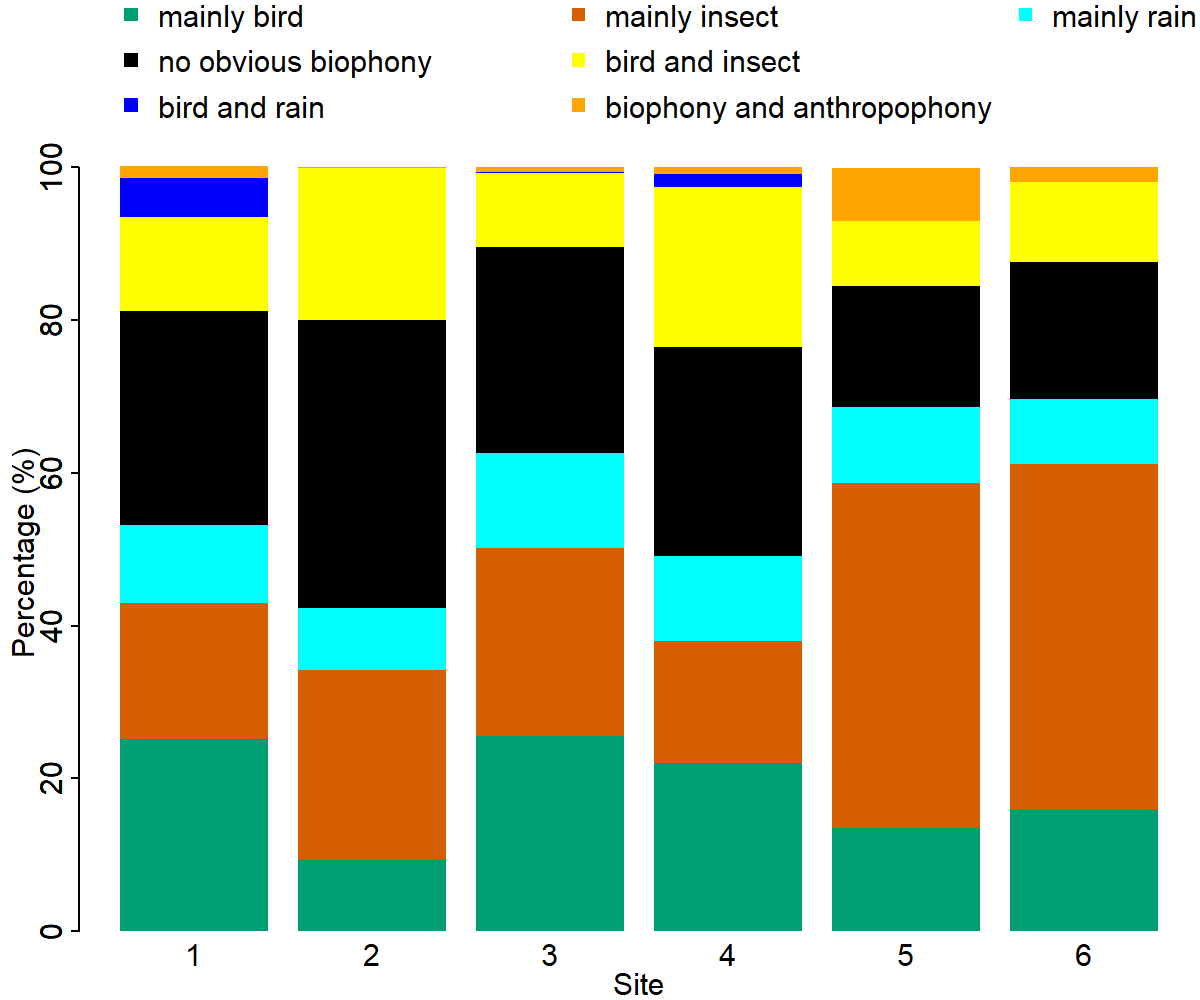

Supplement: Supplemental Information 2 [file peerj-11-16462-s002.zip › Supplemental_Information_S2_data/drawpicture/output/percent.png]

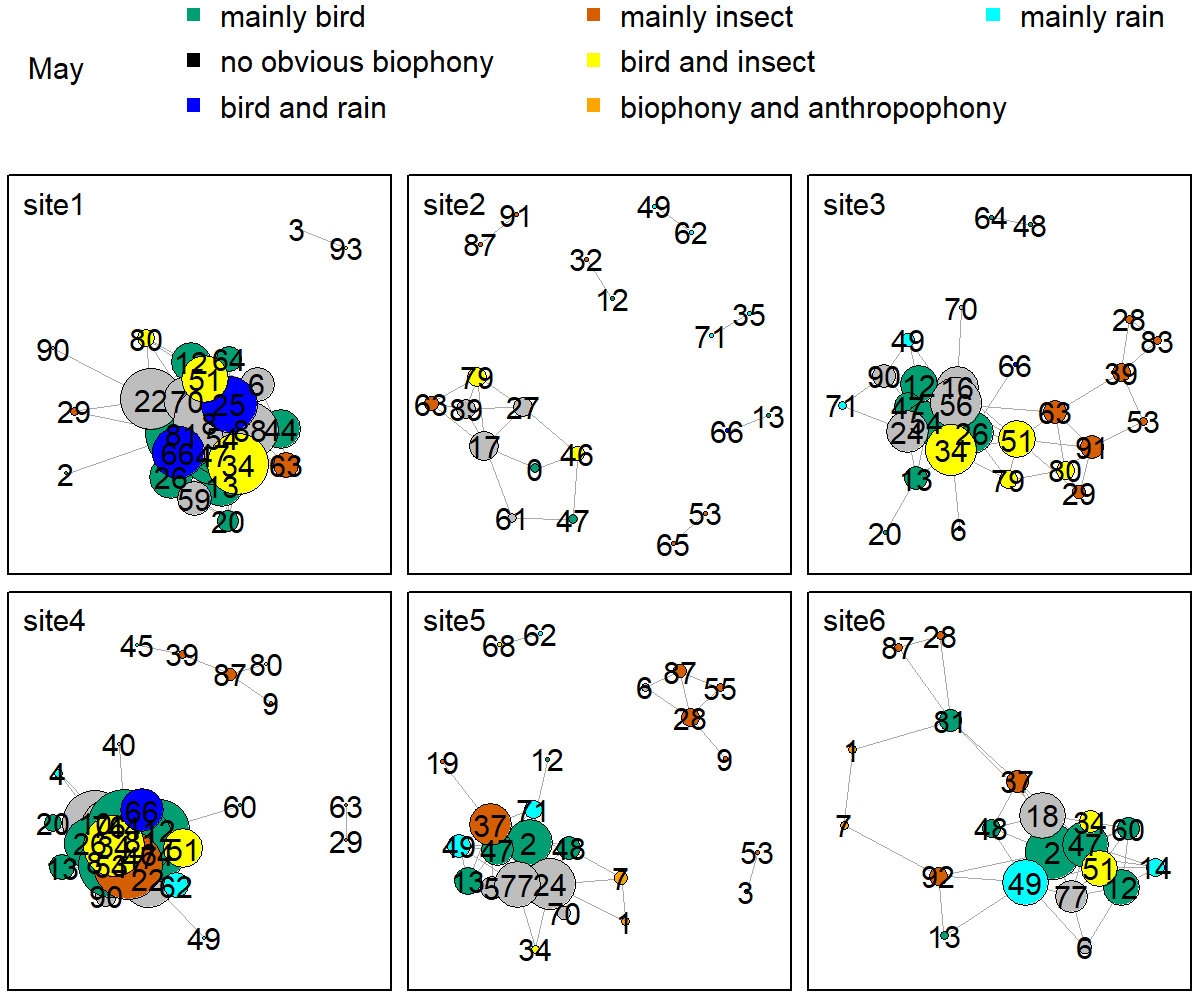

Supplement: Supplemental Information 2 [file peerj-11-16462-s002.zip › Supplemental_Information_S2_data/drawpicture/output/socialnetwork-month5.png]

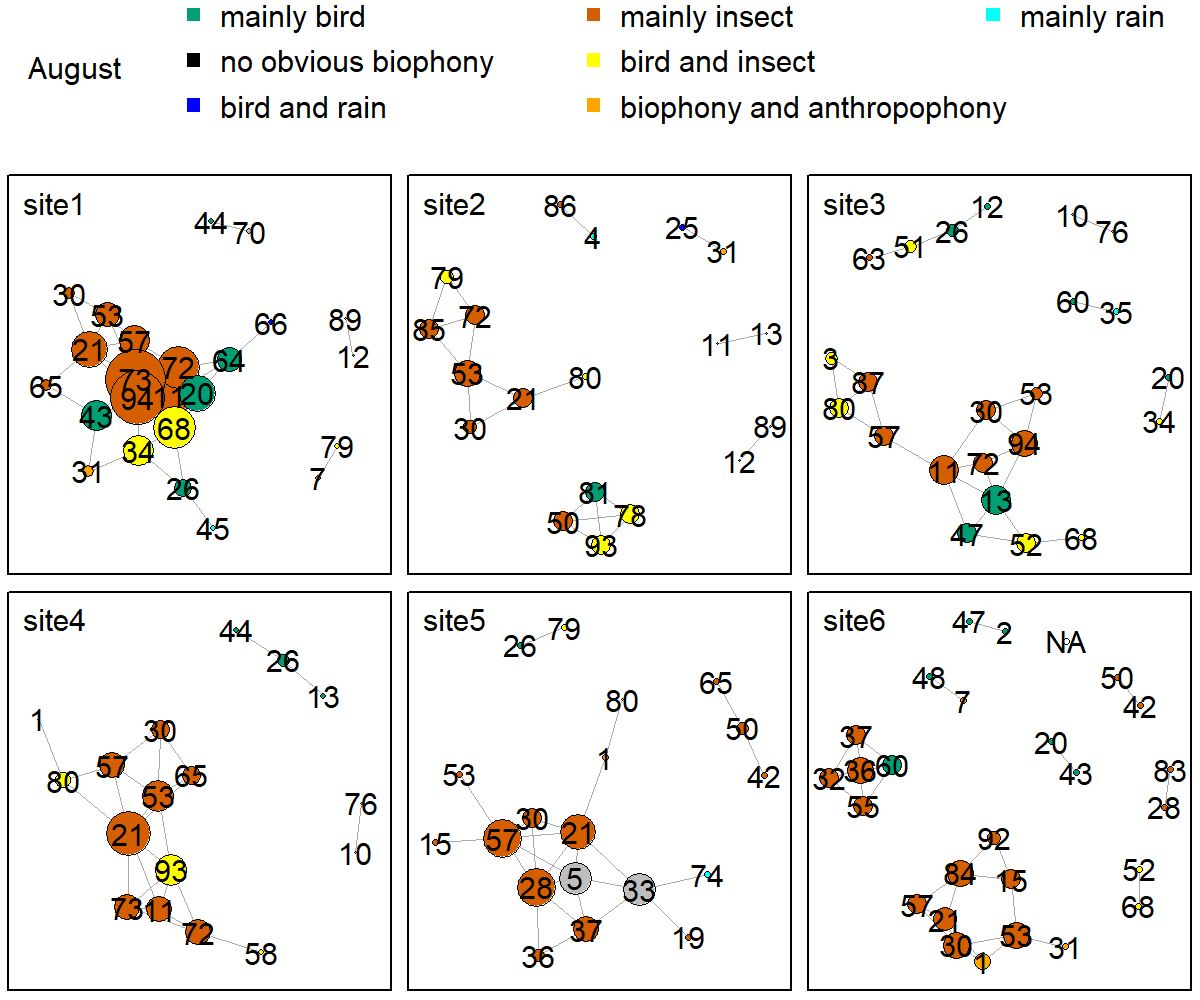

Supplement: Supplemental Information 2 [file peerj-11-16462-s002.zip › Supplemental_Information_S2_data/drawpicture/output/socialnetwork-month8.png]

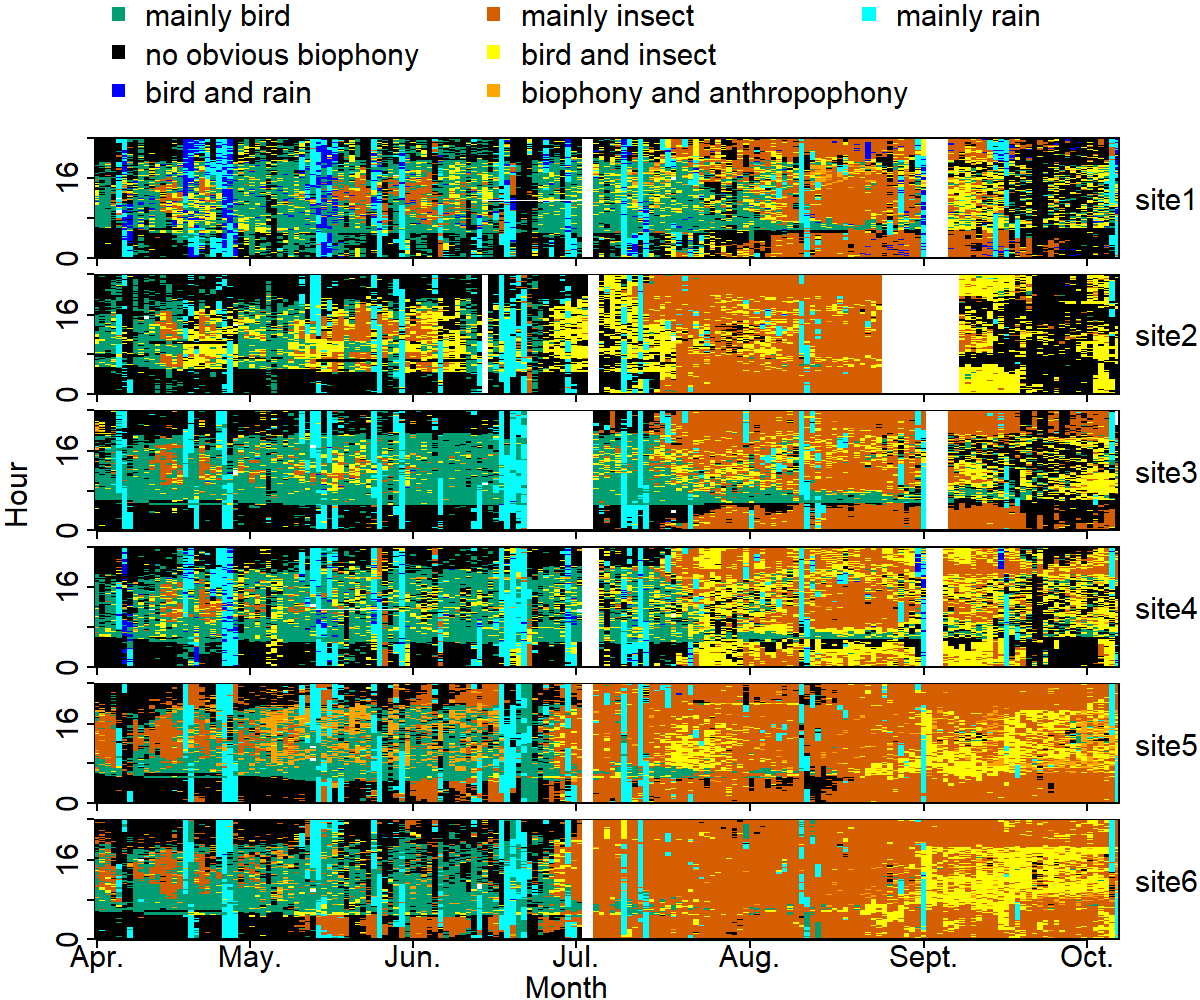

Supplement: Supplemental Information 2 [file peerj-11-16462-s002.zip › Supplemental_Information_S2_data/drawpicture/output/visual-soundscape.png]

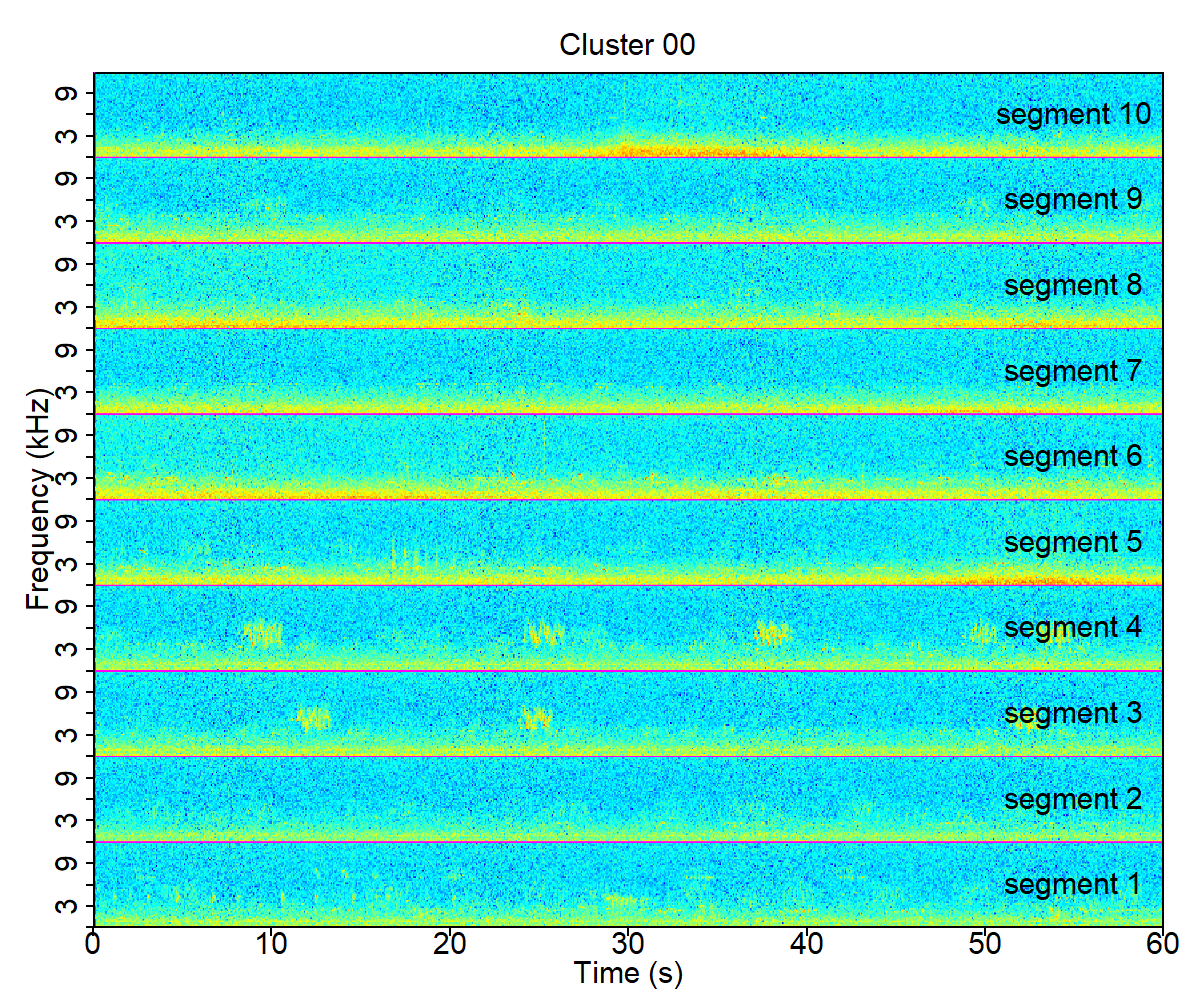

Supplement: Supplemental Information 3 — The spectrograms were computed using a Hann window, FFT = 512, window overlap of 50%, and frame size of 100%. The X-axis represents time, the Y-axis represents frequency. There are 10 audio segments for each cluster. [file peerj-11-16462-s003.zip › Supplemental_Information_S3_spec95_01/Cluster 00.png]

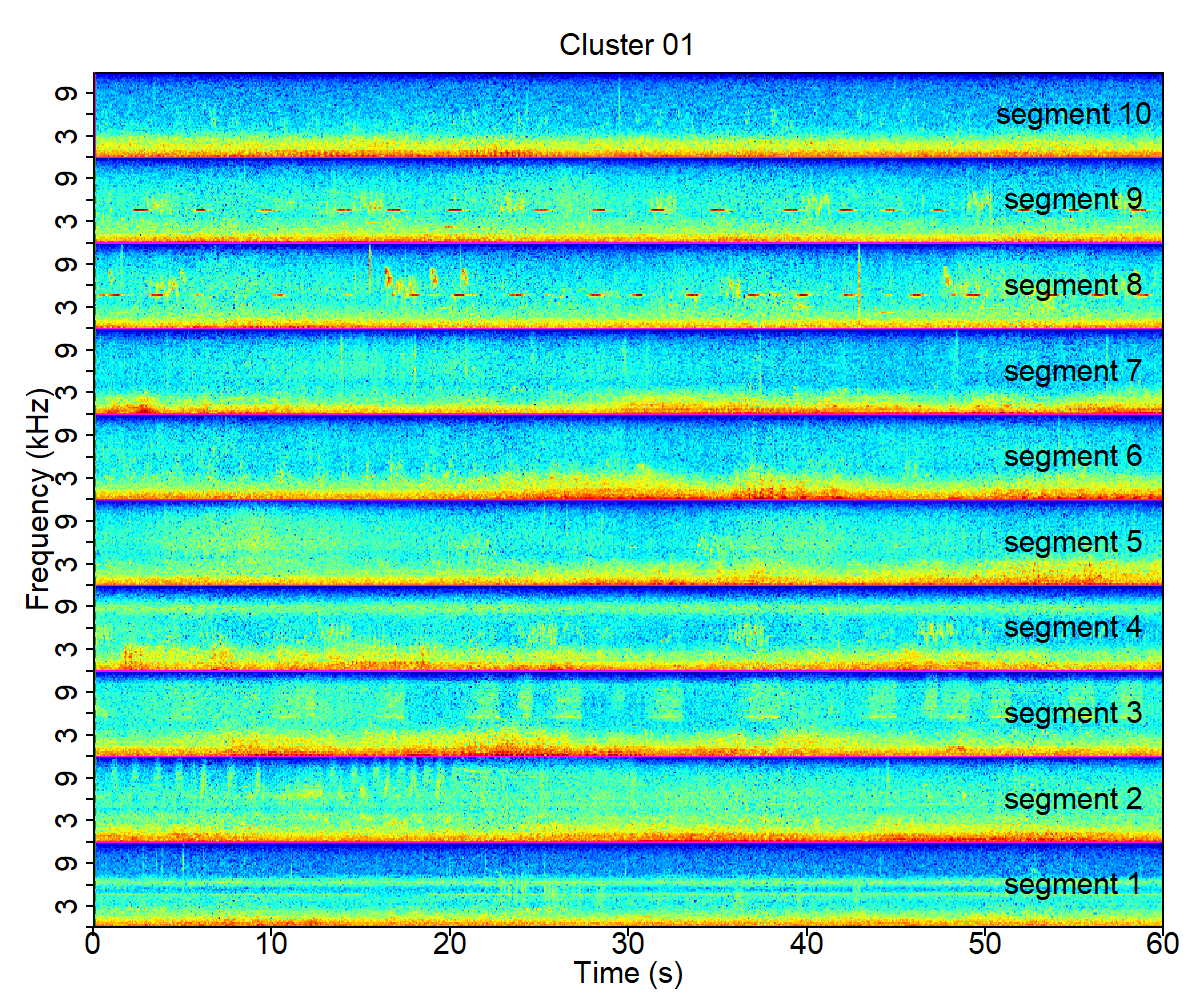

Supplement: Supplemental Information 3 — The spectrograms were computed using a Hann window, FFT = 512, window overlap of 50%, and frame size of 100%. The X-axis represents time, the Y-axis represents frequency. There are 10 audio segments for each cluster. [file peerj-11-16462-s003.zip › Supplemental_Information_S3_spec95_01/Cluster 01.png]

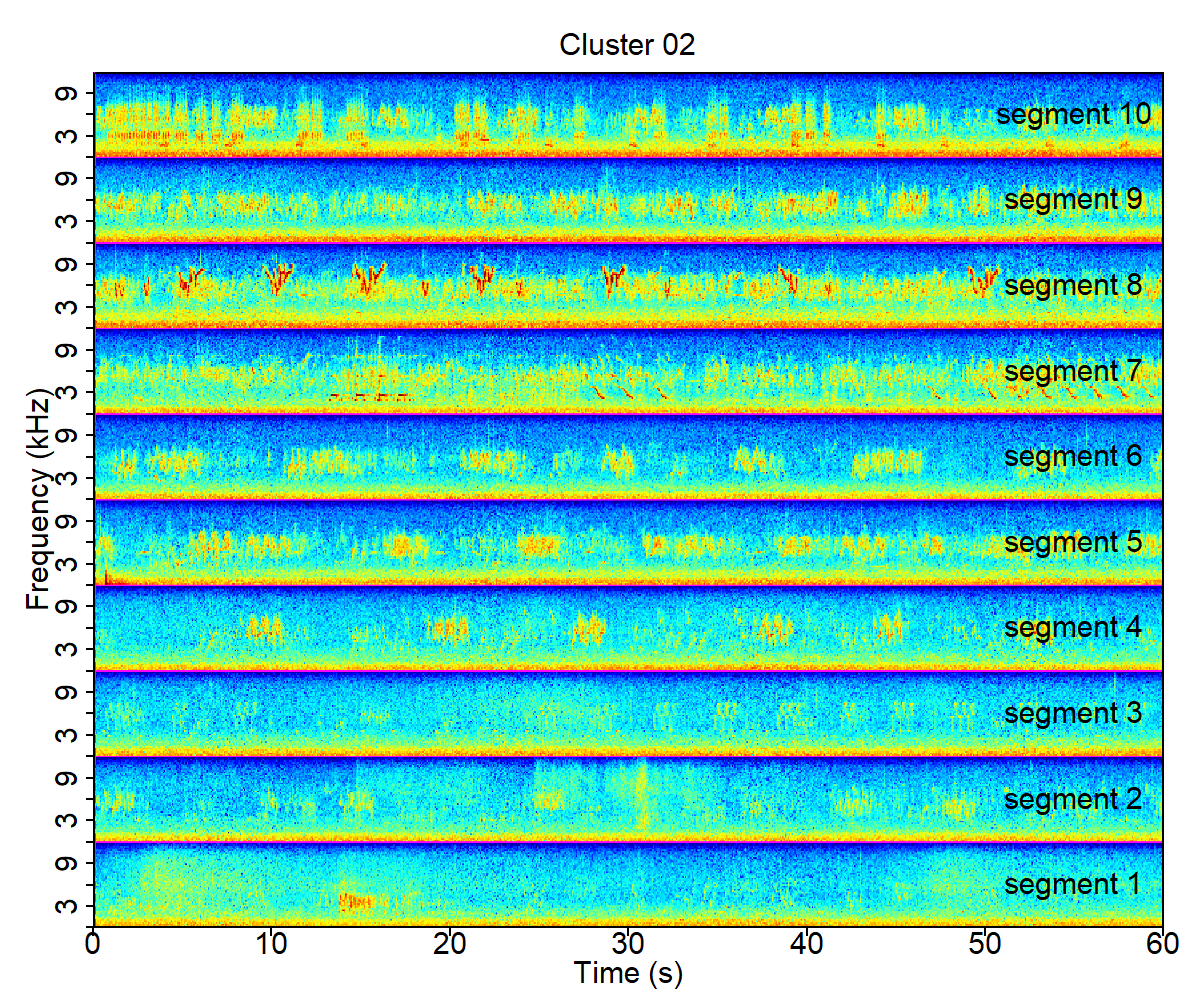

Supplement: Supplemental Information 3 — The spectrograms were computed using a Hann window, FFT = 512, window overlap of 50%, and frame size of 100%. The X-axis represents time, the Y-axis represents frequency. There are 10 audio segments for each cluster. [file peerj-11-16462-s003.zip › Supplemental_Information_S3_spec95_01/Cluster 02.png]

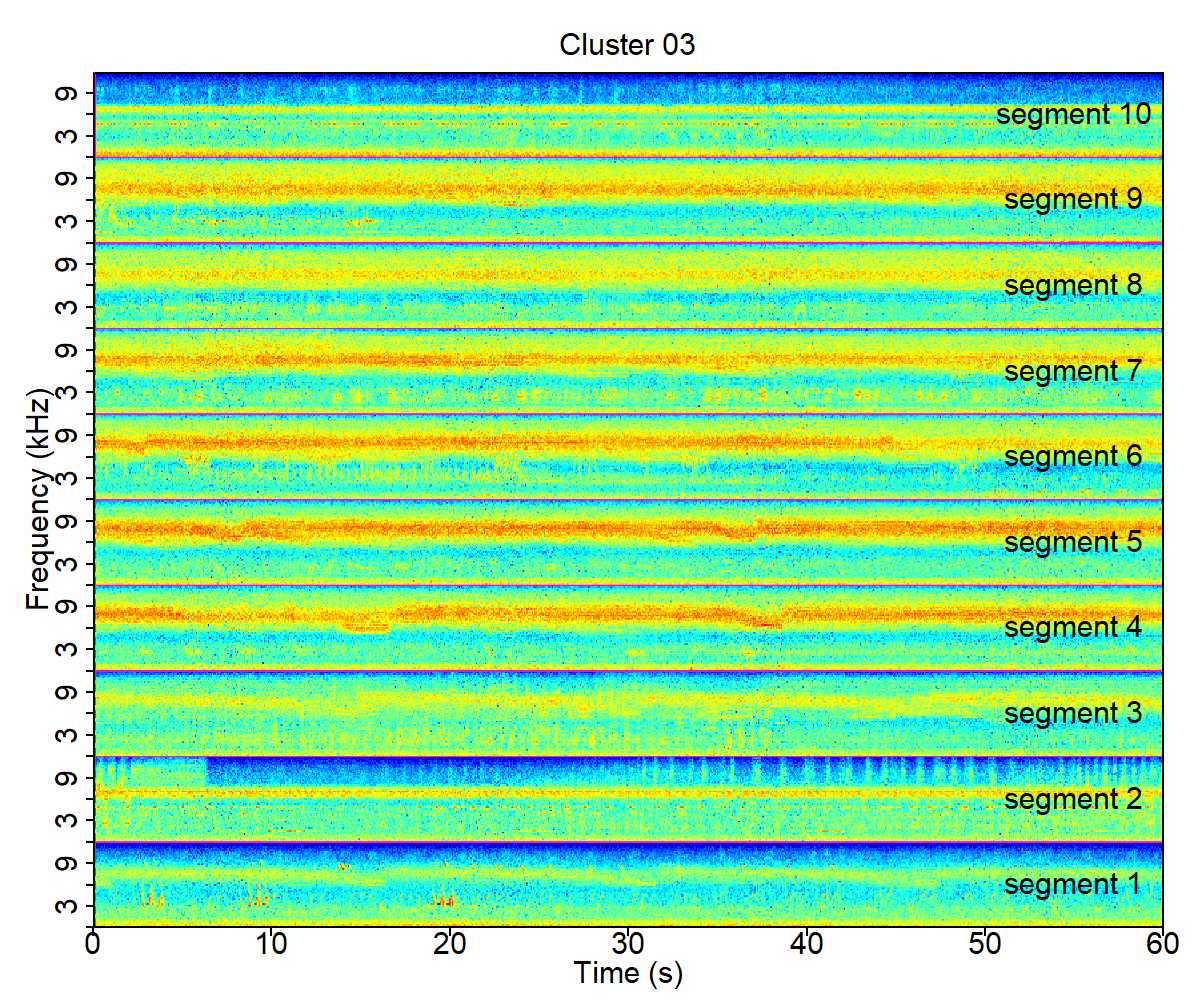

Supplement: Supplemental Information 3 — The spectrograms were computed using a Hann window, FFT = 512, window overlap of 50%, and frame size of 100%. The X-axis represents time, the Y-axis represents frequency. There are 10 audio segments for each cluster. [file peerj-11-16462-s003.zip › Supplemental_Information_S3_spec95_01/Cluster 03.png]

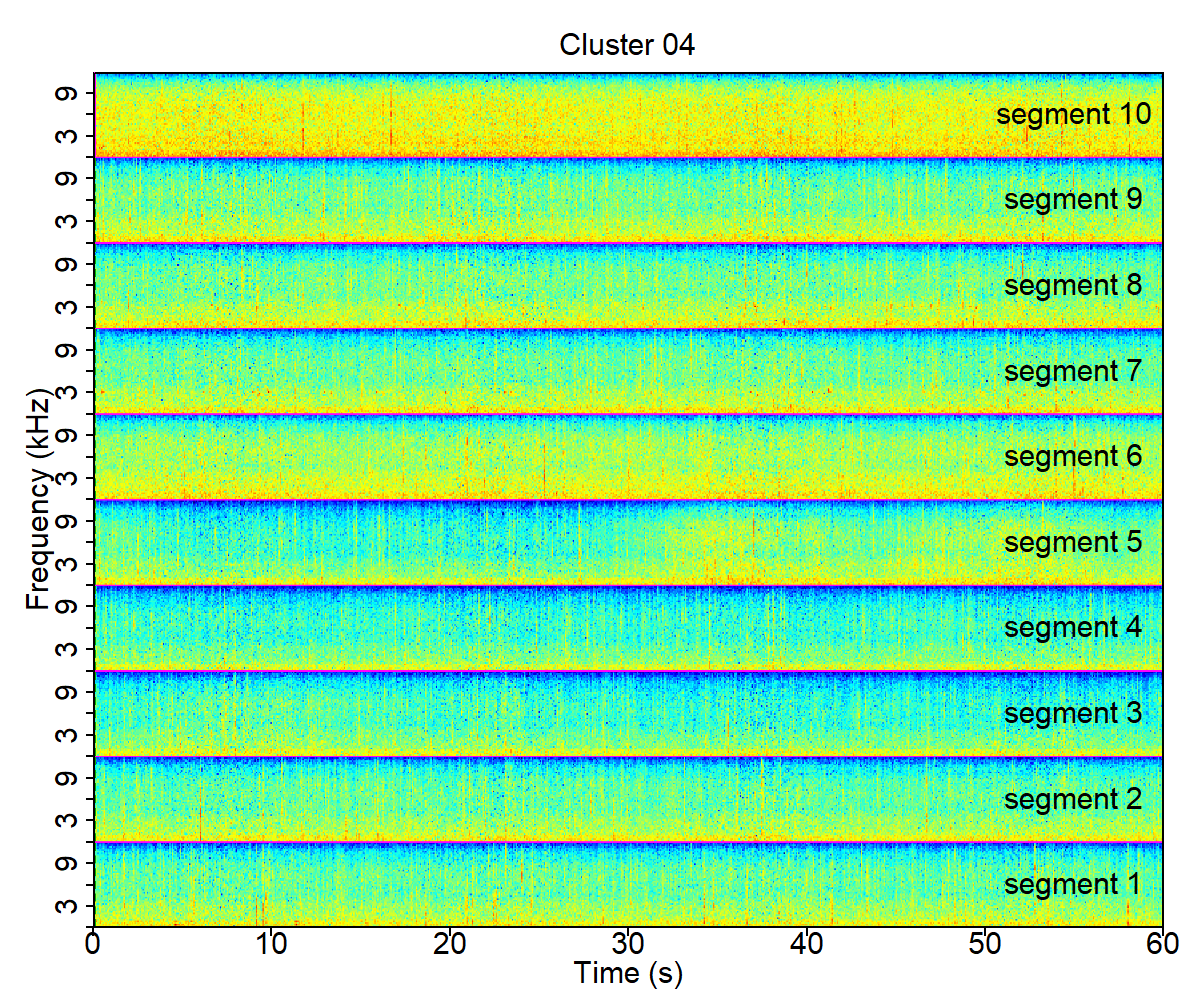

Supplement: Supplemental Information 3 — The spectrograms were computed using a Hann window, FFT = 512, window overlap of 50%, and frame size of 100%. The X-axis represents time, the Y-axis represents frequency. There are 10 audio segments for each cluster. [file peerj-11-16462-s003.zip › Supplemental_Information_S3_spec95_01/Cluster 04.png]

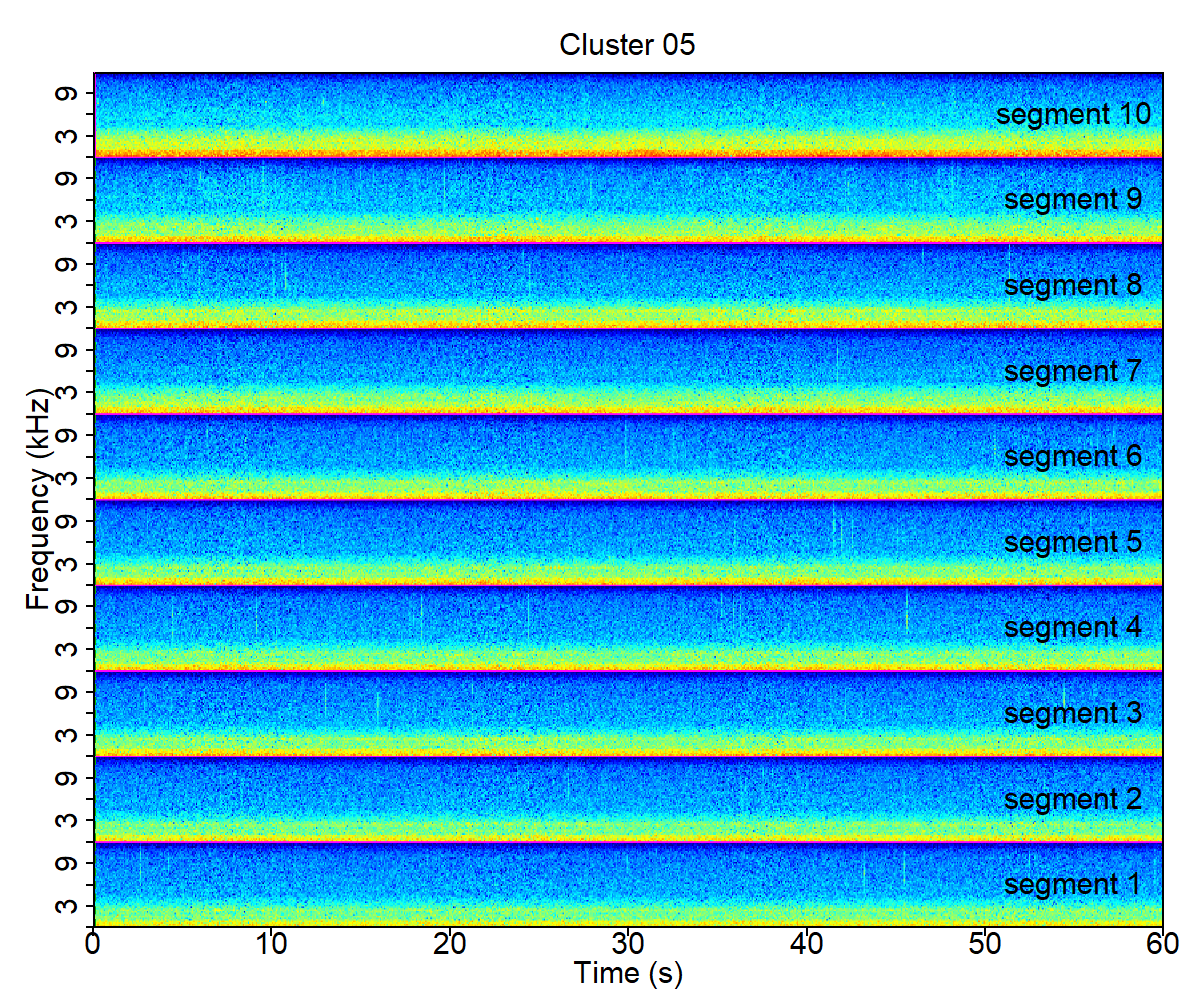

Supplement: Supplemental Information 3 — The spectrograms were computed using a Hann window, FFT = 512, window overlap of 50%, and frame size of 100%. The X-axis represents time, the Y-axis represents frequency. There are 10 audio segments for each cluster. [file peerj-11-16462-s003.zip › Supplemental_Information_S3_spec95_01/Cluster 05.png]

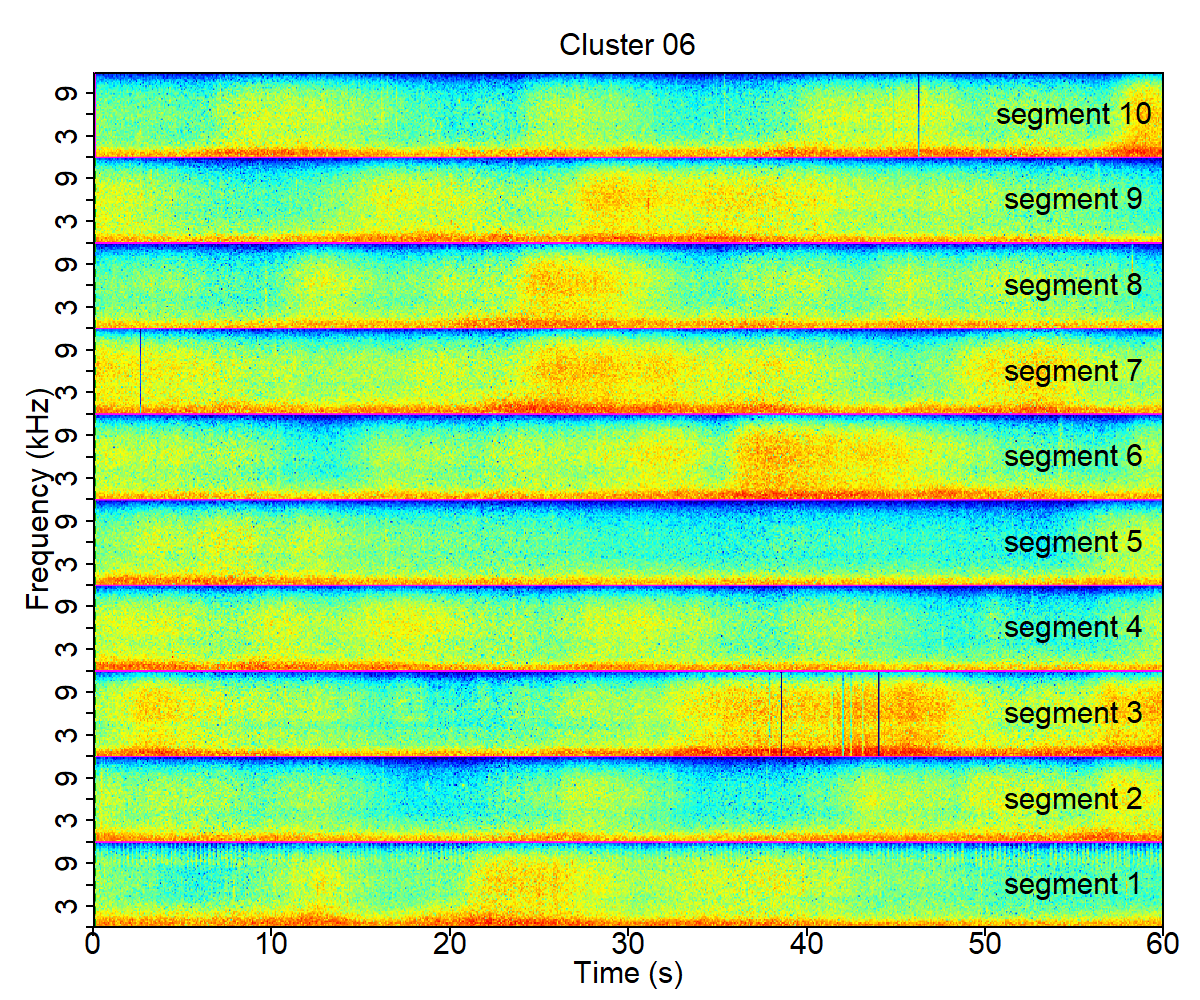

Supplement: Supplemental Information 3 — The spectrograms were computed using a Hann window, FFT = 512, window overlap of 50%, and frame size of 100%. The X-axis represents time, the Y-axis represents frequency. There are 10 audio segments for each cluster. [file peerj-11-16462-s003.zip › Supplemental_Information_S3_spec95_01/Cluster 06.png]

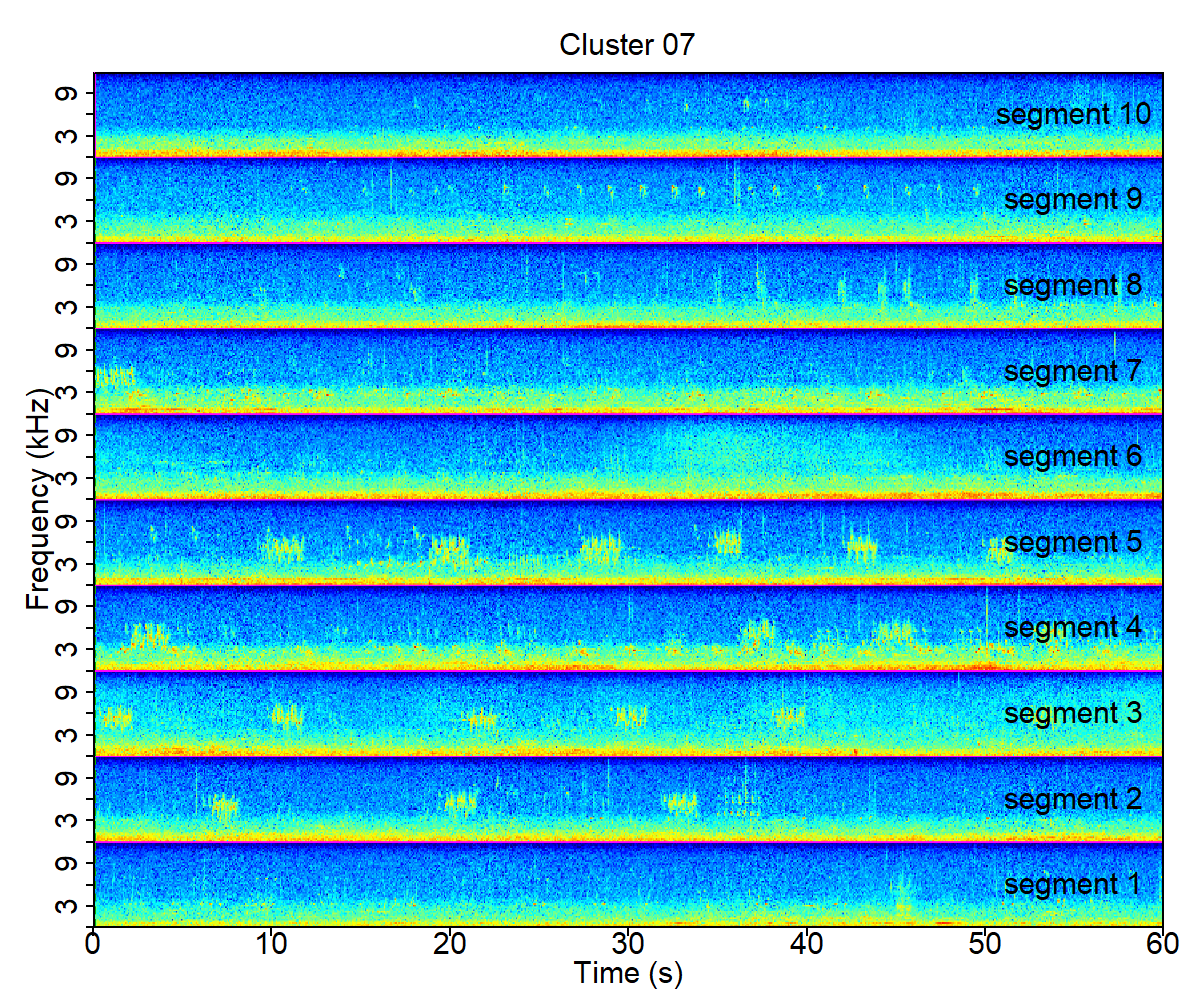

Supplement: Supplemental Information 3 — The spectrograms were computed using a Hann window, FFT = 512, window overlap of 50%, and frame size of 100%. The X-axis represents time, the Y-axis represents frequency. There are 10 audio segments for each cluster. [file peerj-11-16462-s003.zip › Supplemental_Information_S3_spec95_01/Cluster 07.png]

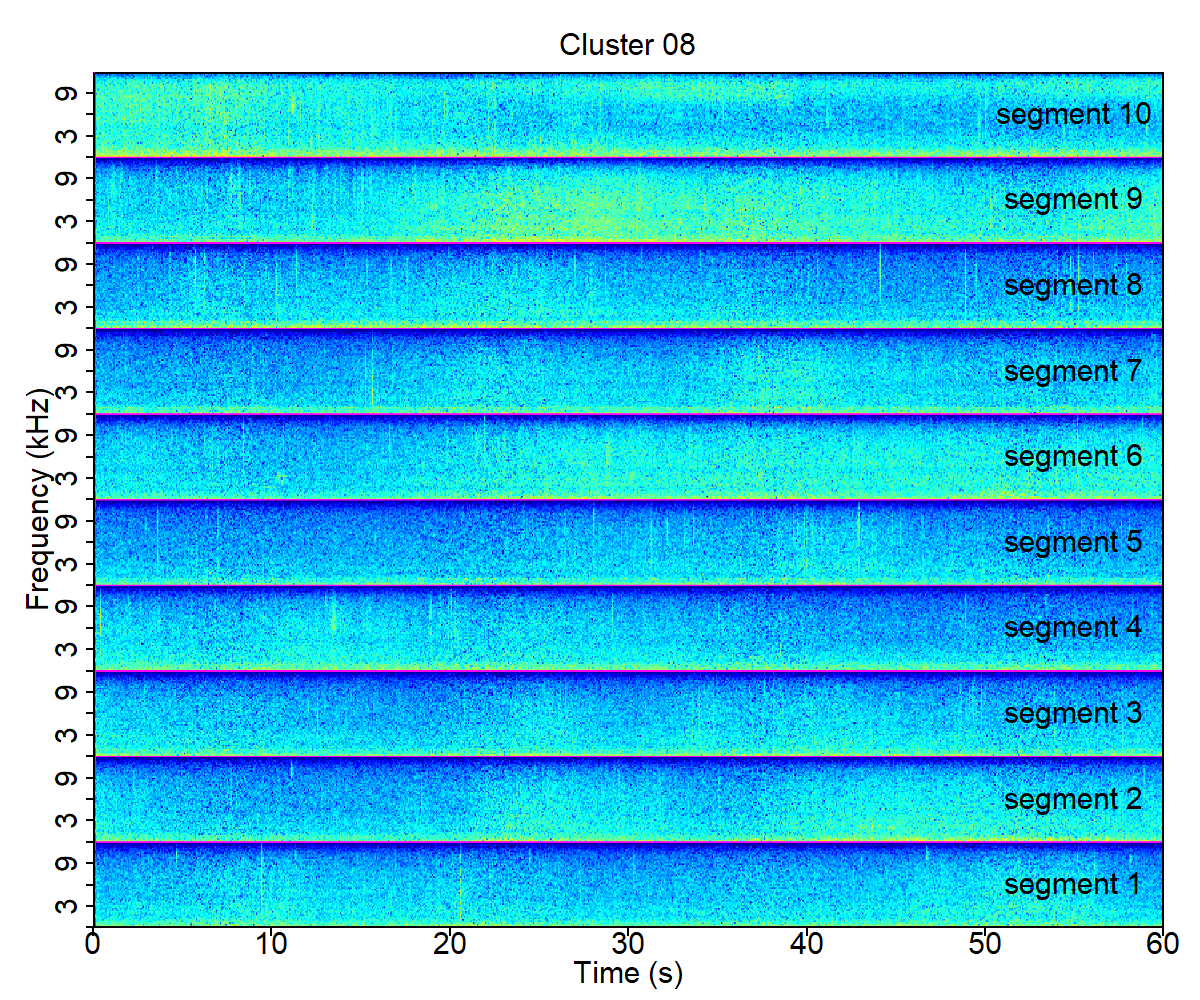

Supplement: Supplemental Information 3 — The spectrograms were computed using a Hann window, FFT = 512, window overlap of 50%, and frame size of 100%. The X-axis represents time, the Y-axis represents frequency. There are 10 audio segments for each cluster. [file peerj-11-16462-s003.zip › Supplemental_Information_S3_spec95_01/Cluster 08.png]

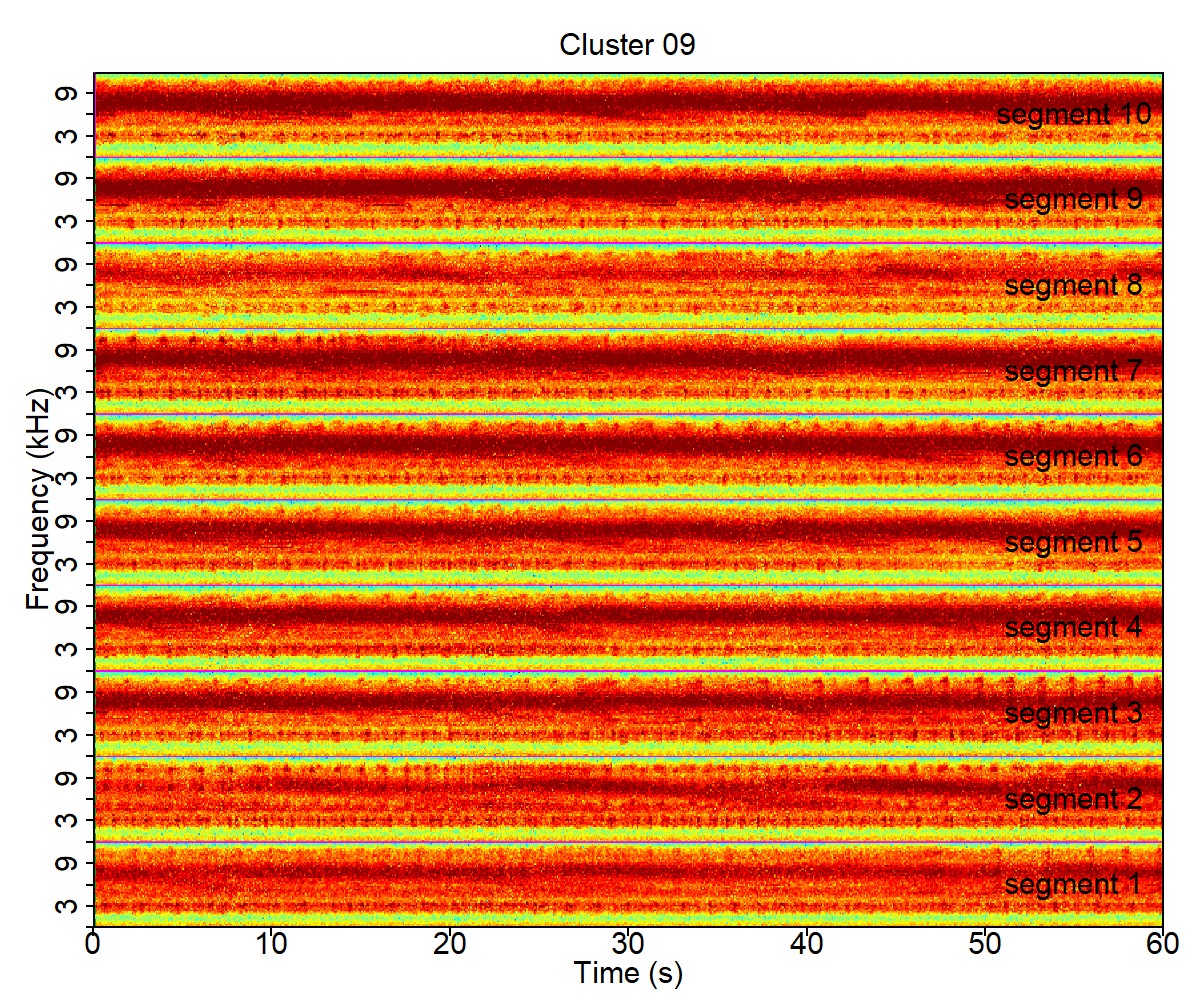

Supplement: Supplemental Information 3 — The spectrograms were computed using a Hann window, FFT = 512, window overlap of 50%, and frame size of 100%. The X-axis represents time, the Y-axis represents frequency. There are 10 audio segments for each cluster. [file peerj-11-16462-s003.zip › Supplemental_Information_S3_spec95_01/Cluster 09.png]

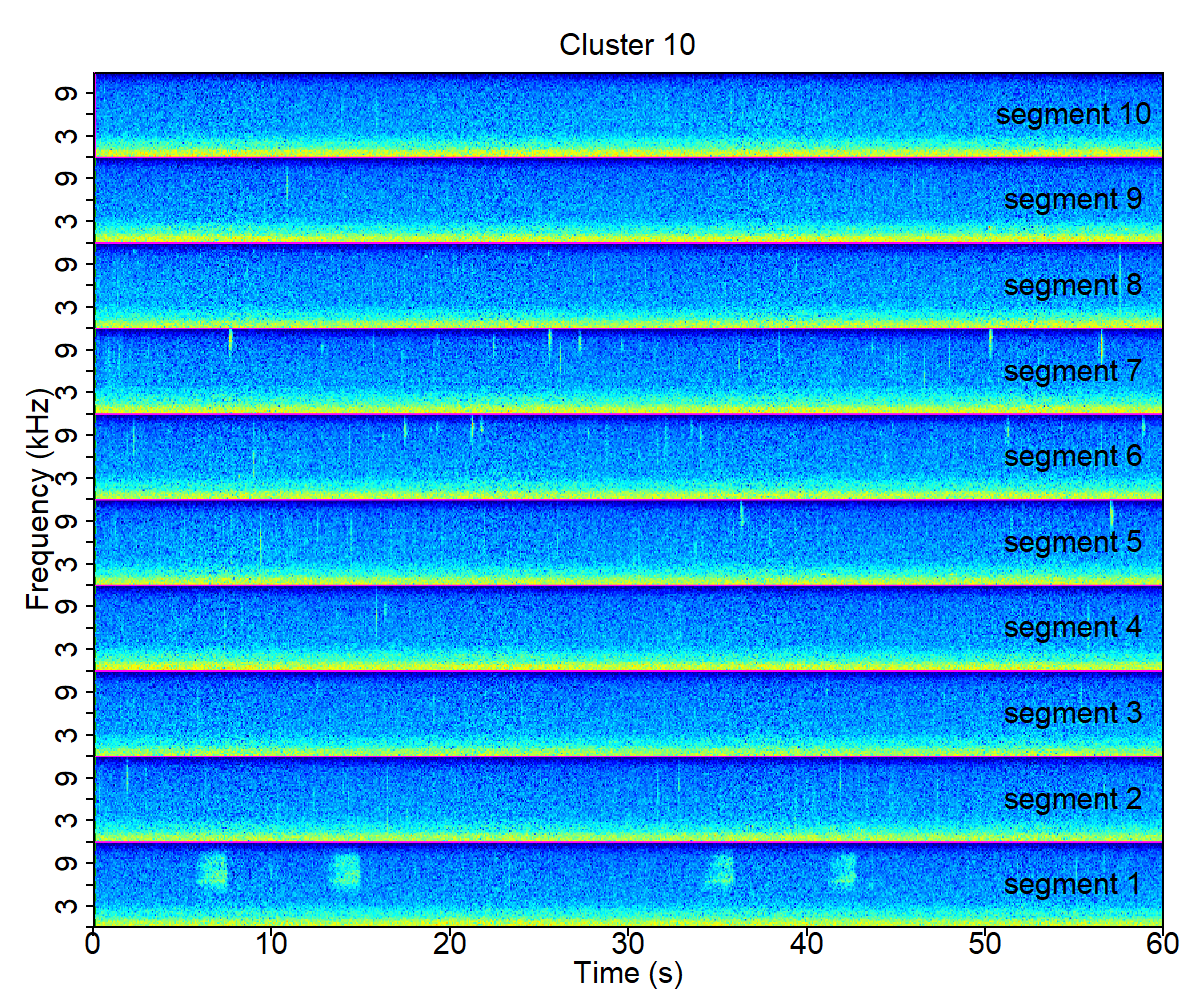

Supplement: Supplemental Information 3 — The spectrograms were computed using a Hann window, FFT = 512, window overlap of 50%, and frame size of 100%. The X-axis represents time, the Y-axis represents frequency. There are 10 audio segments for each cluster. [file peerj-11-16462-s003.zip › Supplemental_Information_S3_spec95_01/Cluster 10.png]

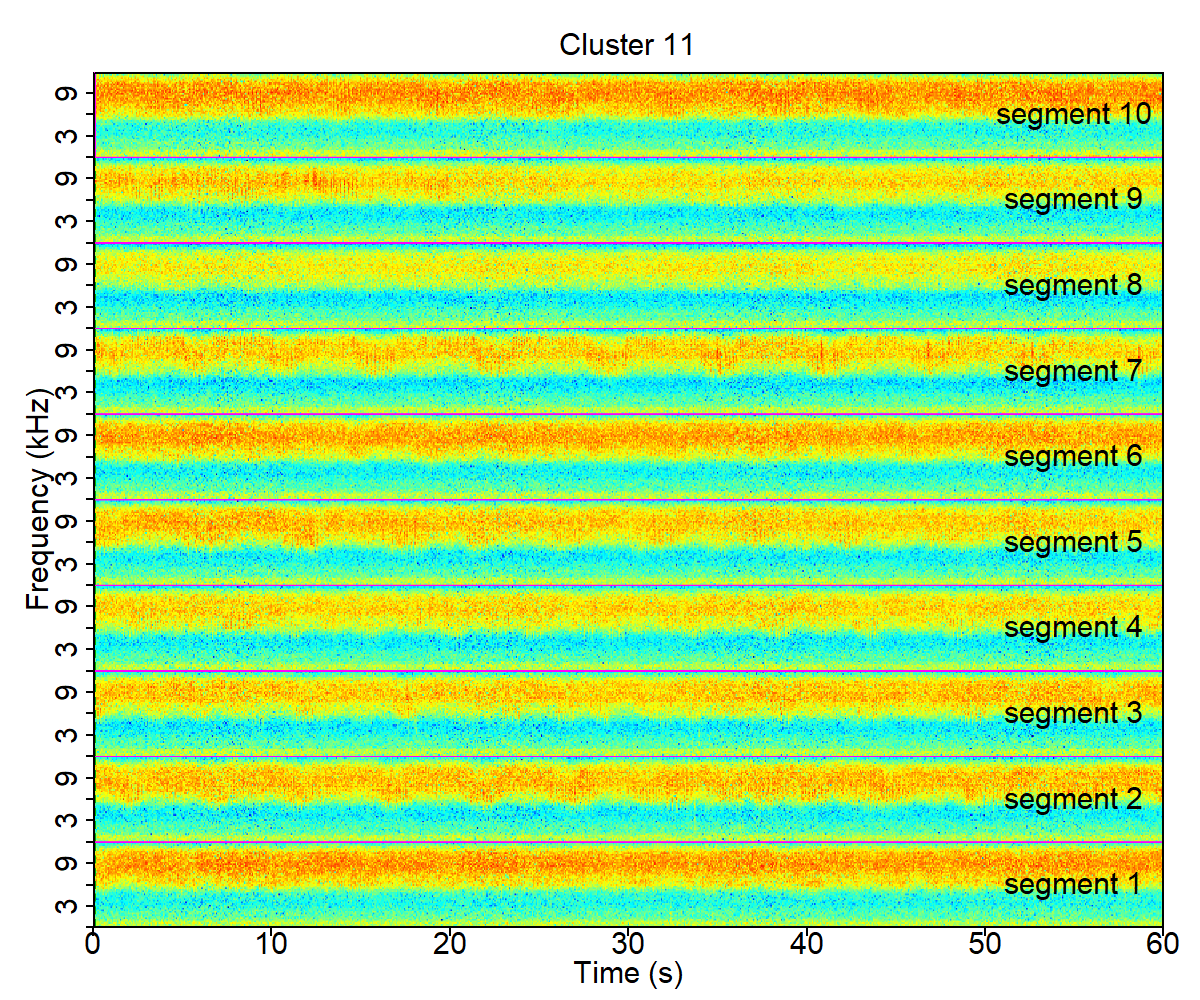

Supplement: Supplemental Information 3 — The spectrograms were computed using a Hann window, FFT = 512, window overlap of 50%, and frame size of 100%. The X-axis represents time, the Y-axis represents frequency. There are 10 audio segments for each cluster. [file peerj-11-16462-s003.zip › Supplemental_Information_S3_spec95_01/Cluster 11.png]

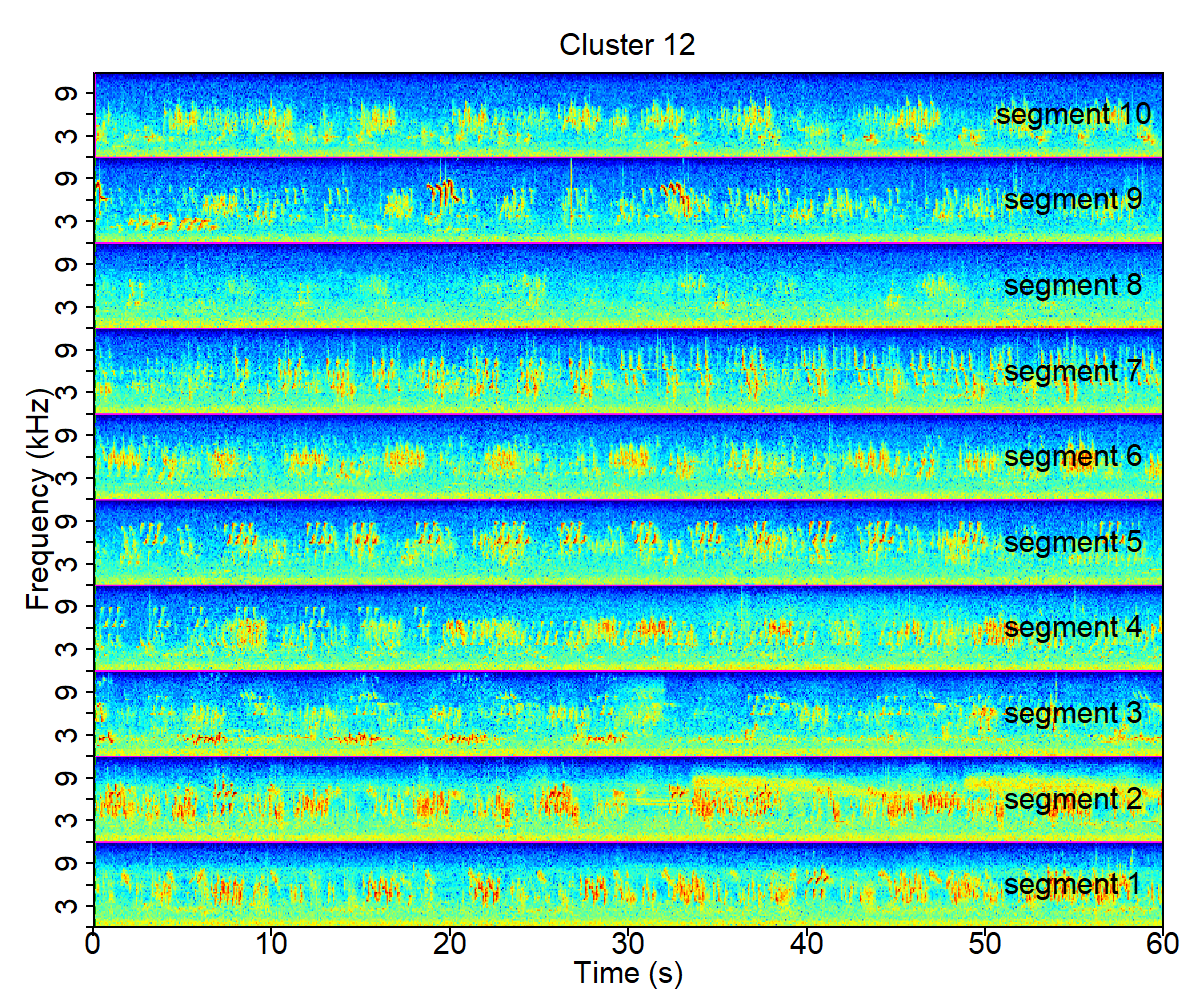

Supplement: Supplemental Information 3 — The spectrograms were computed using a Hann window, FFT = 512, window overlap of 50%, and frame size of 100%. The X-axis represents time, the Y-axis represents frequency. There are 10 audio segments for each cluster. [file peerj-11-16462-s003.zip › Supplemental_Information_S3_spec95_01/Cluster 12.png]

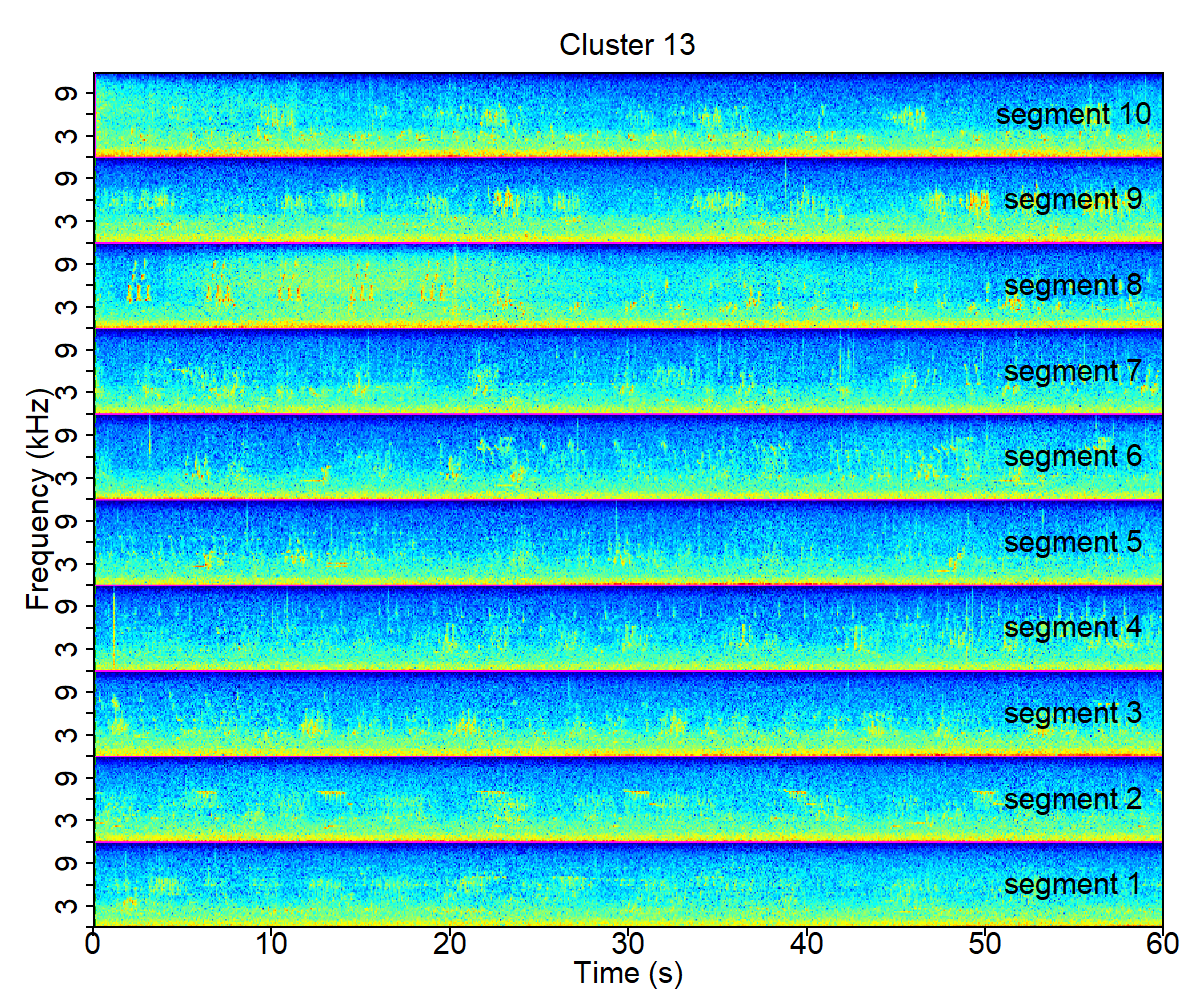

Supplement: Supplemental Information 3 — The spectrograms were computed using a Hann window, FFT = 512, window overlap of 50%, and frame size of 100%. The X-axis represents time, the Y-axis represents frequency. There are 10 audio segments for each cluster. [file peerj-11-16462-s003.zip › Supplemental_Information_S3_spec95_01/Cluster 13.png]

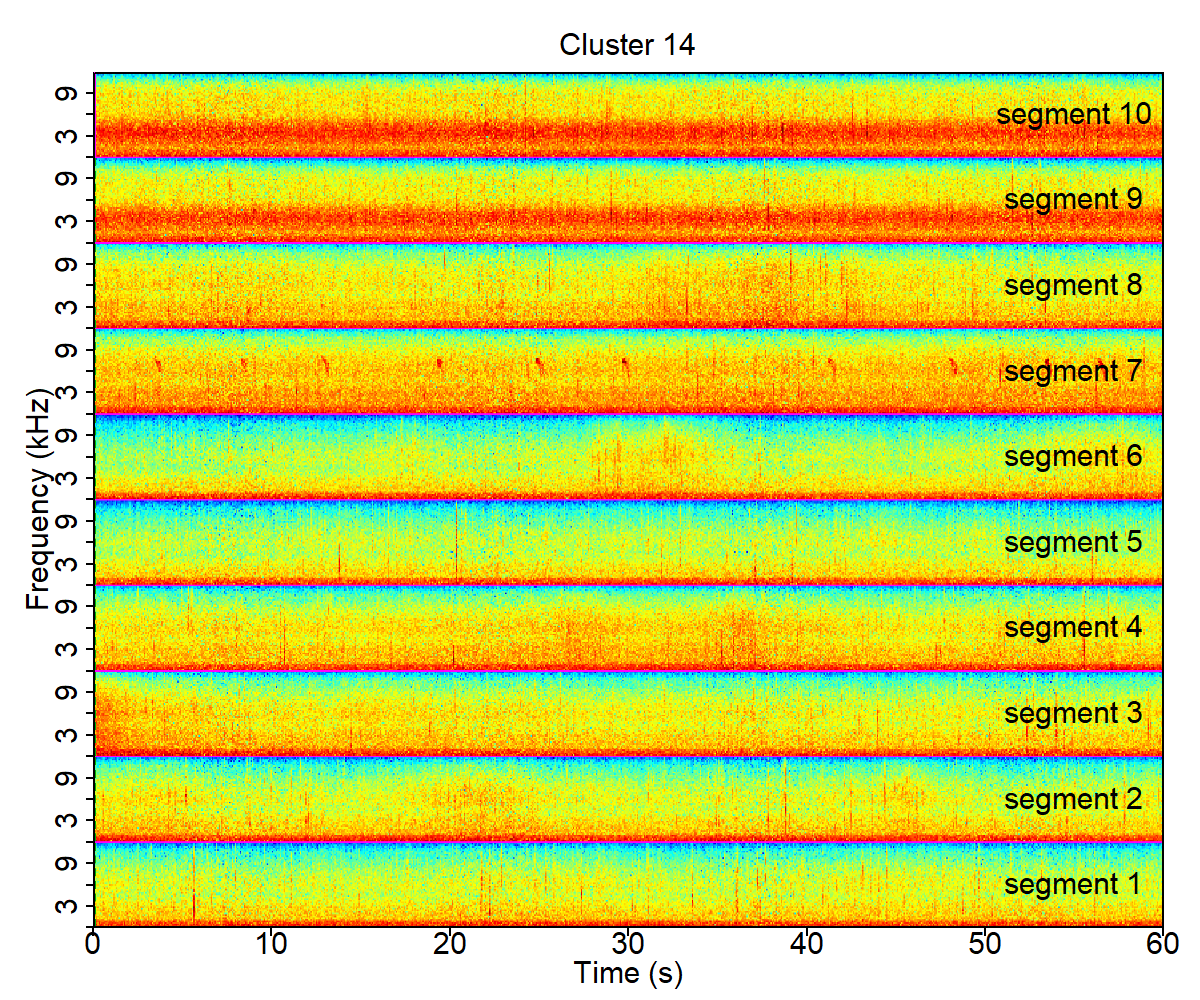

Supplement: Supplemental Information 3 — The spectrograms were computed using a Hann window, FFT = 512, window overlap of 50%, and frame size of 100%. The X-axis represents time, the Y-axis represents frequency. There are 10 audio segments for each cluster. [file peerj-11-16462-s003.zip › Supplemental_Information_S3_spec95_01/Cluster 14.png]

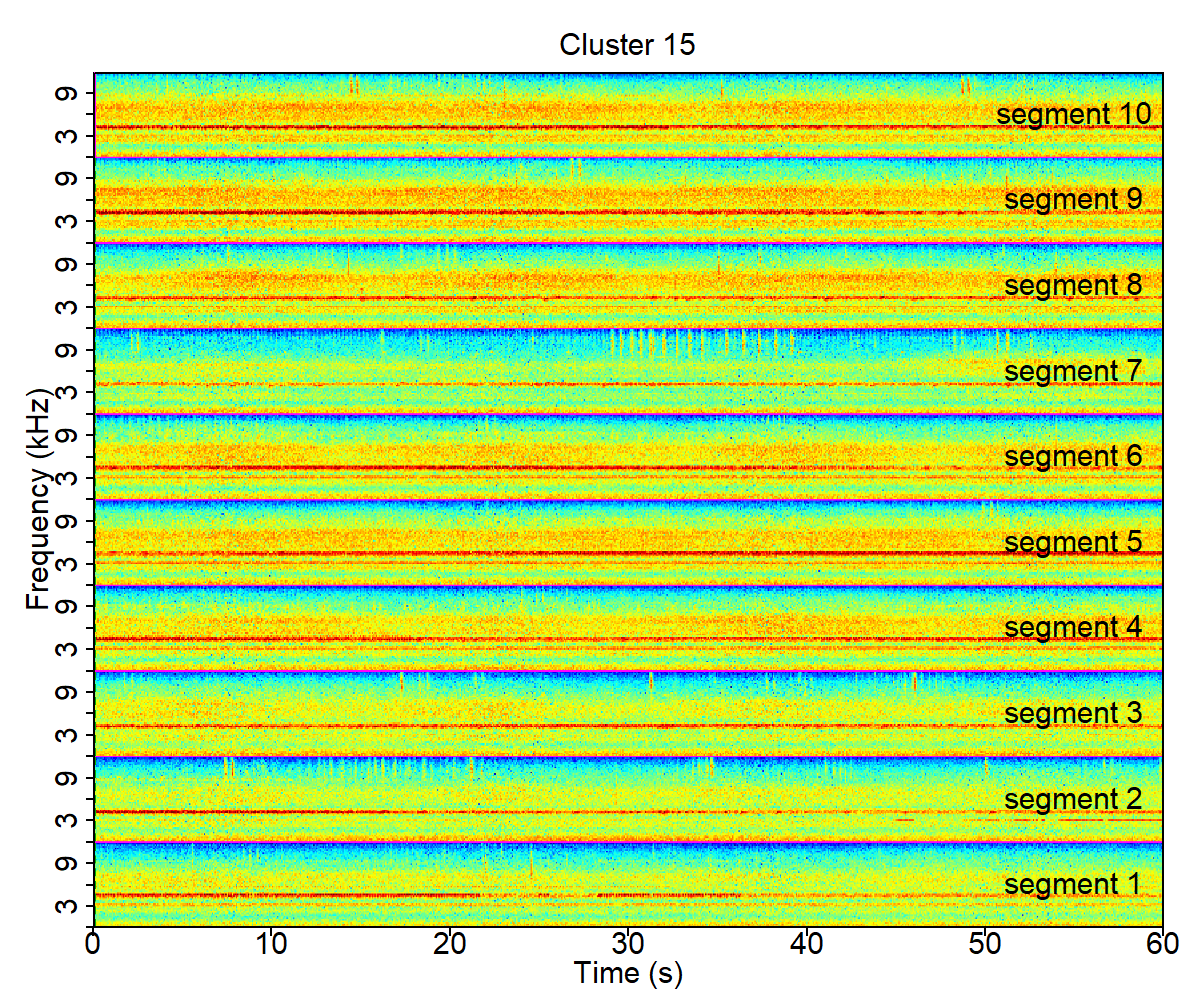

Supplement: Supplemental Information 3 — The spectrograms were computed using a Hann window, FFT = 512, window overlap of 50%, and frame size of 100%. The X-axis represents time, the Y-axis represents frequency. There are 10 audio segments for each cluster. [file peerj-11-16462-s003.zip › Supplemental_Information_S3_spec95_01/Cluster 15.png]

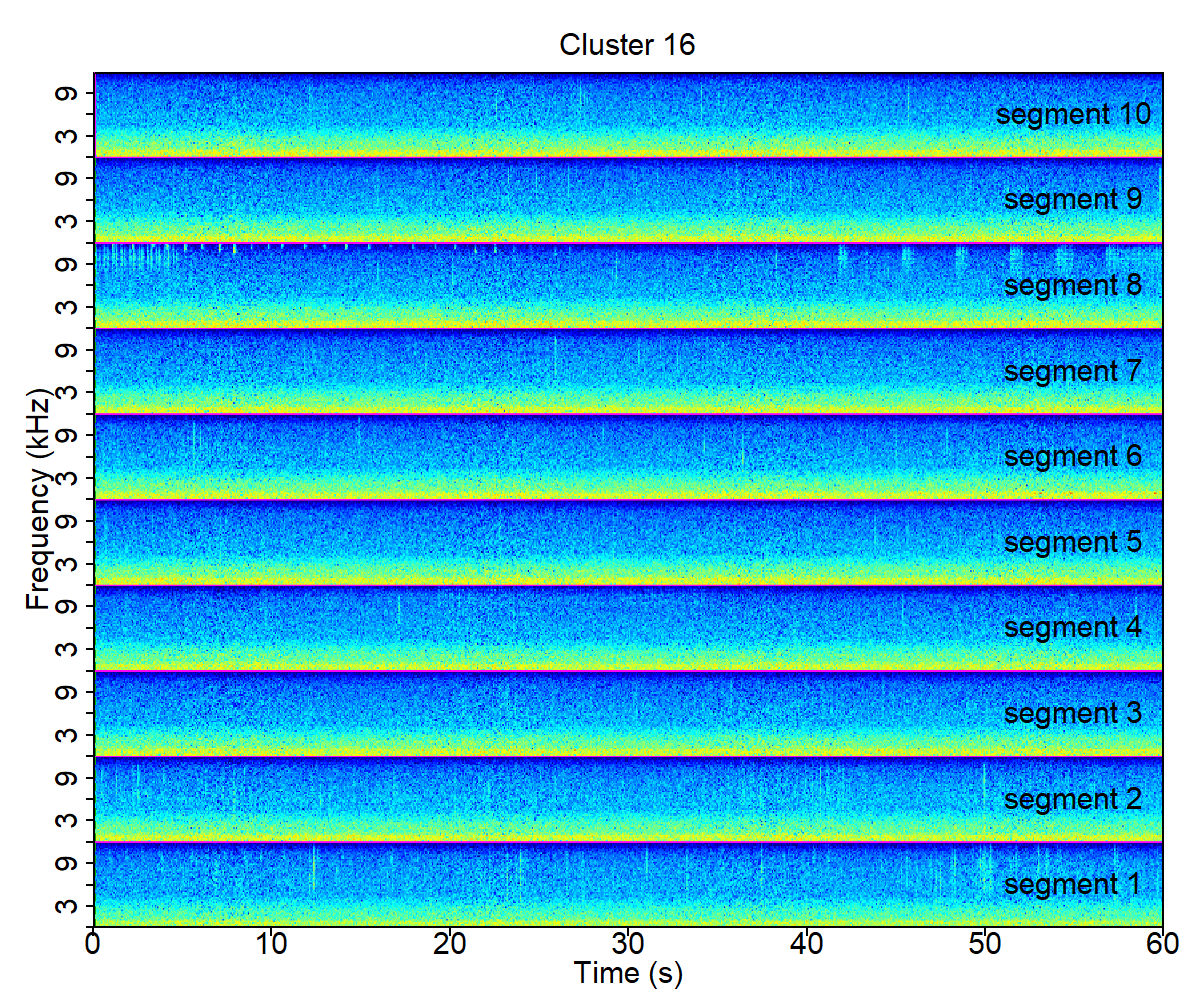

Supplement: Supplemental Information 3 — The spectrograms were computed using a Hann window, FFT = 512, window overlap of 50%, and frame size of 100%. The X-axis represents time, the Y-axis represents frequency. There are 10 audio segments for each cluster. [file peerj-11-16462-s003.zip › Supplemental_Information_S3_spec95_01/Cluster 16.png]

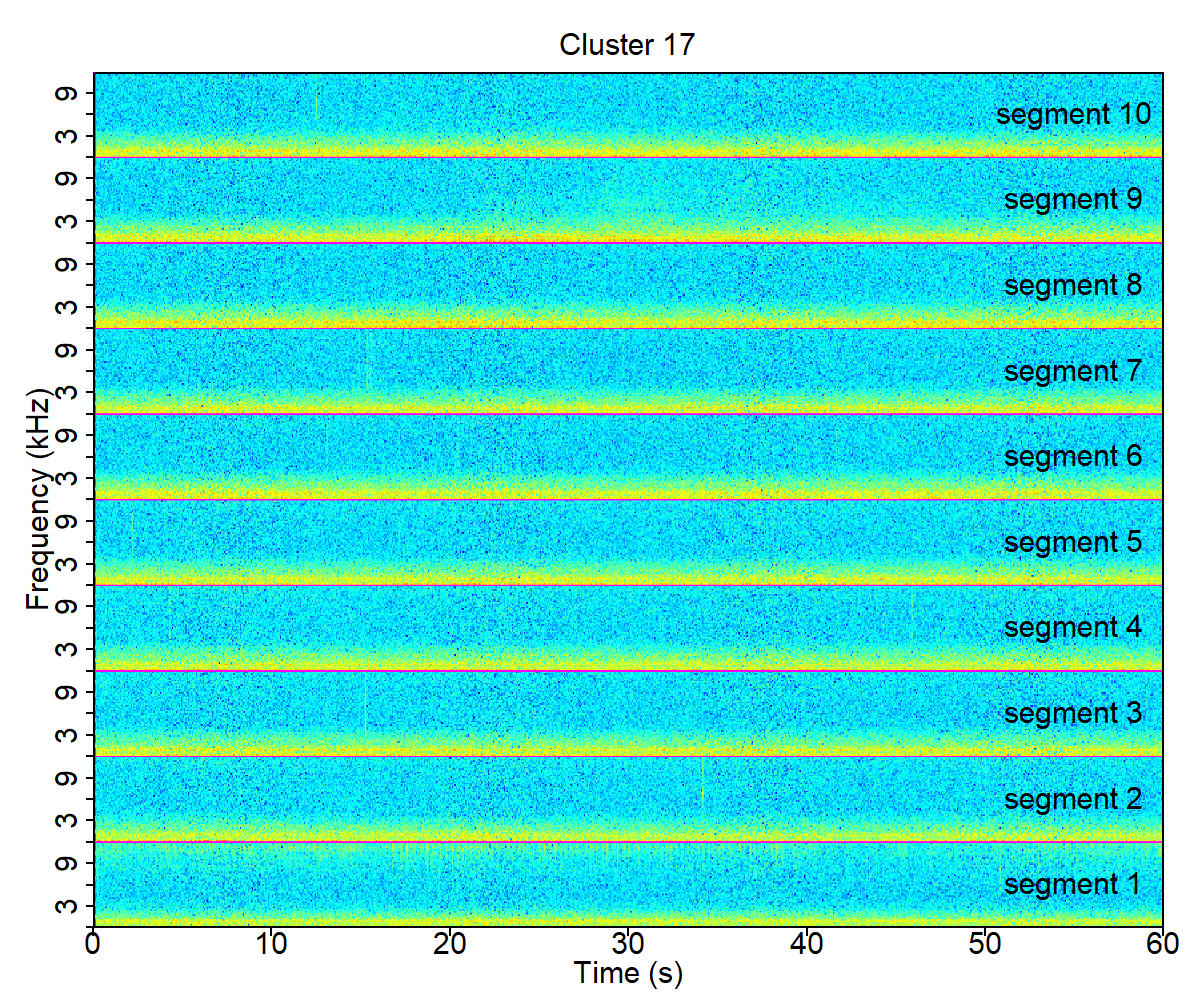

Supplement: Supplemental Information 3 — The spectrograms were computed using a Hann window, FFT = 512, window overlap of 50%, and frame size of 100%. The X-axis represents time, the Y-axis represents frequency. There are 10 audio segments for each cluster. [file peerj-11-16462-s003.zip › Supplemental_Information_S3_spec95_01/Cluster 17.png]

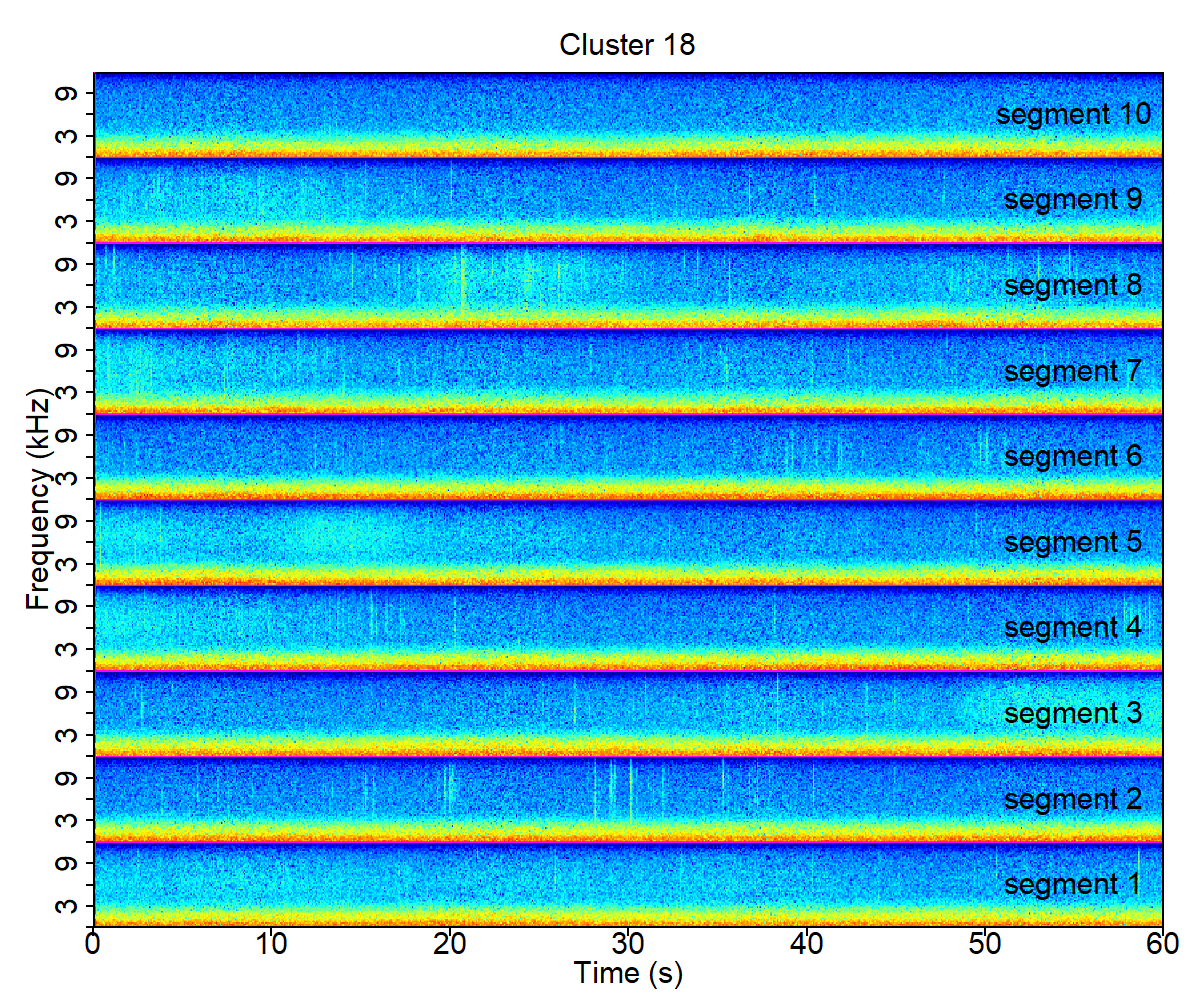

Supplement: Supplemental Information 3 — The spectrograms were computed using a Hann window, FFT = 512, window overlap of 50%, and frame size of 100%. The X-axis represents time, the Y-axis represents frequency. There are 10 audio segments for each cluster. [file peerj-11-16462-s003.zip › Supplemental_Information_S3_spec95_01/Cluster 18.png]

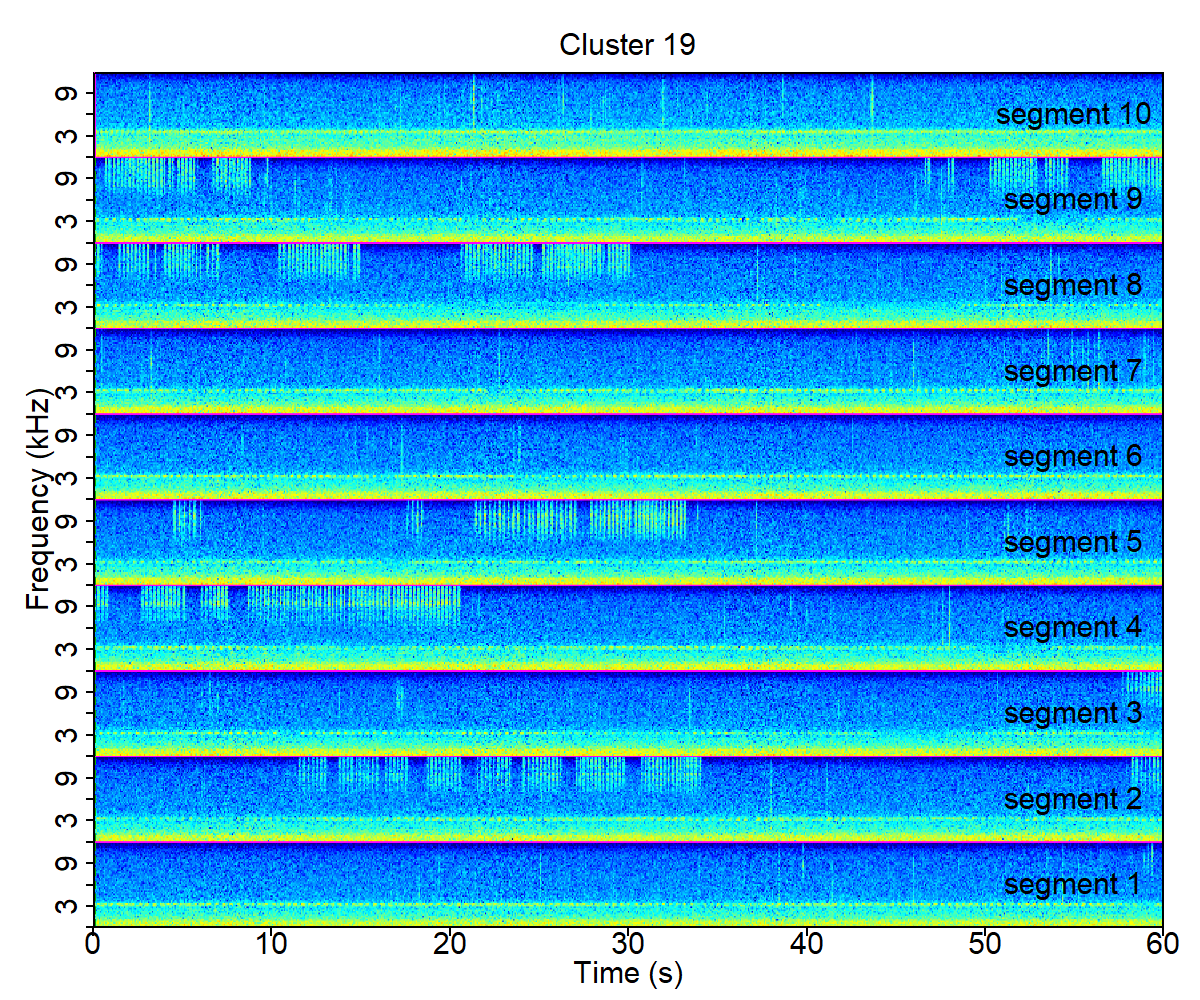

Supplement: Supplemental Information 4 — The spectrograms were computed using a Hann window, FFT = 512, window overlap of 50%, and frame size of 100%. The X-axis represents time, the Y-axis represents frequency. There are 10 audio segments for each cluster. [file peerj-11-16462-s004.zip › Supplemental_Information_S3_spec95_02/Cluster 19.png]

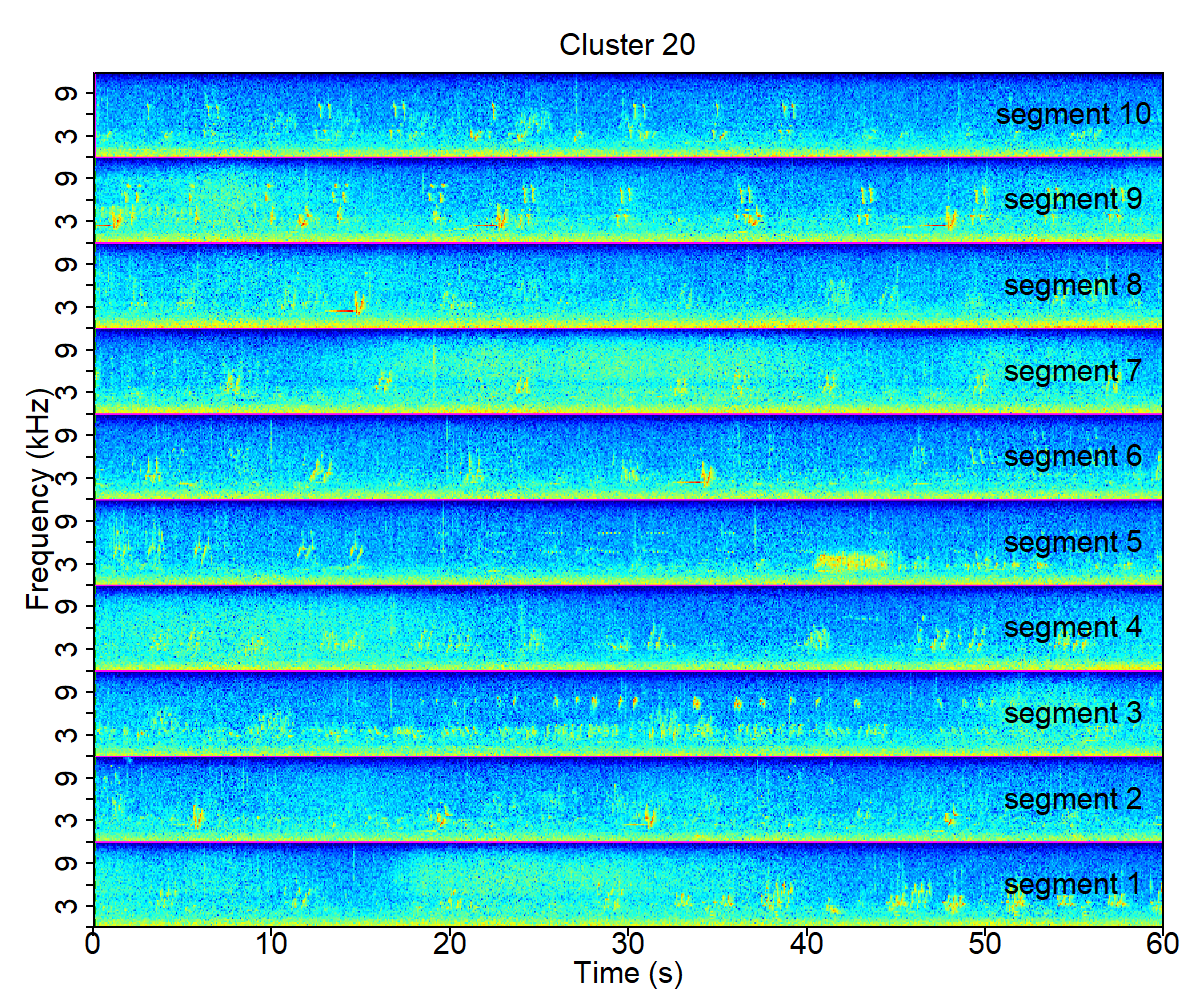

Supplement: Supplemental Information 4 — The spectrograms were computed using a Hann window, FFT = 512, window overlap of 50%, and frame size of 100%. The X-axis represents time, the Y-axis represents frequency. There are 10 audio segments for each cluster. [file peerj-11-16462-s004.zip › Supplemental_Information_S3_spec95_02/Cluster 20.png]

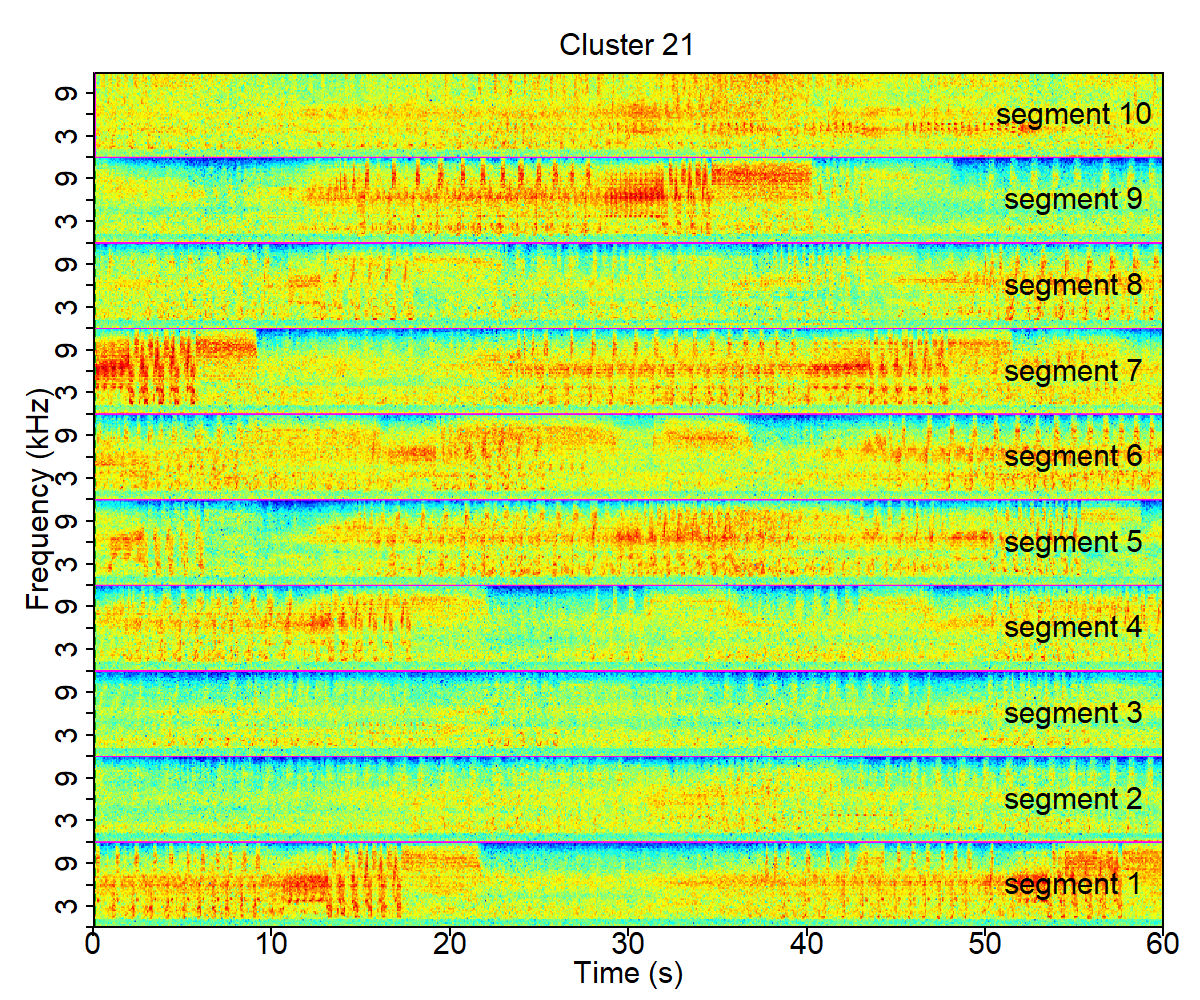

Supplement: Supplemental Information 4 — The spectrograms were computed using a Hann window, FFT = 512, window overlap of 50%, and frame size of 100%. The X-axis represents time, the Y-axis represents frequency. There are 10 audio segments for each cluster. [file peerj-11-16462-s004.zip › Supplemental_Information_S3_spec95_02/Cluster 21.png]

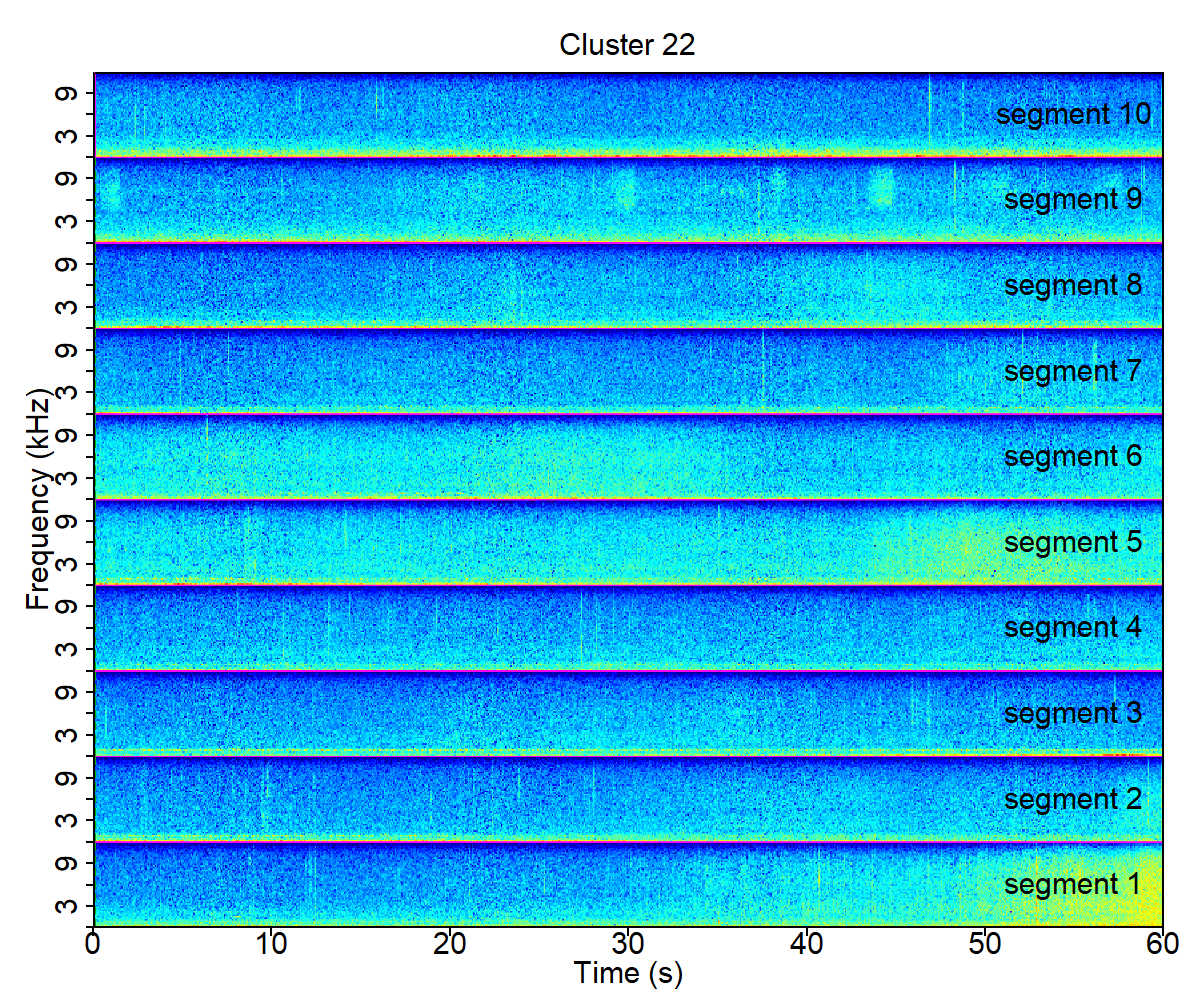

Supplement: Supplemental Information 4 — The spectrograms were computed using a Hann window, FFT = 512, window overlap of 50%, and frame size of 100%. The X-axis represents time, the Y-axis represents frequency. There are 10 audio segments for each cluster. [file peerj-11-16462-s004.zip › Supplemental_Information_S3_spec95_02/Cluster 22.png]

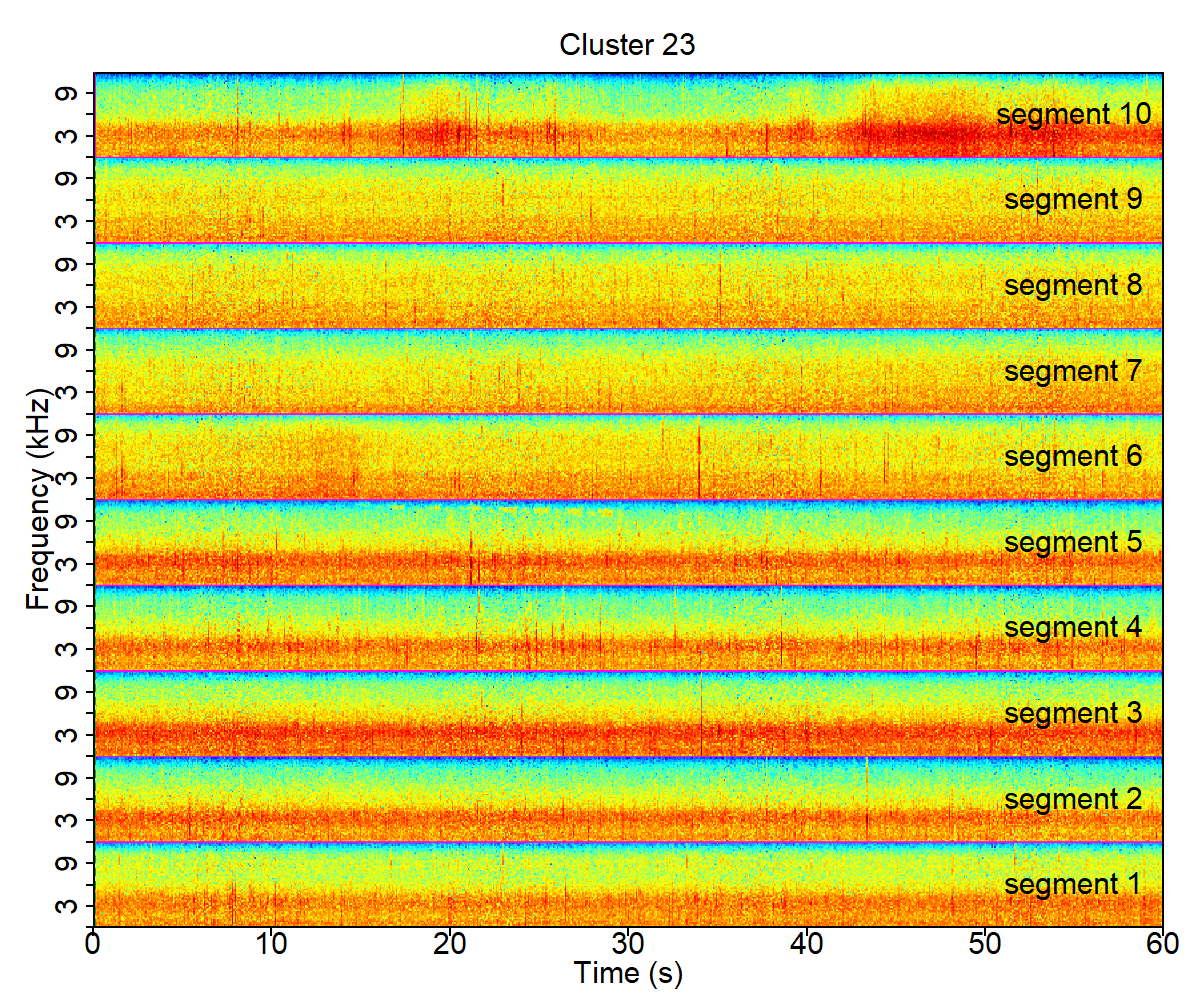

Supplement: Supplemental Information 4 — The spectrograms were computed using a Hann window, FFT = 512, window overlap of 50%, and frame size of 100%. The X-axis represents time, the Y-axis represents frequency. There are 10 audio segments for each cluster. [file peerj-11-16462-s004.zip › Supplemental_Information_S3_spec95_02/Cluster 23.png]

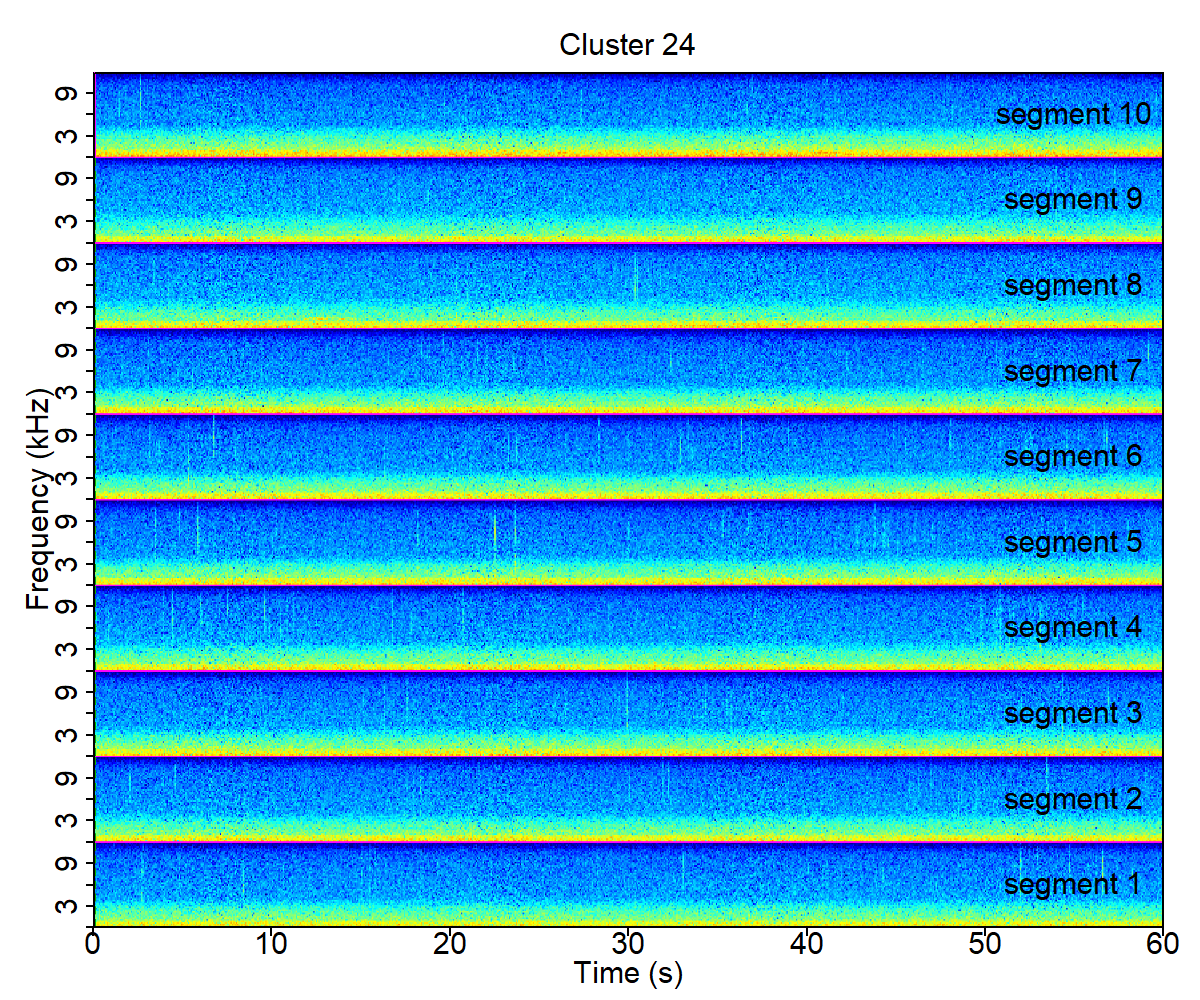

Supplement: Supplemental Information 4 — The spectrograms were computed using a Hann window, FFT = 512, window overlap of 50%, and frame size of 100%. The X-axis represents time, the Y-axis represents frequency. There are 10 audio segments for each cluster. [file peerj-11-16462-s004.zip › Supplemental_Information_S3_spec95_02/Cluster 24.png]

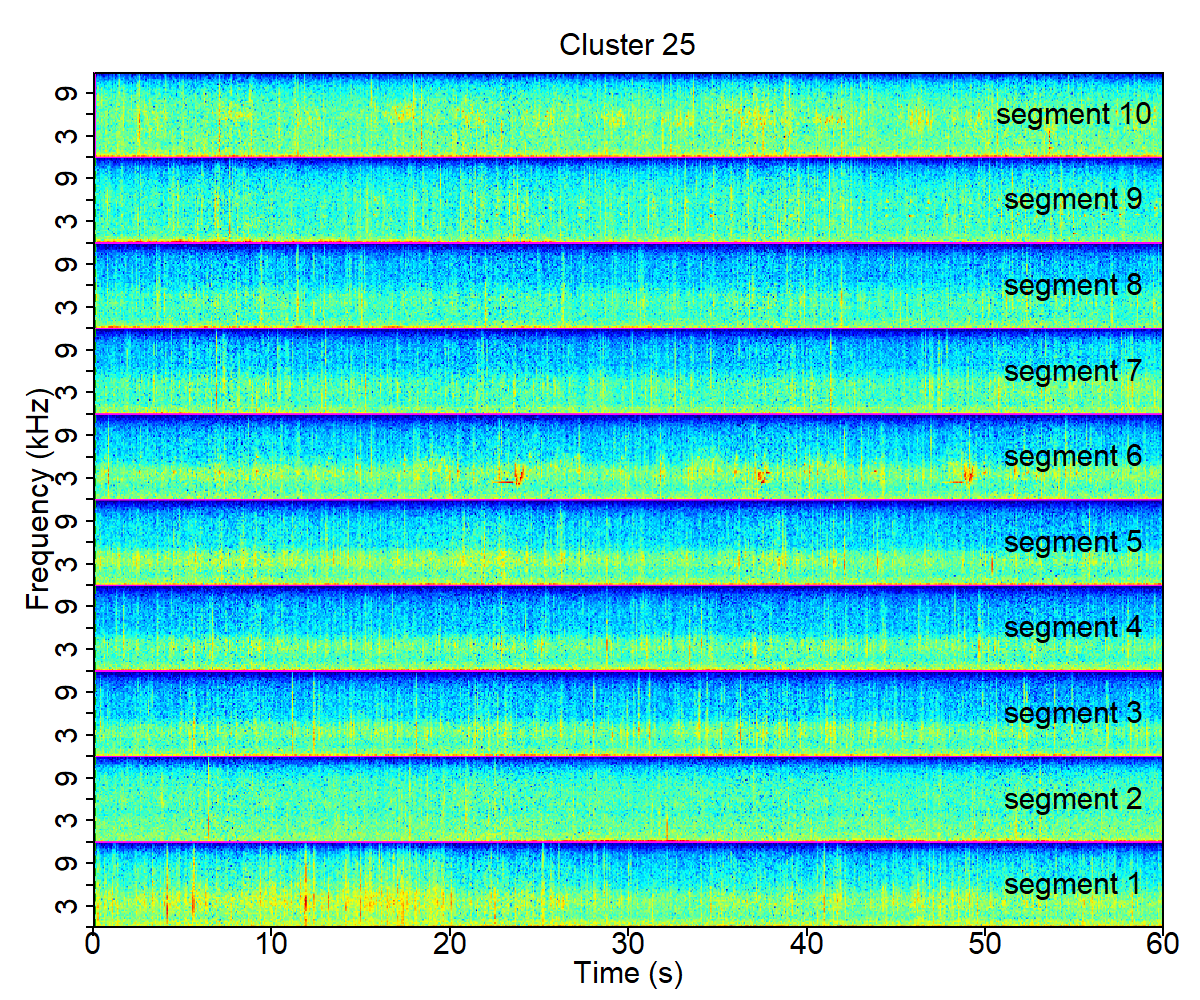

Supplement: Supplemental Information 4 — The spectrograms were computed using a Hann window, FFT = 512, window overlap of 50%, and frame size of 100%. The X-axis represents time, the Y-axis represents frequency. There are 10 audio segments for each cluster. [file peerj-11-16462-s004.zip › Supplemental_Information_S3_spec95_02/Cluster 25.png]

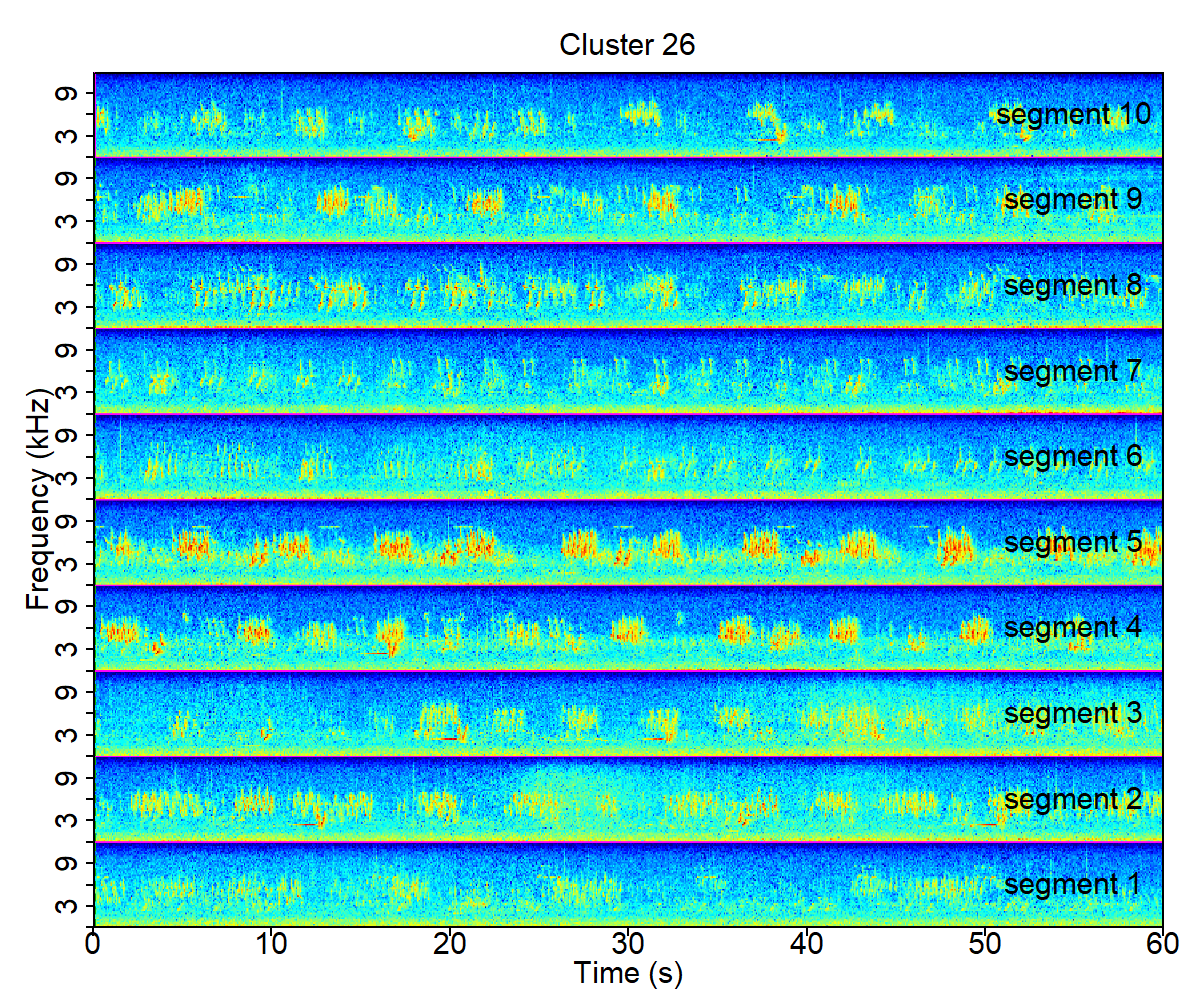

Supplement: Supplemental Information 4 — The spectrograms were computed using a Hann window, FFT = 512, window overlap of 50%, and frame size of 100%. The X-axis represents time, the Y-axis represents frequency. There are 10 audio segments for each cluster. [file peerj-11-16462-s004.zip › Supplemental_Information_S3_spec95_02/Cluster 26.png]

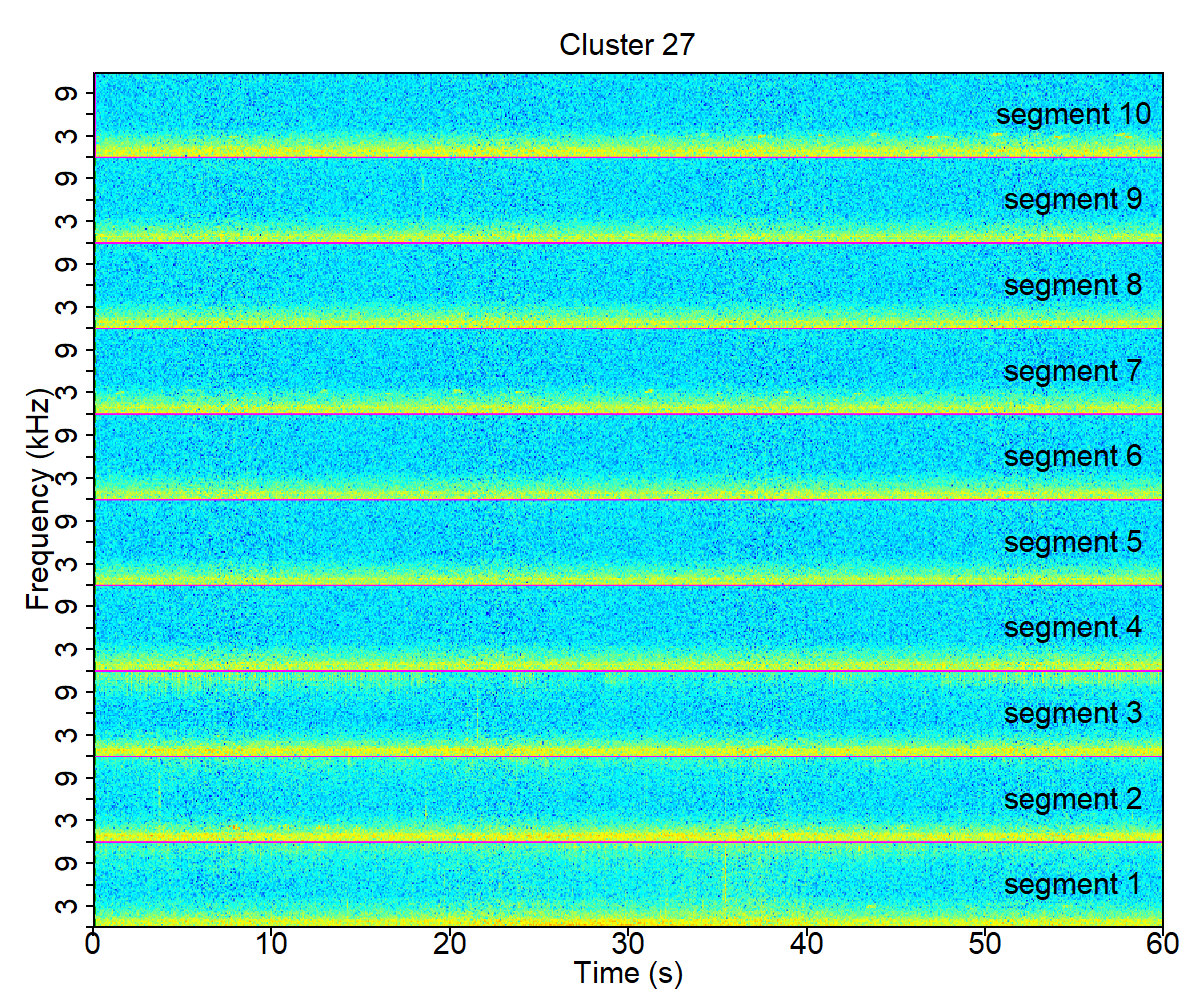

Supplement: Supplemental Information 4 — The spectrograms were computed using a Hann window, FFT = 512, window overlap of 50%, and frame size of 100%. The X-axis represents time, the Y-axis represents frequency. There are 10 audio segments for each cluster. [file peerj-11-16462-s004.zip › Supplemental_Information_S3_spec95_02/Cluster 27.png]

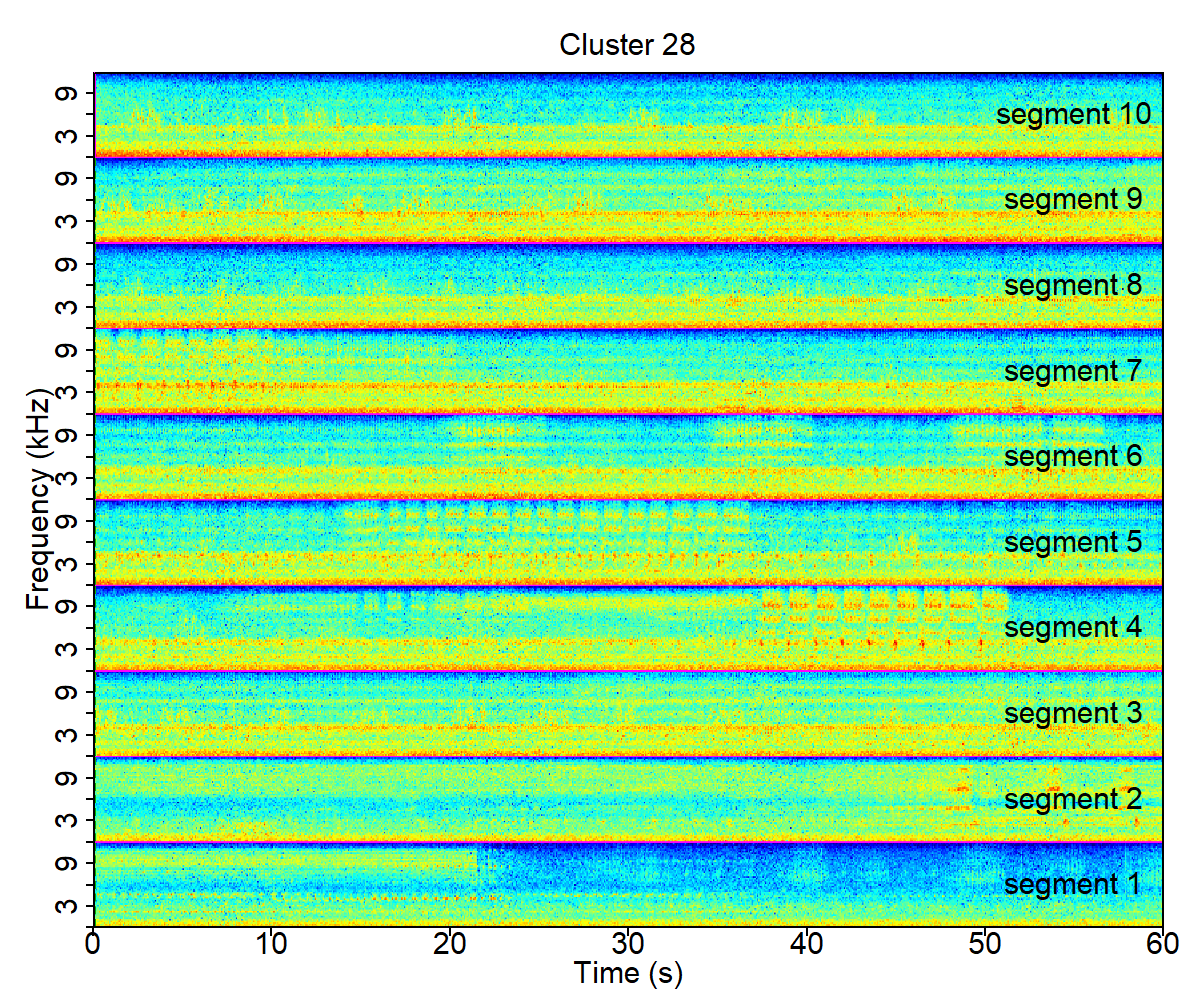

Supplement: Supplemental Information 4 — The spectrograms were computed using a Hann window, FFT = 512, window overlap of 50%, and frame size of 100%. The X-axis represents time, the Y-axis represents frequency. There are 10 audio segments for each cluster. [file peerj-11-16462-s004.zip › Supplemental_Information_S3_spec95_02/Cluster 28.png]

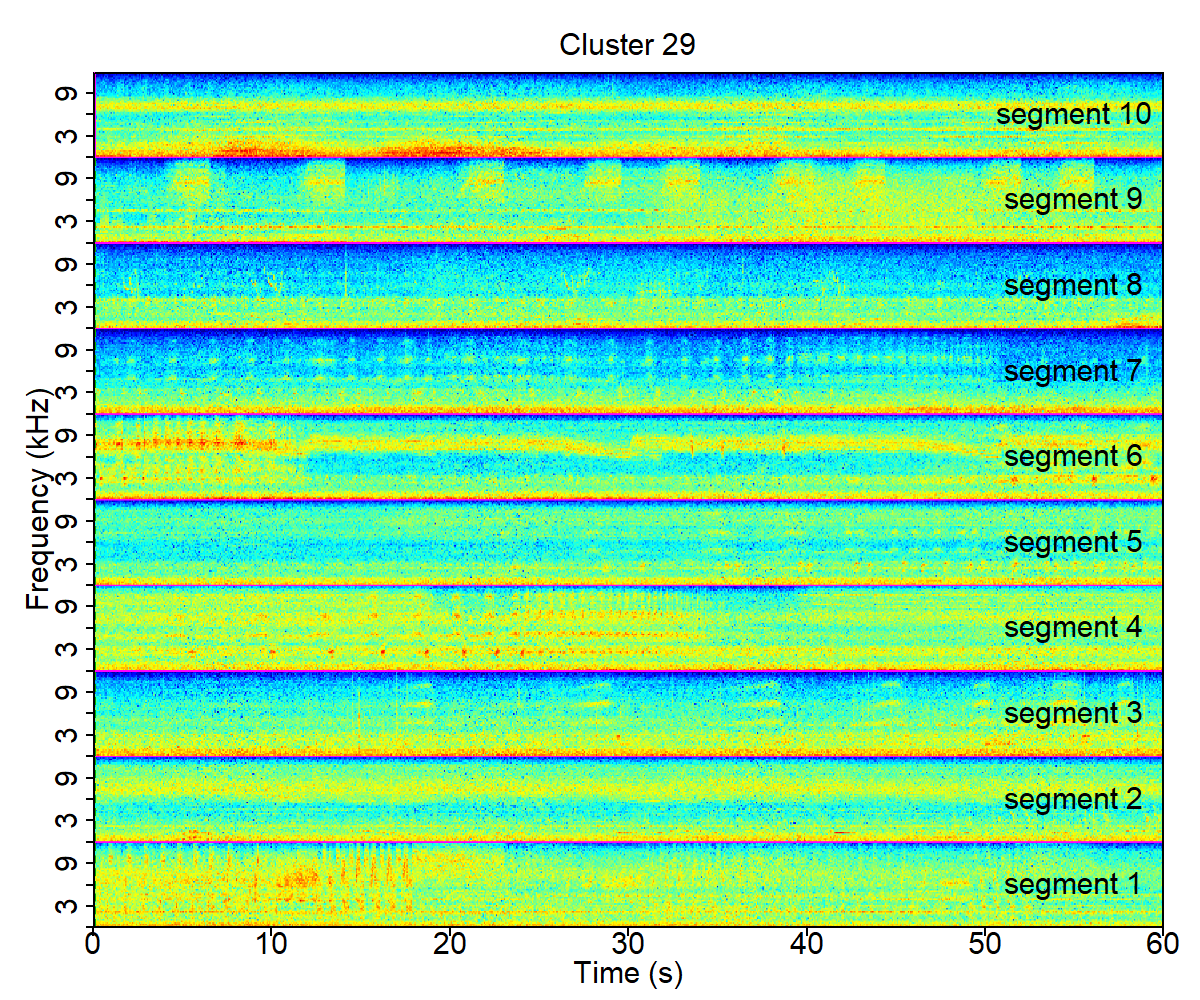

Supplement: Supplemental Information 4 — The spectrograms were computed using a Hann window, FFT = 512, window overlap of 50%, and frame size of 100%. The X-axis represents time, the Y-axis represents frequency. There are 10 audio segments for each cluster. [file peerj-11-16462-s004.zip › Supplemental_Information_S3_spec95_02/Cluster 29.png]

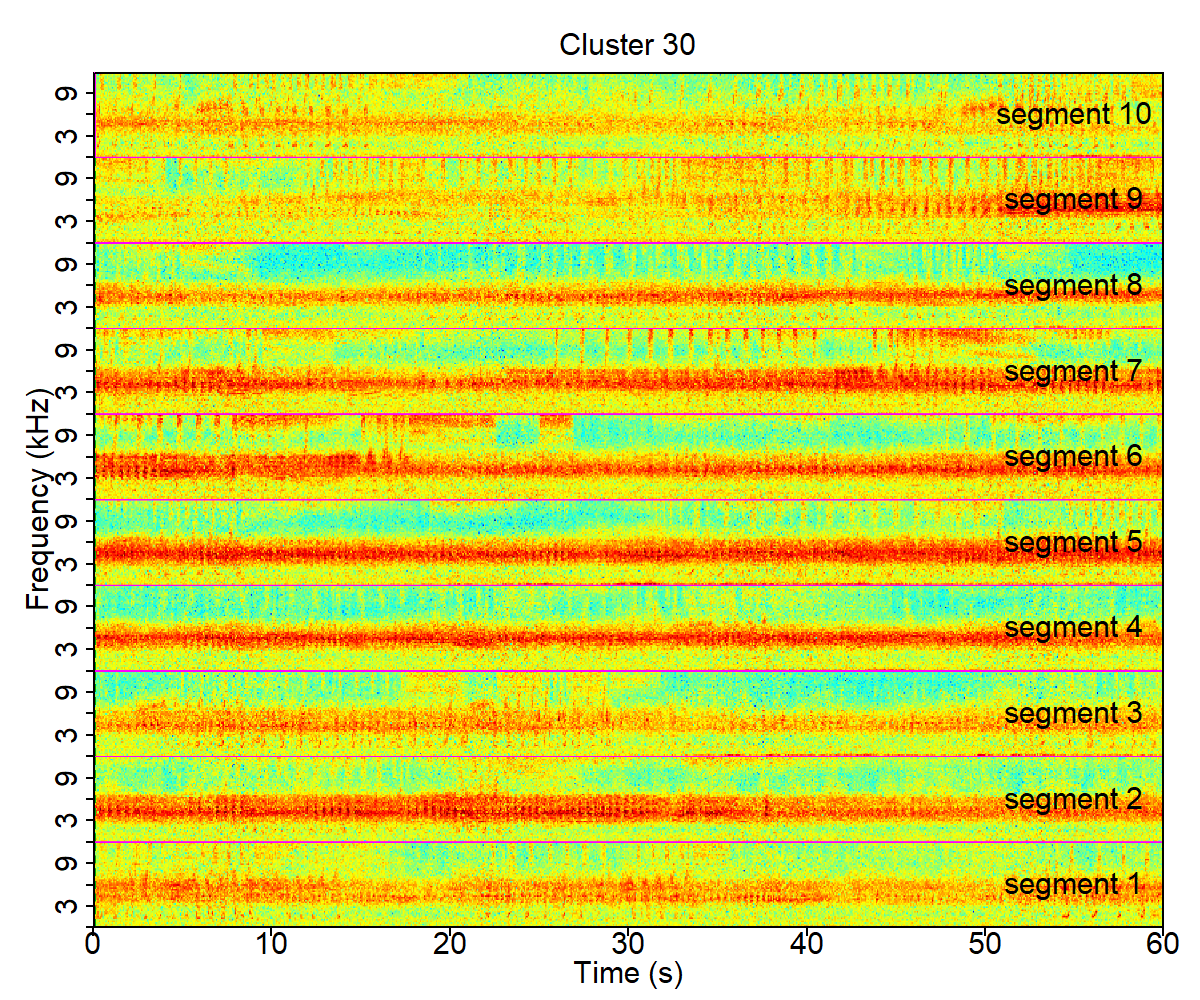

Supplement: Supplemental Information 4 — The spectrograms were computed using a Hann window, FFT = 512, window overlap of 50%, and frame size of 100%. The X-axis represents time, the Y-axis represents frequency. There are 10 audio segments for each cluster. [file peerj-11-16462-s004.zip › Supplemental_Information_S3_spec95_02/Cluster 30.png]

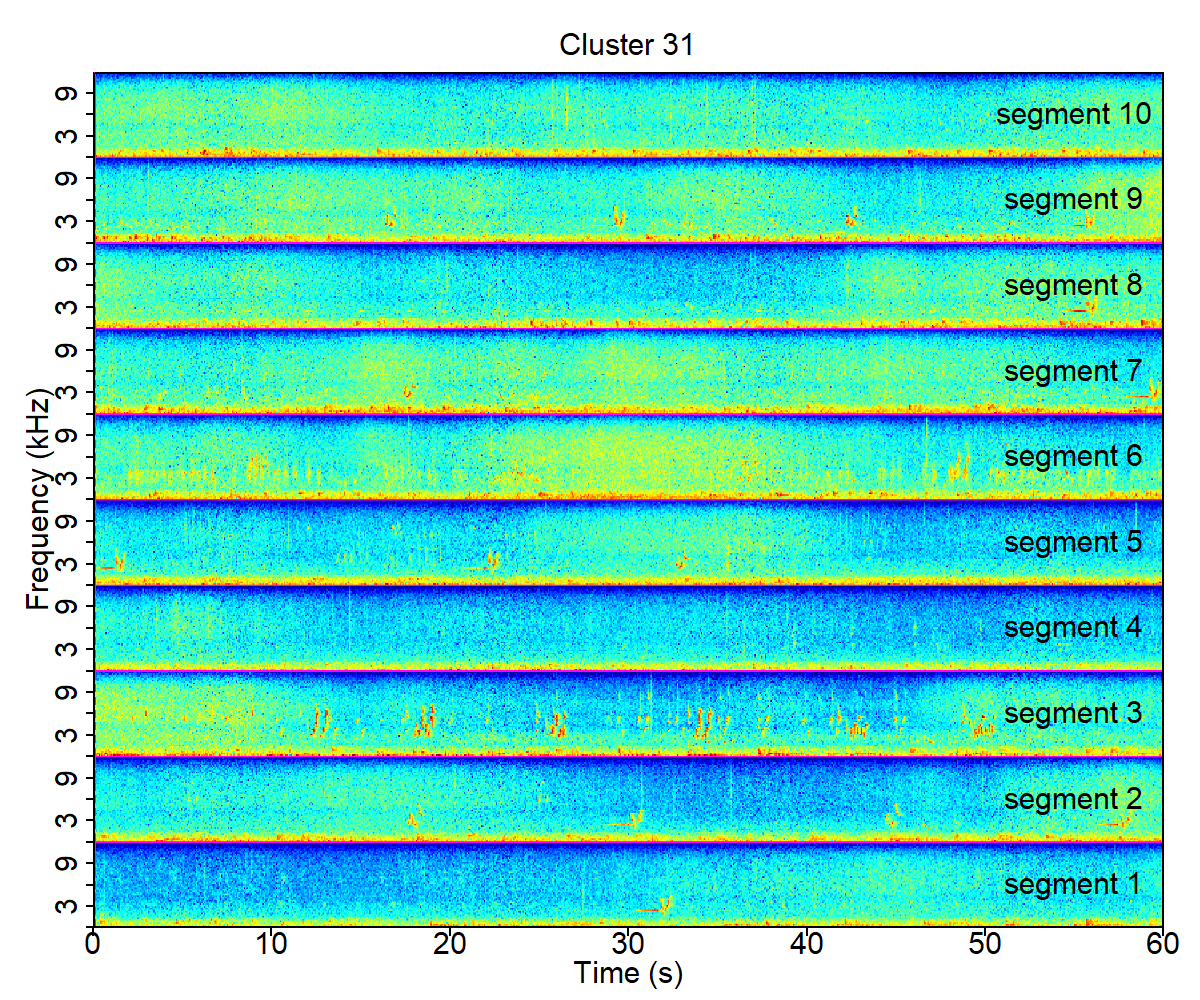

Supplement: Supplemental Information 4 — The spectrograms were computed using a Hann window, FFT = 512, window overlap of 50%, and frame size of 100%. The X-axis represents time, the Y-axis represents frequency. There are 10 audio segments for each cluster. [file peerj-11-16462-s004.zip › Supplemental_Information_S3_spec95_02/Cluster 31.png]

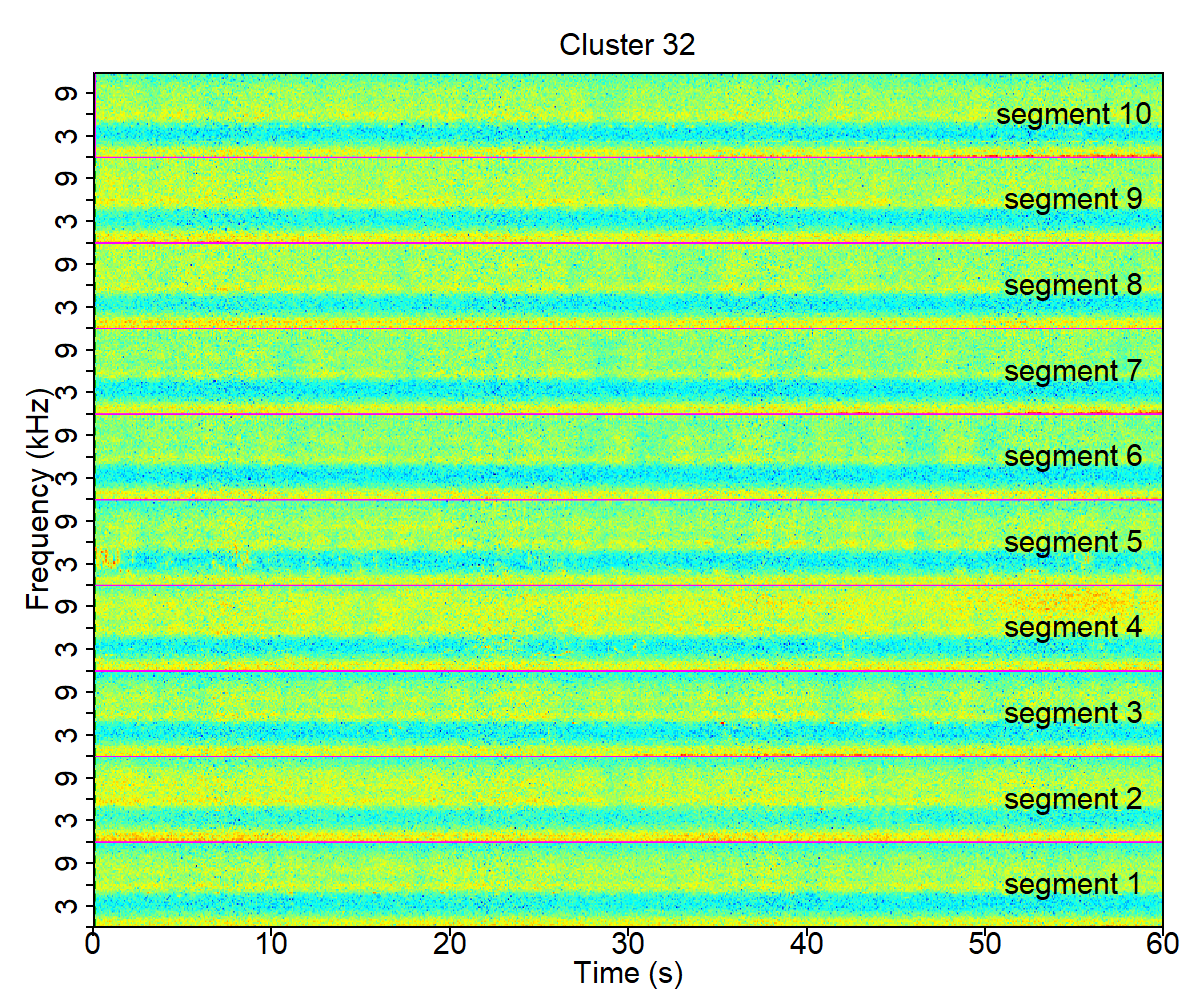

Supplement: Supplemental Information 4 — The spectrograms were computed using a Hann window, FFT = 512, window overlap of 50%, and frame size of 100%. The X-axis represents time, the Y-axis represents frequency. There are 10 audio segments for each cluster. [file peerj-11-16462-s004.zip › Supplemental_Information_S3_spec95_02/Cluster 32.png]

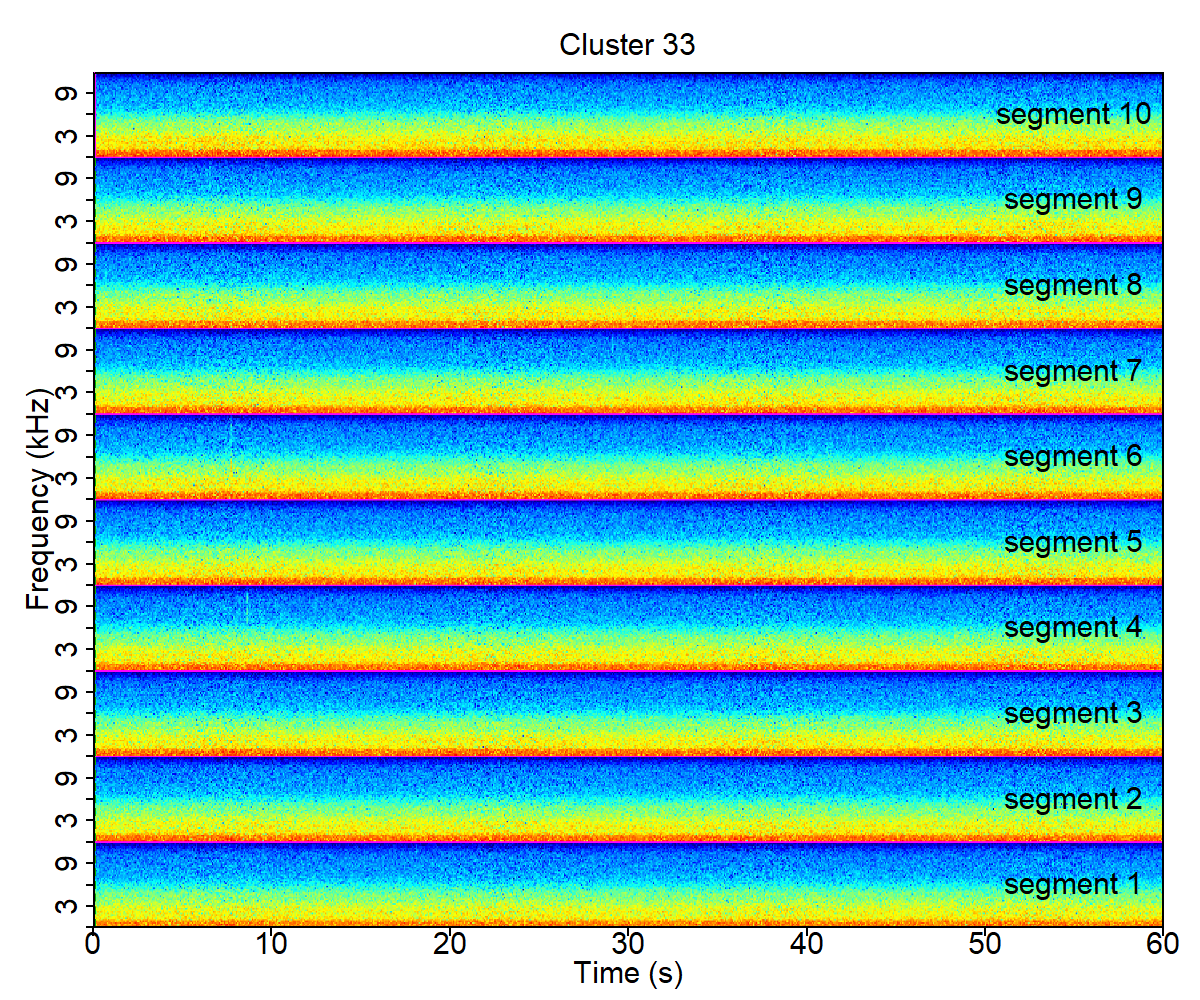

Supplement: Supplemental Information 4 — The spectrograms were computed using a Hann window, FFT = 512, window overlap of 50%, and frame size of 100%. The X-axis represents time, the Y-axis represents frequency. There are 10 audio segments for each cluster. [file peerj-11-16462-s004.zip › Supplemental_Information_S3_spec95_02/Cluster 33.png]

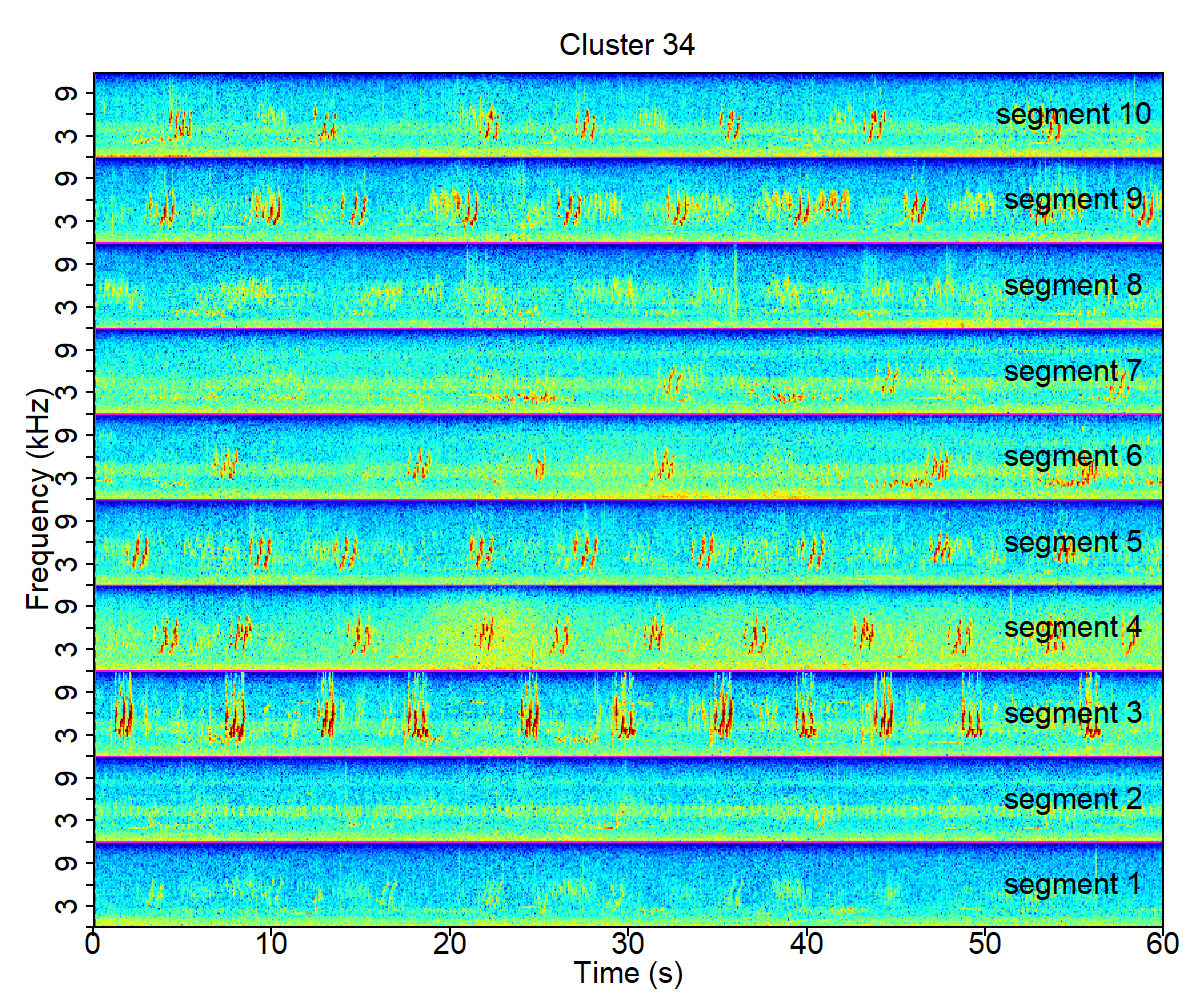

Supplement: Supplemental Information 4 — The spectrograms were computed using a Hann window, FFT = 512, window overlap of 50%, and frame size of 100%. The X-axis represents time, the Y-axis represents frequency. There are 10 audio segments for each cluster. [file peerj-11-16462-s004.zip › Supplemental_Information_S3_spec95_02/Cluster 34.png]

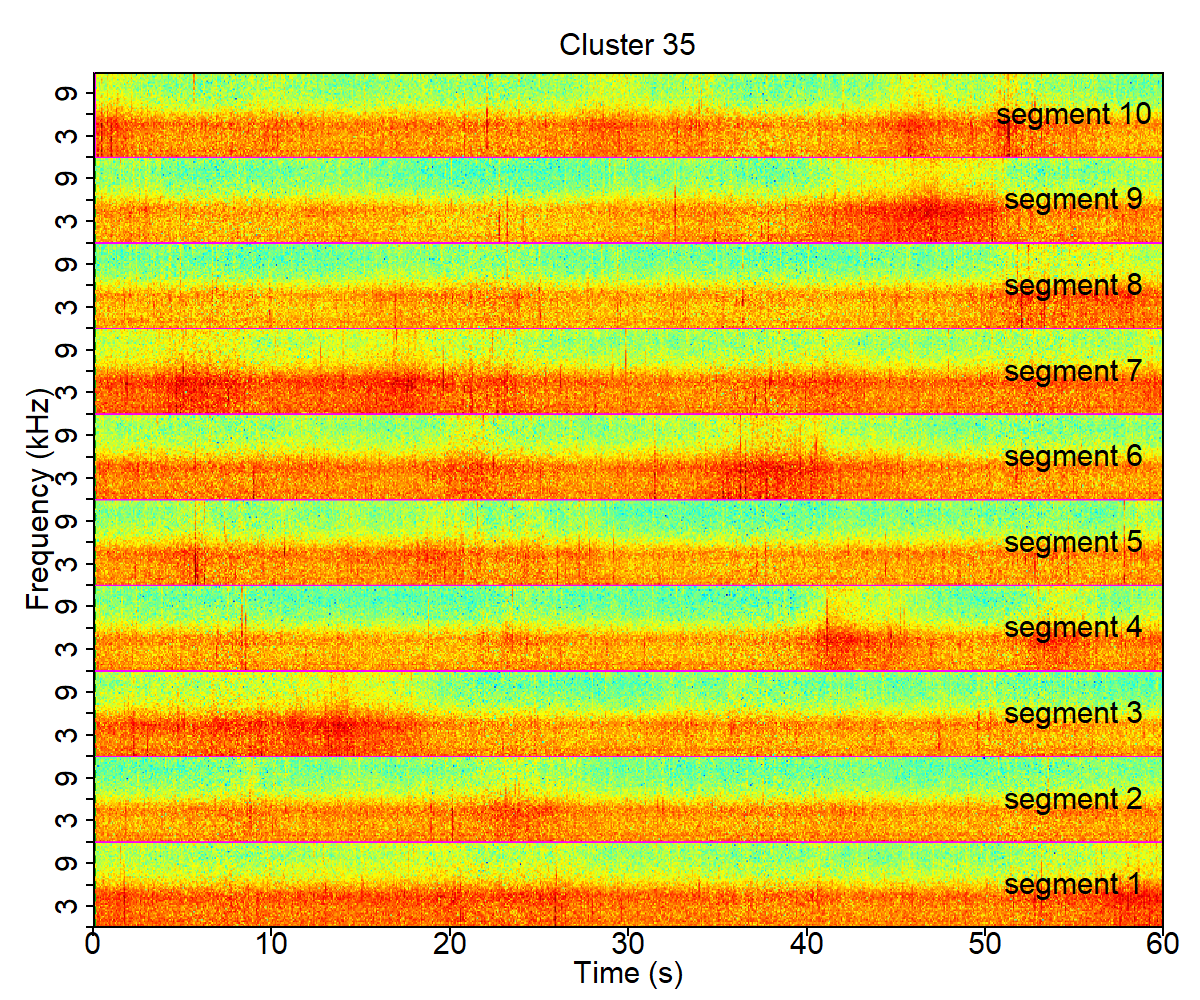

Supplement: Supplemental Information 4 — The spectrograms were computed using a Hann window, FFT = 512, window overlap of 50%, and frame size of 100%. The X-axis represents time, the Y-axis represents frequency. There are 10 audio segments for each cluster. [file peerj-11-16462-s004.zip › Supplemental_Information_S3_spec95_02/Cluster 35.png]

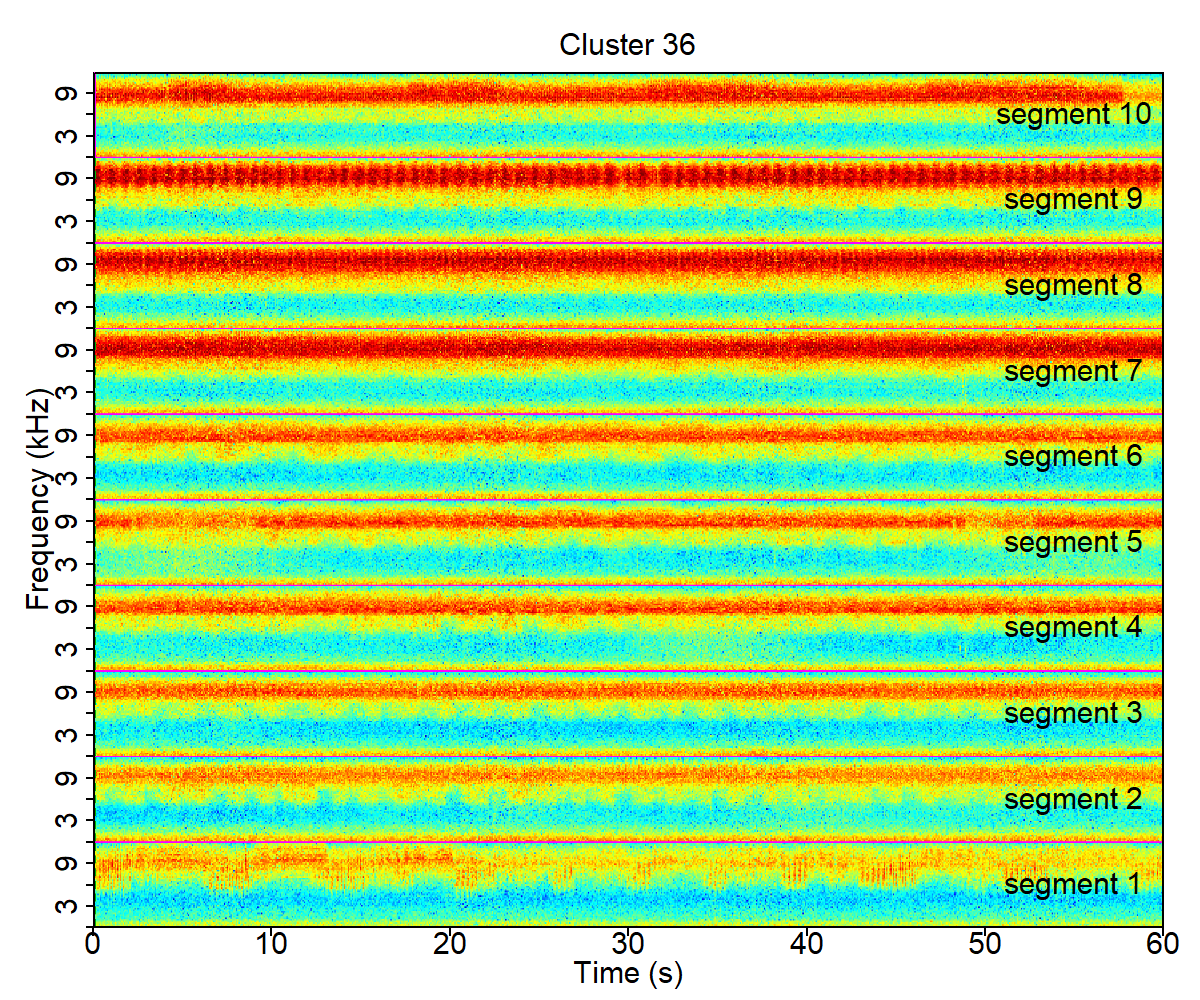

Supplement: Supplemental Information 4 — The spectrograms were computed using a Hann window, FFT = 512, window overlap of 50%, and frame size of 100%. The X-axis represents time, the Y-axis represents frequency. There are 10 audio segments for each cluster. [file peerj-11-16462-s004.zip › Supplemental_Information_S3_spec95_02/Cluster 36.png]

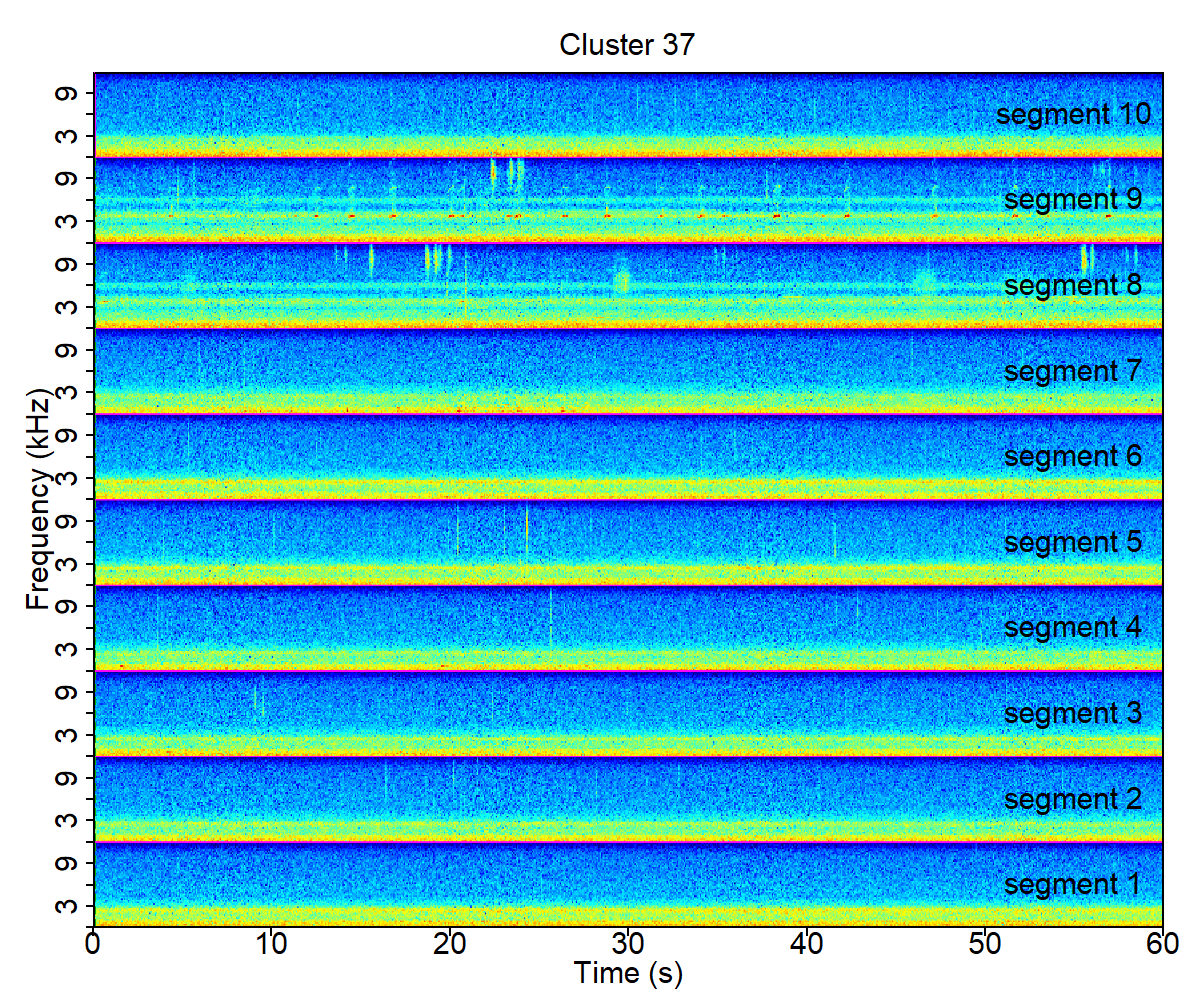

Supplement: Supplemental Information 4 — The spectrograms were computed using a Hann window, FFT = 512, window overlap of 50%, and frame size of 100%. The X-axis represents time, the Y-axis represents frequency. There are 10 audio segments for each cluster. [file peerj-11-16462-s004.zip › Supplemental_Information_S3_spec95_02/Cluster 37.png]

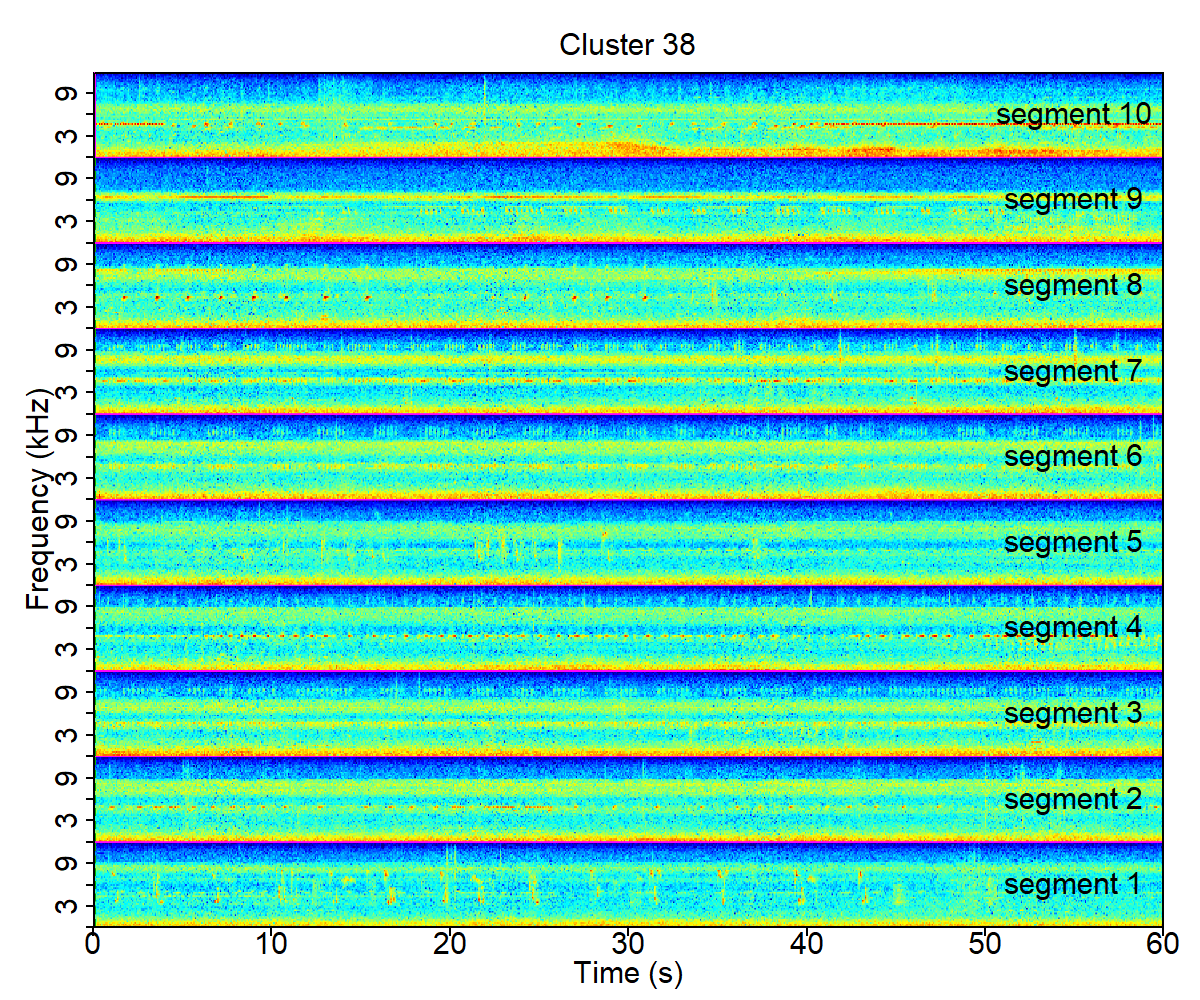

Supplement: Supplemental Information 5 — The spectrograms were computed using a Hann window, FFT = 512, window overlap of 50%, and frame size of 100%. The X-axis represents time, the Y-axis represents frequency. There are 10 audio segments for each cluster. [file peerj-11-16462-s005.zip › Supplemental_Information_S3_spec95_03/Cluster 38.png]

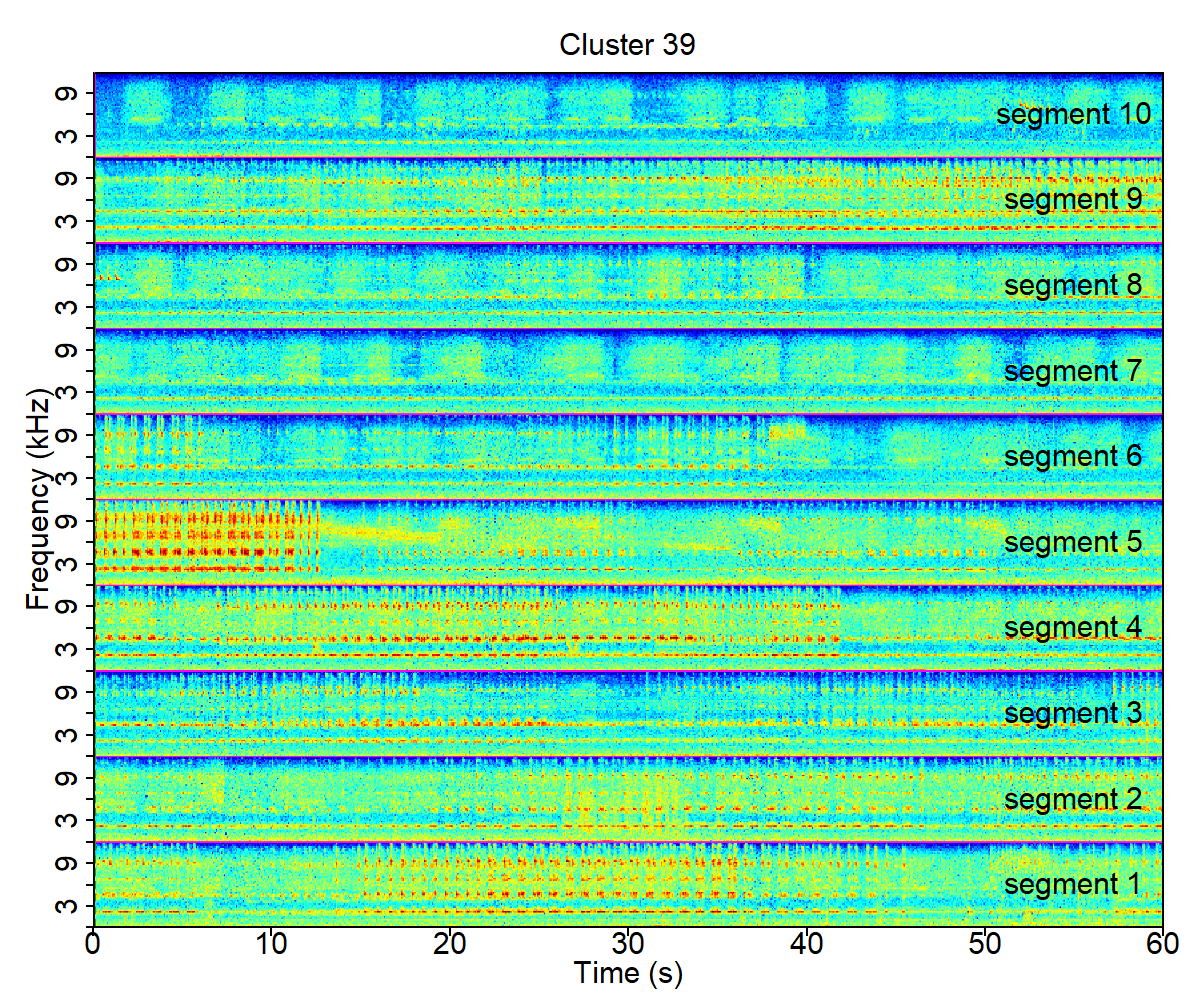

Supplement: Supplemental Information 5 — The spectrograms were computed using a Hann window, FFT = 512, window overlap of 50%, and frame size of 100%. The X-axis represents time, the Y-axis represents frequency. There are 10 audio segments for each cluster. [file peerj-11-16462-s005.zip › Supplemental_Information_S3_spec95_03/Cluster 39.png]

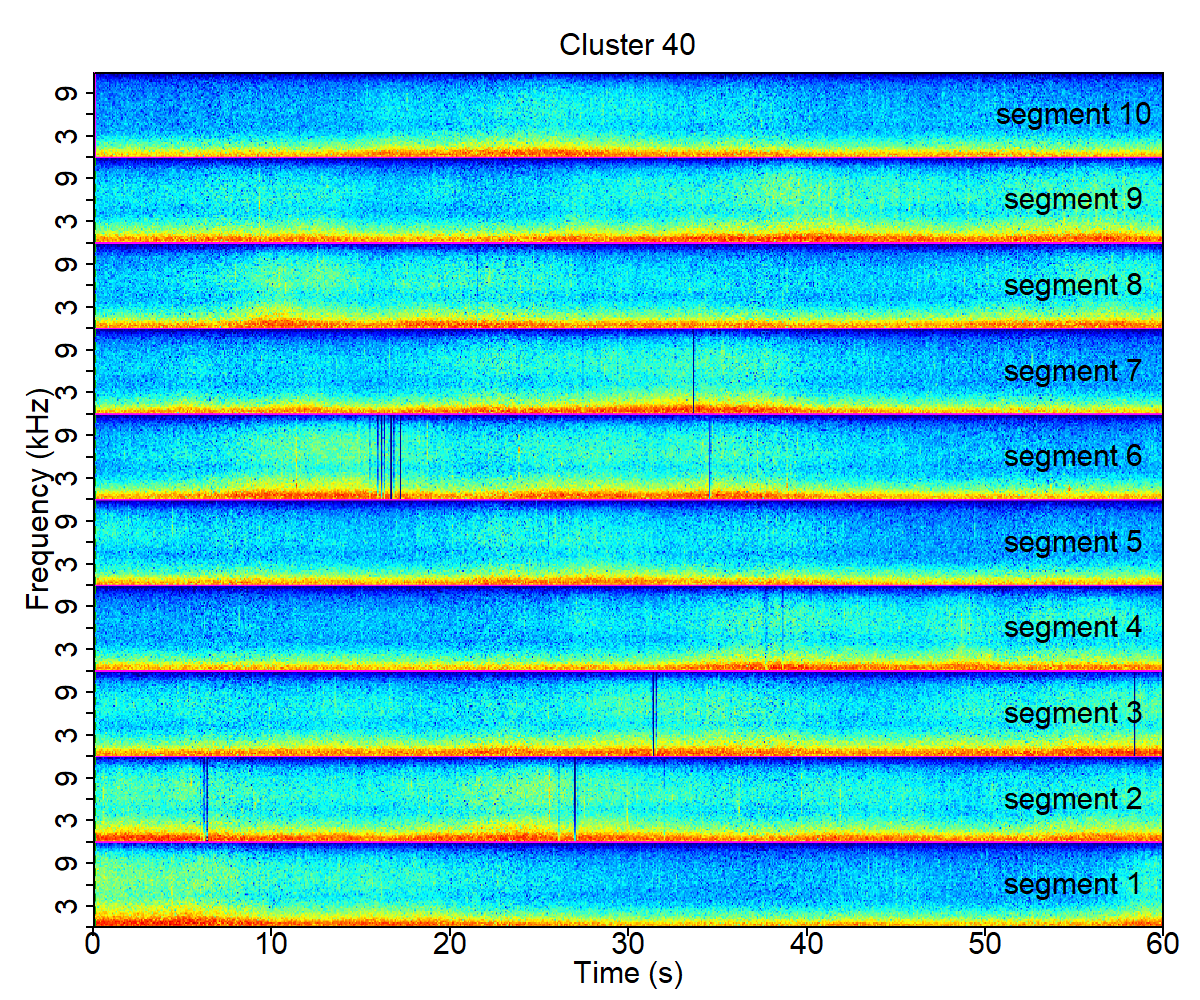

Supplement: Supplemental Information 5 — The spectrograms were computed using a Hann window, FFT = 512, window overlap of 50%, and frame size of 100%. The X-axis represents time, the Y-axis represents frequency. There are 10 audio segments for each cluster. [file peerj-11-16462-s005.zip › Supplemental_Information_S3_spec95_03/Cluster 40.png]

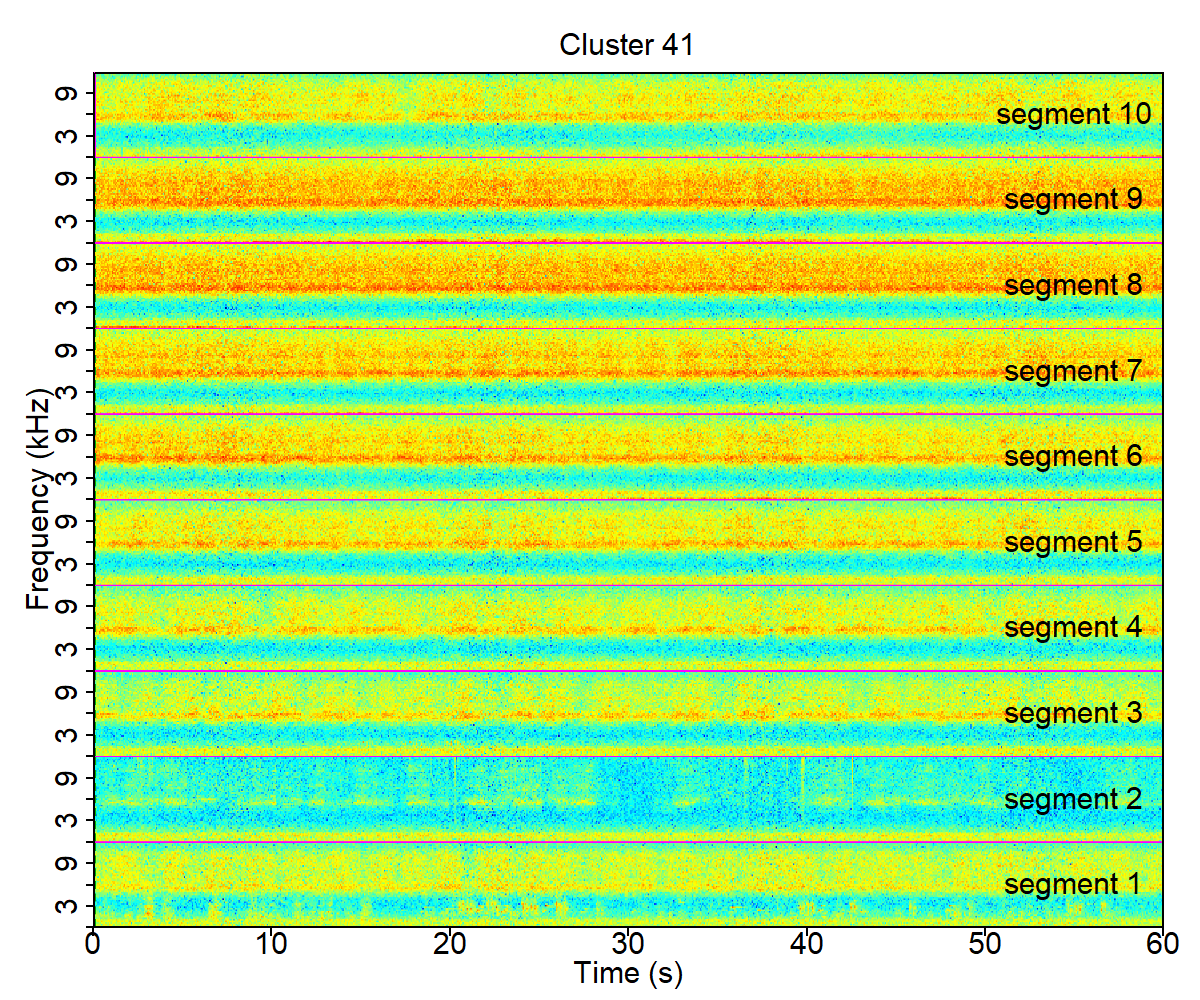

Supplement: Supplemental Information 5 — The spectrograms were computed using a Hann window, FFT = 512, window overlap of 50%, and frame size of 100%. The X-axis represents time, the Y-axis represents frequency. There are 10 audio segments for each cluster. [file peerj-11-16462-s005.zip › Supplemental_Information_S3_spec95_03/Cluster 41.png]

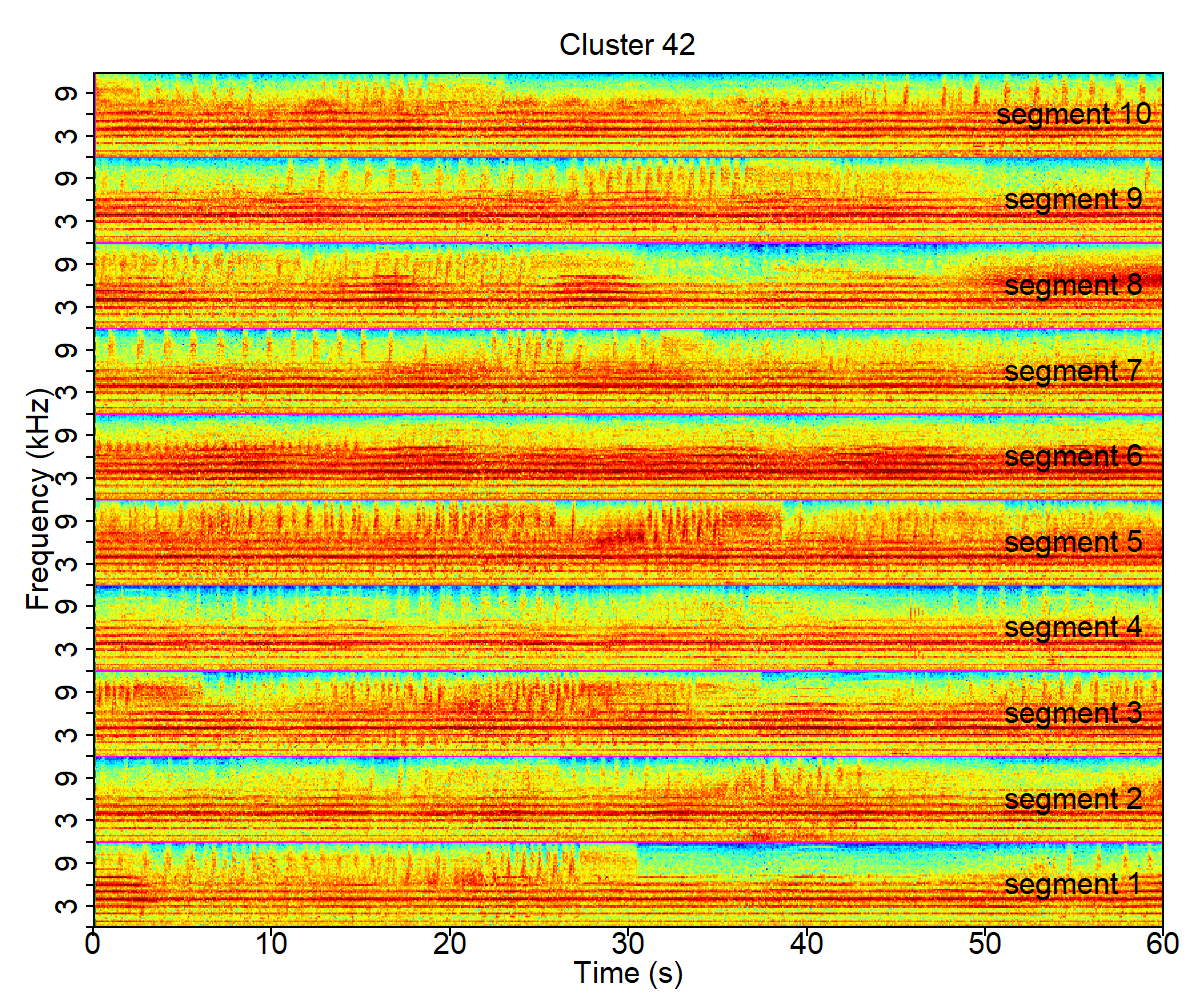

Supplement: Supplemental Information 5 — The spectrograms were computed using a Hann window, FFT = 512, window overlap of 50%, and frame size of 100%. The X-axis represents time, the Y-axis represents frequency. There are 10 audio segments for each cluster. [file peerj-11-16462-s005.zip › Supplemental_Information_S3_spec95_03/Cluster 42.png]

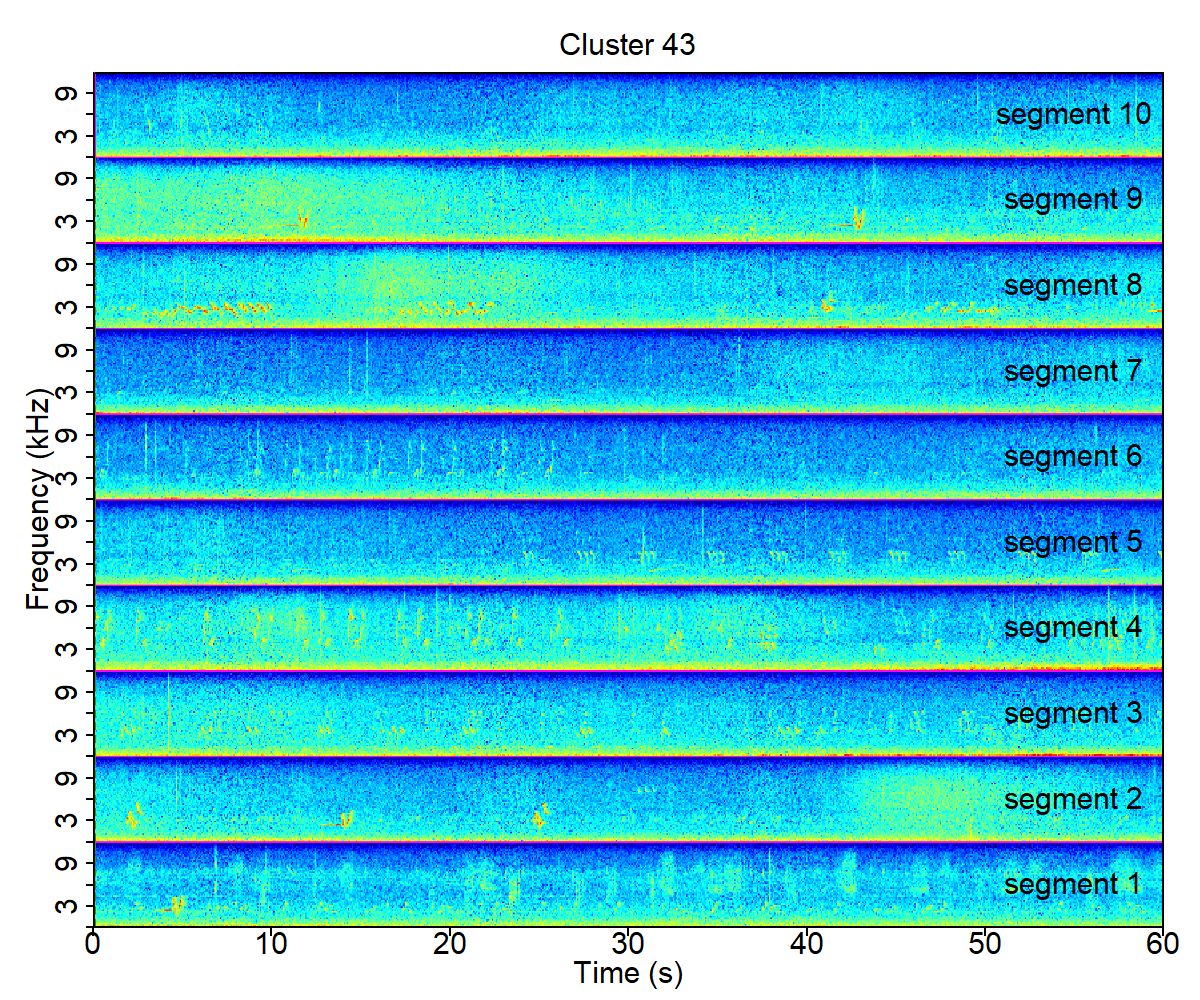

Supplement: Supplemental Information 5 — The spectrograms were computed using a Hann window, FFT = 512, window overlap of 50%, and frame size of 100%. The X-axis represents time, the Y-axis represents frequency. There are 10 audio segments for each cluster. [file peerj-11-16462-s005.zip › Supplemental_Information_S3_spec95_03/Cluster 43.png]

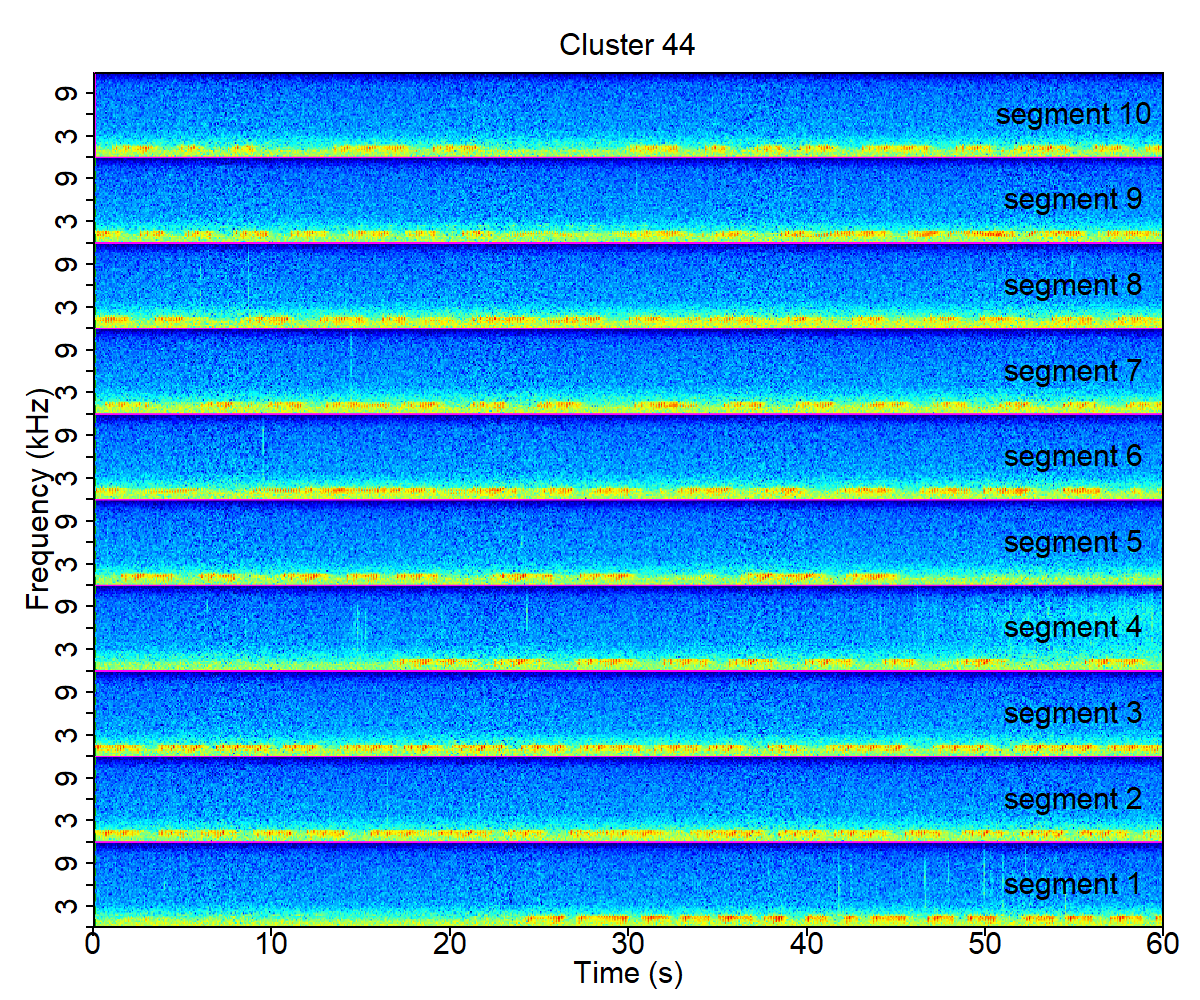

Supplement: Supplemental Information 5 — The spectrograms were computed using a Hann window, FFT = 512, window overlap of 50%, and frame size of 100%. The X-axis represents time, the Y-axis represents frequency. There are 10 audio segments for each cluster. [file peerj-11-16462-s005.zip › Supplemental_Information_S3_spec95_03/Cluster 44.png]

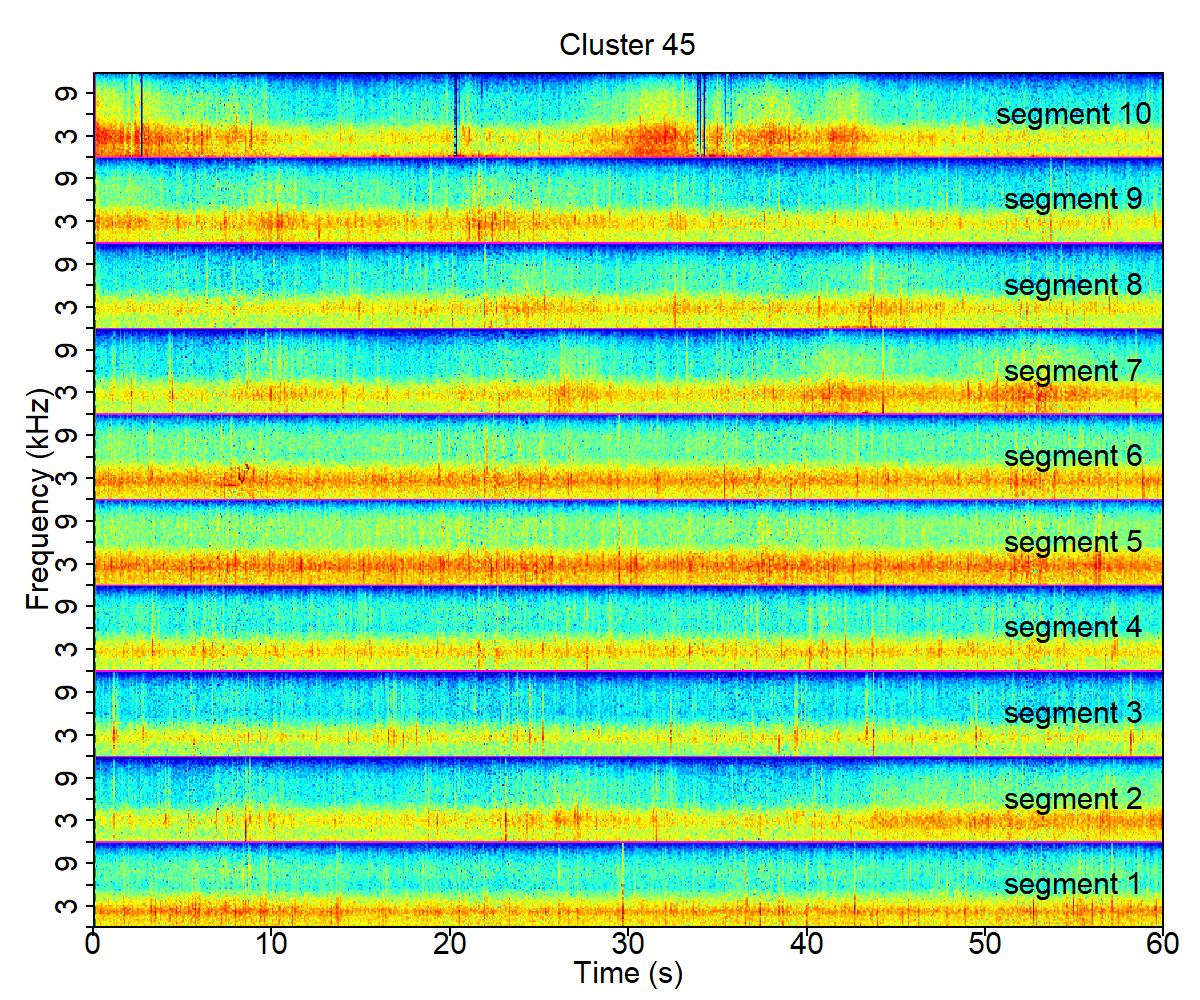

Supplement: Supplemental Information 5 — The spectrograms were computed using a Hann window, FFT = 512, window overlap of 50%, and frame size of 100%. The X-axis represents time, the Y-axis represents frequency. There are 10 audio segments for each cluster. [file peerj-11-16462-s005.zip › Supplemental_Information_S3_spec95_03/Cluster 45.png]

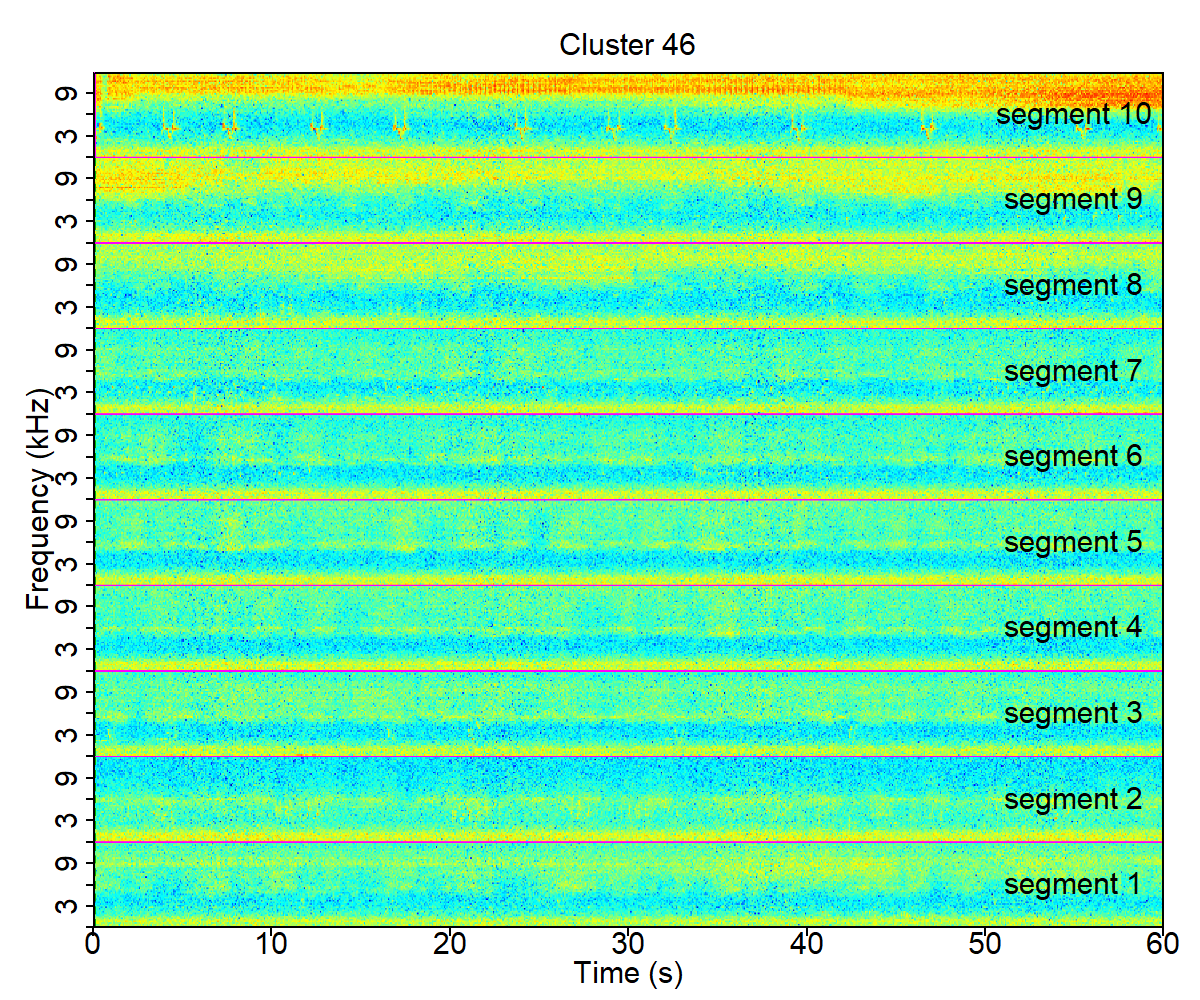

Supplement: Supplemental Information 5 — The spectrograms were computed using a Hann window, FFT = 512, window overlap of 50%, and frame size of 100%. The X-axis represents time, the Y-axis represents frequency. There are 10 audio segments for each cluster. [file peerj-11-16462-s005.zip › Supplemental_Information_S3_spec95_03/Cluster 46.png]

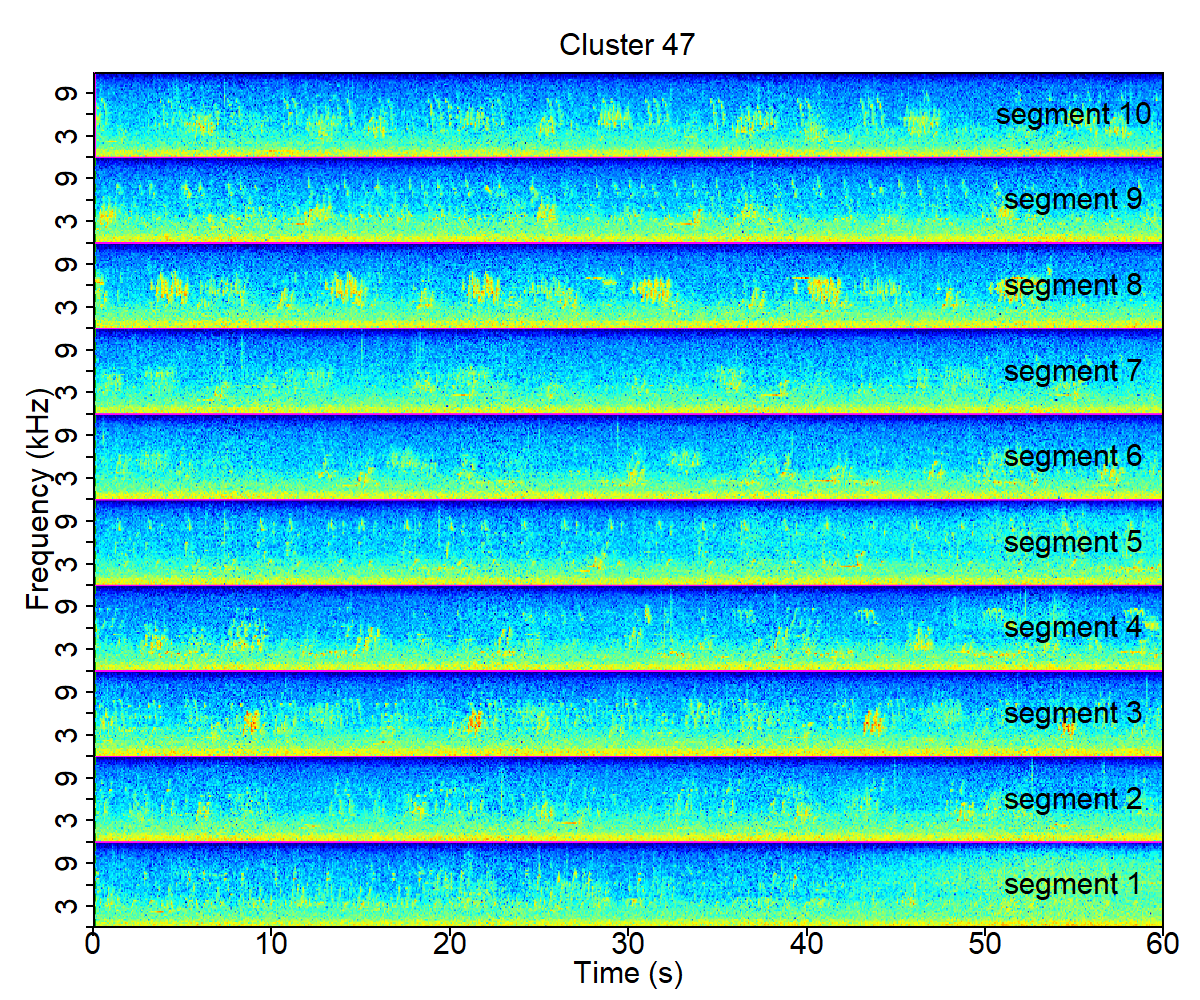

Supplement: Supplemental Information 5 — The spectrograms were computed using a Hann window, FFT = 512, window overlap of 50%, and frame size of 100%. The X-axis represents time, the Y-axis represents frequency. There are 10 audio segments for each cluster. [file peerj-11-16462-s005.zip › Supplemental_Information_S3_spec95_03/Cluster 47.png]

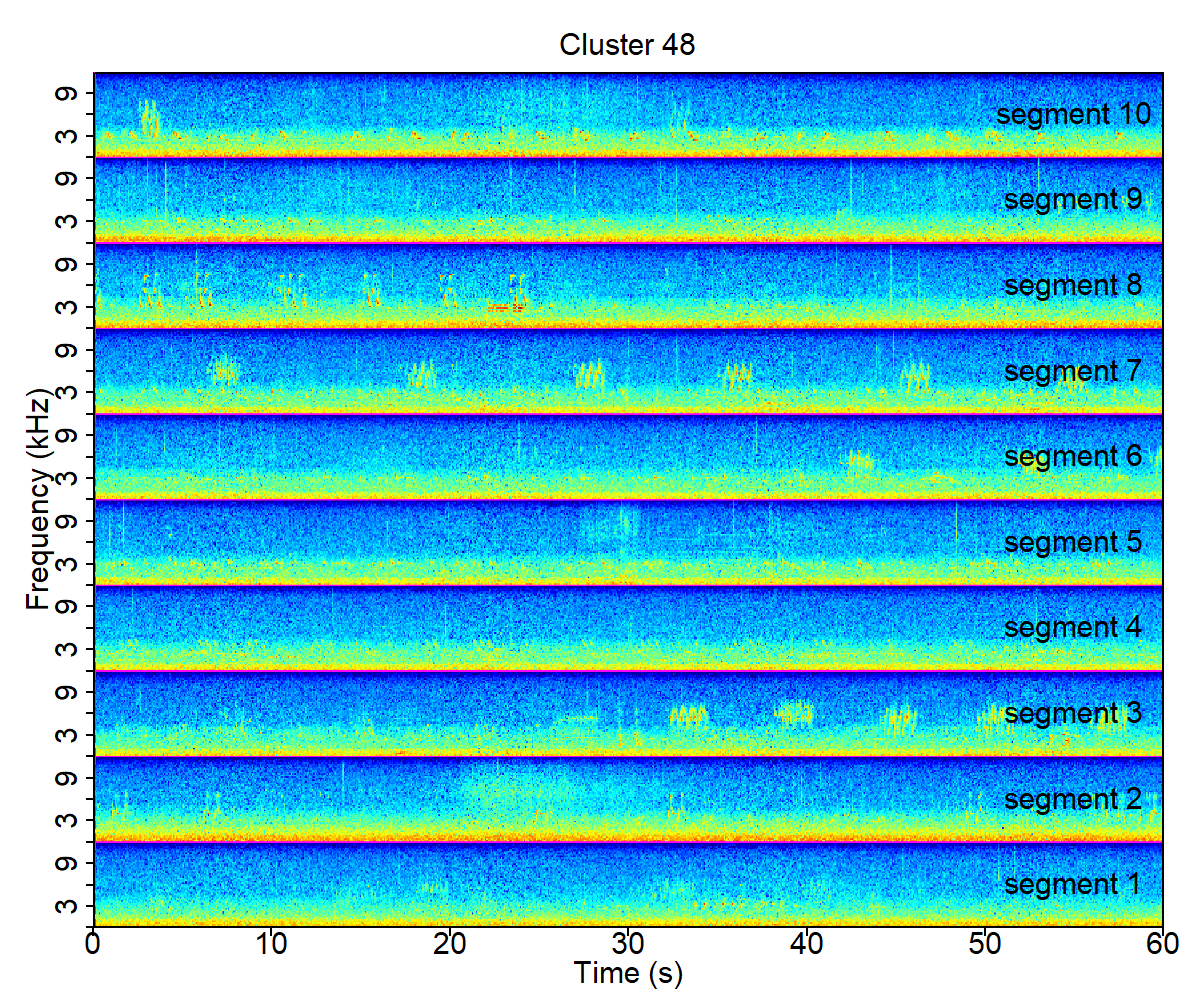

Supplement: Supplemental Information 5 — The spectrograms were computed using a Hann window, FFT = 512, window overlap of 50%, and frame size of 100%. The X-axis represents time, the Y-axis represents frequency. There are 10 audio segments for each cluster. [file peerj-11-16462-s005.zip › Supplemental_Information_S3_spec95_03/Cluster 48.png]

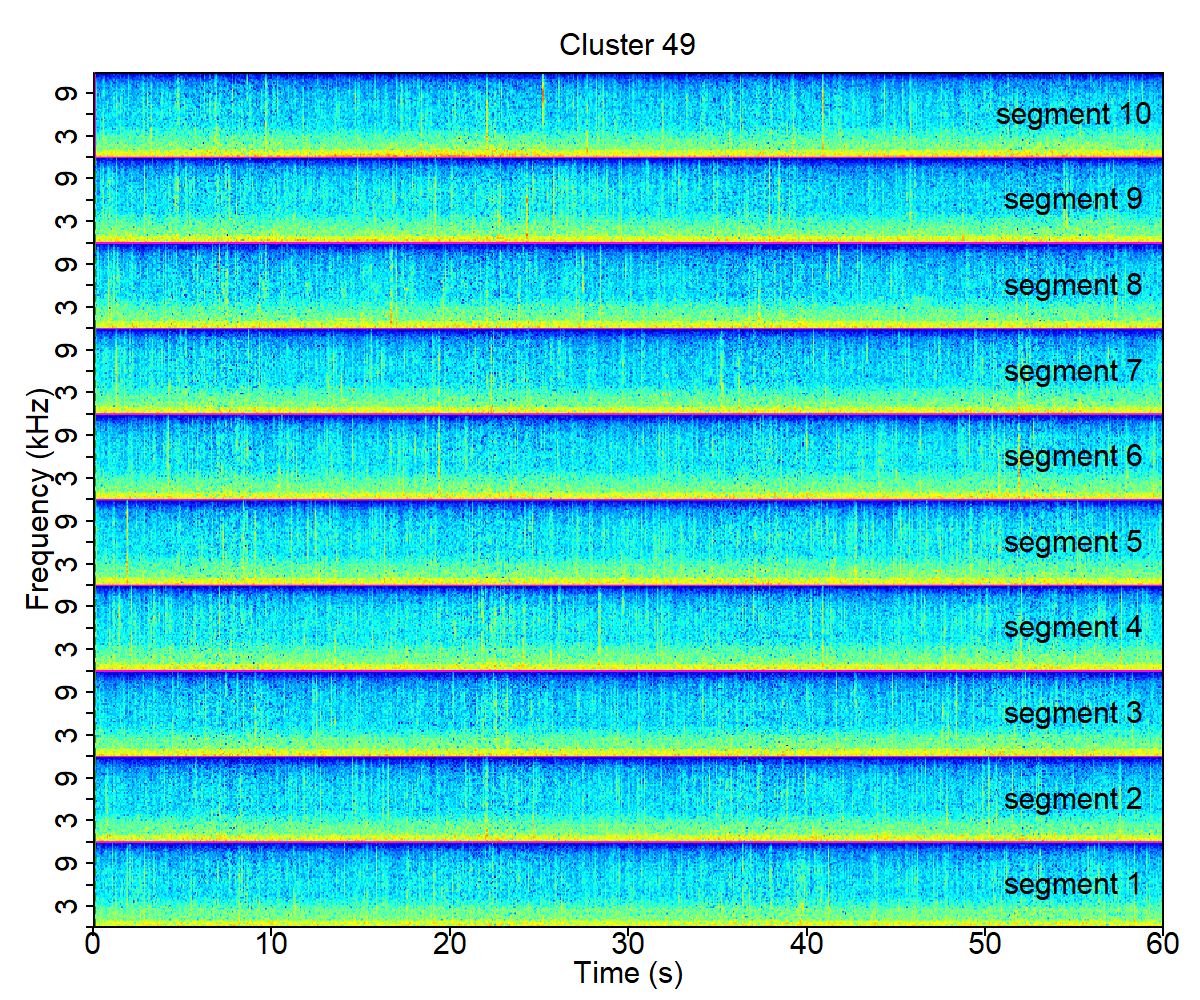

Supplement: Supplemental Information 5 — The spectrograms were computed using a Hann window, FFT = 512, window overlap of 50%, and frame size of 100%. The X-axis represents time, the Y-axis represents frequency. There are 10 audio segments for each cluster. [file peerj-11-16462-s005.zip › Supplemental_Information_S3_spec95_03/Cluster 49.png]

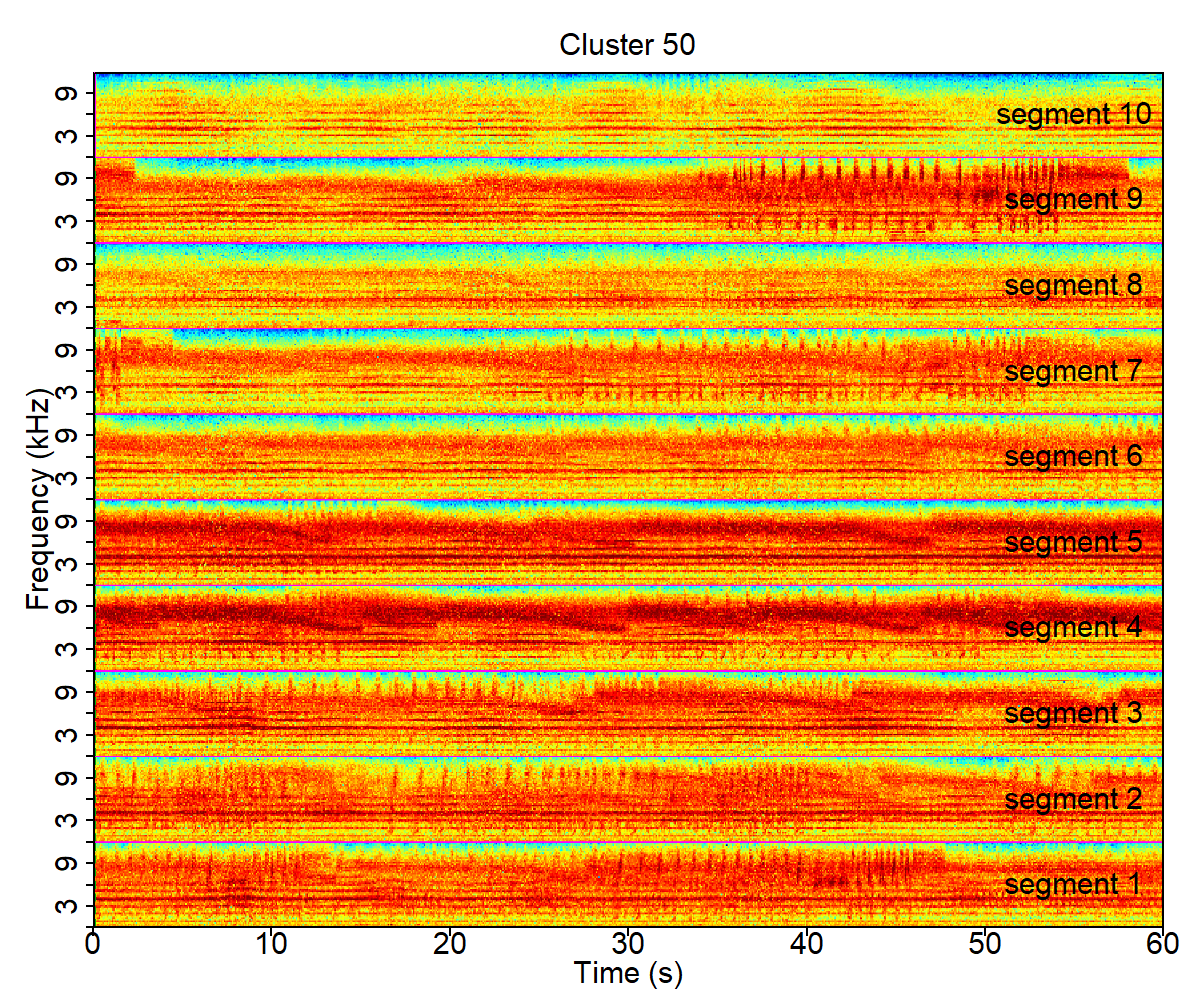

Supplement: Supplemental Information 5 — The spectrograms were computed using a Hann window, FFT = 512, window overlap of 50%, and frame size of 100%. The X-axis represents time, the Y-axis represents frequency. There are 10 audio segments for each cluster. [file peerj-11-16462-s005.zip › Supplemental_Information_S3_spec95_03/Cluster 50.png]

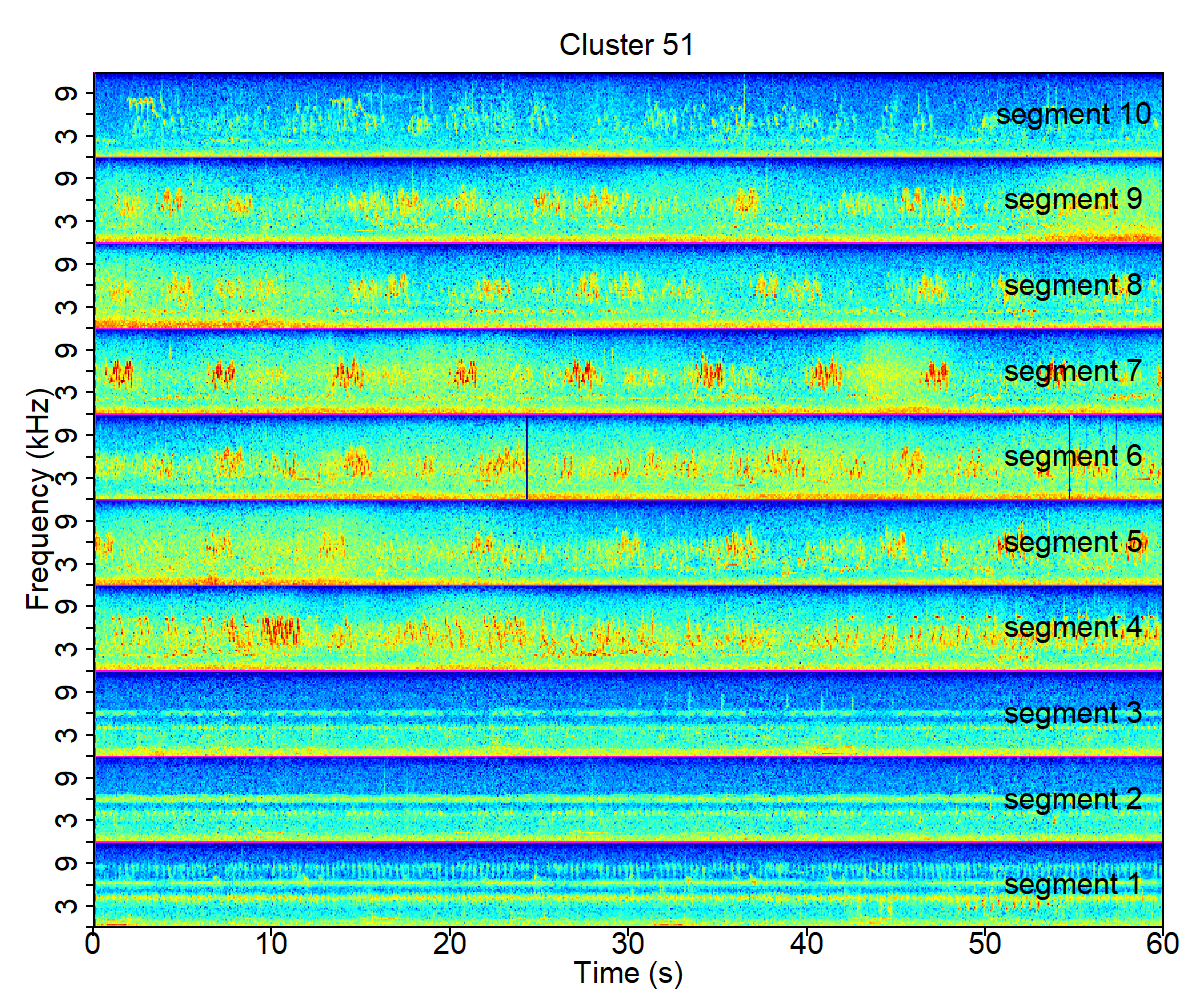

Supplement: Supplemental Information 5 — The spectrograms were computed using a Hann window, FFT = 512, window overlap of 50%, and frame size of 100%. The X-axis represents time, the Y-axis represents frequency. There are 10 audio segments for each cluster. [file peerj-11-16462-s005.zip › Supplemental_Information_S3_spec95_03/Cluster 51.png]

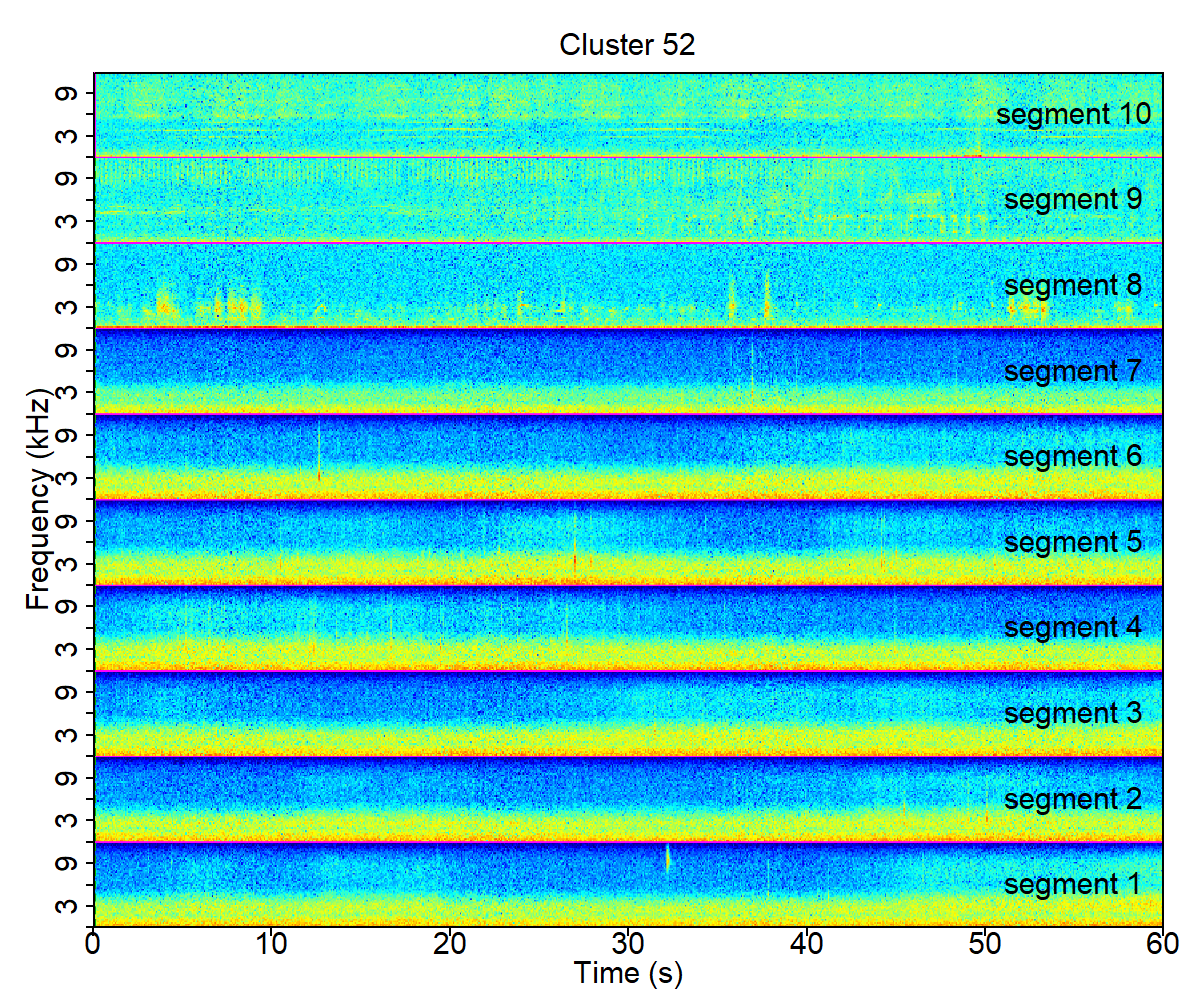

Supplement: Supplemental Information 5 — The spectrograms were computed using a Hann window, FFT = 512, window overlap of 50%, and frame size of 100%. The X-axis represents time, the Y-axis represents frequency. There are 10 audio segments for each cluster. [file peerj-11-16462-s005.zip › Supplemental_Information_S3_spec95_03/Cluster 52.png]

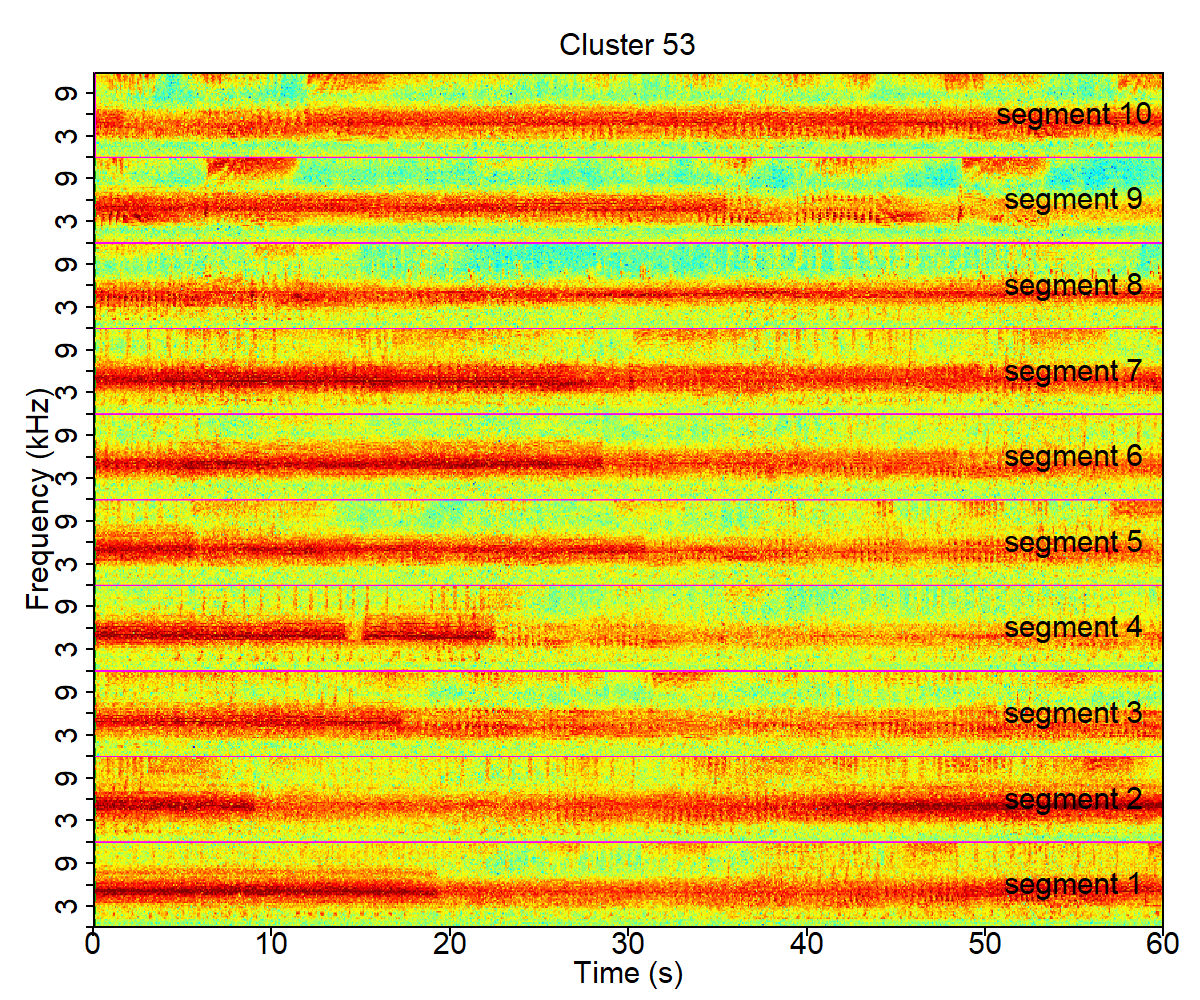

Supplement: Supplemental Information 5 — The spectrograms were computed using a Hann window, FFT = 512, window overlap of 50%, and frame size of 100%. The X-axis represents time, the Y-axis represents frequency. There are 10 audio segments for each cluster. [file peerj-11-16462-s005.zip › Supplemental_Information_S3_spec95_03/Cluster 53.png]

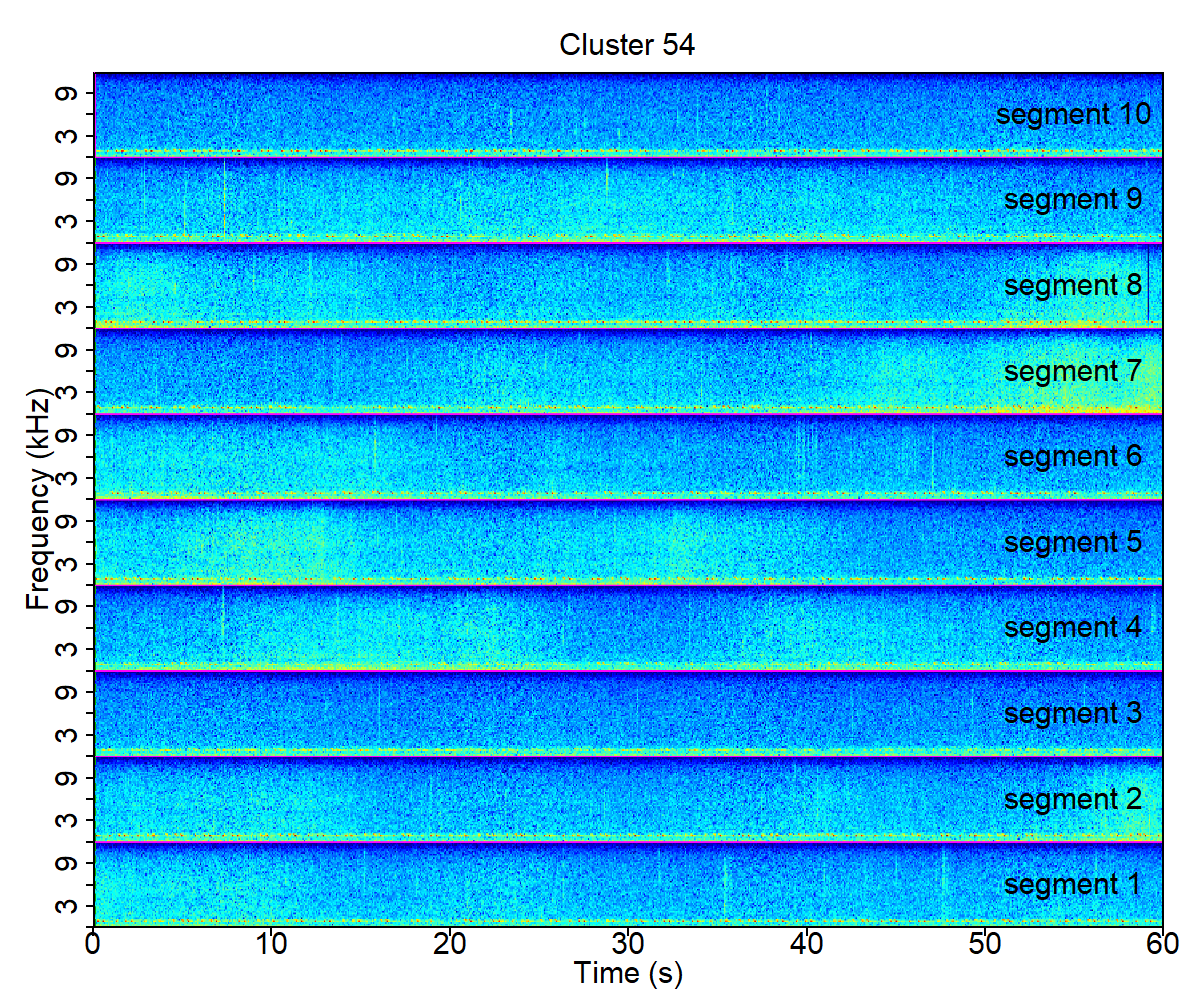

Supplement: Supplemental Information 5 — The spectrograms were computed using a Hann window, FFT = 512, window overlap of 50%, and frame size of 100%. The X-axis represents time, the Y-axis represents frequency. There are 10 audio segments for each cluster. [file peerj-11-16462-s005.zip › Supplemental_Information_S3_spec95_03/Cluster 54.png]

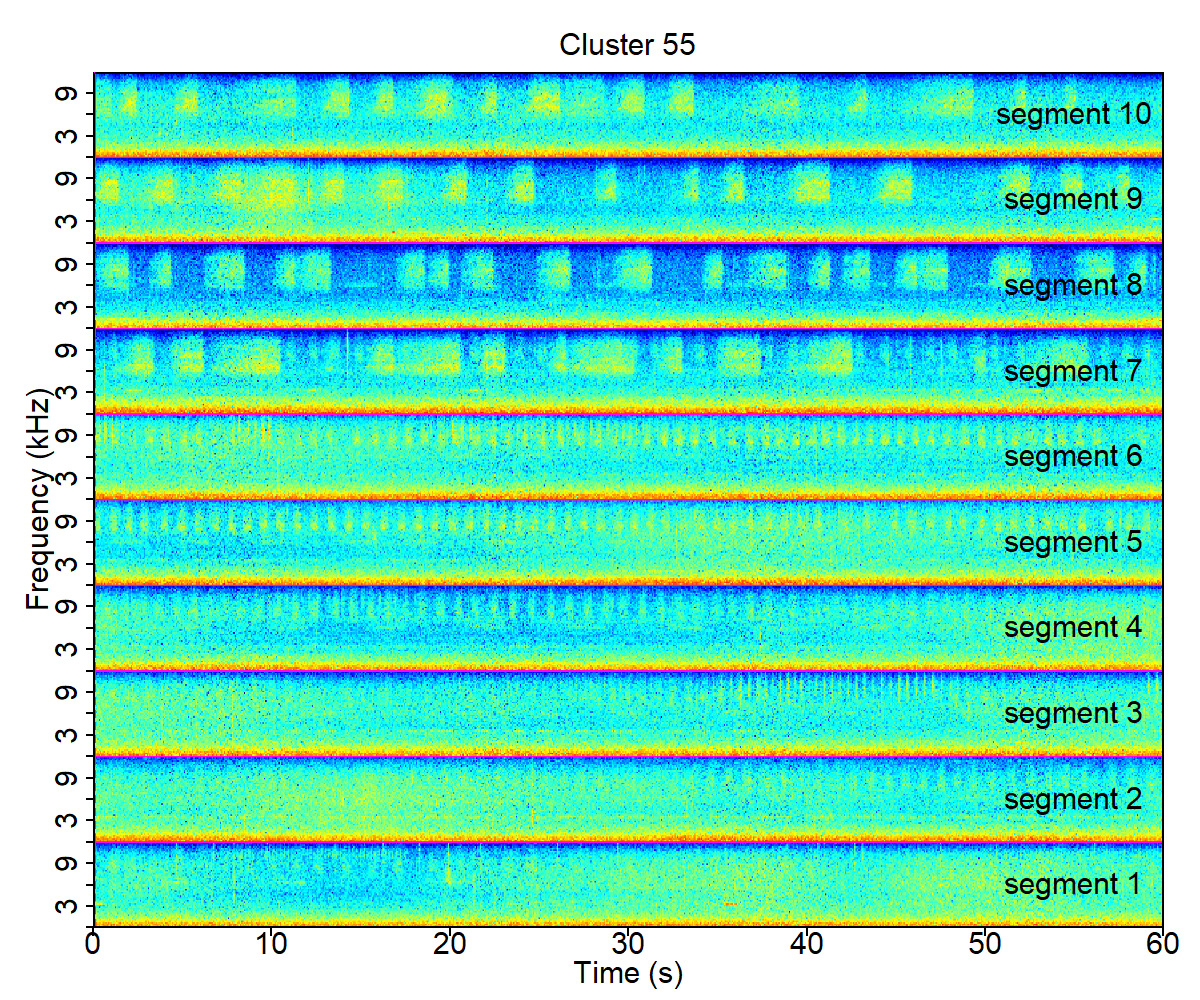

Supplement: Supplemental Information 5 — The spectrograms were computed using a Hann window, FFT = 512, window overlap of 50%, and frame size of 100%. The X-axis represents time, the Y-axis represents frequency. There are 10 audio segments for each cluster. [file peerj-11-16462-s005.zip › Supplemental_Information_S3_spec95_03/Cluster 55.png]

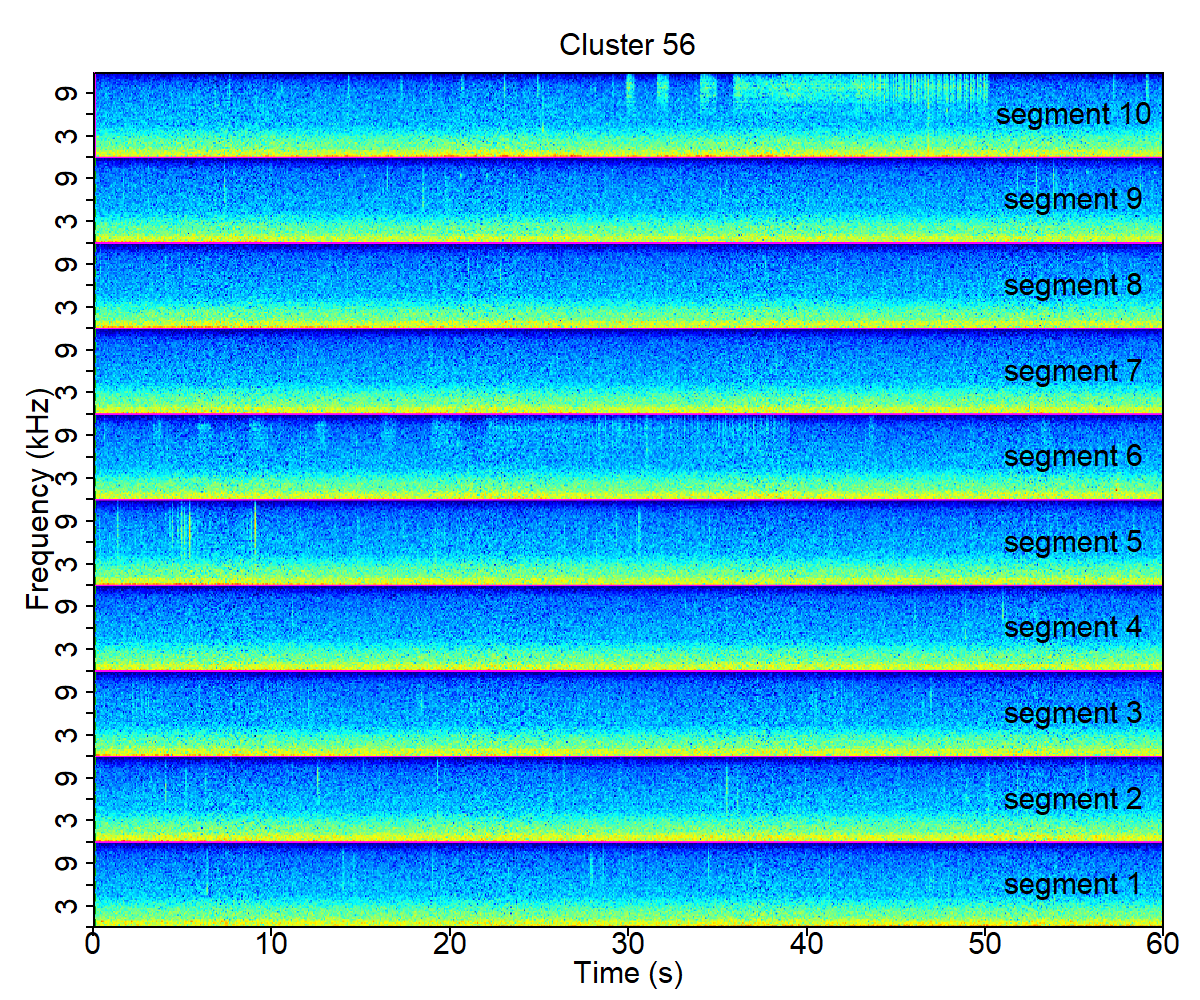

Supplement: Supplemental Information 5 — The spectrograms were computed using a Hann window, FFT = 512, window overlap of 50%, and frame size of 100%. The X-axis represents time, the Y-axis represents frequency. There are 10 audio segments for each cluster. [file peerj-11-16462-s005.zip › Supplemental_Information_S3_spec95_03/Cluster 56.png]

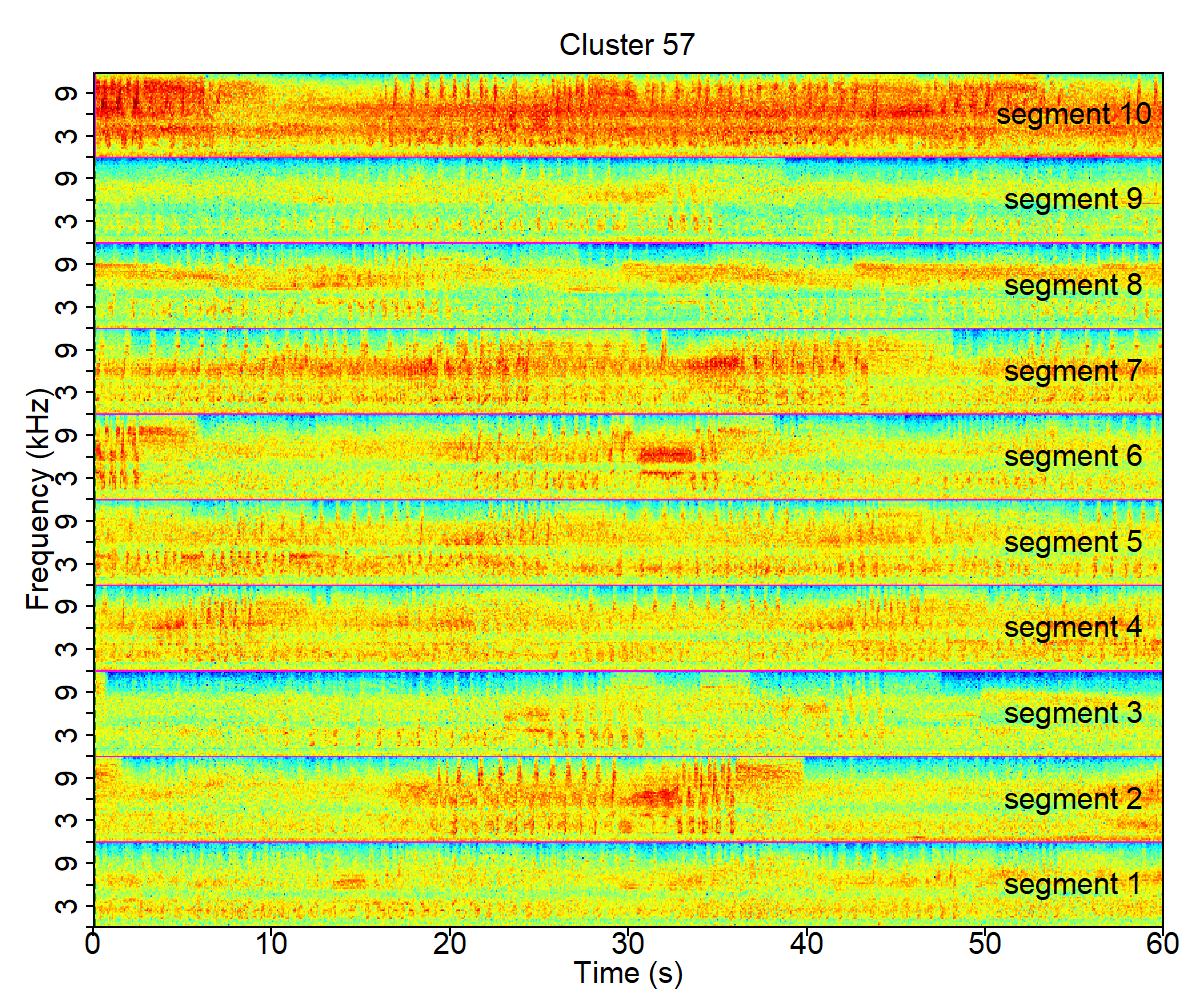

Supplement: Supplemental Information 6 — The spectrograms were computed using a Hann window, FFT = 512, window overlap of 50%, and frame size of 100%. The X-axis represents time, the Y-axis represents frequency. There are 10 audio segments for each cluster. [file peerj-11-16462-s006.zip › Supplemental_Information_S3_spec95_04/Cluster 57.png]

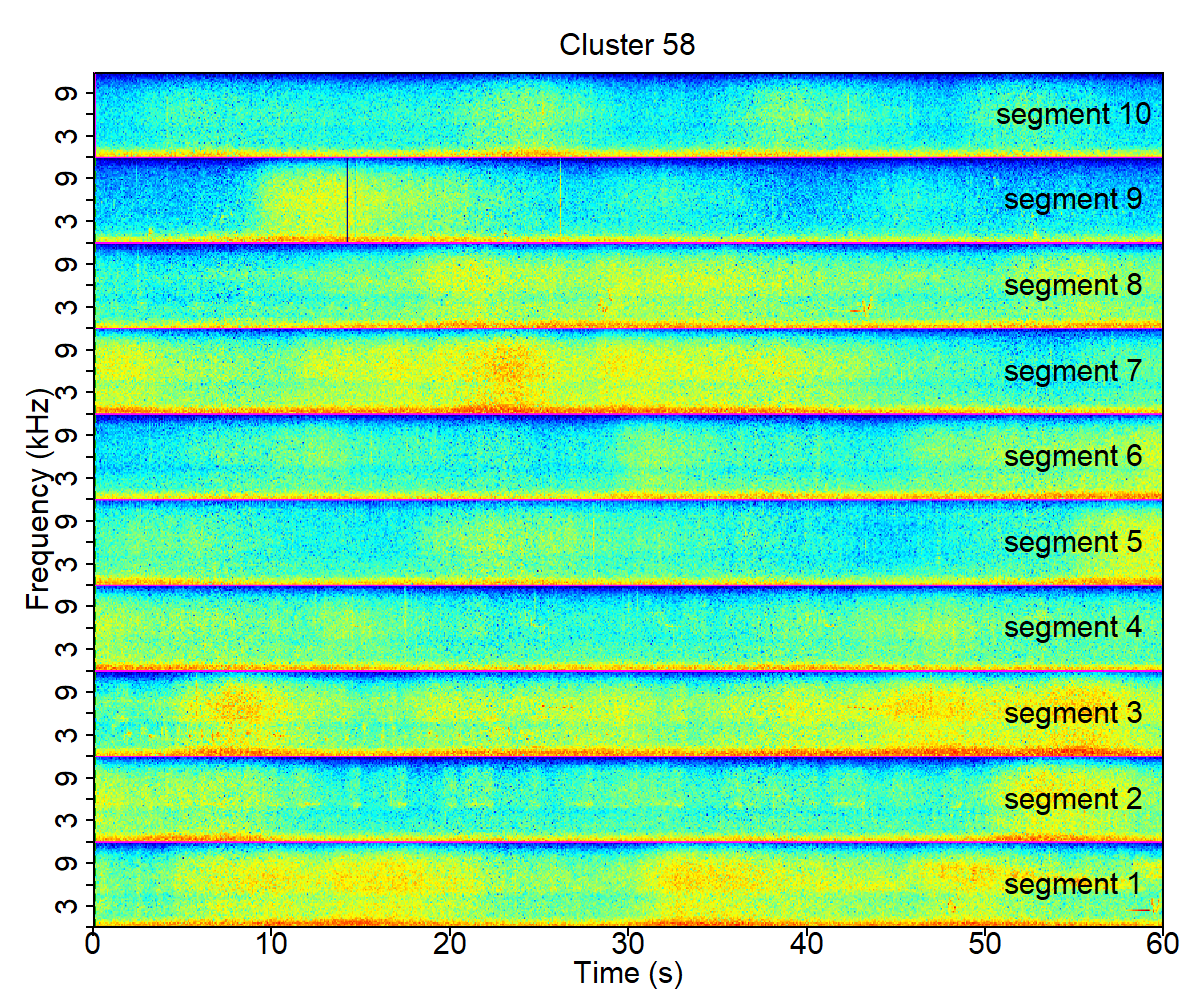

Supplement: Supplemental Information 6 — The spectrograms were computed using a Hann window, FFT = 512, window overlap of 50%, and frame size of 100%. The X-axis represents time, the Y-axis represents frequency. There are 10 audio segments for each cluster. [file peerj-11-16462-s006.zip › Supplemental_Information_S3_spec95_04/Cluster 58.png]

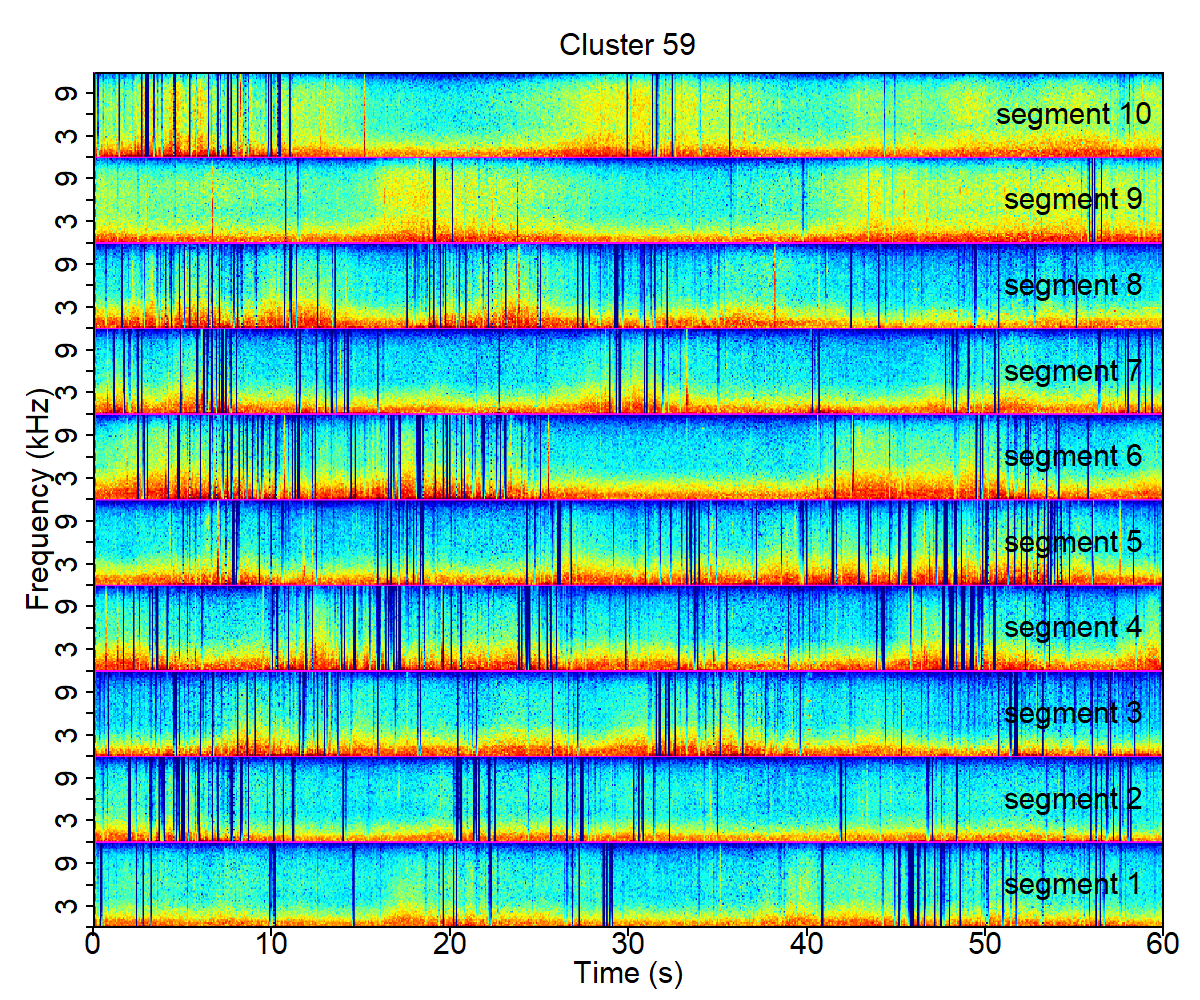

Supplement: Supplemental Information 6 — The spectrograms were computed using a Hann window, FFT = 512, window overlap of 50%, and frame size of 100%. The X-axis represents time, the Y-axis represents frequency. There are 10 audio segments for each cluster. [file peerj-11-16462-s006.zip › Supplemental_Information_S3_spec95_04/Cluster 59.png]

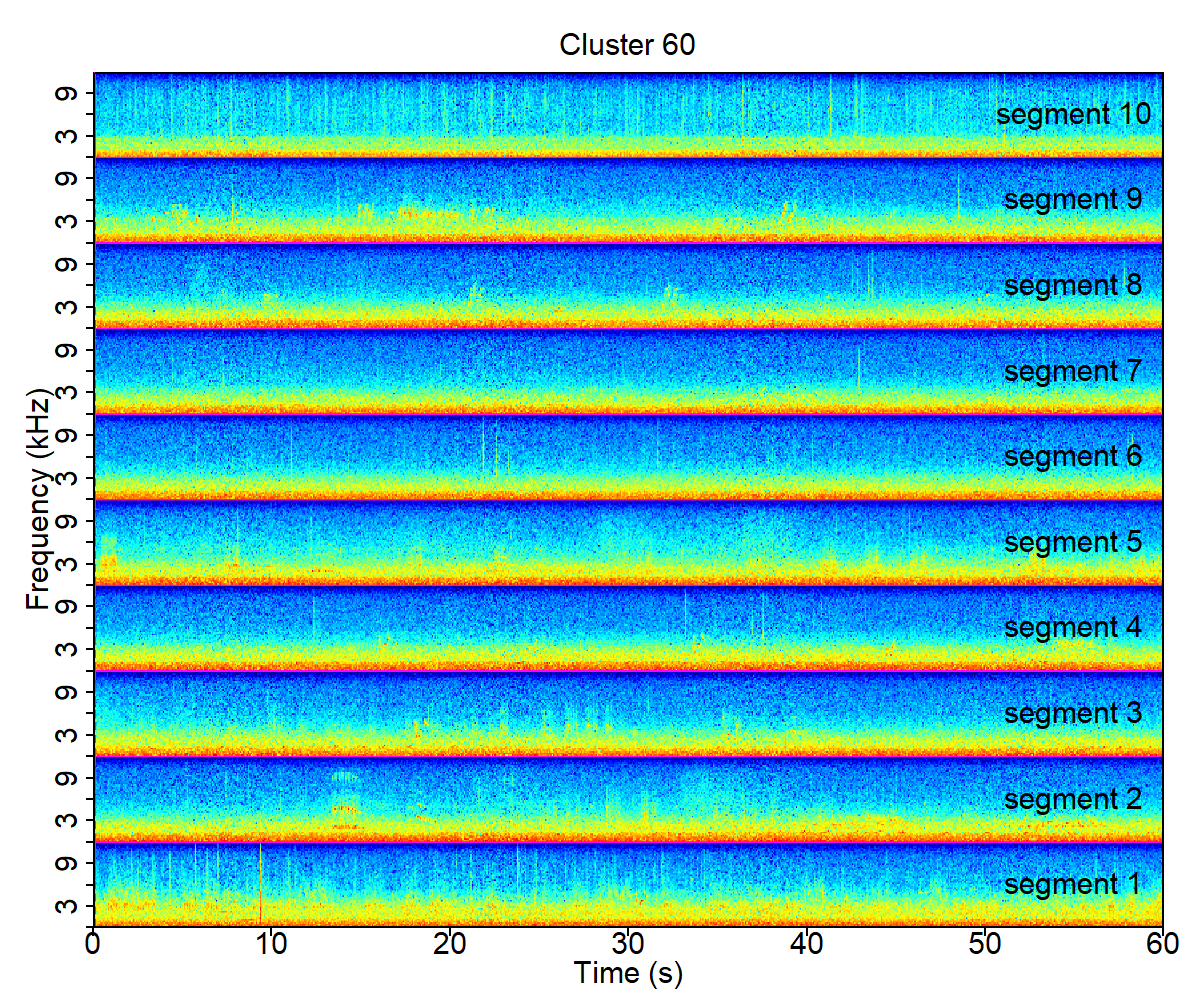

Supplement: Supplemental Information 6 — The spectrograms were computed using a Hann window, FFT = 512, window overlap of 50%, and frame size of 100%. The X-axis represents time, the Y-axis represents frequency. There are 10 audio segments for each cluster. [file peerj-11-16462-s006.zip › Supplemental_Information_S3_spec95_04/Cluster 60.png]

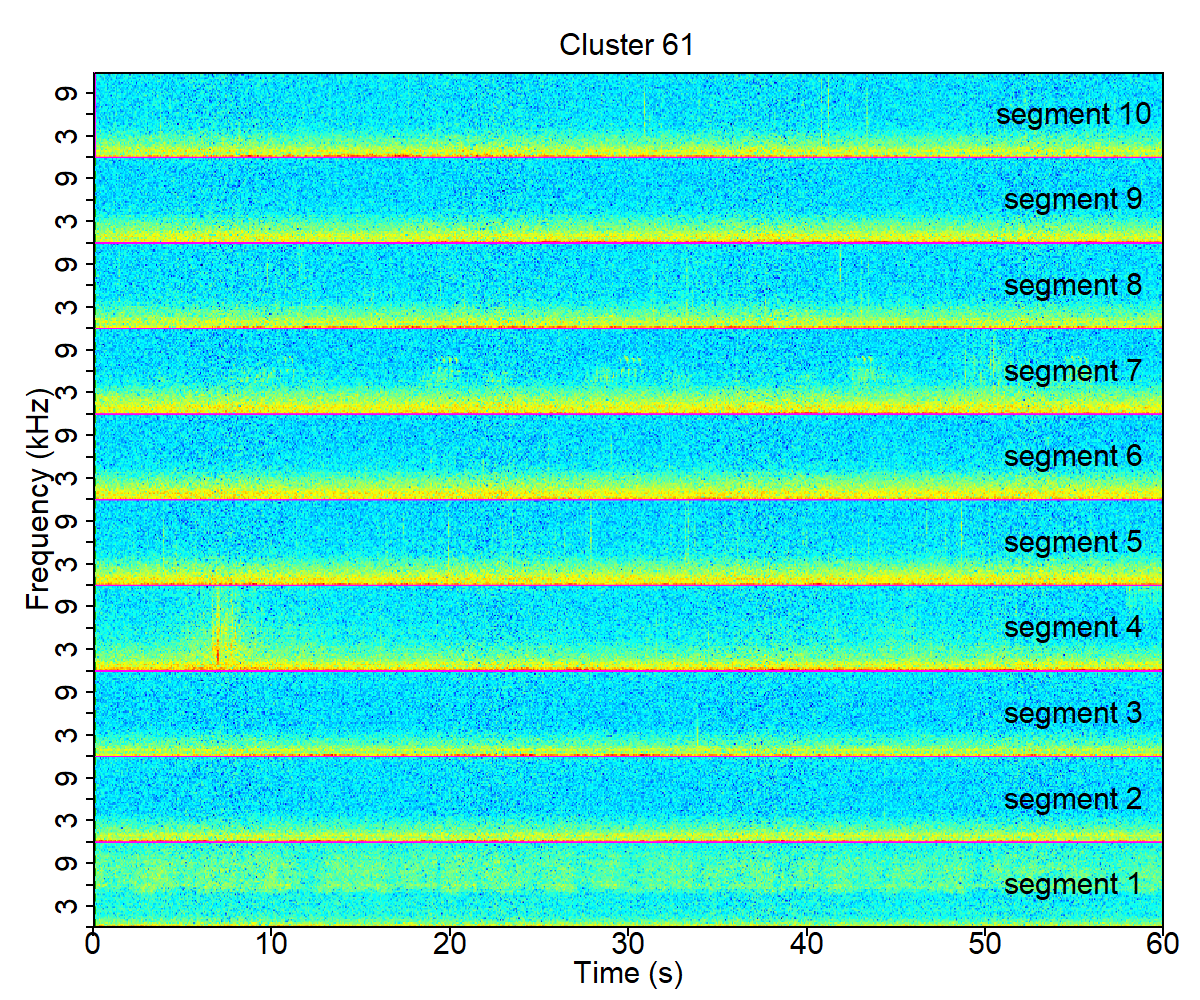

Supplement: Supplemental Information 6 — The spectrograms were computed using a Hann window, FFT = 512, window overlap of 50%, and frame size of 100%. The X-axis represents time, the Y-axis represents frequency. There are 10 audio segments for each cluster. [file peerj-11-16462-s006.zip › Supplemental_Information_S3_spec95_04/Cluster 61.png]

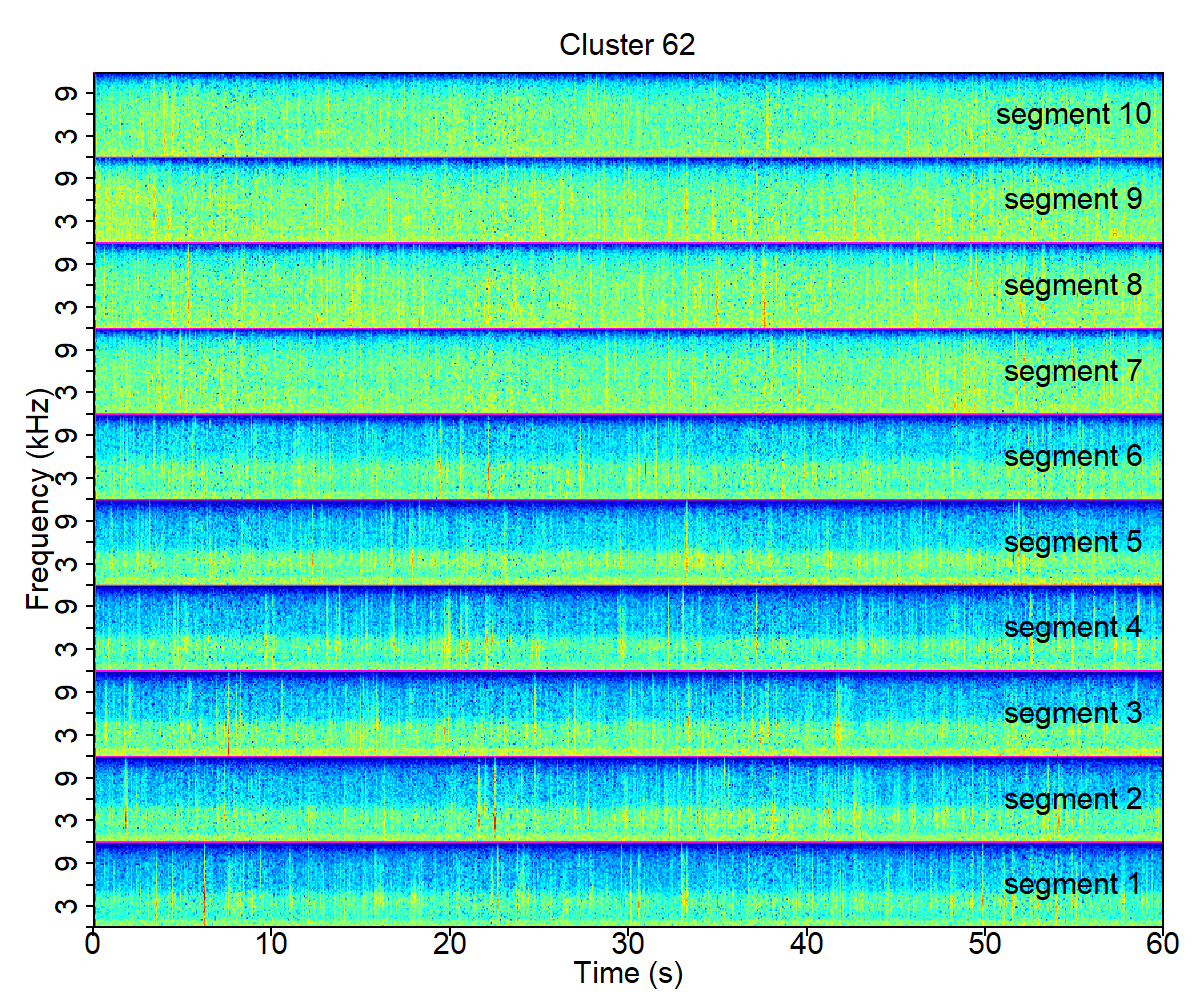

Supplement: Supplemental Information 6 — The spectrograms were computed using a Hann window, FFT = 512, window overlap of 50%, and frame size of 100%. The X-axis represents time, the Y-axis represents frequency. There are 10 audio segments for each cluster. [file peerj-11-16462-s006.zip › Supplemental_Information_S3_spec95_04/Cluster 62.png]

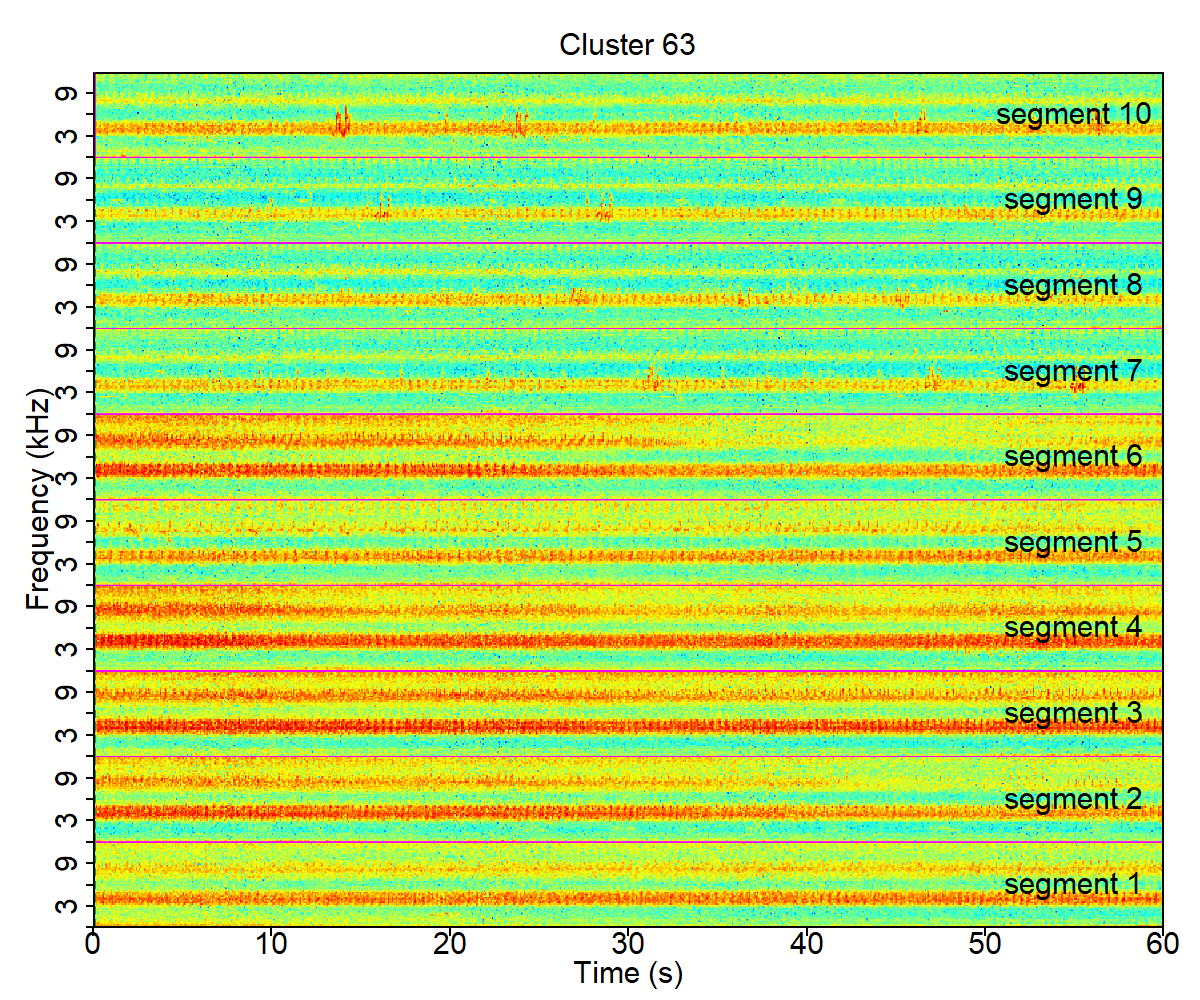

Supplement: Supplemental Information 6 — The spectrograms were computed using a Hann window, FFT = 512, window overlap of 50%, and frame size of 100%. The X-axis represents time, the Y-axis represents frequency. There are 10 audio segments for each cluster. [file peerj-11-16462-s006.zip › Supplemental_Information_S3_spec95_04/Cluster 63.png]

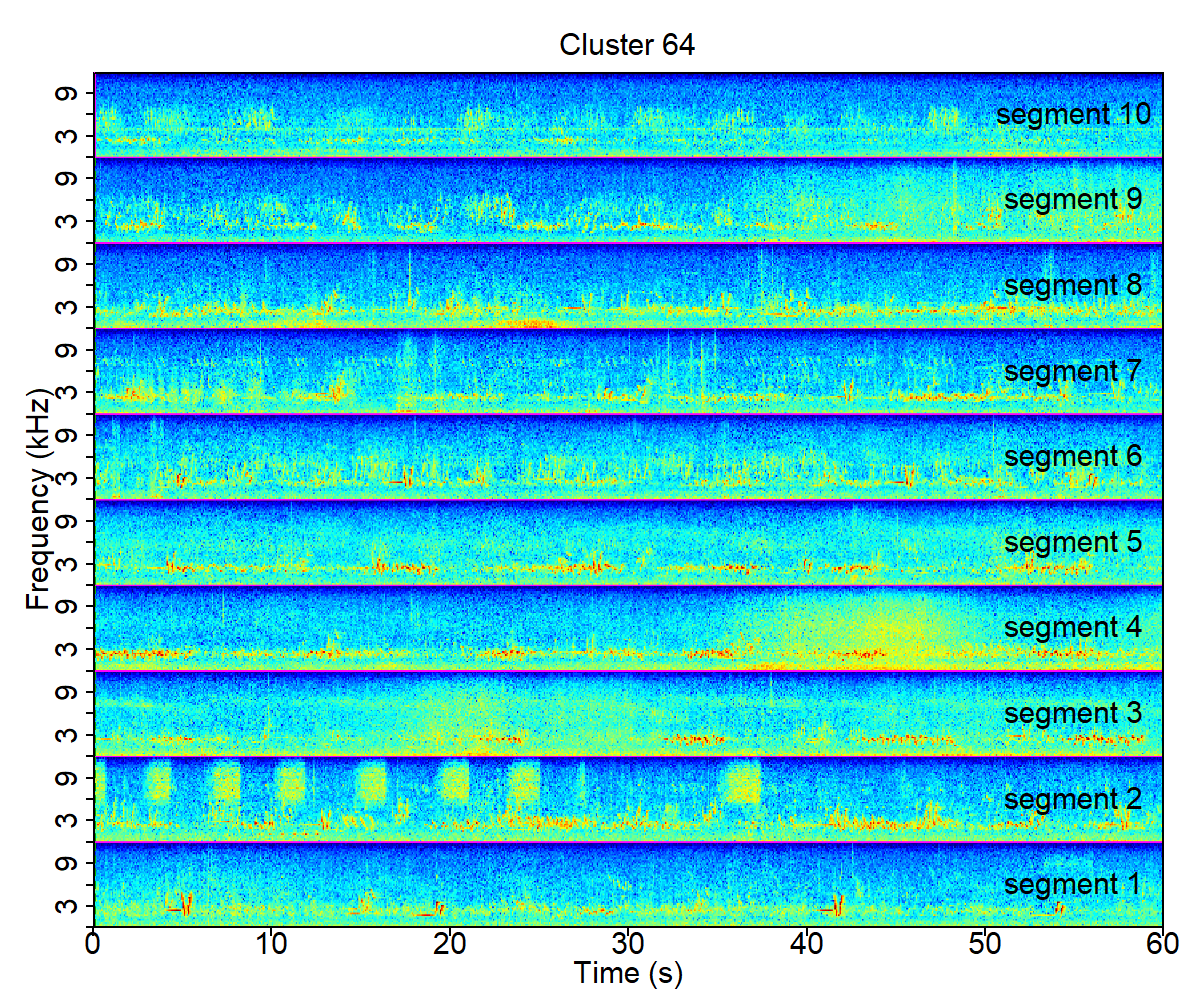

Supplement: Supplemental Information 6 — The spectrograms were computed using a Hann window, FFT = 512, window overlap of 50%, and frame size of 100%. The X-axis represents time, the Y-axis represents frequency. There are 10 audio segments for each cluster. [file peerj-11-16462-s006.zip › Supplemental_Information_S3_spec95_04/Cluster 64.png]

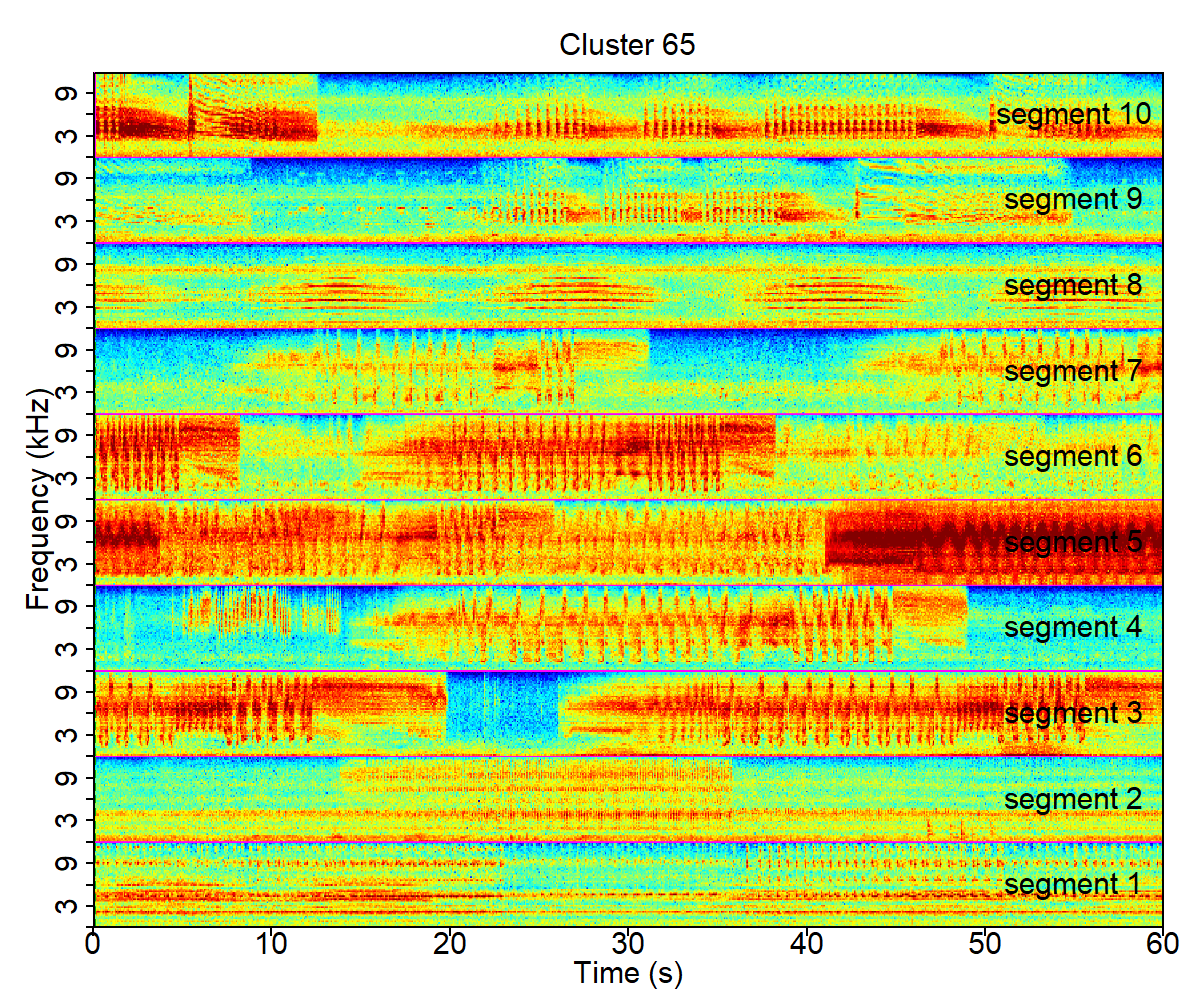

Supplement: Supplemental Information 6 — The spectrograms were computed using a Hann window, FFT = 512, window overlap of 50%, and frame size of 100%. The X-axis represents time, the Y-axis represents frequency. There are 10 audio segments for each cluster. [file peerj-11-16462-s006.zip › Supplemental_Information_S3_spec95_04/Cluster 65.png]

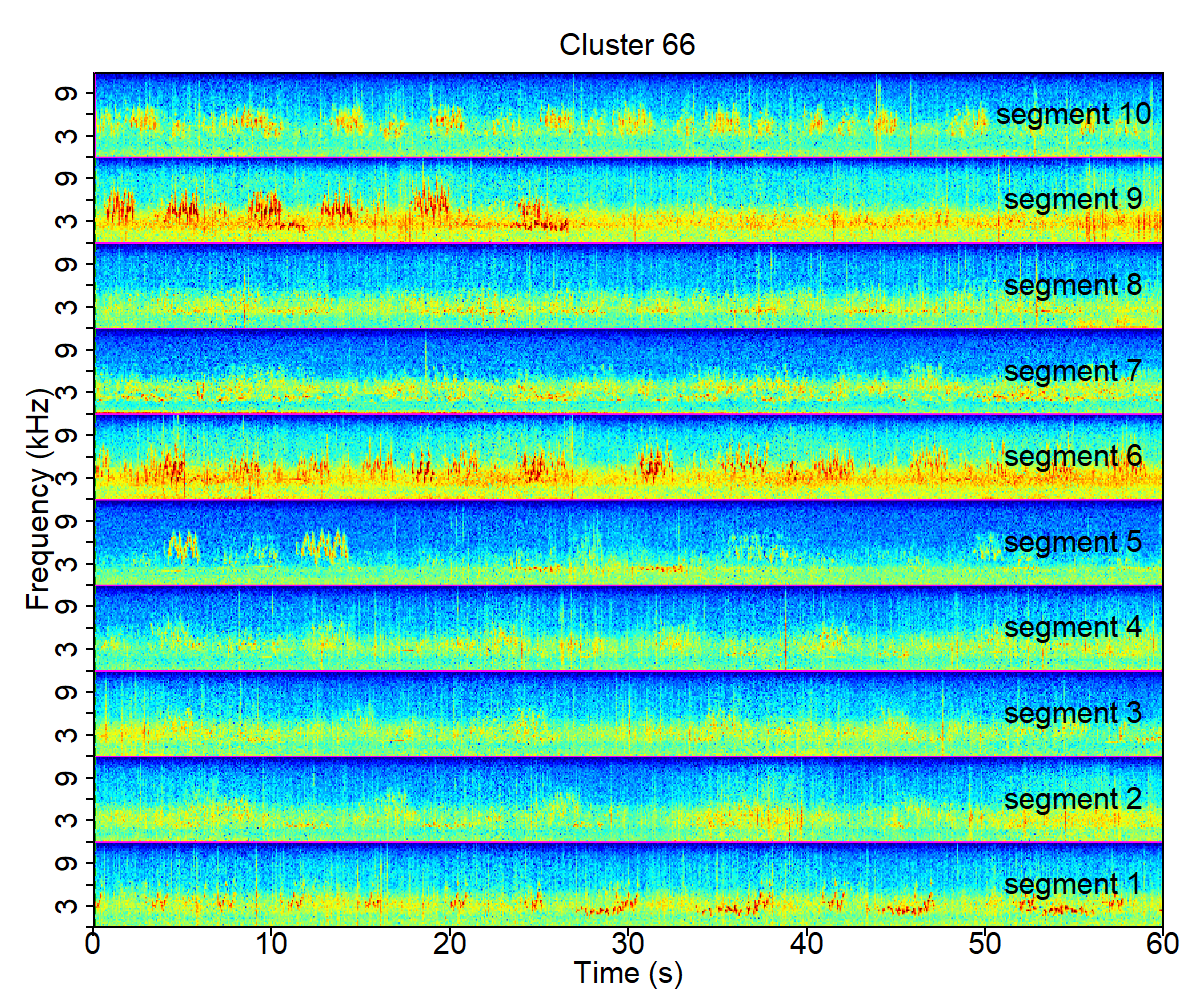

Supplement: Supplemental Information 6 — The spectrograms were computed using a Hann window, FFT = 512, window overlap of 50%, and frame size of 100%. The X-axis represents time, the Y-axis represents frequency. There are 10 audio segments for each cluster. [file peerj-11-16462-s006.zip › Supplemental_Information_S3_spec95_04/Cluster 66.png]

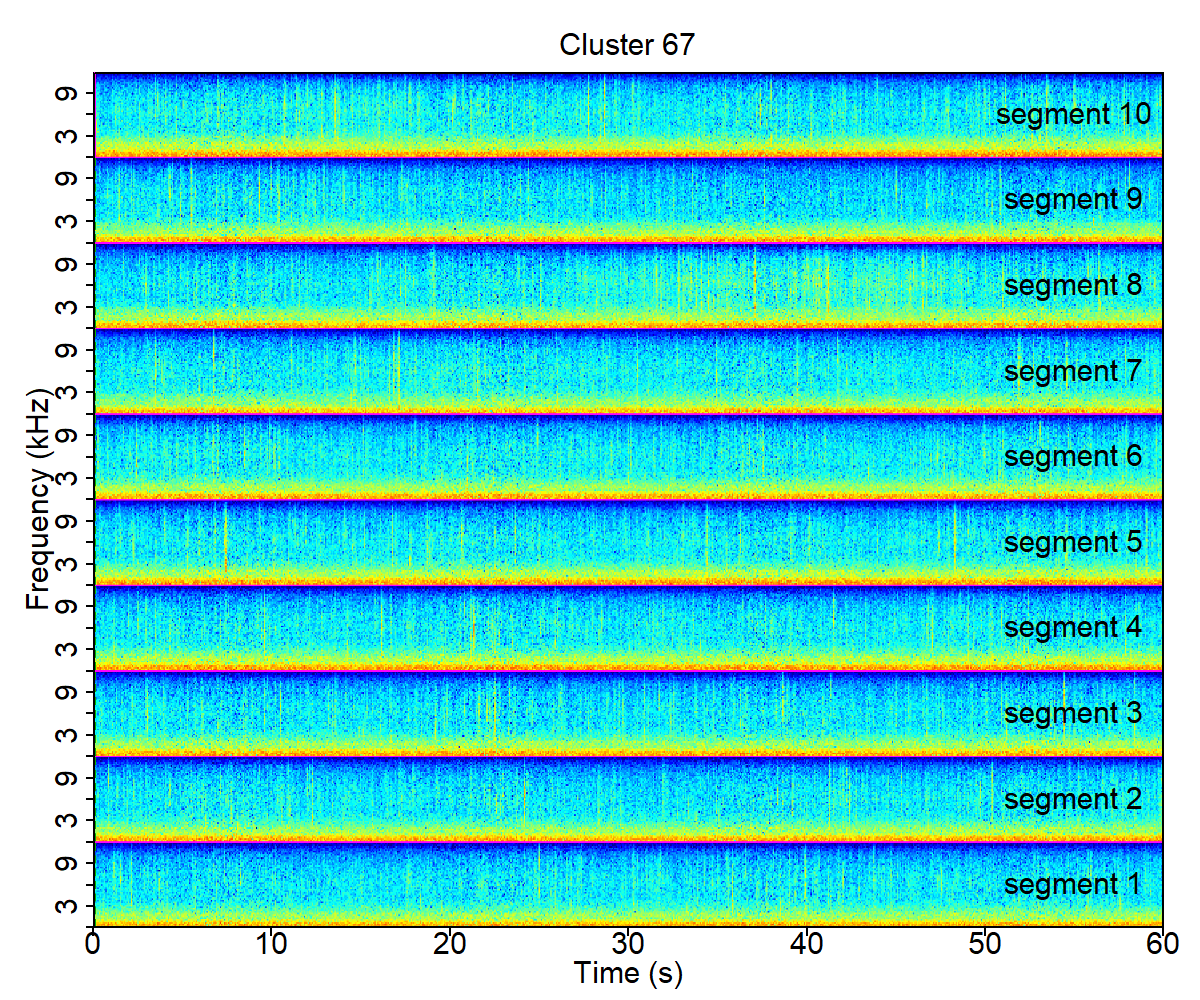

Supplement: Supplemental Information 6 — The spectrograms were computed using a Hann window, FFT = 512, window overlap of 50%, and frame size of 100%. The X-axis represents time, the Y-axis represents frequency. There are 10 audio segments for each cluster. [file peerj-11-16462-s006.zip › Supplemental_Information_S3_spec95_04/Cluster 67.png]

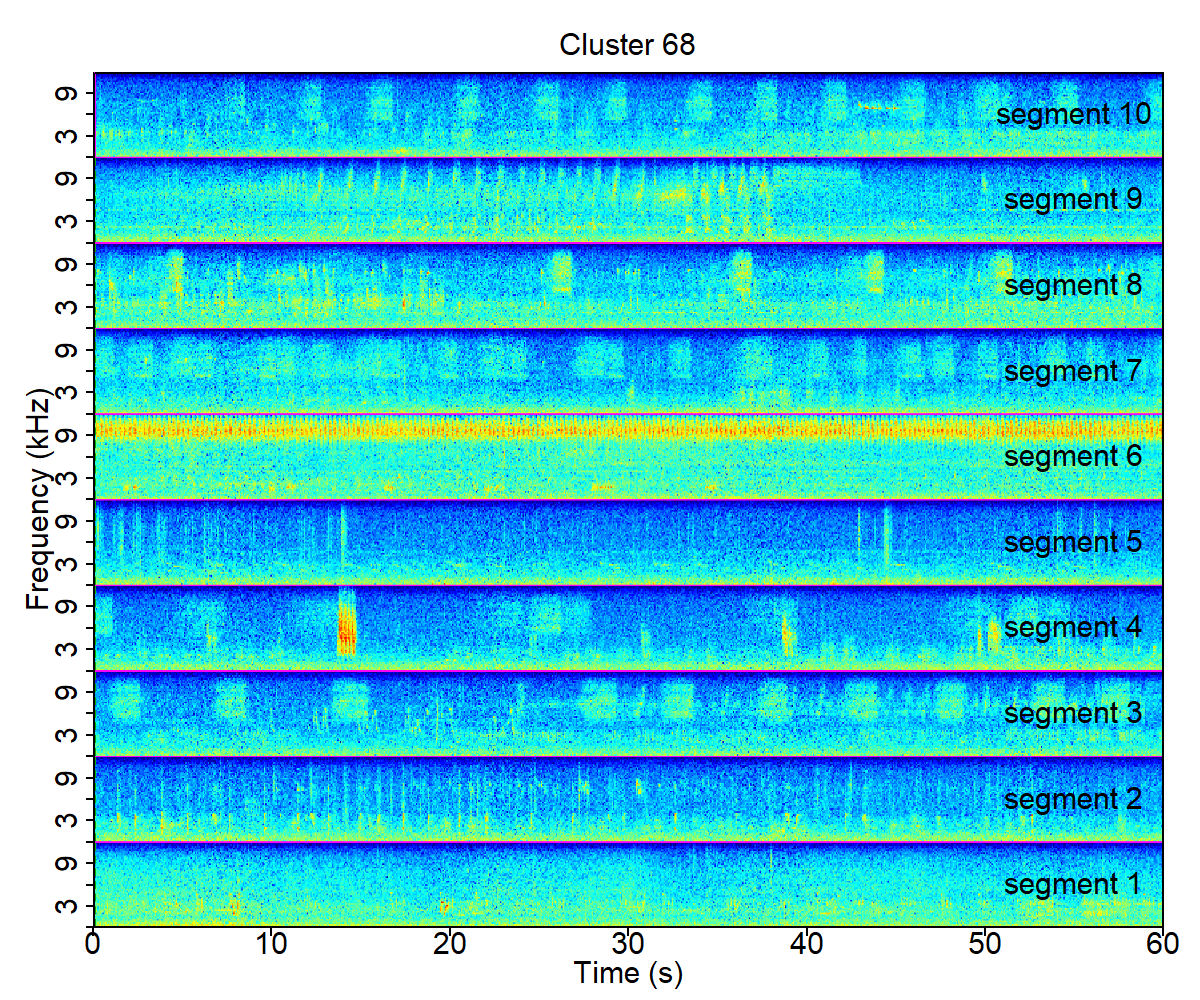

Supplement: Supplemental Information 6 — The spectrograms were computed using a Hann window, FFT = 512, window overlap of 50%, and frame size of 100%. The X-axis represents time, the Y-axis represents frequency. There are 10 audio segments for each cluster. [file peerj-11-16462-s006.zip › Supplemental_Information_S3_spec95_04/Cluster 68.png]

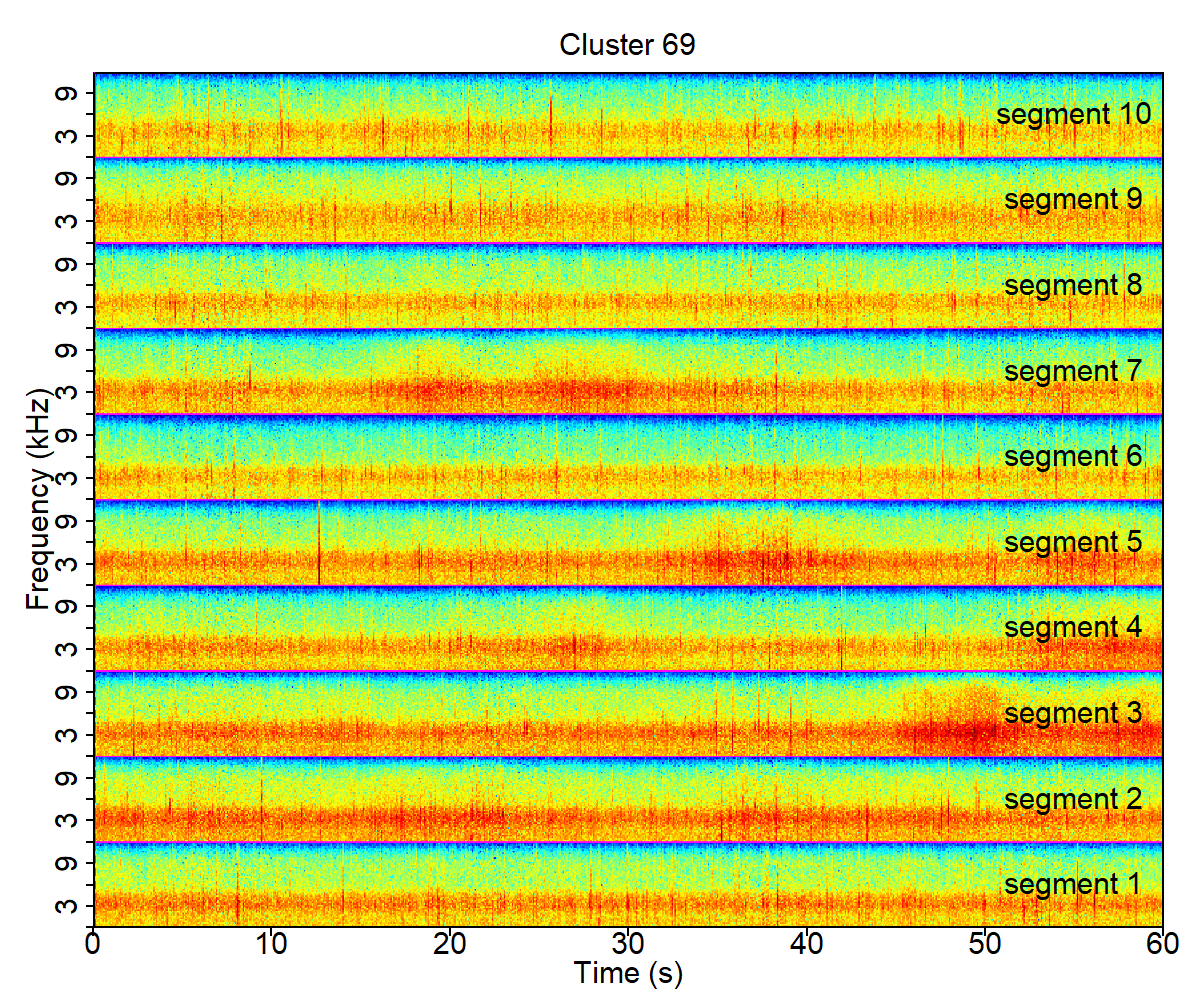

Supplement: Supplemental Information 6 — The spectrograms were computed using a Hann window, FFT = 512, window overlap of 50%, and frame size of 100%. The X-axis represents time, the Y-axis represents frequency. There are 10 audio segments for each cluster. [file peerj-11-16462-s006.zip › Supplemental_Information_S3_spec95_04/Cluster 69.png]

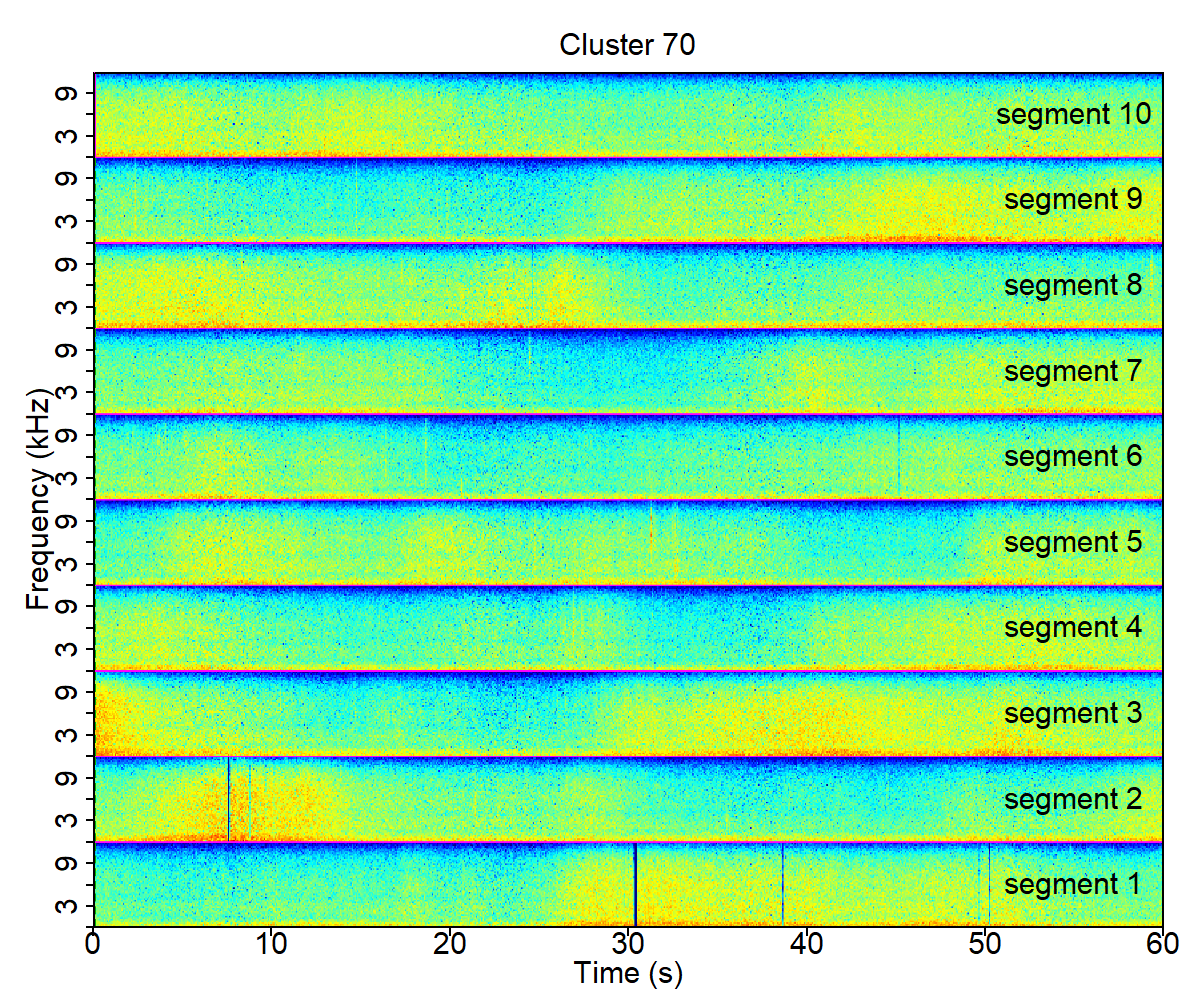

Supplement: Supplemental Information 6 — The spectrograms were computed using a Hann window, FFT = 512, window overlap of 50%, and frame size of 100%. The X-axis represents time, the Y-axis represents frequency. There are 10 audio segments for each cluster. [file peerj-11-16462-s006.zip › Supplemental_Information_S3_spec95_04/Cluster 70.png]

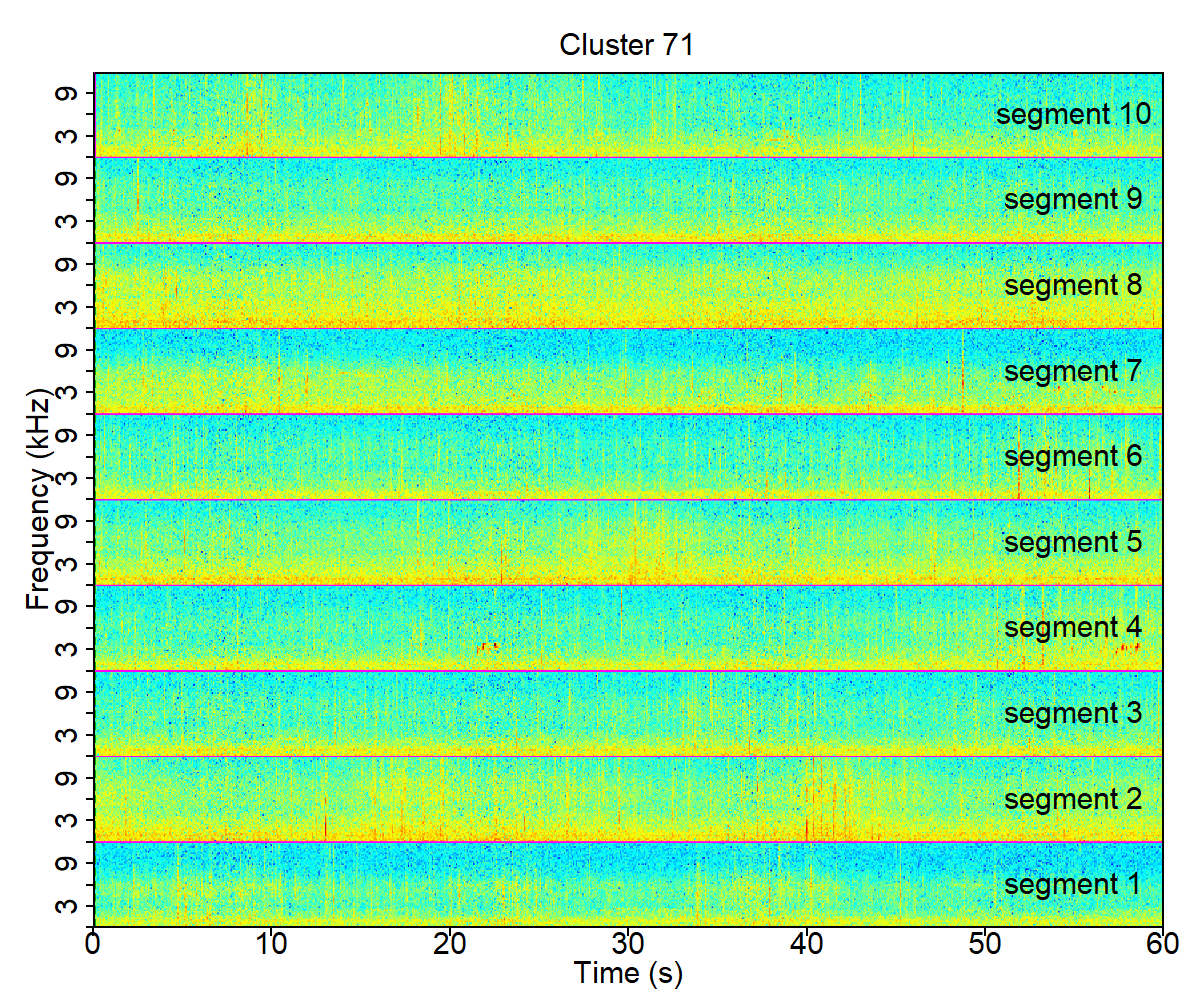

Supplement: Supplemental Information 6 — The spectrograms were computed using a Hann window, FFT = 512, window overlap of 50%, and frame size of 100%. The X-axis represents time, the Y-axis represents frequency. There are 10 audio segments for each cluster. [file peerj-11-16462-s006.zip › Supplemental_Information_S3_spec95_04/Cluster 71.png]

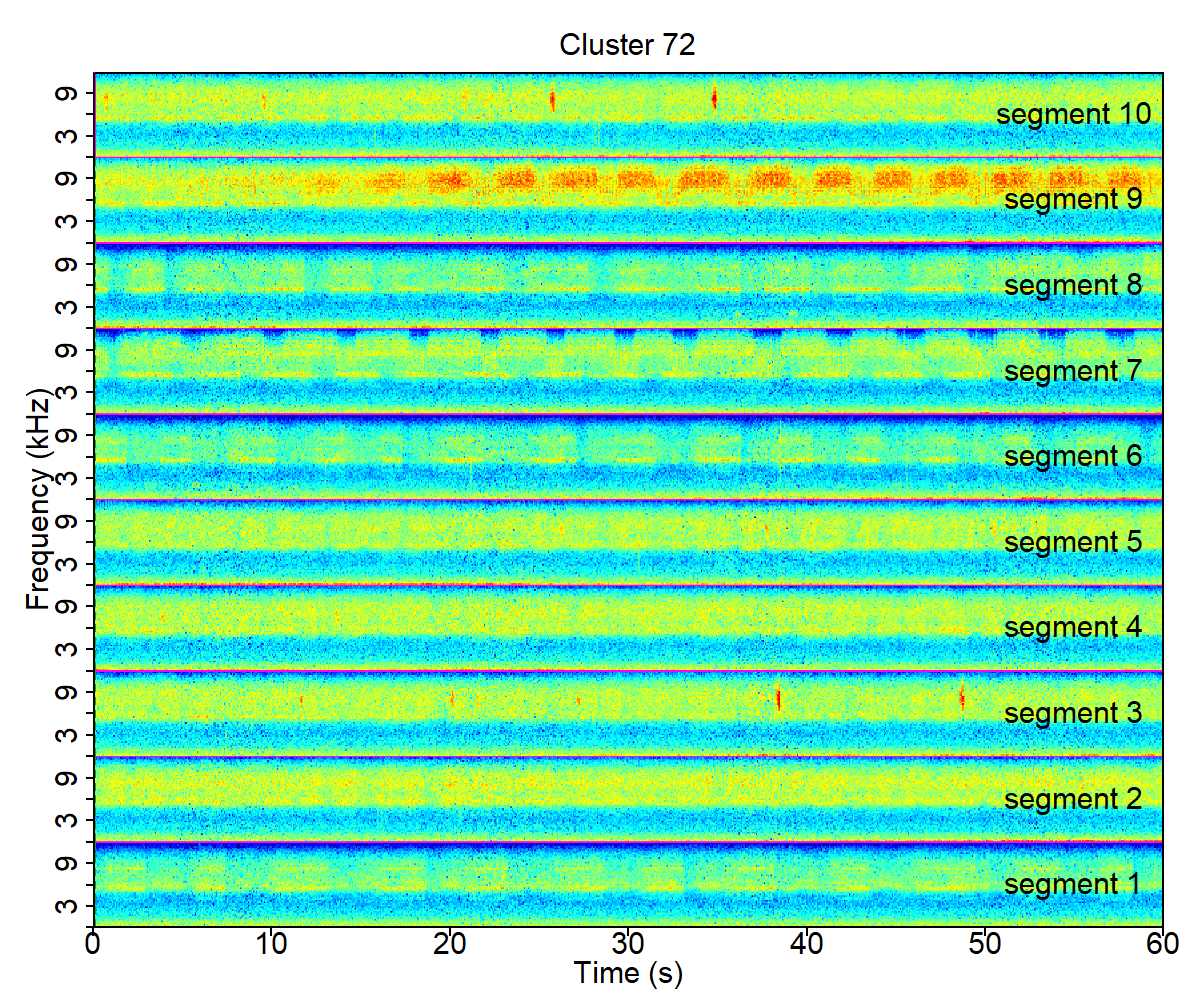

Supplement: Supplemental Information 6 — The spectrograms were computed using a Hann window, FFT = 512, window overlap of 50%, and frame size of 100%. The X-axis represents time, the Y-axis represents frequency. There are 10 audio segments for each cluster. [file peerj-11-16462-s006.zip › Supplemental_Information_S3_spec95_04/Cluster 72.png]

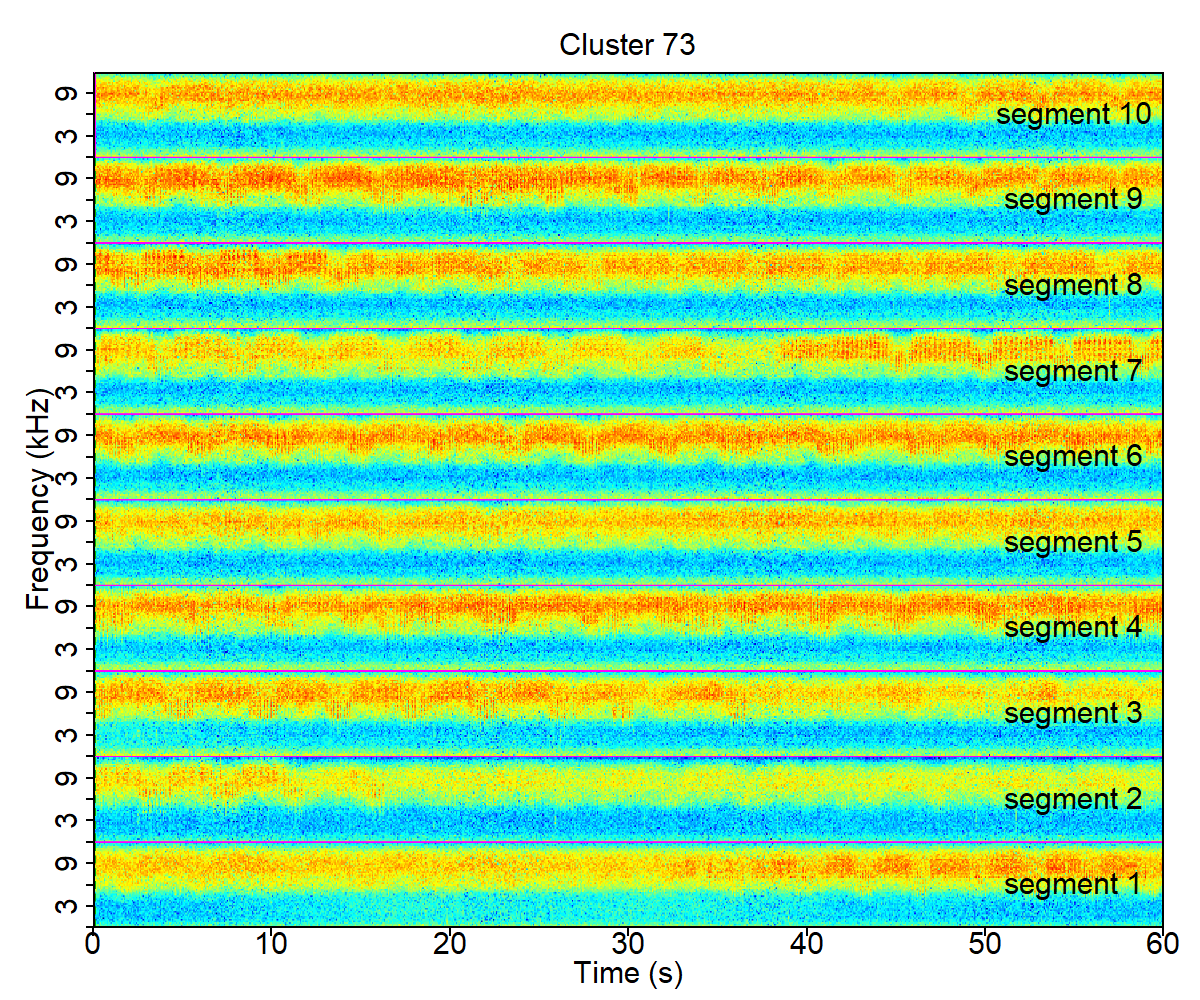

Supplement: Supplemental Information 6 — The spectrograms were computed using a Hann window, FFT = 512, window overlap of 50%, and frame size of 100%. The X-axis represents time, the Y-axis represents frequency. There are 10 audio segments for each cluster. [file peerj-11-16462-s006.zip › Supplemental_Information_S3_spec95_04/Cluster 73.png]

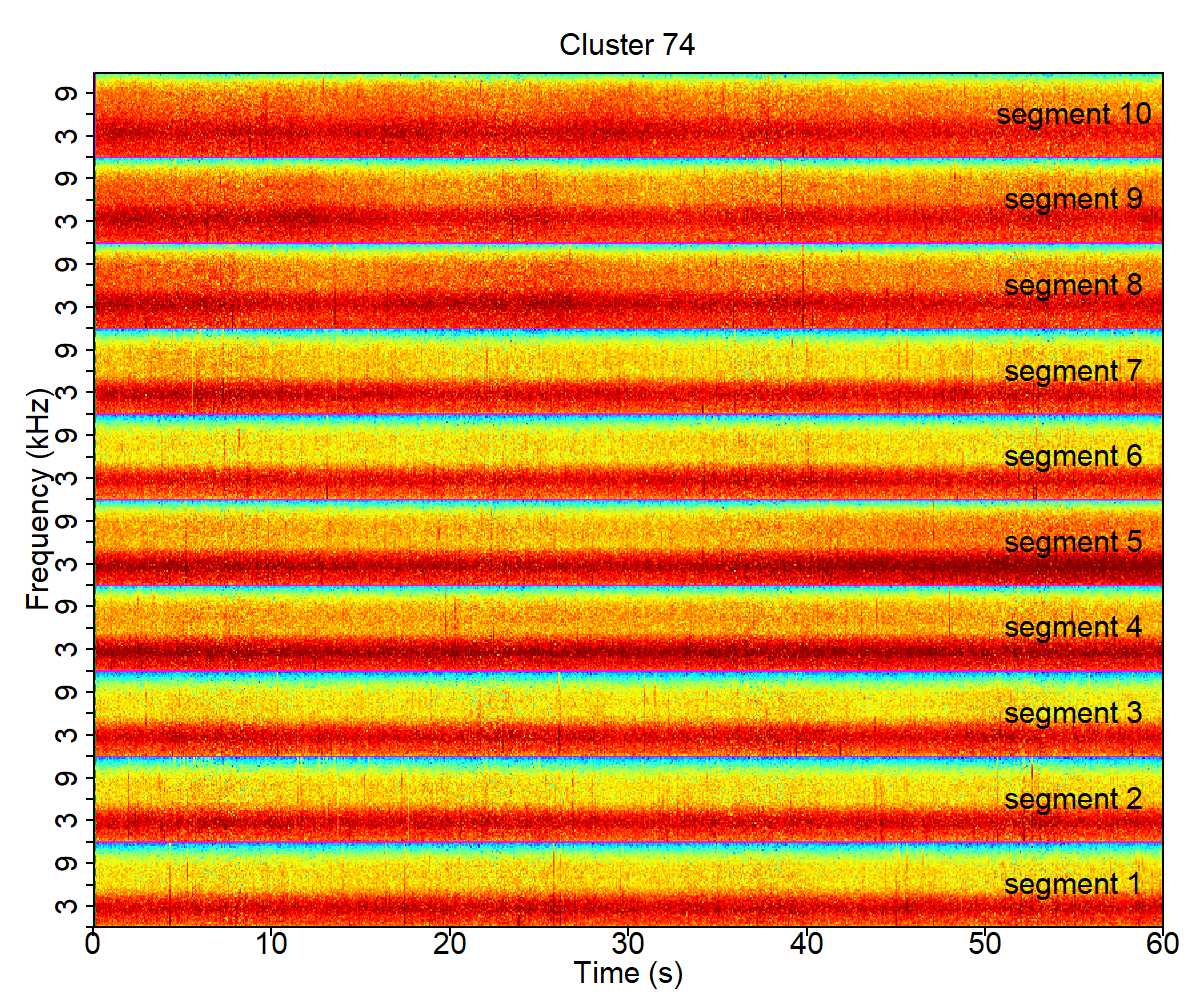

Supplement: Supplemental Information 6 — The spectrograms were computed using a Hann window, FFT = 512, window overlap of 50%, and frame size of 100%. The X-axis represents time, the Y-axis represents frequency. There are 10 audio segments for each cluster. [file peerj-11-16462-s006.zip › Supplemental_Information_S3_spec95_04/Cluster 74.png]

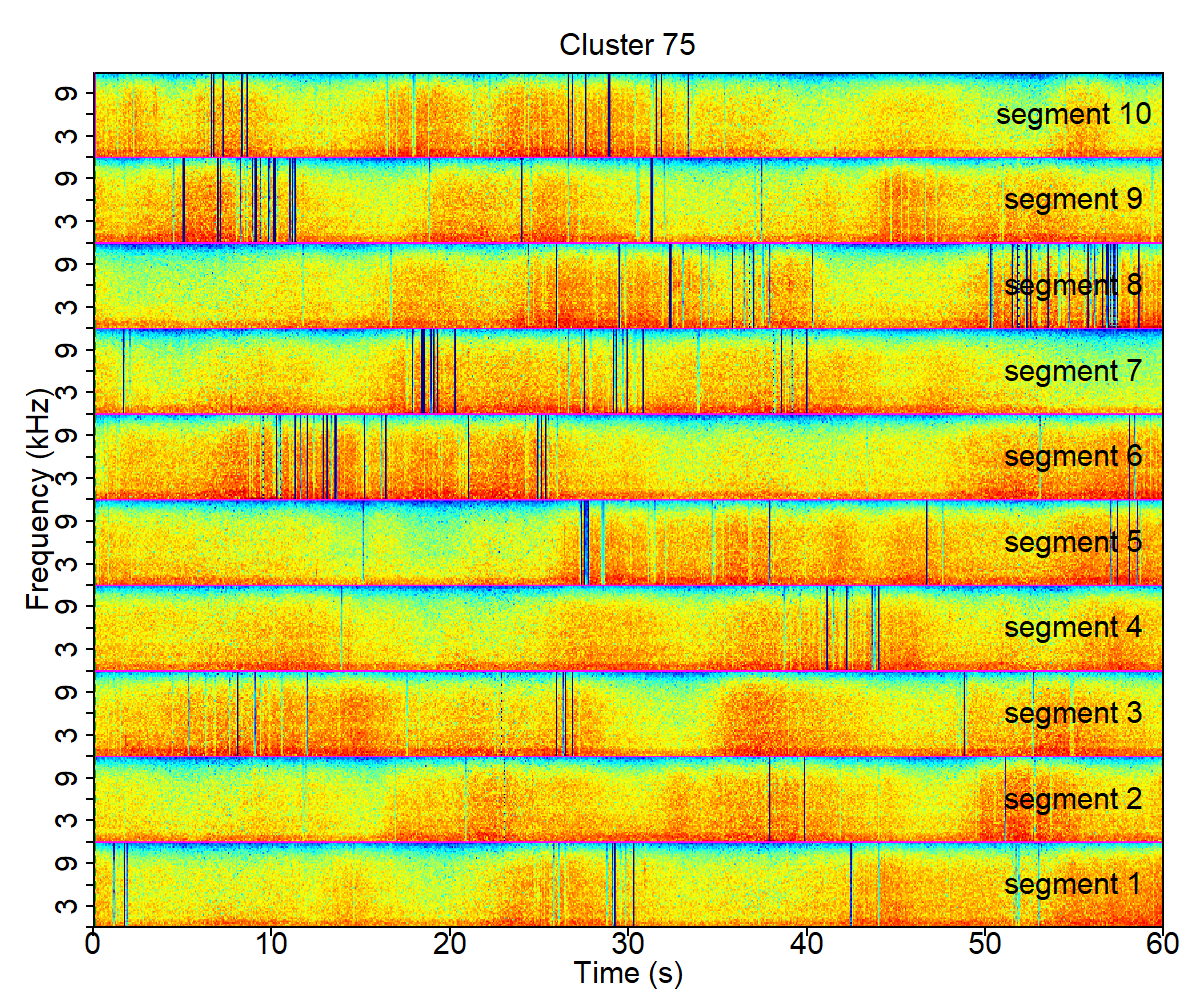

Supplement: Supplemental Information 6 — The spectrograms were computed using a Hann window, FFT = 512, window overlap of 50%, and frame size of 100%. The X-axis represents time, the Y-axis represents frequency. There are 10 audio segments for each cluster. [file peerj-11-16462-s006.zip › Supplemental_Information_S3_spec95_04/Cluster 75.png]

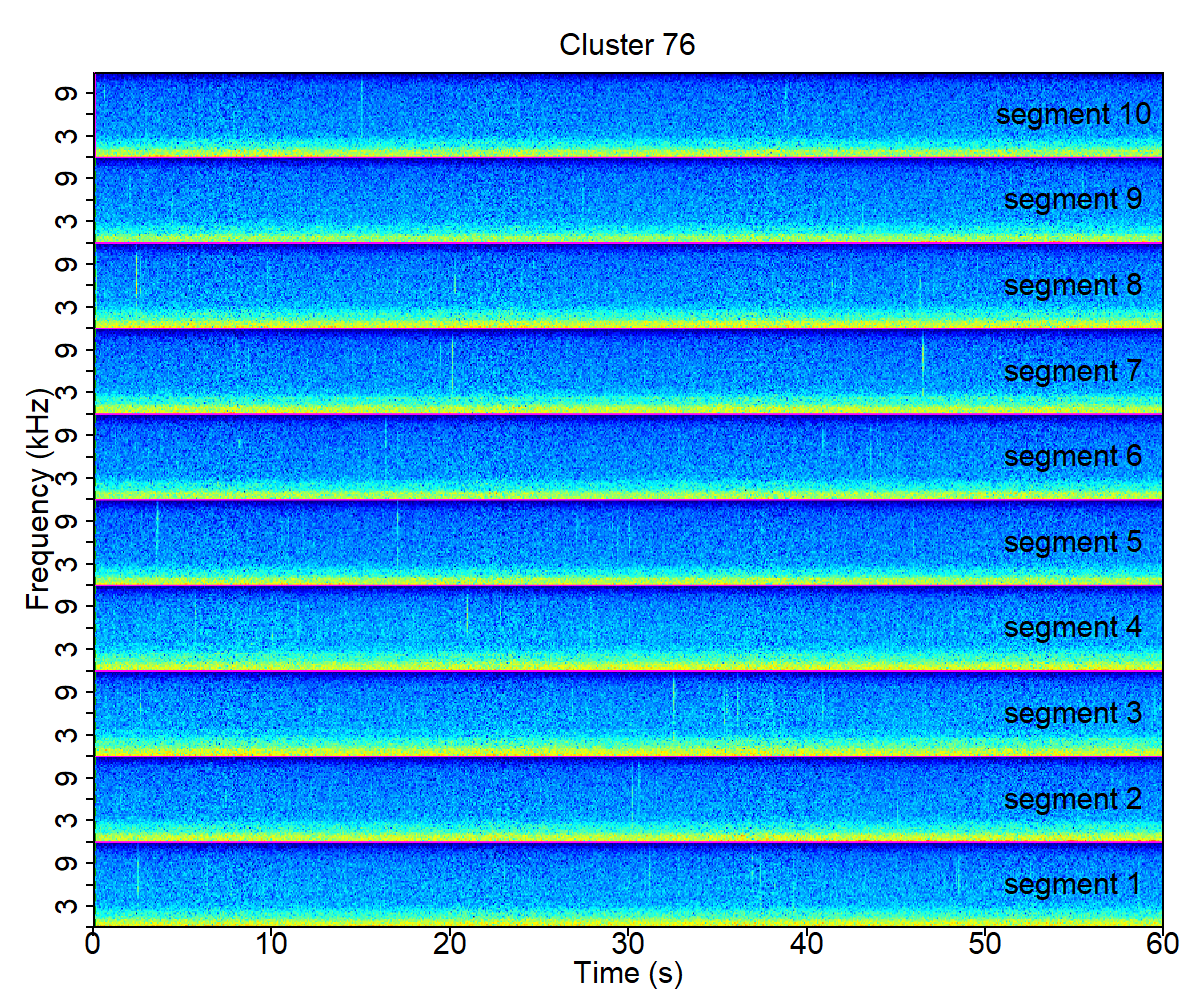

Supplement: Supplemental Information 7 — The spectrograms were computed using a Hann window, FFT = 512, window overlap of 50%, and frame size of 100%. The X-axis represents time, the Y-axis represents frequency. There are 10 audio segments for each cluster. [file peerj-11-16462-s007.zip › Supplemental_Information_S3_spec95_05/Cluster 76.png]

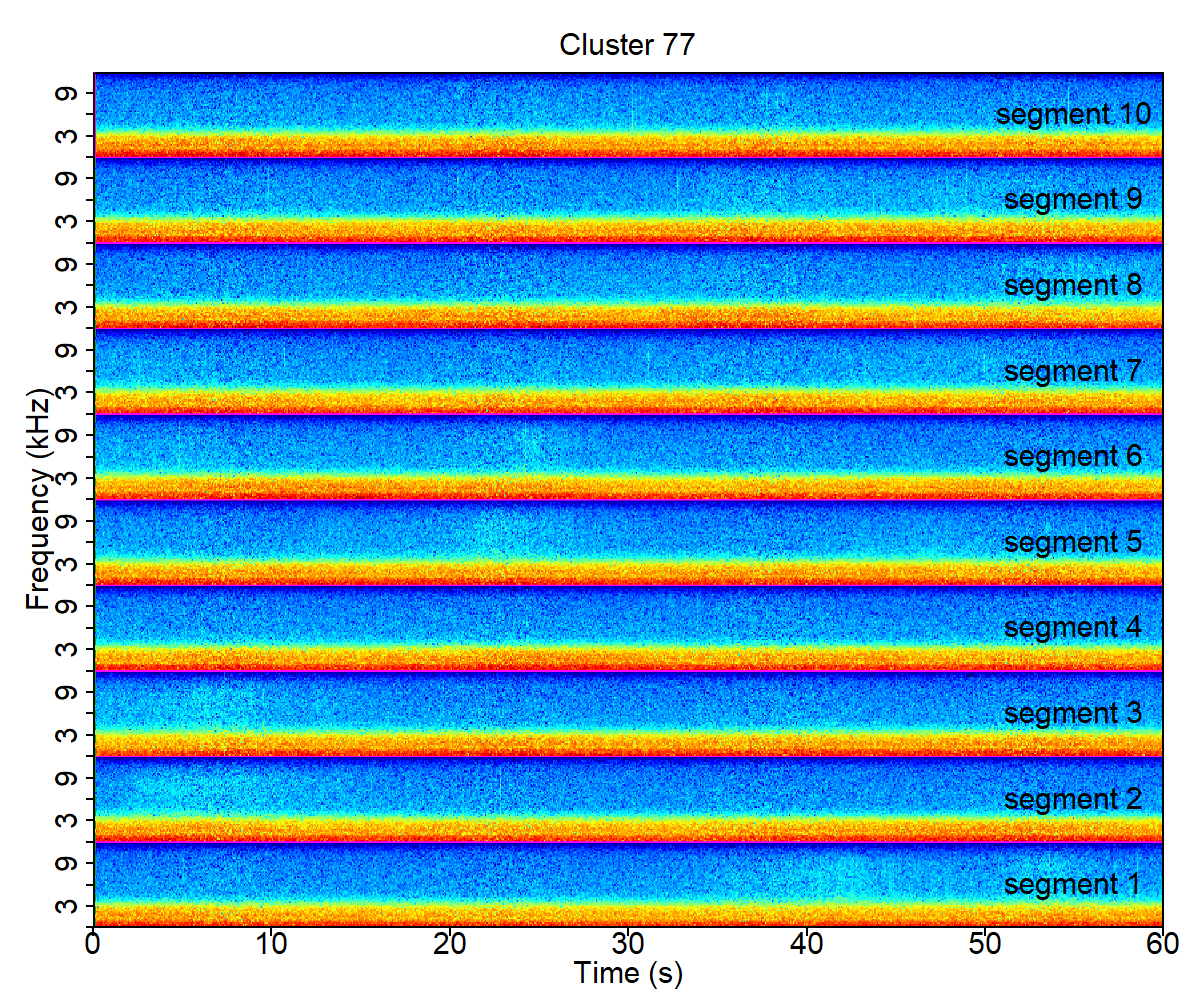

Supplement: Supplemental Information 7 — The spectrograms were computed using a Hann window, FFT = 512, window overlap of 50%, and frame size of 100%. The X-axis represents time, the Y-axis represents frequency. There are 10 audio segments for each cluster. [file peerj-11-16462-s007.zip › Supplemental_Information_S3_spec95_05/Cluster 77.png]

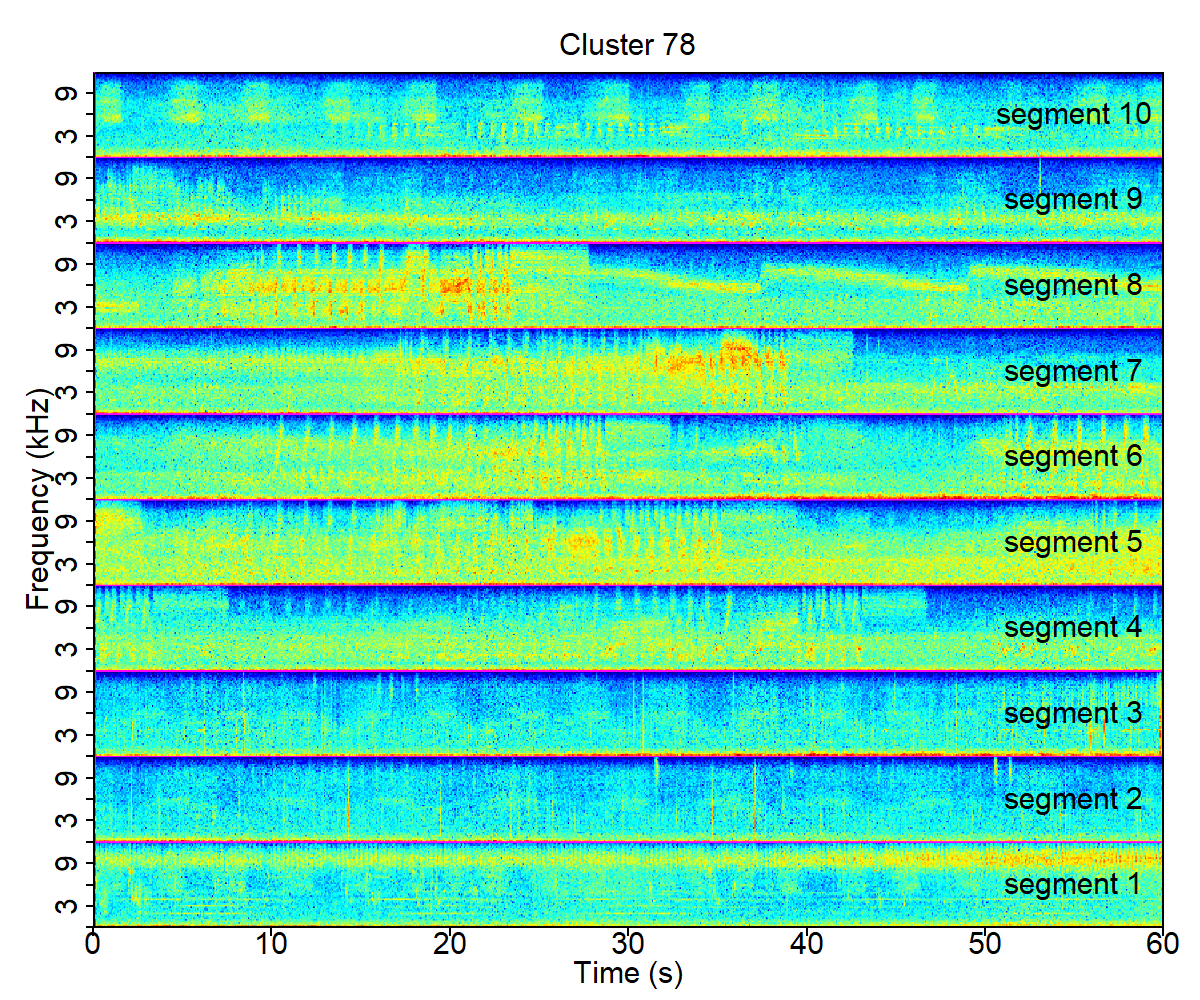

Supplement: Supplemental Information 7 — The spectrograms were computed using a Hann window, FFT = 512, window overlap of 50%, and frame size of 100%. The X-axis represents time, the Y-axis represents frequency. There are 10 audio segments for each cluster. [file peerj-11-16462-s007.zip › Supplemental_Information_S3_spec95_05/Cluster 78.png]

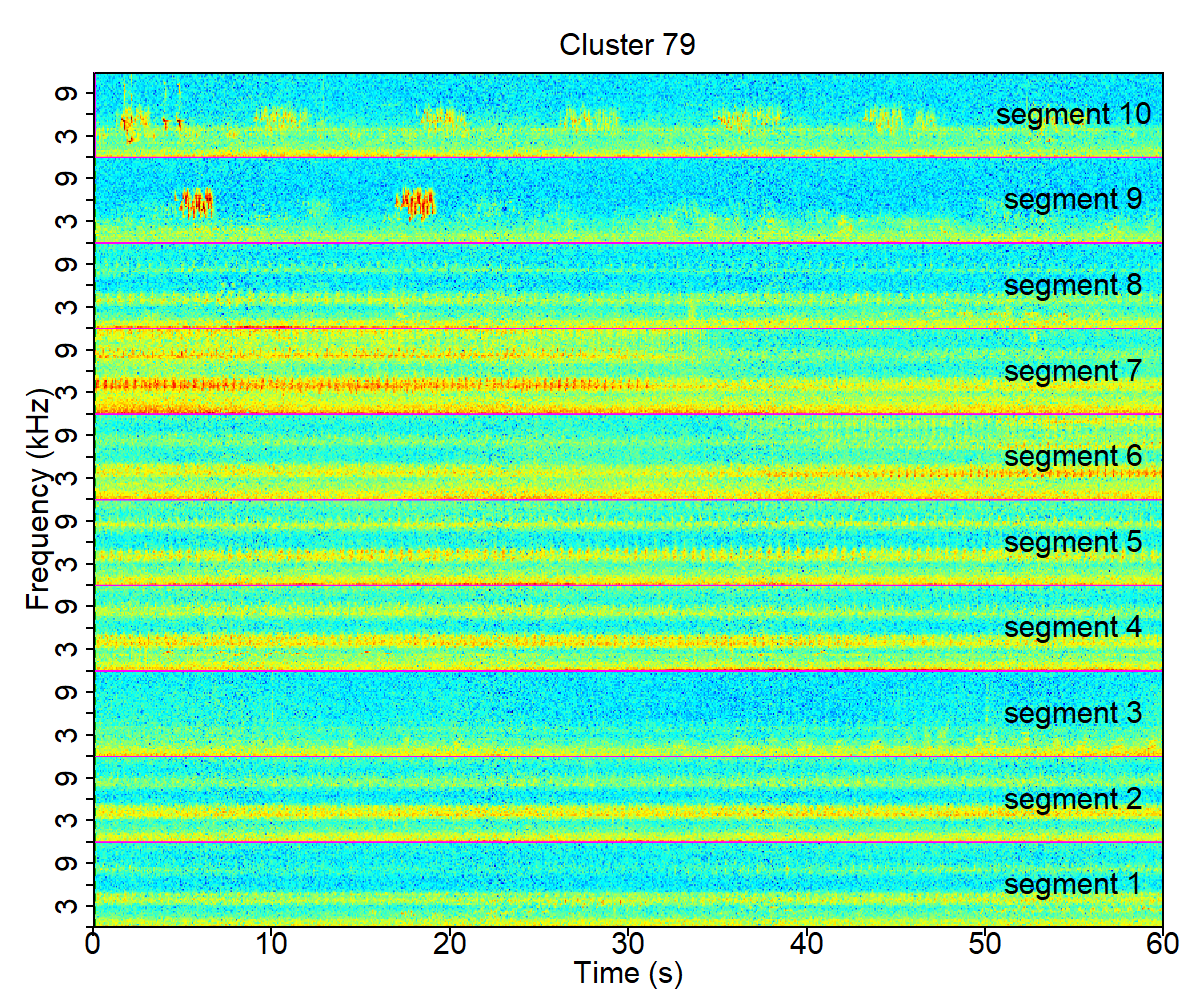

Supplement: Supplemental Information 7 — The spectrograms were computed using a Hann window, FFT = 512, window overlap of 50%, and frame size of 100%. The X-axis represents time, the Y-axis represents frequency. There are 10 audio segments for each cluster. [file peerj-11-16462-s007.zip › Supplemental_Information_S3_spec95_05/Cluster 79.png]

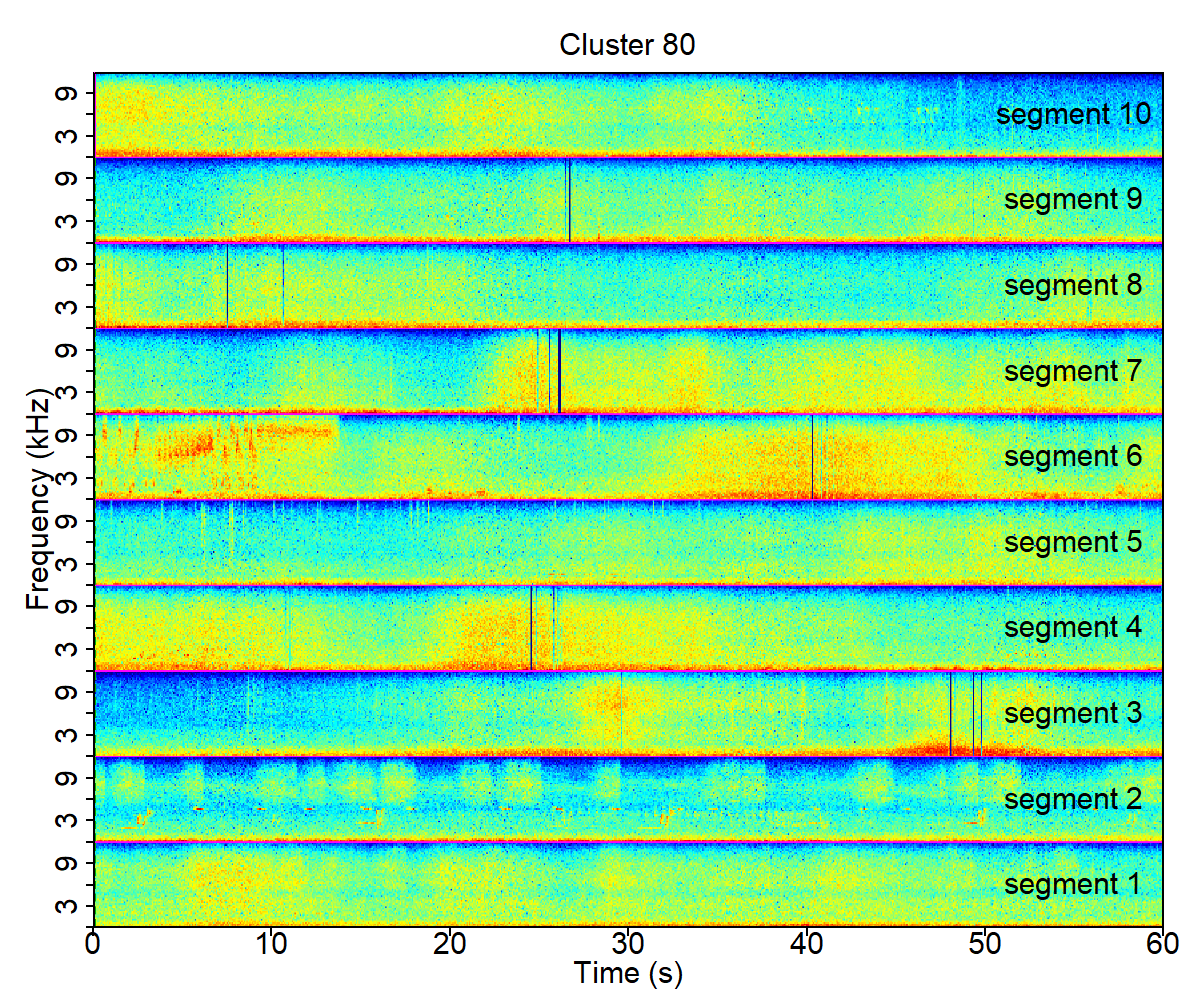

Supplement: Supplemental Information 7 — The spectrograms were computed using a Hann window, FFT = 512, window overlap of 50%, and frame size of 100%. The X-axis represents time, the Y-axis represents frequency. There are 10 audio segments for each cluster. [file peerj-11-16462-s007.zip › Supplemental_Information_S3_spec95_05/Cluster 80.png]

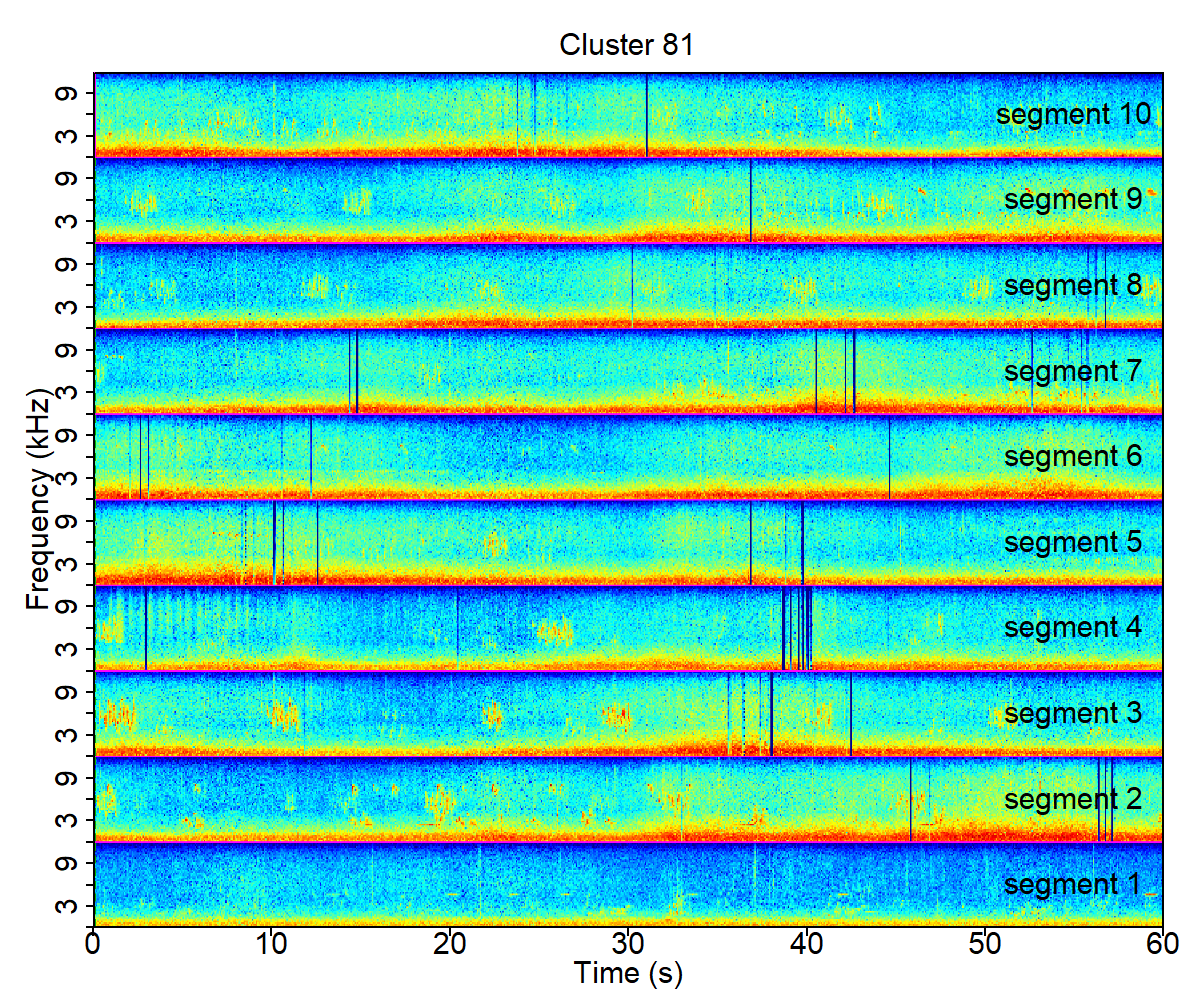

Supplement: Supplemental Information 7 — The spectrograms were computed using a Hann window, FFT = 512, window overlap of 50%, and frame size of 100%. The X-axis represents time, the Y-axis represents frequency. There are 10 audio segments for each cluster. [file peerj-11-16462-s007.zip › Supplemental_Information_S3_spec95_05/Cluster 81.png]

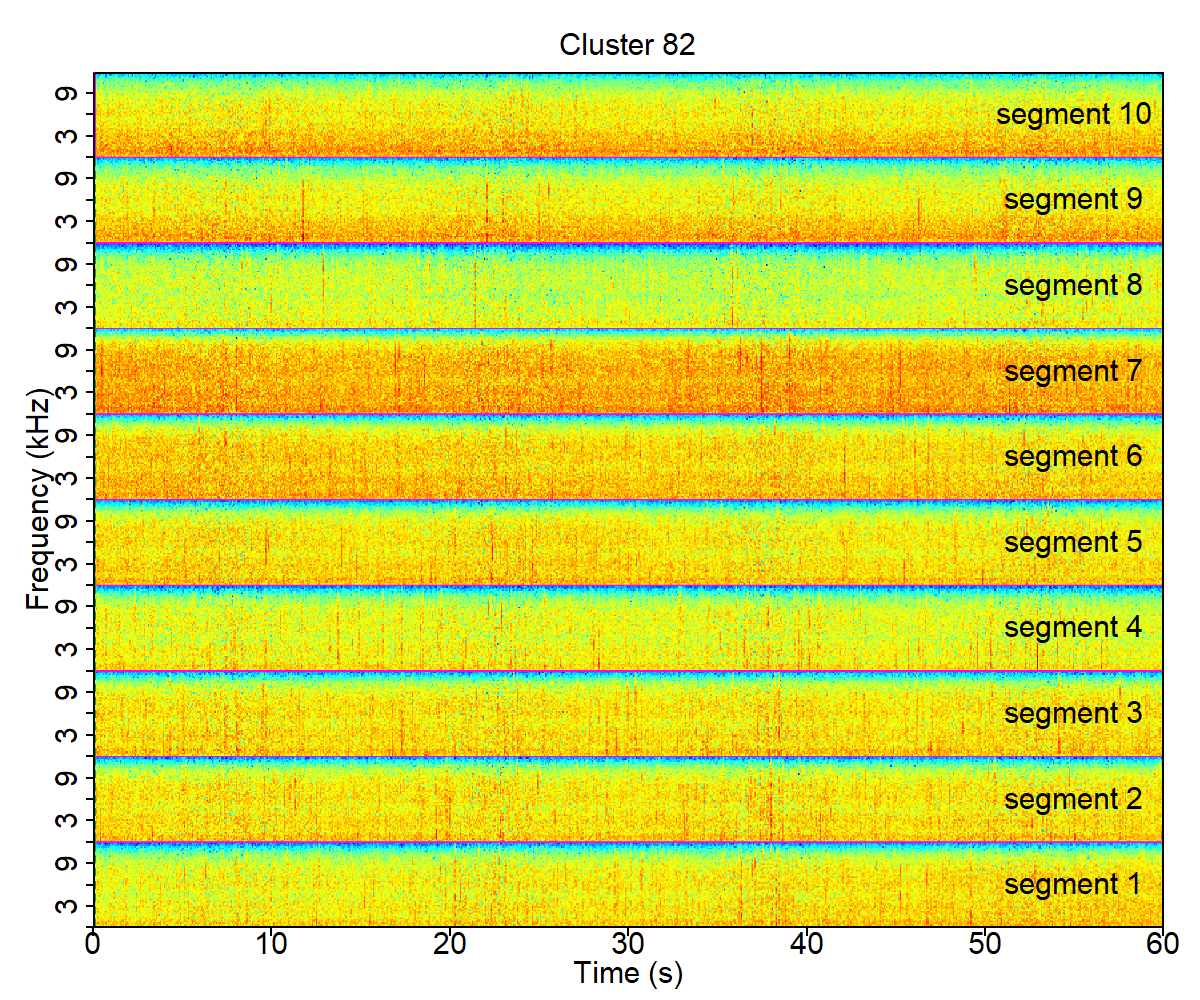

Supplement: Supplemental Information 7 — The spectrograms were computed using a Hann window, FFT = 512, window overlap of 50%, and frame size of 100%. The X-axis represents time, the Y-axis represents frequency. There are 10 audio segments for each cluster. [file peerj-11-16462-s007.zip › Supplemental_Information_S3_spec95_05/Cluster 82.png]

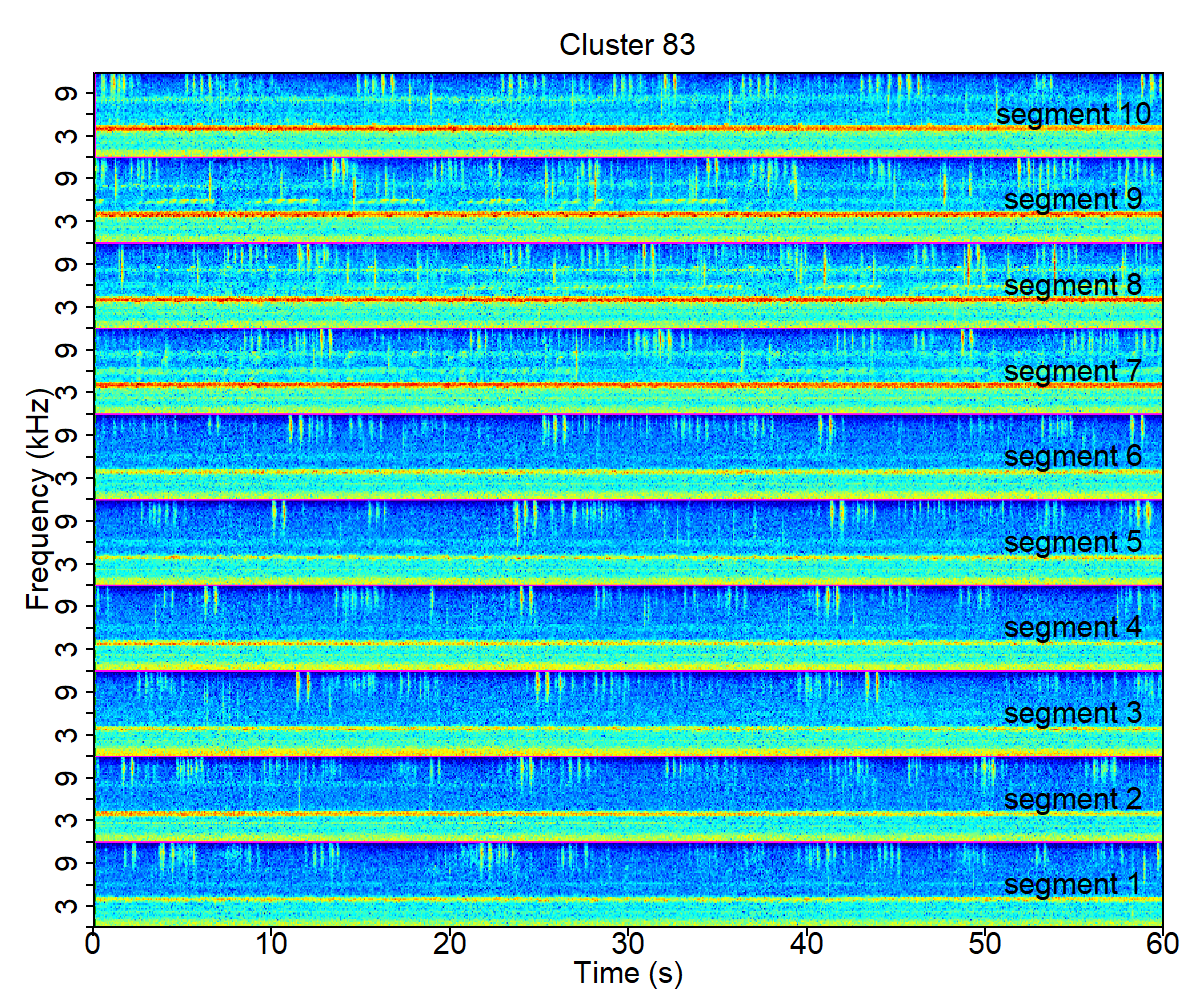

Supplement: Supplemental Information 7 — The spectrograms were computed using a Hann window, FFT = 512, window overlap of 50%, and frame size of 100%. The X-axis represents time, the Y-axis represents frequency. There are 10 audio segments for each cluster. [file peerj-11-16462-s007.zip › Supplemental_Information_S3_spec95_05/Cluster 83.png]

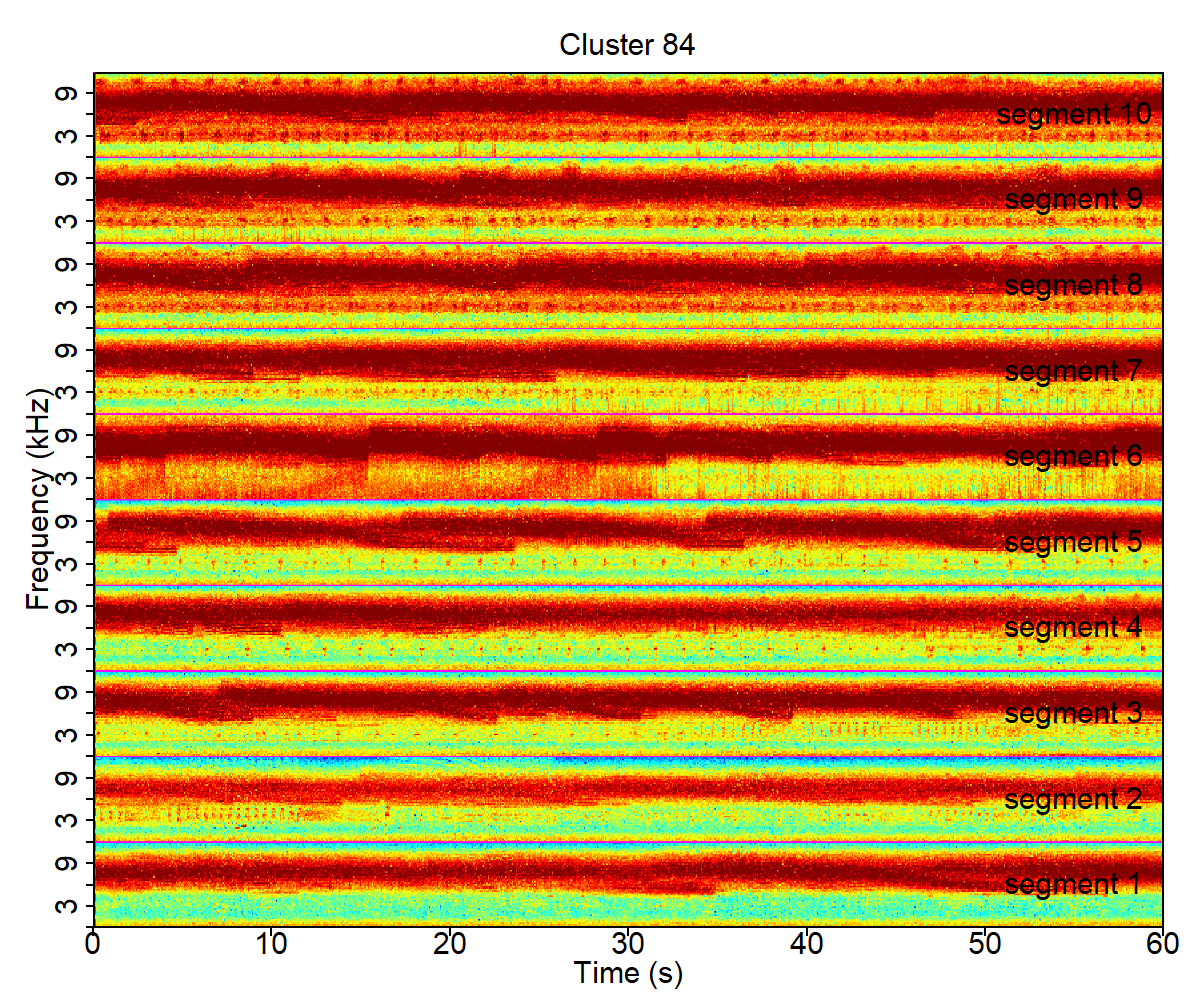

Supplement: Supplemental Information 7 — The spectrograms were computed using a Hann window, FFT = 512, window overlap of 50%, and frame size of 100%. The X-axis represents time, the Y-axis represents frequency. There are 10 audio segments for each cluster. [file peerj-11-16462-s007.zip › Supplemental_Information_S3_spec95_05/Cluster 84.png]

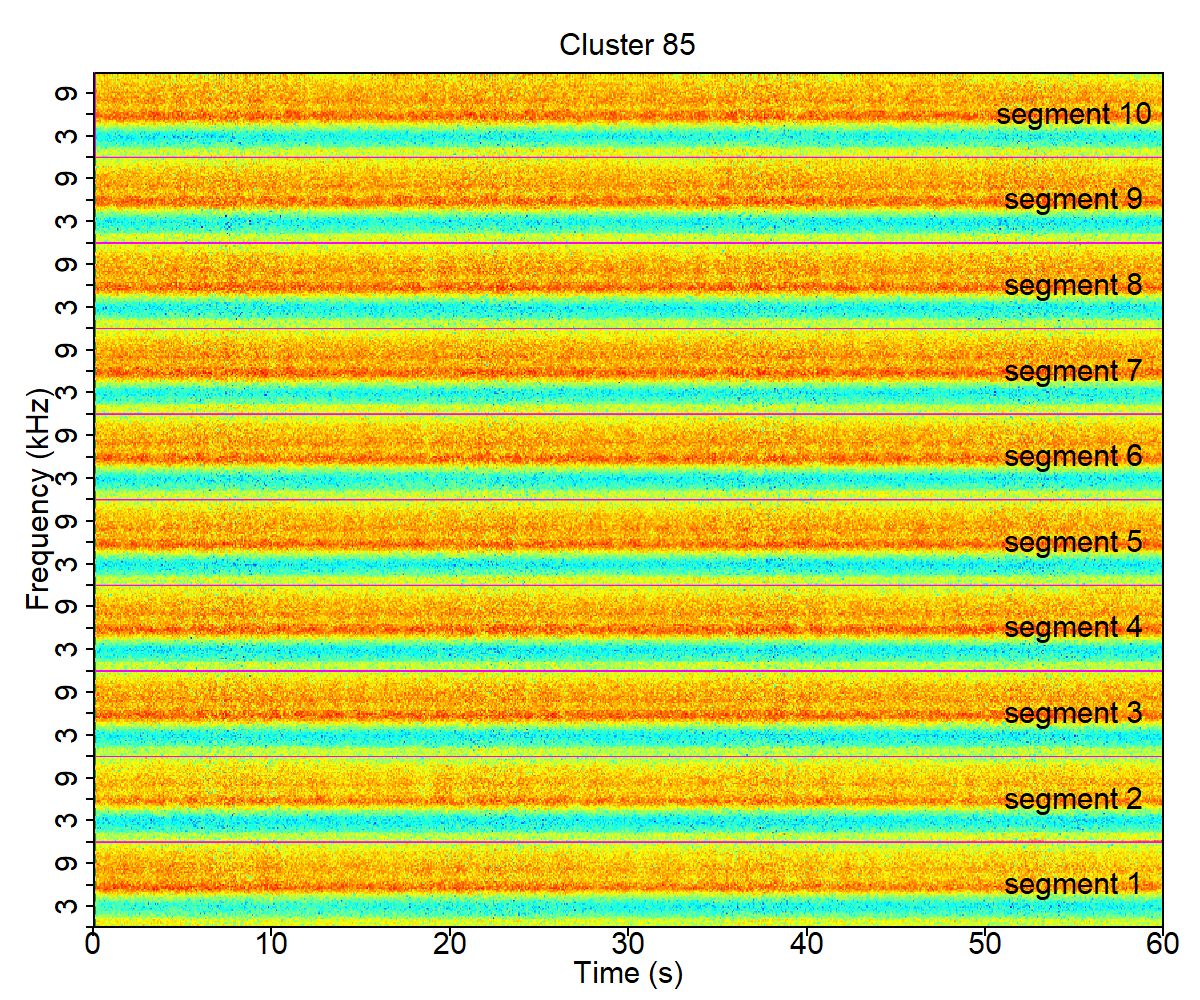

Supplement: Supplemental Information 7 — The spectrograms were computed using a Hann window, FFT = 512, window overlap of 50%, and frame size of 100%. The X-axis represents time, the Y-axis represents frequency. There are 10 audio segments for each cluster. [file peerj-11-16462-s007.zip › Supplemental_Information_S3_spec95_05/Cluster 85.png]

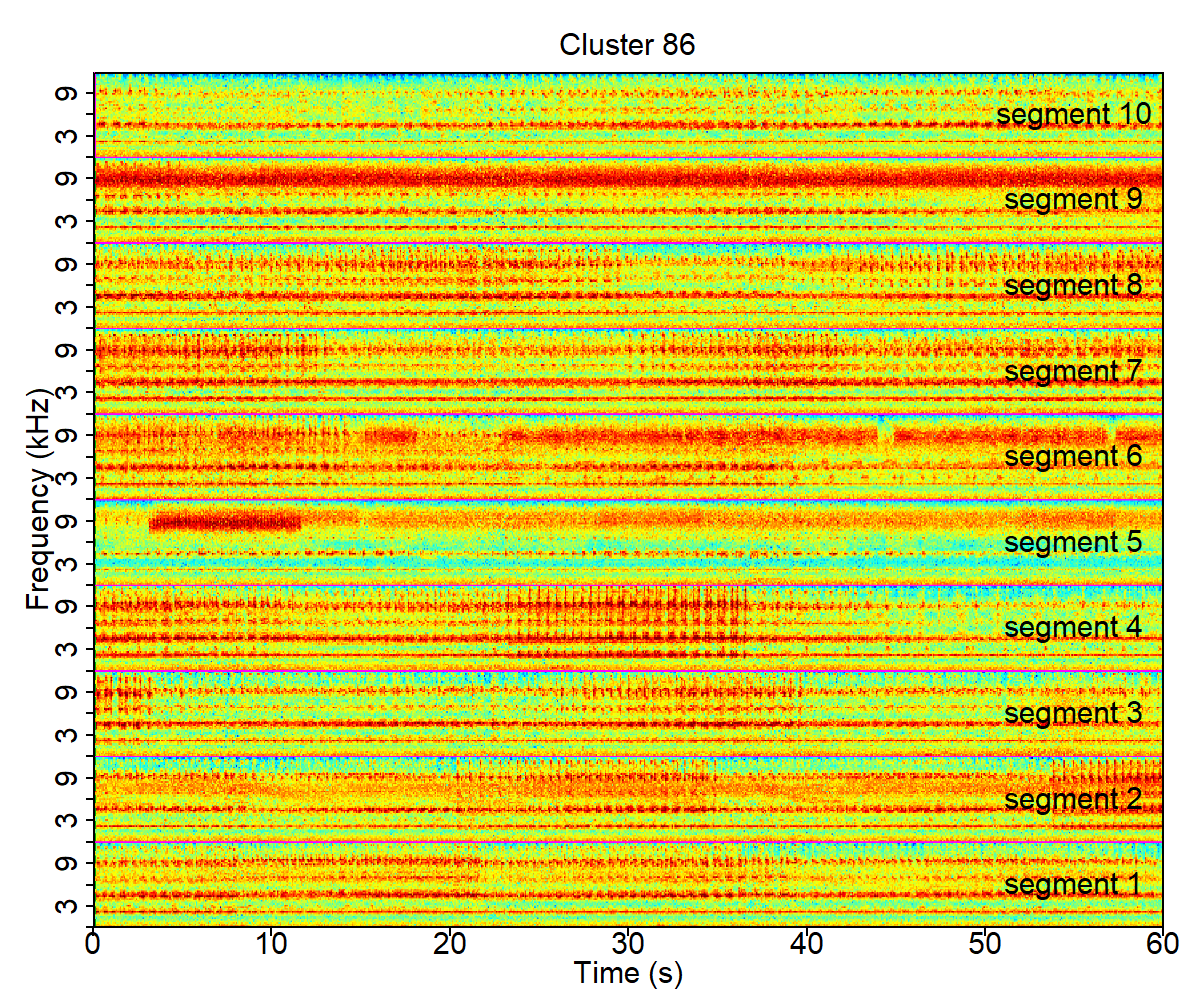

Supplement: Supplemental Information 7 — The spectrograms were computed using a Hann window, FFT = 512, window overlap of 50%, and frame size of 100%. The X-axis represents time, the Y-axis represents frequency. There are 10 audio segments for each cluster. [file peerj-11-16462-s007.zip › Supplemental_Information_S3_spec95_05/Cluster 86.png]

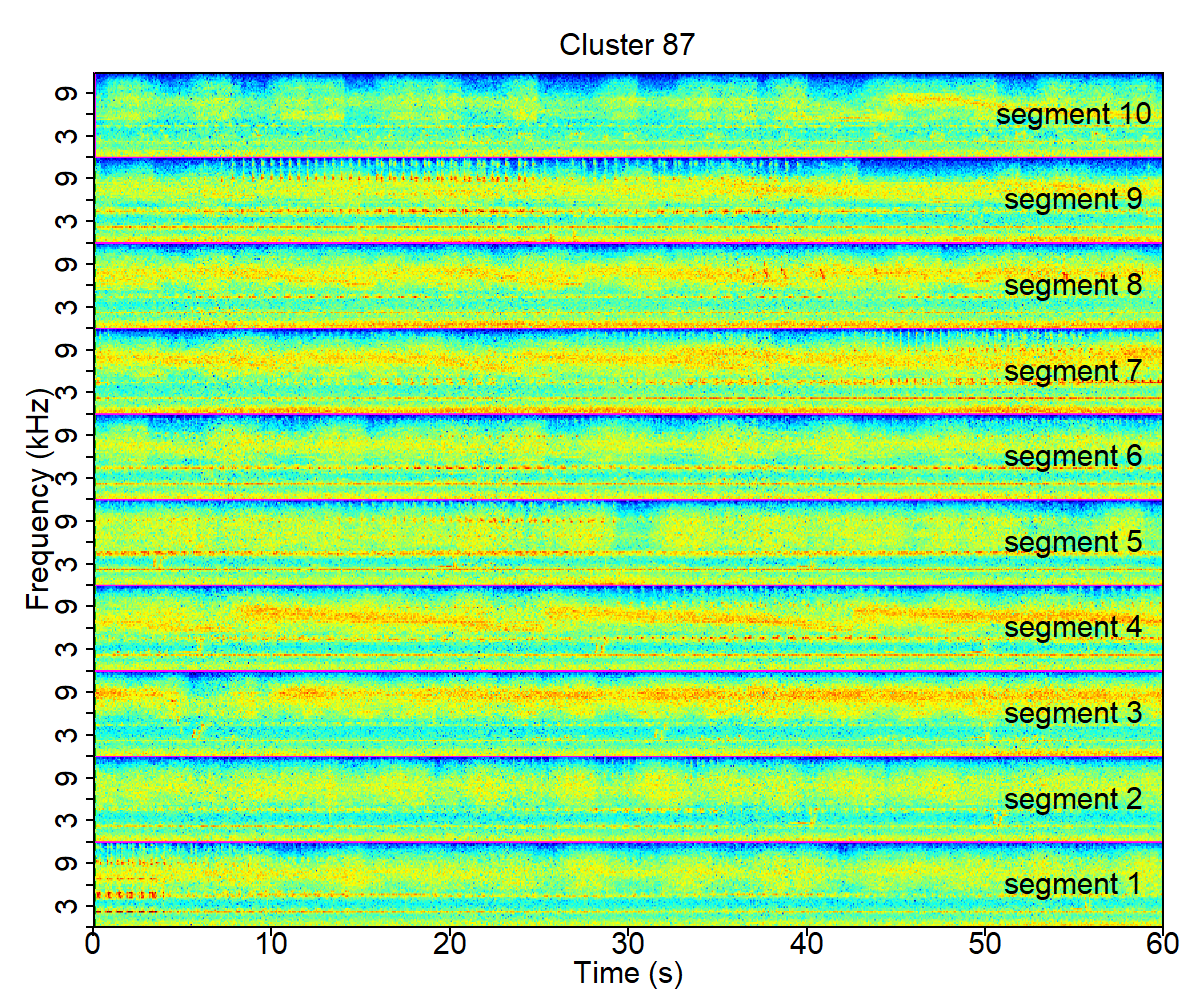

Supplement: Supplemental Information 7 — The spectrograms were computed using a Hann window, FFT = 512, window overlap of 50%, and frame size of 100%. The X-axis represents time, the Y-axis represents frequency. There are 10 audio segments for each cluster. [file peerj-11-16462-s007.zip › Supplemental_Information_S3_spec95_05/Cluster 87.png]

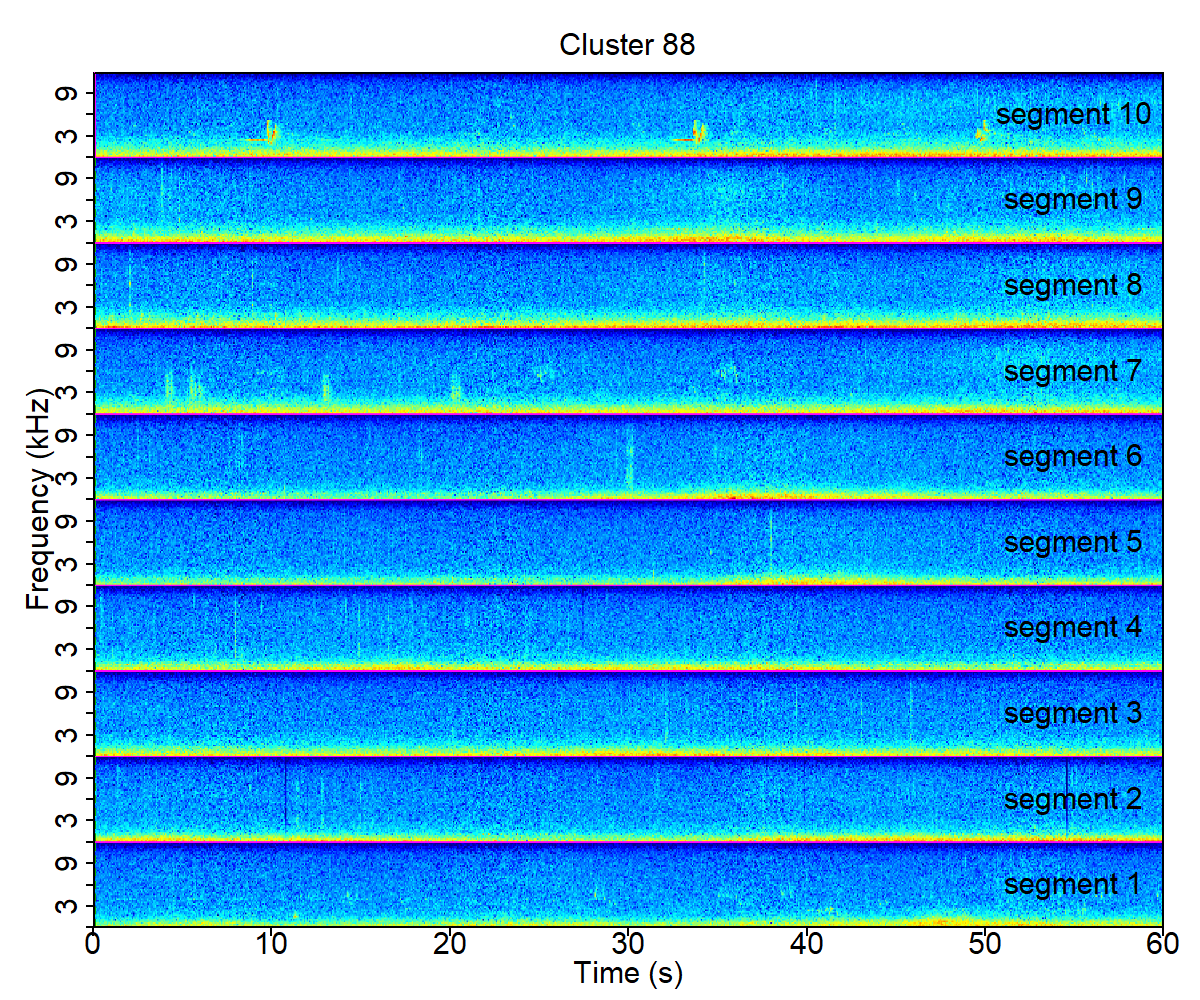

Supplement: Supplemental Information 7 — The spectrograms were computed using a Hann window, FFT = 512, window overlap of 50%, and frame size of 100%. The X-axis represents time, the Y-axis represents frequency. There are 10 audio segments for each cluster. [file peerj-11-16462-s007.zip › Supplemental_Information_S3_spec95_05/Cluster 88.png]

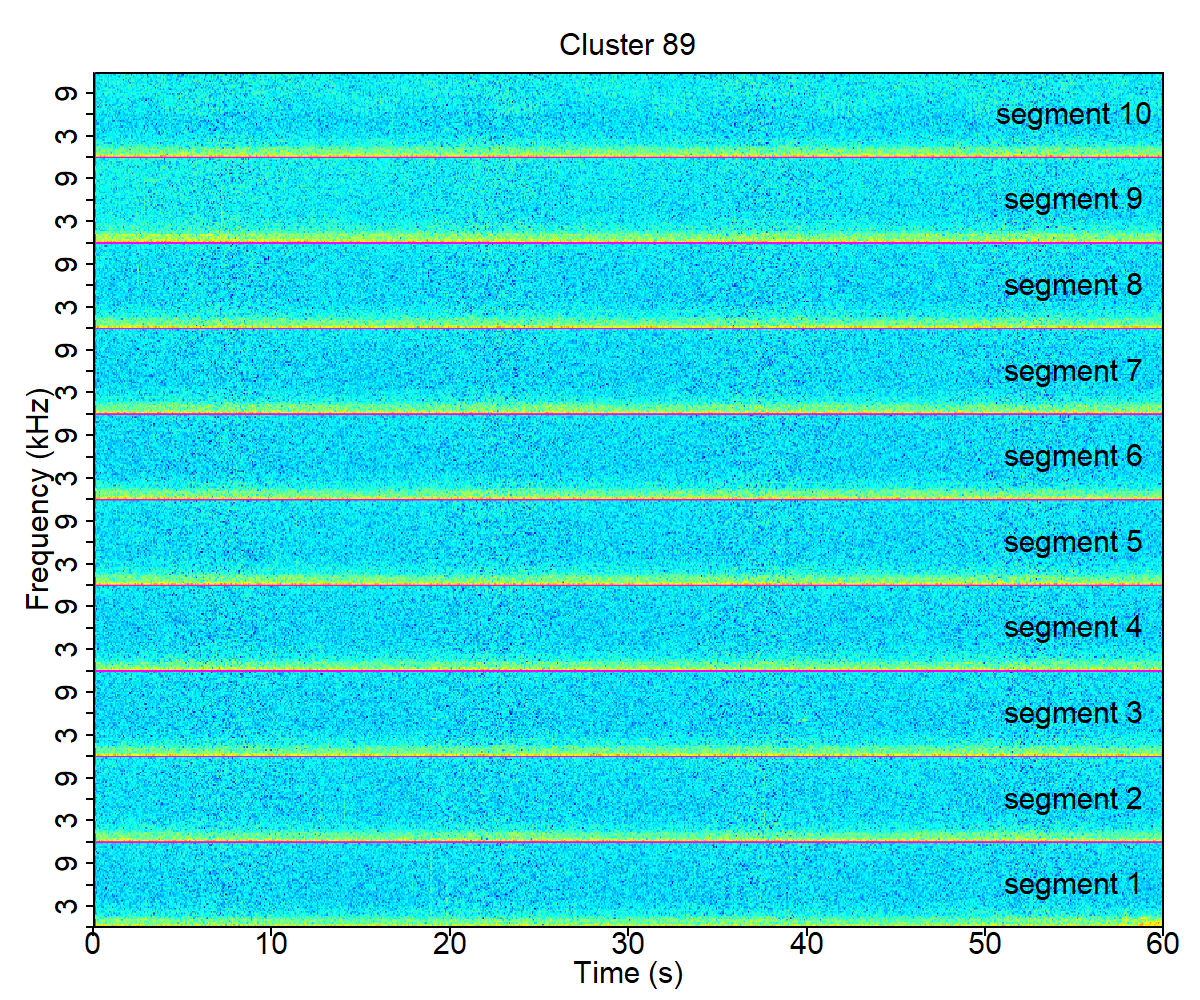

Supplement: Supplemental Information 7 — The spectrograms were computed using a Hann window, FFT = 512, window overlap of 50%, and frame size of 100%. The X-axis represents time, the Y-axis represents frequency. There are 10 audio segments for each cluster. [file peerj-11-16462-s007.zip › Supplemental_Information_S3_spec95_05/Cluster 89.png]

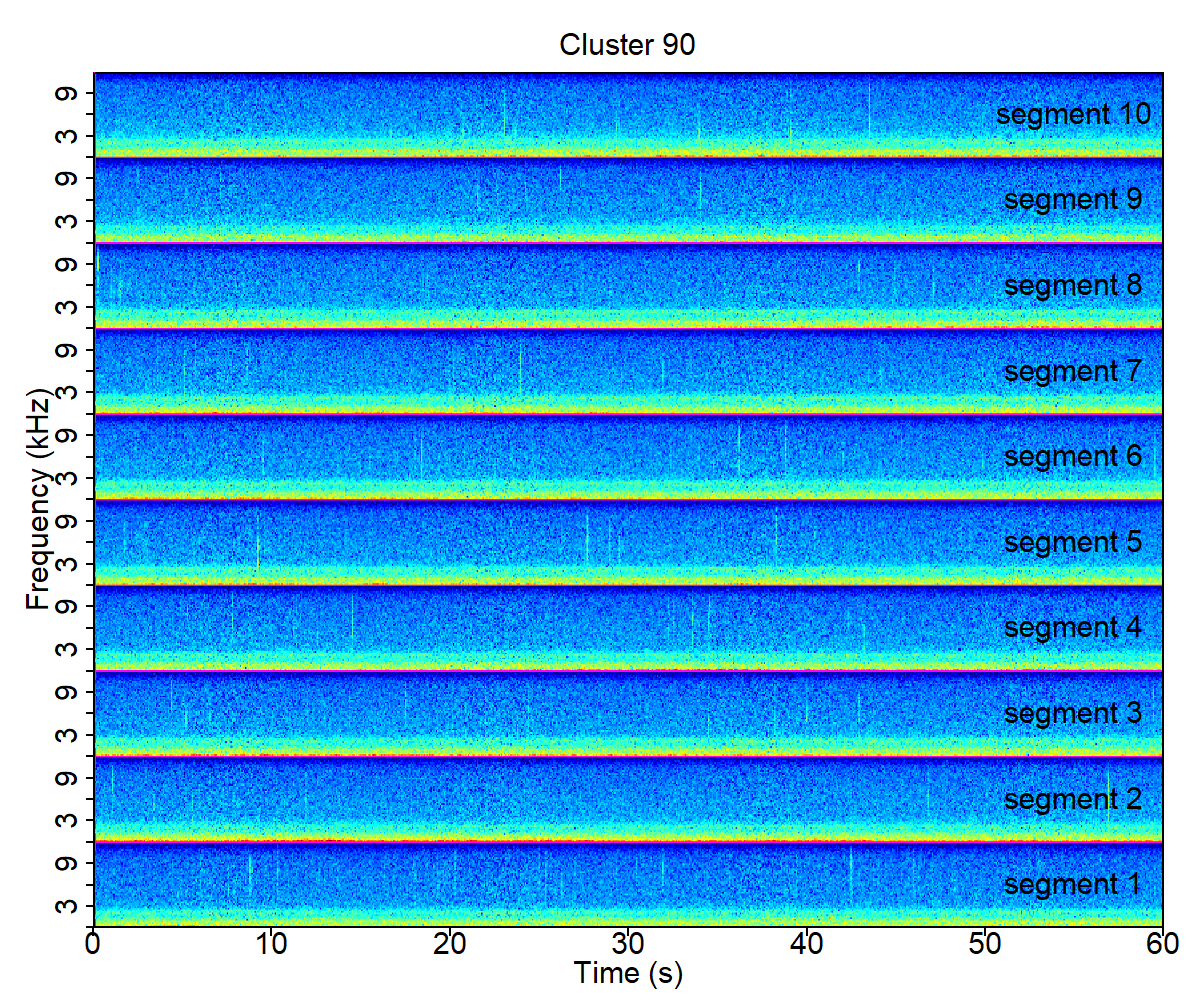

Supplement: Supplemental Information 7 — The spectrograms were computed using a Hann window, FFT = 512, window overlap of 50%, and frame size of 100%. The X-axis represents time, the Y-axis represents frequency. There are 10 audio segments for each cluster. [file peerj-11-16462-s007.zip › Supplemental_Information_S3_spec95_05/Cluster 90.png]

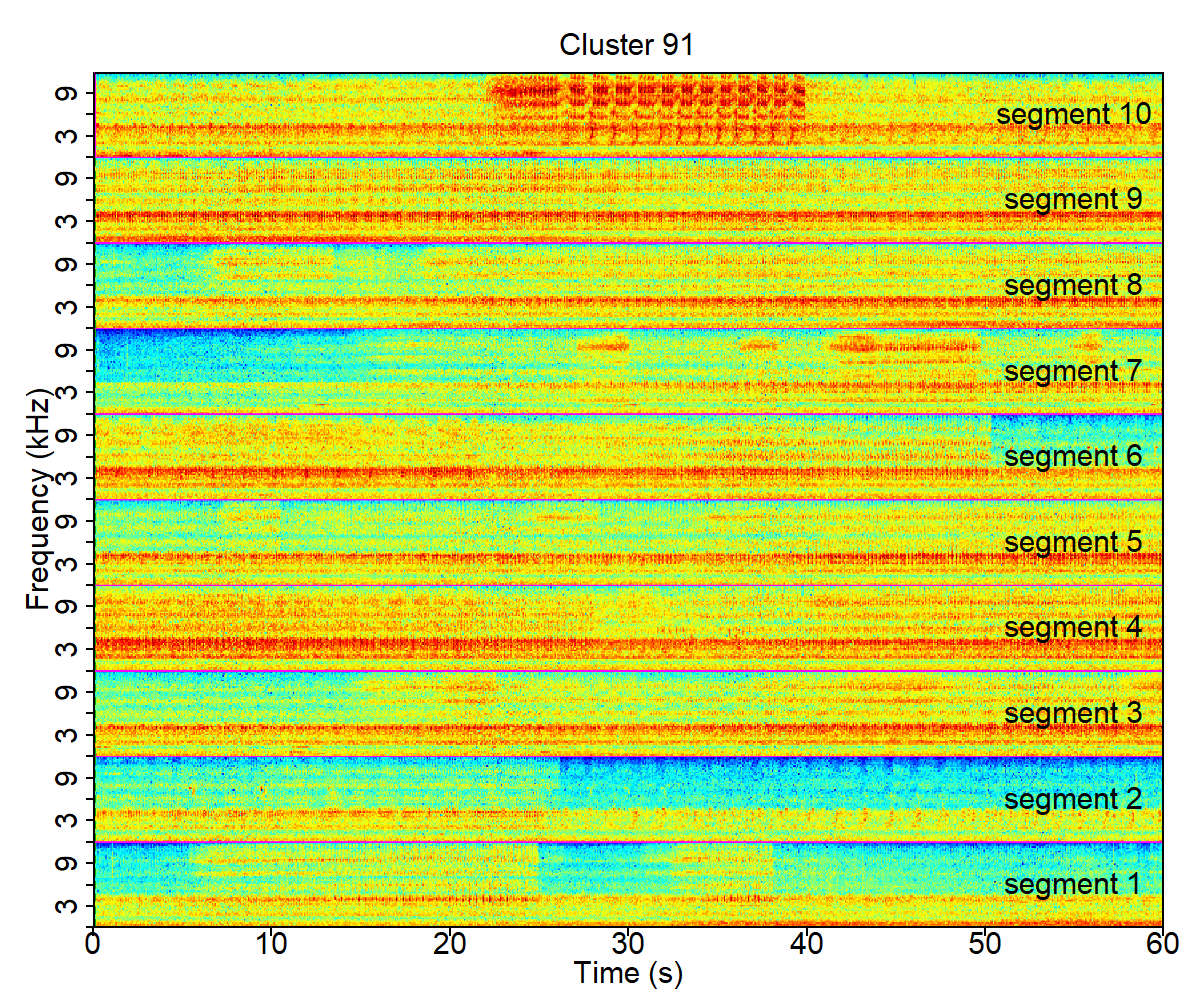

Supplement: Supplemental Information 7 — The spectrograms were computed using a Hann window, FFT = 512, window overlap of 50%, and frame size of 100%. The X-axis represents time, the Y-axis represents frequency. There are 10 audio segments for each cluster. [file peerj-11-16462-s007.zip › Supplemental_Information_S3_spec95_05/Cluster 91.png]

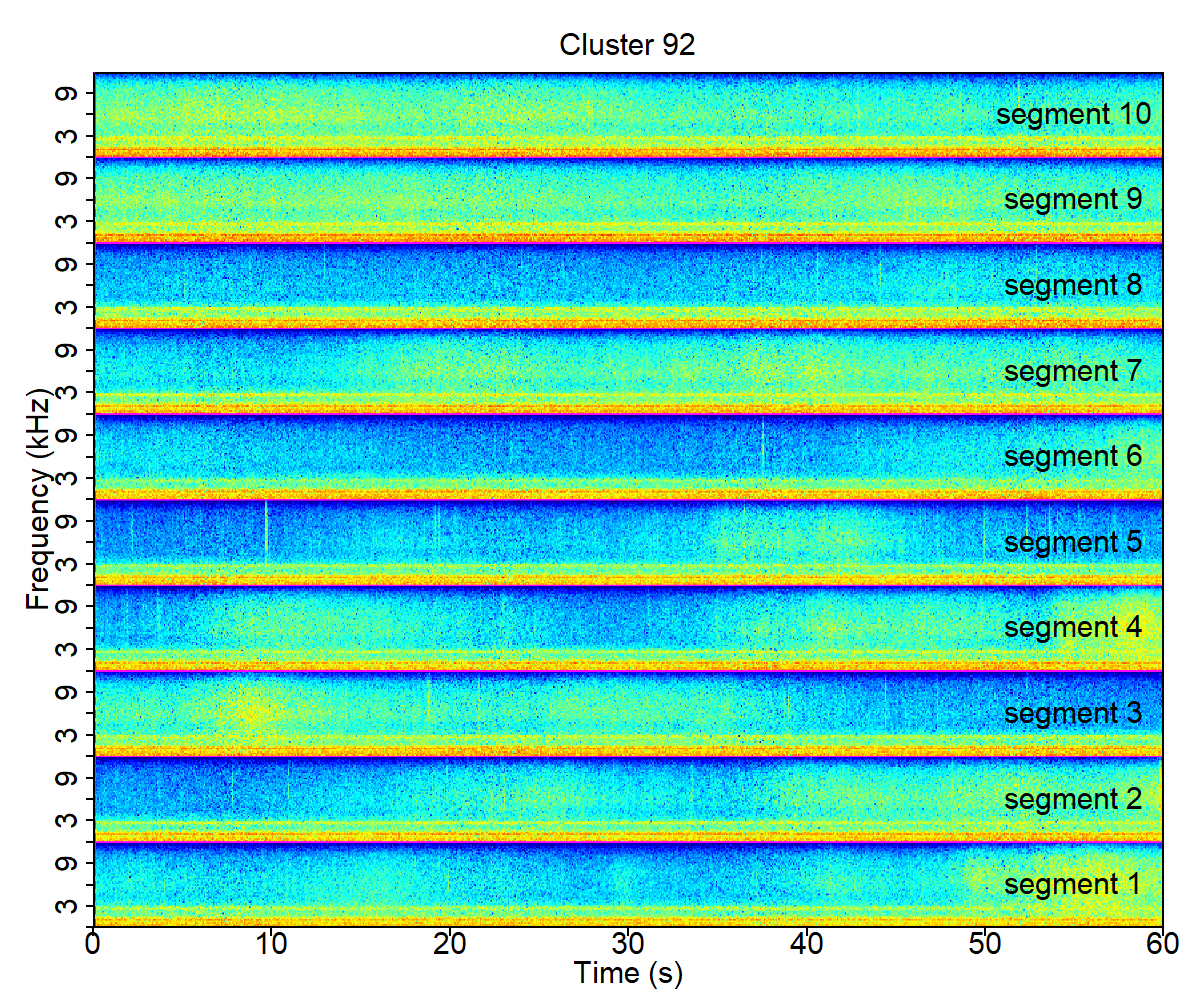

Supplement: Supplemental Information 7 — The spectrograms were computed using a Hann window, FFT = 512, window overlap of 50%, and frame size of 100%. The X-axis represents time, the Y-axis represents frequency. There are 10 audio segments for each cluster. [file peerj-11-16462-s007.zip › Supplemental_Information_S3_spec95_05/Cluster 92.png]

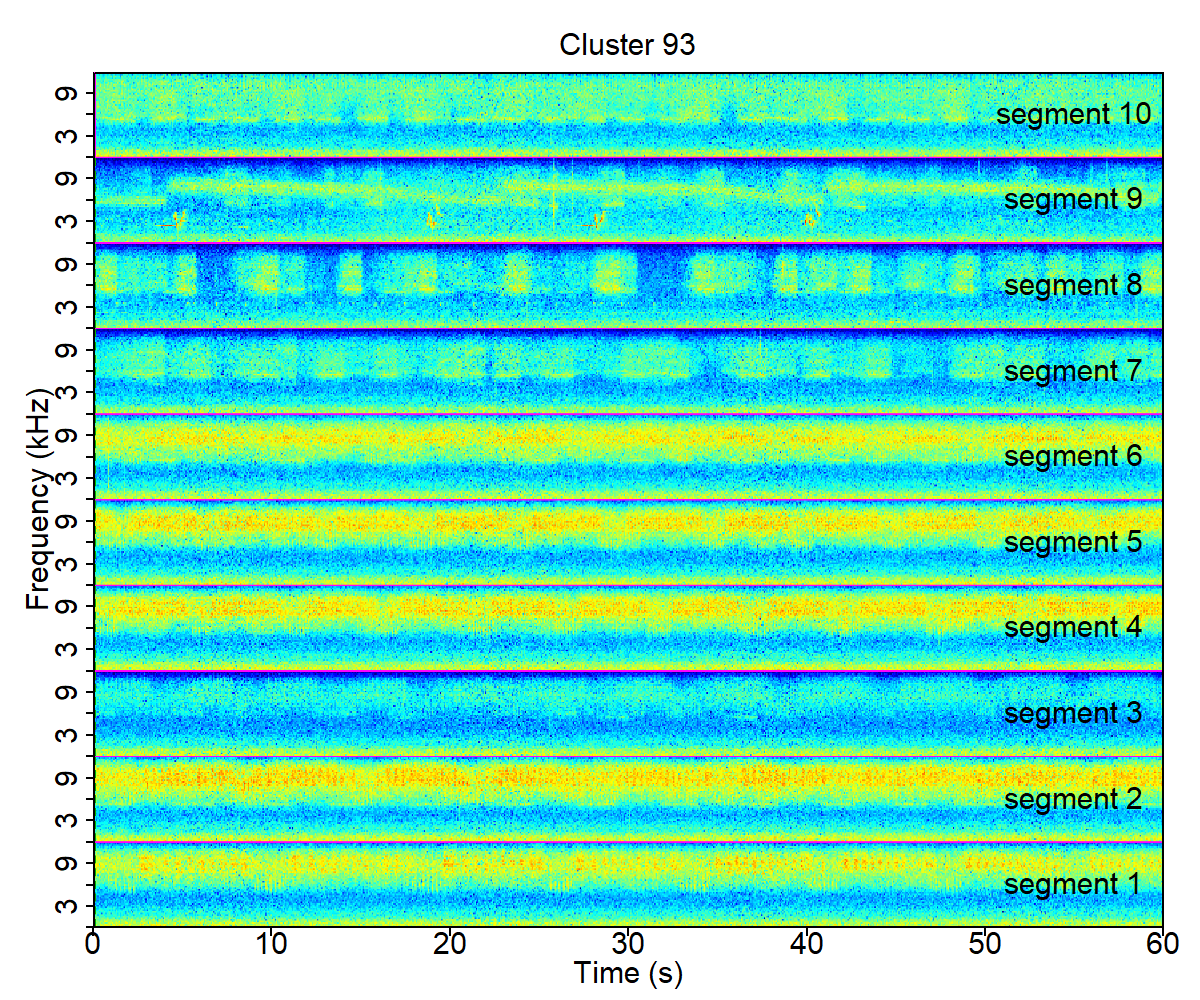

Supplement: Supplemental Information 7 — The spectrograms were computed using a Hann window, FFT = 512, window overlap of 50%, and frame size of 100%. The X-axis represents time, the Y-axis represents frequency. There are 10 audio segments for each cluster. [file peerj-11-16462-s007.zip › Supplemental_Information_S3_spec95_05/Cluster 93.png]
