# Supplementary figures and images for: VGGish-based detection of biological sound components and their spatio-temporal variations in a subtropical forest in eastern China (part 2 of 2)
Source: PeerJ. 2023 Nov 15;11:e16462. doi: 10.7717/peerj.16462 (PMC10656901; doi:10.7717/peerj.16462)

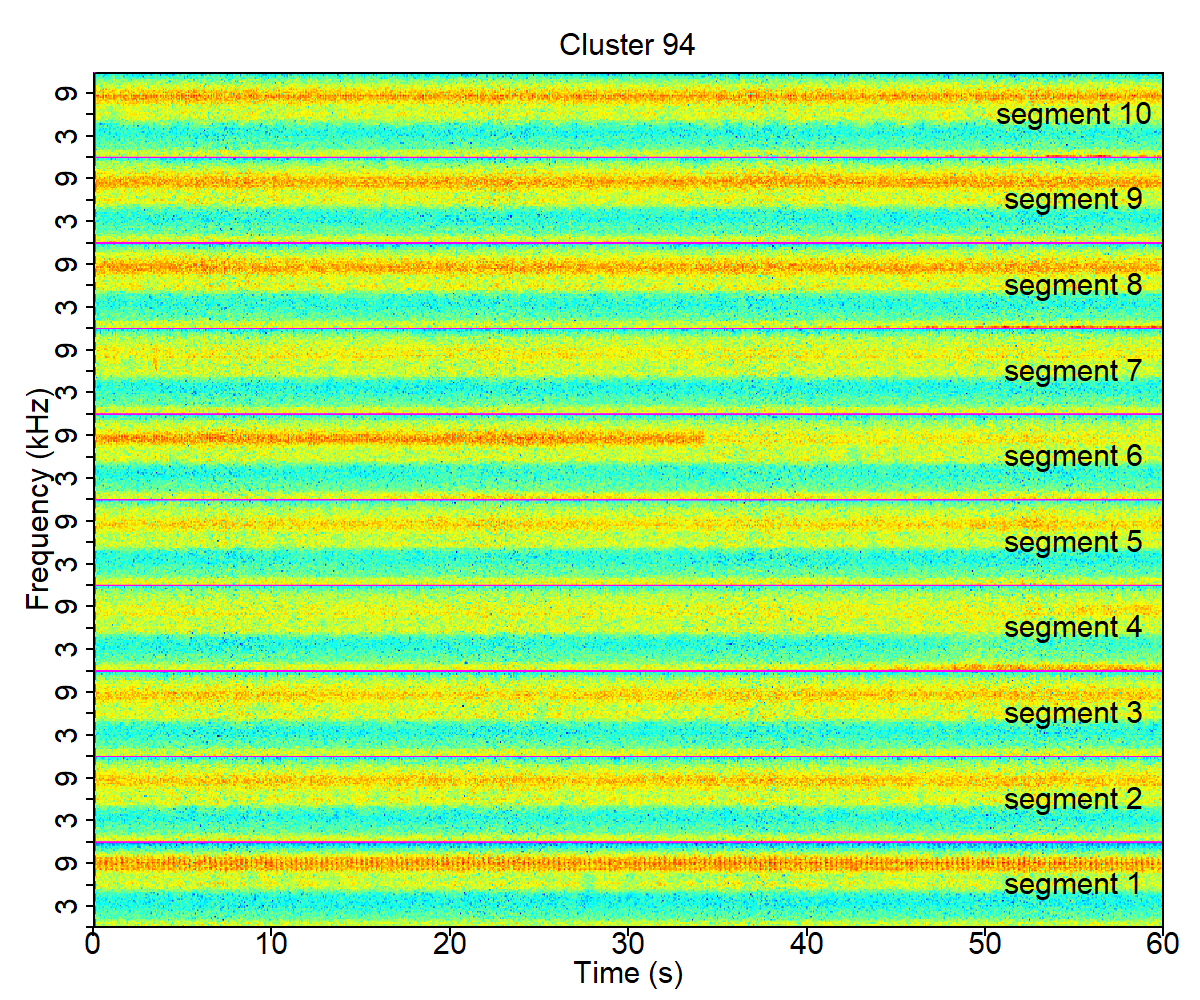

Supplement: Supplemental Information 7 — The spectrograms were computed using a Hann window, FFT = 512, window overlap of 50%, and frame size of 100%. The X-axis represents time, the Y-axis represents frequency. There are 10 audio segments for each cluster. [file peerj-11-16462-s007.zip › Supplemental_Information_S3_spec95_05/Cluster 94.png]
